# Direct amidation of esters with nitroarenes

**DOI:** 10.1038/ncomms14878

**Published:** 2017-03-27

**Authors:** Chi Wai Cheung, Marten Leendert Ploeger, Xile Hu

**Affiliations:** 1 Laboratory of Inorganic Synthesis and Catalysis, Institute of Chemical Sciences and Engineering, Ecole Polytechnique Fédérale de Lausanne (EPFL), ISIC-LSCI, BCH 3305, Lausanne 1015, Switzerland

## Abstract

Esters are one of the most common functional groups in natural and synthetic products, and the one-step conversion of the ester group into other functional groups is an attractive strategy in organic synthesis. Direct amidation of esters is particularly appealing due to the omnipresence of the amide moiety in biomolecules, fine chemicals, and drug candidates. However, efficient methods for direct amidation of unactivated esters are still lacking. Here we report nickel-catalysed reductive coupling of unactivated esters with nitroarenes to furnish in one step a wide range of amides bearing functional groups relevant to the development of drugs and agrochemicals. The method has been used to expedite the syntheses of bio-active molecules and natural products, as well as their post-synthetic modifications. Preliminary mechanistic study indicates a reaction pathway distinct from conventional amidation methods using anilines as nitrogen sources. The work provides a novel and efficient method for amide synthesis.

## Abstract (web summary)

Direct conversion of esters to amides, while attractive, is often limited to activated esters or highly nucleophilic amines. Here the authors report a nickel-catalysed reductive coupling between unactivated esters and nitroarenes, giving a direct route to aromatic amides.

Esters are common in nature and have found widespread applications in industry^1^. In nature, esters are responsible for the aroma of many flowers and fruits, and fats are generally triesters. In industry, polyesters, such as polyethylene terephthalate and cellulose acetate, are produced in millions-tons scale every year. Esters are important compounds in medicine, pharmacology, and flagrance as well. Ester linkages can be found in top-selling drugs, such as Tamiflu, Lovaza, Methylphenidate and Fenofibrate^2^. Fragrance additives applied in flavor and perfume industries, such as citronellyl formate and geranyl acetate, also contain ester groups. Compared to other carbonyl groups such as ketones, aldehydes and carboxylic acids, esters are more stable, which leads to applications as solvents and as protecting groups for carboxylic acids.

Because esters are ubiquitous synthetic intermediates, bio-active molecules and materials, direct conversion of the ester group into other functional groups is attractive in organic synthesis. A particularly important conversion is the transformation of esters into amides^3^,^4^, which are key components of peptides and proteins, as well as privileged molecules in medicinal chemistry^5^, as exemplified in some top-selling drugs such as Harvoni, Januiva, Glivec and Symbicort^6^. Direct amidation of esters with amines typically requires stoichiometric amounts of promoters or metal mediators^7^,^8^,^9^,^10^ (Fig. 1a). Catalytic methods are rare^11^,^12^ and notable examples include a Zr(O*t*-Bu)_4_-HOAt system developed by Porco and co-workers (HOAt=1-hydroxy-7-azabenzotriazole)^13^ and a Ru-pincer system developed by Milstein and co-workers^14^. These reactions proceed via nucleophilic substitution at the acyl carbon and mostly work with strongly nucleophilic alkyl amines, while anilines are often not suitable reaction partners. Garg and co-workers recently reported nickel-catalysed direct amidation of esters with secondary aryl amines to furnish tertiary anilides^15^ (Fig. 1b). A stoichiometric amount of aluminum *tert*-butoxide (Al(O*t*-Bu)_3_) was needed to shift the thermodynamic equilibrium towards amide formation and to facilitate the kinetics by carbonyl activation. While density functional theory computations suggested that these amidation reactions proceeded via an unusual oxidative addition of the acyl carbon-oxygen bonds of esters, the scope of esters is largely limited to those containing a naphthyl group, which are specialized and activated substrates compared to conventional dialkyl and alkyl aryl esters.

Here, we describe a nickel-catalysed reductive coupling method for the direct amidation of esters with nitroarenes (Fig. 1c). Nitroarenes offer several advantages over anilines as nitrogen sources: (i) nitroarenes are generally cheaper and more stable; (ii) as anilines are often prepared by hydrogenation of nitroarenes^16^, direct use of nitroarenes is more step-economical; further, substrates containing reducible groups, such as alkenes and nitriles, can be tolerated. (iii) Functional groups reactive towards anilines such as alkyl halide and ketones can be tolerated. Using the optimized protocols, numerous unactivated alkyl and aryl esters are converted into amides in one step. The method tolerates a large array of functional groups important for drug discovery such as dibenzothiazepinone and dibenzooxazepinone heterocycles. The method is used for expedited syntheses of intermediates to a handful of drugs and bio-active molecules. It is also successfully applied for the final step of the synthesis of a complex natural product, (-)-rhazinilam. The utility of the method is further demonstrated in the one-step amidation of a drug and a herbicide containing each an ester group. Preliminary mechanistic study suggests a unique reaction pathway involving azoarene as a key intermediate.

## Results

### Reaction design

**Figure 2:**
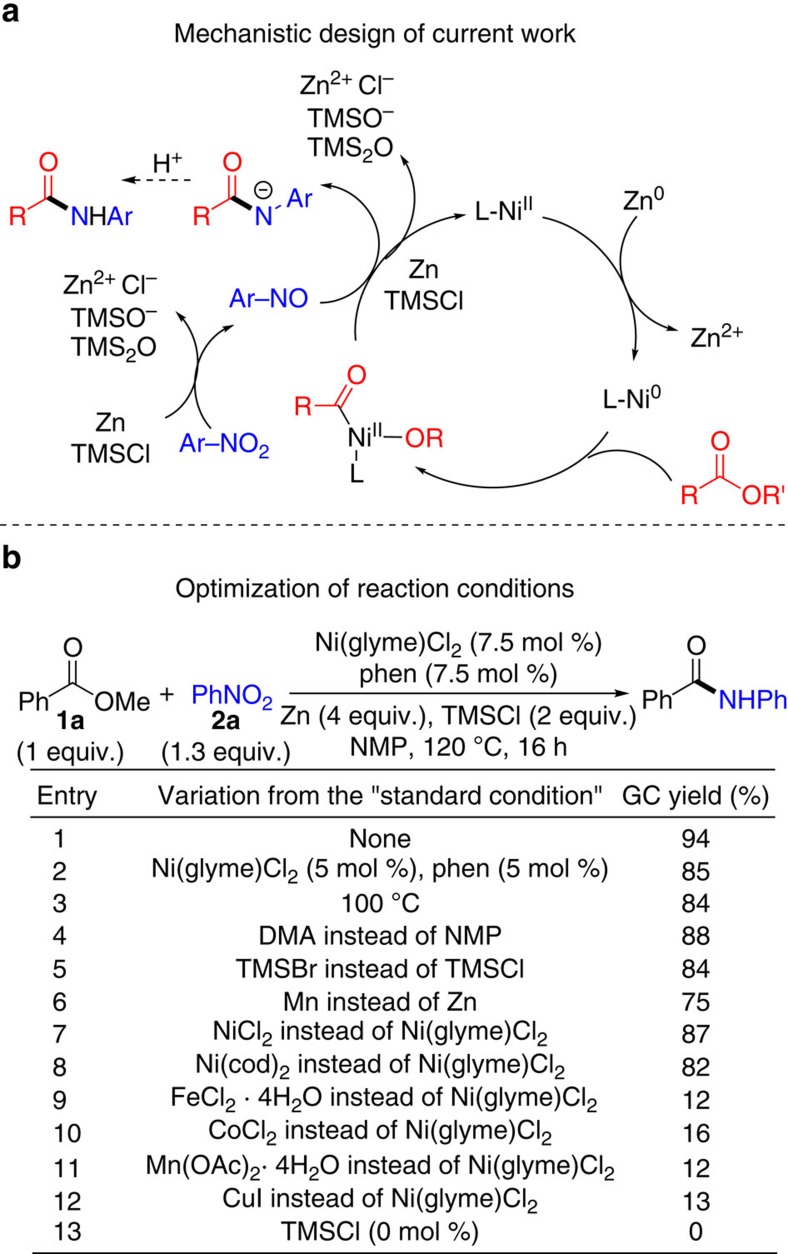
Design and optimization of direct amidation of esters with nitroarenes. (**a**) Mechanistic design for nickel-catalysed amidation of ester with nitroarene. (**b**) Optimization of nickel-catalysed amidation of ester with nitroarene. Ar, aryl; DMA, dimethylacetamide; L, ligand; Me, methyl; Ni, nickel (pre)catalyst; phen, 1,10-phenanthroline; Ph, phenyl; TMSBr, bromotrimethylsilane; TMSCl, chlorotrimethylsilane; Zn, zinc.

We recently reported iron-catalysed reductive coupling of nitroarenes with alkyl halides to form secondary aryl alkyl amines^17^. To further expand the scope of this new carbon–nitrogen bond coupling methodology, we became interested in the coupling of nitroarenes with esters. Whereas alkyl halides readily undergo oxidative addition via single-electron transfer in the presence of a base metal catalyst, for example, iron and nickel^18^, esters are much more inert towards oxidative addition. Shi and co-workers reported a rare experimental demonstration of oxidative addition of aryl esters on Ni(0) *N*-heterocyclic carbene complexes^19^, while Garg, Houk and co-workers described computational evidence for Ni-catalysed activation of acyl C–O bonds of methyl naphthoates^15^. These previous reports suggest that oxidative addition of the more inert unactivated methyl esters on a nickel centre is possible, even though experimental evidence for such a process is still lacking. With this consideration in mind, we envisioned the following pathway for reductive coupling of an ester with a nitroarene (Fig. 2a): A nickel(II) precatalyst (L-Ni^II^) is reduced by zinc (Zn) to nickel(0), which activates an ester to form a nickel(II) acyl complex. Meanwhile, in the presence of a Lewis acid, chlorotrimethylsilane (TMSCl), a nitroarene is reduced by zinc form a nitrosoarene^17^,^20^, as indicated in the Fe-catalysed reductive coupling of nitroarenes with alkyl halides^17^. In the presence of Zn and TMSCl, the nickel acyl complex might react with the nitrosoarene to form an amide anion, which is further converted to amide upon protonolysis. Silyl ether (TMS_2_O), and zinc(II) chlorides and siloxides are the expected by-products. Although such an amide formation reaction has yet to be reported, we were encouraged by the successful amine formation from alkyl halides and nitrosoarenes under Fe-catalysed reductive coupling conditions^17^.

### Screening of reaction conditions

The reaction of an aryl alkyl ester, methyl benzoate (**1a**), with nitrobenzene (**2a**) was used as the test reaction. After the screening of reaction parameters (Fig. 2b and Supplementary Tables 1–10), the optimized conditions were found involving the use of *N*-methylpyrrolidone (NMP) as solvent, nickel(II) chloride ethylene glycol dimethyl ether complex (Ni(glyme)Cl_2,_ 7.5 mol%) as catalyst, 1,10-phenanthroline (phen, 7.5 mol%) as ligand, Zn (4 equiv.) as reductant, and TMSCl (2 equiv.) as additive (Fig. 2b, entries 1–6). The optimal loading of nitrobenzene was 1.3 equiv. The reaction was completed after 16 h at 120 °C. After an acidic workup, the desired amide product, *N*-phenyl benzoate, was obtained in 94% yield (Fig. 2b, entry 1). Among various nickel salts, Ni(glyme)Cl_2_ was the optimal catalyst (Fig. 2b, entries 7 and 8). When Ni(glyme)Cl_2_ was replaced by catalysts based on iron, cobalt, manganese and copper, the yields were much lower (Fig. 2b, entries 9–12). Without TMSCl but under otherwise optimal conditions, no product was formed (Fig. 2b, entry 13). When a dialkyl ester, methyl decanoate (**1b**), was used (Supplementary Table 11), the optimal reaction conditions were found to be the same, except that a slightly lower loading of **2a** (1.2 equiv.) and a lower temperature (90 °C) were sufficient, affording *N*-phenyl decanoate quantitatively (Supplementary Table 11, entry 1).

### Scope

**Figure 3:**
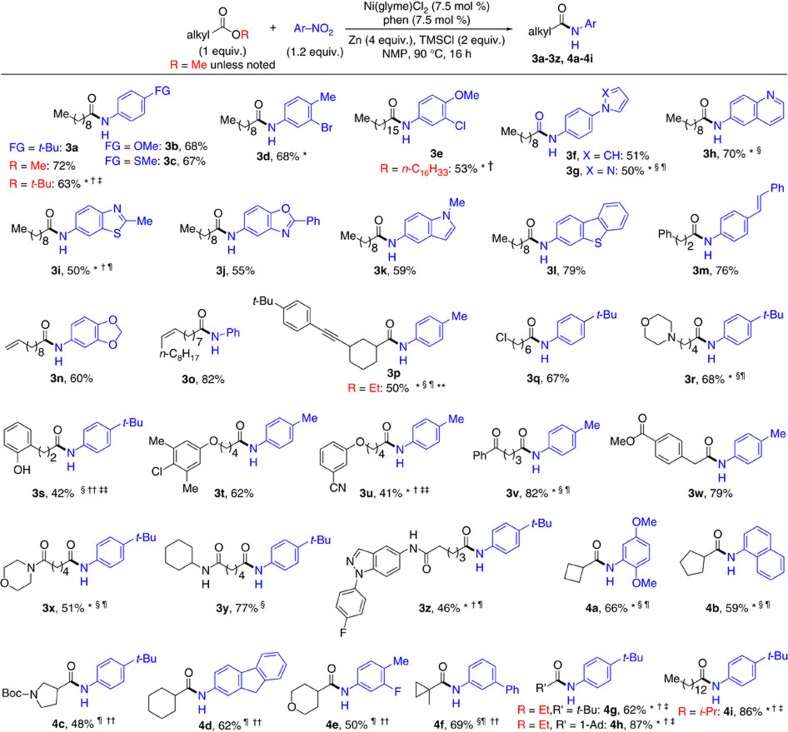
Amide synthesis via reductive coupling of alkyl alkanoate with nitro(hetero)arene. Unless otherwise noted, methyl alkanoates were used (R=Me), and the optimized conditions as shown in the equation were applied; the data are reported as isolated yield. See Supplementary Materials for experimental details. Boc, *tert*-butyloxycarbonyl; *i*-Pr, isopropyl; Me, methyl; Ph, phenyl; *t*-Bu, *tert*-butyl; 1-Ad, 1-adamantyl. *ArNO_2_ (1.5 equiv). ^†^Ni(glyme)Cl_2_ (15 mol%), phen (15 mol%). ^‡^140 °C; Mn (5 equiv.) and TMSI (2 equiv.) were used instead of Zn and TMSCl. ^§^Ni(glyme)Cl_2_ (10 mol%), phen (10 mol%). ^¶^120 °C. **Diastereomer ratio (d.r.) of product∼4:1. ^††^ArNO_2_ (1.3 equiv). ^‡‡^140 °C.

**Figure 4:**
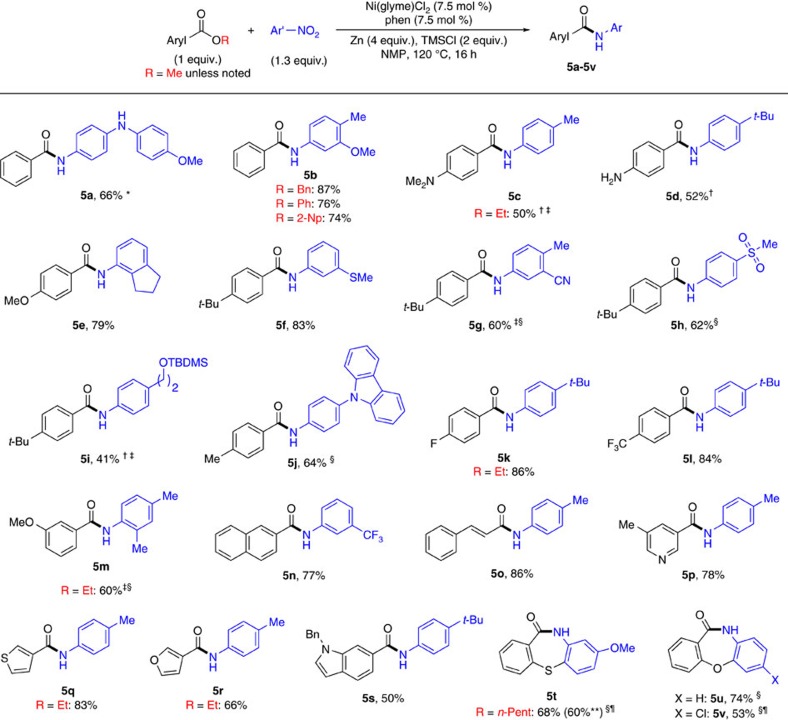
Amide synthesis via reductive coupling of alkyl arenoate with nitroarene. Unless otherwise noted, methyl arenoates were used (R=Me), and the optimized conditions as shown in the equation were applied; the data are reported as isolated yield. See Supplementary Materials for experimental details. Bn, benzyl; Et, ethyl; Me, methyl; Ph, phenyl; TBDMS, *tert*-butyldimethylsilyl; *t*-Bu, *tert*-butyl; 2-Np, 2-naphthyl; *n*-Pent=*n*-pentyl. *ArNO_2_ (1.2 equiv.). ^†^Ni(glyme)Cl_2_ (15 mol%), phen (15 mol%). ^‡^ArNO_2_ (1.5 equiv.). ^§^Ni(glyme)Cl_2_ (10 mol%), phen (10 mol%). ^¶^90 °C. **Gram scale synthesis using 2.93 mmol of substrate.

The optimal conditions were applicable for the coupling of numerous esters and nitro(hetero)arenes. The scope for the coupling of alkyl esters is shown in Fig. 3, while the scope for the coupling of aryl esters is shown in Fig. 4. Non-functionalized alkyl alkanoates bearing primary (**3a, 3e**), secondary (**4a, 4b** and **4d**), and tertiary (**4f–4h**) alkyl groups as well as aromatic rings (**3m**) reacted to give the corresponding amides in high yields. Functionalized alkyl alkanoates also reacted smoothly, tolerating a wide range of groups such as alkene (**3n, 3o**), alkyne (**3p**), chloroalkane (**3q**), amine (**3r**), phenol (**3s**), chloroarene (**3t**), nitrile (**3u**), keto (**3v**), ester (**3w**), tertiary amide (**3x**), secondary amide (**3y, 3z**), carbamate (**4c**) and ether (**4e**). Similarly, alkyl arenoates bearing substituents with various electronic properties could be amidated. Electron-neutral phenyl group (**5a**), electron-rich aromatic groups (amine (**5c, 5d**), *p*-methoxy (**5e**), *tert*-butyl (**5f**), methyl (**5j**), naphthyl (**5n**)), and electron-deficient aromatic groups (fluoro (**5k**), trifluoromethyl (**5l**), *m*-methoxy (**5m**)) all could be incorporated in the ester coupling partners. Esters containing potentially coordinating groups such as cinnamide (**5o**), pyridyl (**5p**), thienyl (**5q**), furyl (**5r**) and indolyl (**5s**) also reacted smoothly to give the amides in high yields. Various alkyl groups attached to the oxygen atom of the ester linkage were tolerated, including non-activated methyl, ethyl (e.g., **3p, 5c**), pentyl (**5t**), and hexadecyl groups (**3e**), as well as the benzyl group (**5b**). Phenyl and naphthyl esters could also be coupled (**5b**). The use of more sterically encumbered esters bearing *tert*-butyl (**3a, 4g**), adamantyl (**4h**), and isopropyl groups (**4i**) required manganese (Mn) as reductant in place of zinc and iodotrimethylsilane (TMSI) as additive in place of TMSCl in order to afford the amides in high yields. Moreover, the coupling method is insensitive to the electronics of nitroarenes (Figs 3 and 4), as nitrobenzene (**3o**) and its derivatives containing electron-donating (**3a**–**3c, 3m, 3p, 4d, 5a**) or electron-withdrawing groups (**3d**, **3e, 4e, 5f–5h, 5n**) all reacted to yield the corresponding amides in synthetically useful yields. Functional groups such as thiomethyl (**3c, 5f**), bromo (**3d**), chloro (**3e**), olefin (**3m**), fluoro (**4e**), amino (**5a**), nitrile (**5g**), sulfonyl (**5h**), protected alcohol (**5i**), and trifluoromethyl (**5n**) groups were all tolerated on the nitroarene coupling partners. Nitroarenes with sterically encumbered 2,5-dimethoxyphenyl (**4a**), 1-naphthyl (**4b**), indanyl (**5e**), and 2,4-xylyl (**5m**) were suitable reaction partners as well. Importantly, nitroheteroarenes were successfully coupled, giving rise to various heteroaryl amides containing pyrrole (**3f**), pyrazole (**3g**), quinoline (**3h**), benzothiazole (**3i**), benzoxazole (**3j**), indole (**3k**), benzothiophene (**3l**), benzodioxole (**3n**), and carbazole (**5j**) moieties. Notably, the intramolecular reductive coupling of alkyl arenoates with nitroarenes was successful, and seven-membered cyclic amides, dibenzothiazepinone (**5t**) and dibenzooxazepinone (**5u**, **5v**), were synthesized in this way. Gram-scale synthesis could be achieved without a significant loss in yield (for example, for **5t**).

### Comparison with amidation of esters with anilines

**Figure 5:**
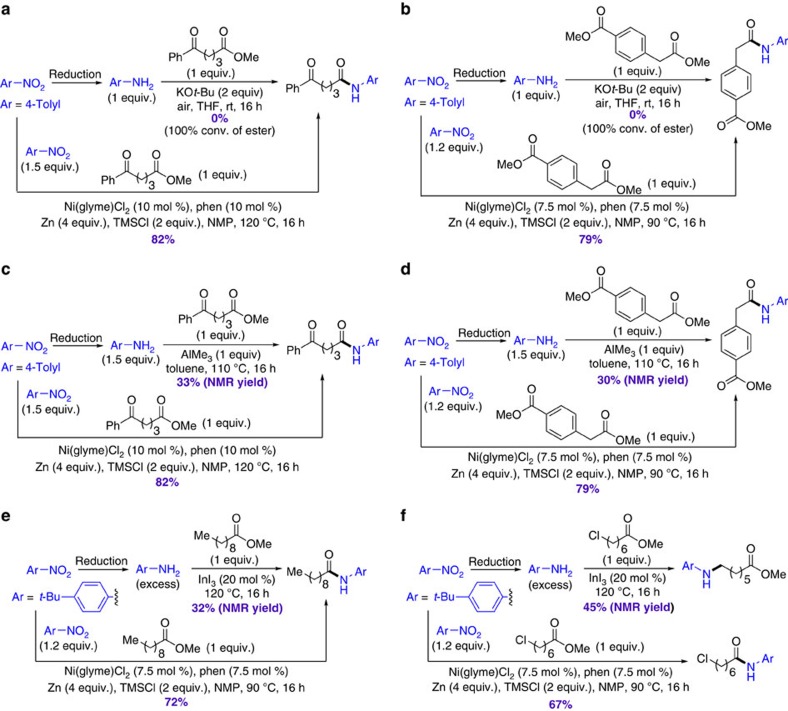
Comparison of nickel-catalysed reductive coupling method and classical amidation methods. (**a**,**b**) Comparison with base-promoted amidation of ester with aniline. (**c**,**d**) Comparison with aluminum-mediated amidation of ester with aniline. (**e**,**f**) Comparison with indium-catalysed amidation of ester with aniline. AlMe_3_, trimethylaluminum; InI_3_, indium(III) triiodide; KO*t*-Bu, potassium *tert*-butoxide; Me, methyl; THF, tetrahydrofuran.

Amidation of esters with anilines is analogous to the current reductive amidation method, so a number of direct comparison experiments were conducted (Fig. 5; Supplementary Fig. 1). Three different protocols for amidation of esters with anilines were employed: base promotion^8^, trimethylaluminum-mediation^7^ and indium catalysis^21^. The base-promoted amidation of esters with anilines is incompatible with base-sensitive and enolizable functional groups such as ketone and ester groups, as shown in Fig. 5a,b. On the other hand, our reductive amidation method is compatible with such functional groups, leading to high amidation yields (Fig. 5a,b). Moreover, the base-promoted amidation with base-sensitive functional groups, such as fluoroaryl and enones, only gave the amides in modest yields (∼50%), while our reductive amidation methods tolerates these groups, giving the amides in high yields (86%, Supplementary Figs 1a and b). The trimethylaluminum-mediated amidation of esters with anilines gave modest yields of amides (∼30%) when substrates have acid-sensitive carbonyl groups (Fig. 5c,d). The current reductive amidation method tolerated these groups, and gave the desired amides in much higher yields (∼80%, Fig. 5c,d). Figure 5e shows an example where the indium-catalysed amidation of a simple ester with an excess of aniline only afforded the amide in a modest yield, while the current method gave the amide in a high yield (72%, Fig. 5e). Because the current amidation method employs nitroarenes as starting reagents instead of anilines, an important advantage is that substrates containing groups that are prone to undesirable side reactions with anilines could be used. For example, methyl 7-chloroheptanoate, which contains an alkyl chloride group susceptible to nucleophilic attack by aniline, reacted with an aniline to give an undesired alkylation product in 45% yield (Fig. 5f). No amidation was observed. In contrast, the current method allowed the selective amidation of methyl 7-chloroheptanoate in a 67% yield, without the alkylation byproduct (Fig. 5f).

### Application

**Figure 6:**
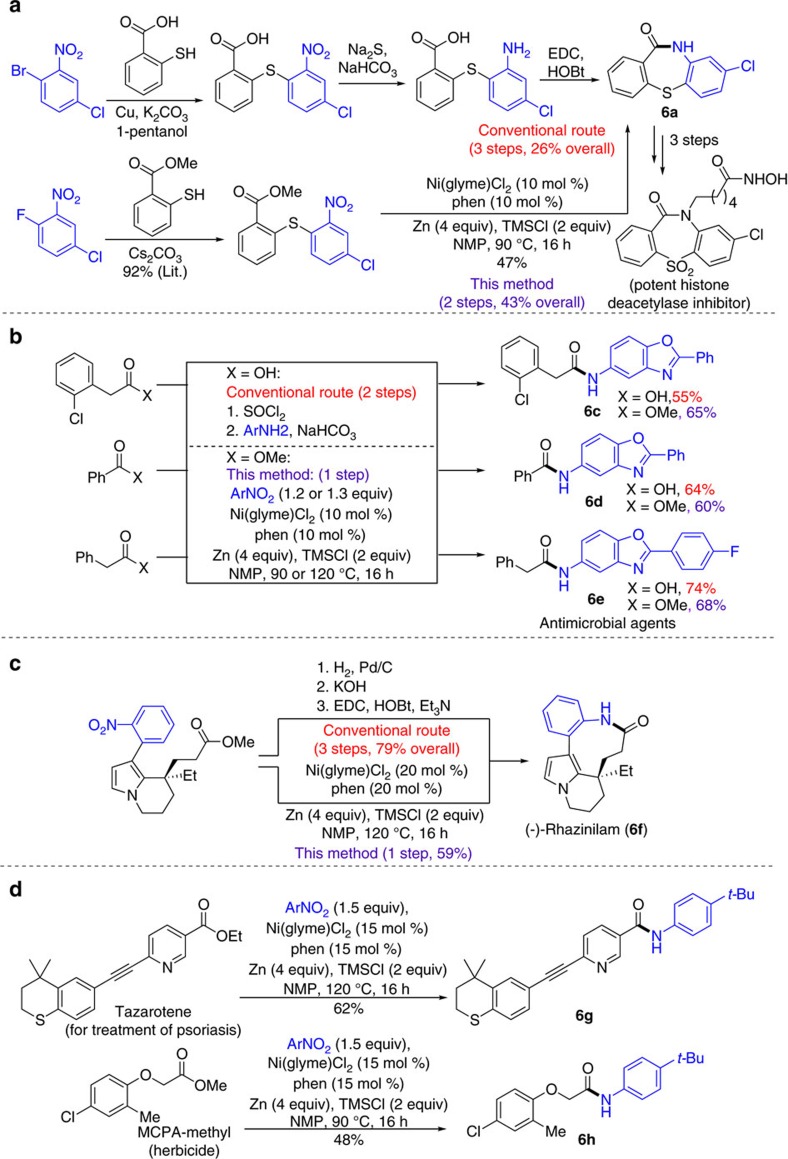
Application in synthesis of bio-active molecules. (**a**) Efficient synthesis of the key intermediate of a potential antitumor agent; (**b**) single-step synthesis of antimicrobial agents; (**c**) single-step synthesis of a natural product; (**d**) modification of ester-containing drug and herbicide. EDC, *N*-ethyl-*N*′-(3-dimethylaminopropyl)carbodiimide; Et, ethyl; Et_3_N, triethylamine; HOBt, 1-hydroxybenzotriazole Lit., reported yield from literature; Me, methyl; Ph, phenyl.

To demonstrate its potential applications in medicinal and agro chemistry, this amidation method was applied for the expedite syntheses of drugs, bioactive molecules, their key intermediates, and a natural product (Fig. 6). The method was applied for the synthesis of **6a**, an intermediate to a potent histone deacetylase inhibitor^22^, in two steps with a 43% overall yields from commercially available starting materials (Fig. 6a). For comparison, the previous method based on the coupling of benzoic acid with aniline required three steps and had a 26% overall yield^22^ (Fig. 6a). Similarly, dibenzothiazepinone **6b**, an intermediate to an approved atypical antipsychotic drug, quetiapine^23^, was synthesized by our method in two steps with a 54% overall yield (Supplementary Fig. 2). Previous method based on the amidation of ester with aniline required four steps and had a comparable yield^23^ (Supplementary Fig. 2). The amidation method was also applied to synthesize antimicrobial agents, **6c**, **6d** and **6e** (refs 24, 25), in one step from esters and nitroheteroarenes (Fig. 6b). The conventional amidation method, based on reactions of carboxylic acids and anilines using thionyl chloride as the coupling reagent, required two steps^24^,^25^ (Fig. 6b). Importantly, our method allowed a single-step synthesis of a natural product, (-)-rhazinilam (**6f**), from methyl (*R*)-3-(8-ethyl-1-(2-nitrophenyl)-5,6,7,8-tetrahydroindolizin-8-yl)propanoate^26^ (Fig. 6c). Previous amidation from the same intermediate relied on the reaction of carboxylic acid with aniline, which required two extra steps for hydrogenation of nitroarene and hydrolysis of ester^26^.

Because ester is a common functional group in pharmaceuticals and agrochemicals, our direct amidation method provides a new possibility of rapid post modification of such compounds. As an example, Tazarotene^27^, a retinoid approved for the treatment of psoriasis, has an ester group. This compound was converted into an amide derivative (**6g**) in one-step with a 62% yield using our method (Fig. 6d). Likewise, MCPA-methyl^28^, a common herbicide, was converted in one-step to an amide (**6h**) using this method (Fig. 6d).

### Mechanistic study

**Figure 7:**
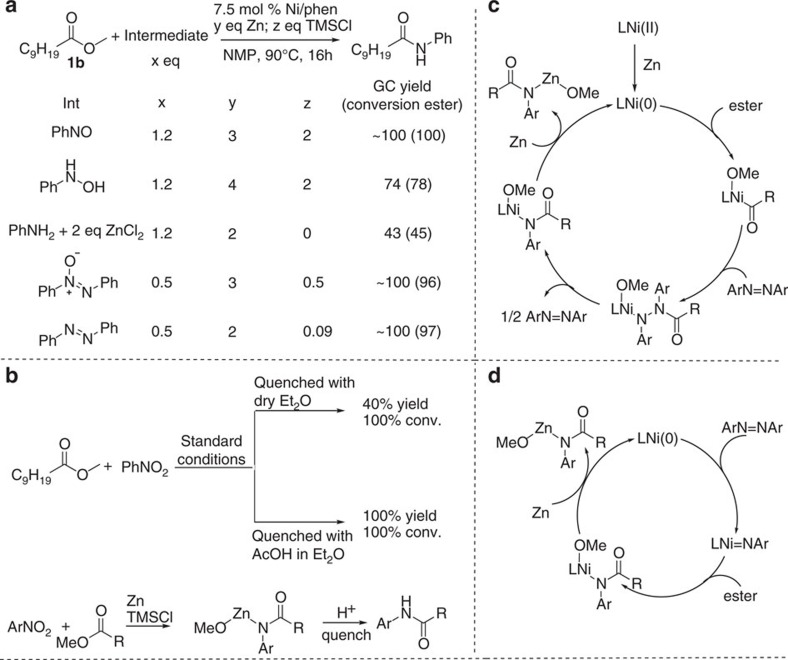
Mechanistic study and hypotheses. (**a**) Reactivity of several potential intermediates. (**b**) Quenching experiments to probe the source of proton. (**c**) Proposed catalytic cycle assuming ester activation. (**d**) Proposed catalytic cycle assuming nitrene formation. Ni, Ni(glyme)Cl_2_.

Several experiments were conducted to gain insight into the mechanism of the reaction. Under reductive conditions, nitrobenzene could be converted to nitrosobenzene, phenylhydroxyamine or aniline, as well as to azoxybenzene and azobenzene^29^, all of which could in principle be intermediates in the formation of amides. Their reactivity under catalytic conditions with methyl decanoate (**1b**) was investigated. The optimal conditions and their corresponding yields are depicted in Fig. 7a (see Supplementary Tables 12–15 for details). Aniline gave poor yields regardless of the conditions, excluding it as an intermediate in the main reaction pathway. A reasonable yield was obtained with phenylhydroxylamine, but reducing the amount of Zn and TMSCl from the standard conditions diminished the yields. As a certain amount of reductant should be consumed to form the phenylhydroxylamine, it is unlikely that phenylhydroxylamine is the predominant intermediate that reacts with the ester. Good yields were obtained with nitrosobenzene, azoxybenzene and azobenzene. Importantly, as these intermediates become more reduced (from nitrosobenzene to azoxybenzene to azobenzene), fewer equivalents of reductant are required to make the reaction work. This result suggests nitrosobenzene as a precursor to form azoxybenzene, which is subsequently reduced to azobenzene, before reacting to form the amide. Indeed, when only 2 equivalents of Zn and 1 equivalent of TMSCl were used with nitrosobenzene as starting material, both azobenzene and azoxybenzene could be observed among the products. Likewise, when insufficient reductant is added to the reaction with azoxybenzene as starting material, azobenzene can be observed. These results indicate that azobenzene is the most likely intermediate for the amidation.

When the reaction was quenched with dry diethyl ether (Et_2_O) and subjected to GC analysis, a yield of only 40% was obtained although the conversion was 100% (Fig. 7b). When the reaction was quenched with a solution of acetic acid in Et_2_O, both the conversion and yield were about 100%. This result suggests that the product of the reaction is a zinc amide before quenching with a proton source in the workup.

Based on the above results, a simple reaction sequence could be proposed for the amidation reactions. Under the reaction conditions, nitrobenzene is reduced by Zn, with the aid of TMSCl, to form azobenzene. The Ni(II) catalyst is reduced by Zn to Ni(0). Under Ni(0) catalysis, ester reacts with azobenzene to form a nickel amidate. Transmetalation to zinc followed by protonolysis furnishes the amide product. This sequence bears some resemblance to Ni-catalysed reductive coupling of carbon electrophiles with isocyanates to form amides^30^,^31^.

When the ester **1b** was treated with an equal amount of Ni(cod)_2_ and 1,10-phenanthroline at 90 °C, no appreciable reaction could be observed by ^1^H and ^13^C NMR. Replacing Ni(cod)_2_ with Ni(glyme)Cl_2_ and Zn (activated with 1 mol% of TMSCl) resulted in no ester activation neither. Thus, the Ni(0) species in our system either could not activate the ester, or the reaction was thermodynamically uphill and could not be observed in a stoichiometric reaction. When azobenzene was treated with two equivalents of the system composed of Ni(glyme)Cl_2,_ 1,10-phenanthroline, and Zn (activated with 1 mol% of TMSCl) at 90 °C, a reaction seemed to occur but it was difficult to follow by ^1^H NMR due to many overlapping peaks. 4,4-Difluoro azobenzene was then used as the substrate, and the reaction was followed by ^19^F NMR. The substrate was converted into a new species with a broad ^19^F signal at −132 p.p.m.. Attempts to identify or isolate this species were unsuccessful. Addition of ester **1b** to the reaction mixture and subsequent reaction at 90 °C then converted this species into the corresponding amide. These observations suggest that the Ni(0) species can activate azobenzene, and the activation might be productive for the overall amidation. For some unknown reasons, if Ni(glyme)Cl_2_ and Zn were replaced by Ni(cod)_2_ in the reaction with 4,4-difluoro azobenzene, no amide formation was observed upon addition of ester.

## Discussion

The reactivity of esters depends on the nature of esters. While primary alkyl esters generally react smoothly at 90 °C, the reactions of secondary and tertiary alkyl esters as well as aryl esters require higher temperatures (120–140 °C) (Figs 3 and 4). An illustrative example is the reaction of methyl 4-(2-methoxy-2-oxoethyl)benzoate (**3w**), where the alkyl ester groups reacted to give predominantly the alkyl amide, while no product originated from aryl ester activation was formed. This reactivity trend might originate from the steric properties of esters. To further probe the relative reactivity of esters, a mixture of two esters with different steric properties was reacted with a nitroarene (Supplementary Fig. 3). It was found that (1) primary alkyl ester reacted faster than aryl ester (Supplementary Fig. 3a); (2) aryl ester reacted faster than secondary alkyl ester (Supplementary Fig. 3b); (3) primary alkyl ester reacted much faster than secondary alkyl ester (Supplementary Fig. 3c). These results are consistent with the steric properties of esters, whose bulkiness has the order of primary alkyl ester<aryl ester<secondary alkyl ester. The trend of reactivity does not follow the electronic properties of esters.

While the current reactions are compatible with a wide range of nitro(hetero)arenes, nitroalkanes do not react to give any amide products despite complete conversion. It is likely that the nitrosoalkane intermediates are less stable than their aryl counterparts and undergo isomerization to give oximes^32^,^33^,^34^, which rapidly dissociate into aldehydes/ketones and hydroxylamine without further reaction to form azoalkanes or nitrenes.

Two different pathways for Ni(0)-catalysed reaction of azobenzene with ester may be envisioned. The first pathway involves oxidative addition of ester on Ni(0) to give Ni(II) acyl complex (Fig. 7c). A similar step has been proposed in several recent reports of Ni-catalysed activation of esters^15^,^19^. An analogous Ni(II) acyl complex was isolated in the reaction of a Ni(0) complex with 1-naphthoate esters^19^. Insertion of the acyl group into azobenzene then gives the Ni(II) hydrazido(−1) intermediate. Migratory insertion into azobenzene is well-known for metal hydrides^35^,^36^, and insertion of an alkyl moiety into azobenzene was reported before^37^. In analogy to the reactivity of an Fe(II) hydrazido(−1) complex reported by Holland and co-workers^38^, two molecules of the Ni(II) hydrozido(−1) intermediate are proposed to react with one another to give azobenzene and the Ni(II) amido intermediate. Under the reductive conditions, the Ni(II) amido intermediate is converted by Zn into Ni(0) to re-enter the catalytic cycle, with concomitant formation of the zinc amide.

Alternatively, azobenzene could react with Ni(0) to form a Ni(II) nitrene intermediate (Fig. 7d). This type of reactivity is known for early transition metals^39^ and was recently reported for iron^40^. Reaction of nitrene with ester via a yet unknown pathway leads to a Ni(II) amidate, which is then reduced by Zn to give a zinc amidate and regenerate the Ni(0) catalyst.

While experimental and computational evidence has been reported for oxidative addition of esters on a Ni(0) centre, the known examples are limited to Ni(0) complexes of electron-rich ligands such as *N*-heterocyclic carbene^15^,^19^. Moreover, only relatively activated substrates aryl esters and methyl naphthoates are reactive. In the current system, the 1,10-phenanthroline ligand is less electron-rich than *N*-heterocyclic carbene, while the substrates are unactivated esters. Both factors disfavour oxidative addition. Moreover, stoichiometric reaction of an ester with Ni(0) species provided no evidence for oxidative addition of ester in the current system. On the other hand, azobenzene reacted with Ni(0) species to give an intermediate, which could be converted into the amide product upon reaction with an ester. Thus, the amidation catalysis reported here is more likely initiated by activation of azobenzene on a Ni(0) species than by oxidative addition of ester. However, oxidative addition of ester cannot be fully ruled out because such a step might be thermodynamically uphill and escape observation. The intermediate formed upon reaction of azobenzene with Ni(0) might be a dormant, off-cycle species.

Although further study is required to elucidate the details of the mechanism for this direct amidation method, the available data point to a reaction pathway very distinct from the pathway of amidation ractions using anilines as the nitrogen source. As shown in Fig. 5, this unique pathway leads to improved scope and group tolerance of the current amidation method over the method of reacting esters with anilines for many substrates. On a practical level, the current method complements the classical method of coupling carboxylic acids with the anilines^41^,^42^,^43^,^44^. The latter requires coupling reagents, some of which are toxic. If a carboxylic acid is more readily available than its ester derivative, the coupling of carboxylic acid with aniline seems more convenient. If the ester derivative is easier to access, then the current amidation method is more advantageous. The examples in Fig. 6 demonstrates these advantages for the expedited syntheses of bio-active molecules and natural product.

In conclusion, a nickel-catalysed reductive coupling method has been developed for the direct amidation of numerous unactivated esters with nitroarenes. The method has broad substrate scope and high functional group tolerance. Complementarity with and considerable advantages over existing amidation methods have been demonstrated. The utility of the method is illustrated by the expedited syntheses of intermediates to a handful of drugs and bio-active molecules, application for the final step of the total synthesis of (-)-rhazinilam, and one-step post-synthetic modification of a drug and a herbicide.

## Methods

### General

Supplementary Figs 4–74 for the NMR spectra, Supplementary Tables 1–15 for the optimization of reactions, and Supplementary Methods for the characterization data can be found in the Supplementary Information.

### General procedure for direct amidation with nitroarenes

An oven-dried 30 ml re-sealable screw-cap test tube equipped with a Teflon-coated magnetic stir bar was sequentially charged with zinc powder (Zn, 4 equiv., 2.0 mmol, 131 mg), ester (1 equiv., 0.50 mmol), nitroarene (1.2 equiv., 0.60 mmol; 1.3 equiv., 0.65 mmol; 1.5 equiv., 0.75 mmol), 1,10-phenanthroline (phen, 7.5 mol %, 6.8 mg; 10 mol %, 9.0 mg; 15 mol %, 13.5 mg;), nickel(II) chloride ethylene glycol dimethyl ether complex (Ni(glyme)Cl_2_, 7.5 mol %, 8.3 mg; 10 mol %, 11.0 mg; 15 mol %, 16.5 mg;), *N*-methylpyrrolidone solvent (NMP, 1.0 ml), and chlorotrimethylsilane (TMSCl, 2 equiv., 1.0 mmol, 128 μl). The resulting mixture was stirred in a preheated oil bath (90–140 °C) for 16 h. After the reaction, the reaction mixture was cooled down to room temperature, and the crude product was acidified with saturated NH_4_Cl solution (∼5 ml) and then neutralized with saturated NaHCO_3_ solution (∼10 ml). The crude product in the aqueous fraction was extracted with EtOAc (∼20 ml). The aqueous fraction was further washed with EtOAc (3 × ∼10 ml). The combined organic fractions were concentrated *in vacuo* with the aid of a rotary evaporator. The crude product residue was purified by preparative thin-layer chromatography using a solvent mixture (dichloromethane, hexanes, and/or ethyl acetate) as an eluent to afford the purified amide product.

### Data availability

All data of the study are available from the authors upon reasonable request.

## Additional information

**How to cite this article:** Cheung, C. W. *et al.* Direct amidation of esters with nitroarenes. *Nat. Commun.* **8**, 14878 doi: 10.1038/ncomms14878 (2017).

**Publisher's note:** Springer Nature remains neutral with regard to jurisdictional claims in published maps and institutional affiliations.

## Supplementary Material

Supplementary Information Supplementary Figures, Supplementary Tables, Supplementary Methods and Supplementary References.

## Figures and Tables

**Figure 1:**
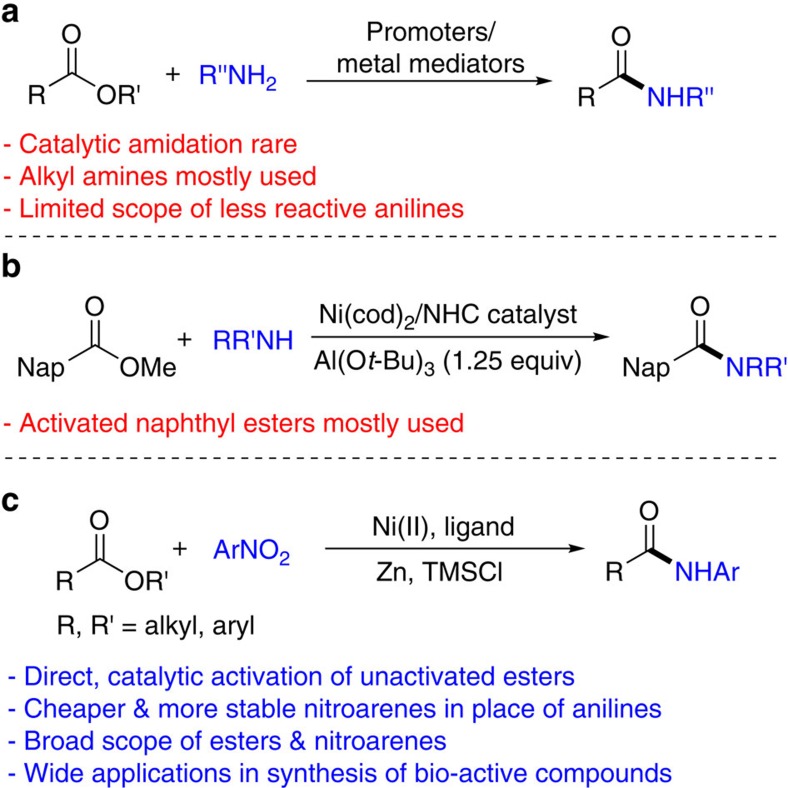
Different approaches for amidation of esters. (**a**) Amidation of esters with promoters or metal mediators; (**b**) nickel-catalysed amidation of naphthyl esters with secondary amines; (**c**) nickel-catalysed direct amidation of esters with nitroarenes. Al(O*t-*Bu)_3_, aluminum *tert*-butoxide; Ar, aryl; Me, methyl; Nap, naphthyl; NHC, *N*-heterocyclic carbene; Ni(cod)_2_, bis(1,5-cyclooctadiene)nickel(0); TMSCl, chlorotrimethylsilane; Zn, zinc.
