# Supplementary material for: Direct amidation of esters with nitroarenes
Source: Nat Commun. 2017 Mar 27;8:14878. doi: 10.1038/ncomms14878 (PMC5378957; doi:10.1038/ncomms14878)
Supplement: Supplementary Information — Supplementary Figures, Supplementary Tables, Supplementary Methods and Supplementary References. [file ncomms14878-s1.pdf]

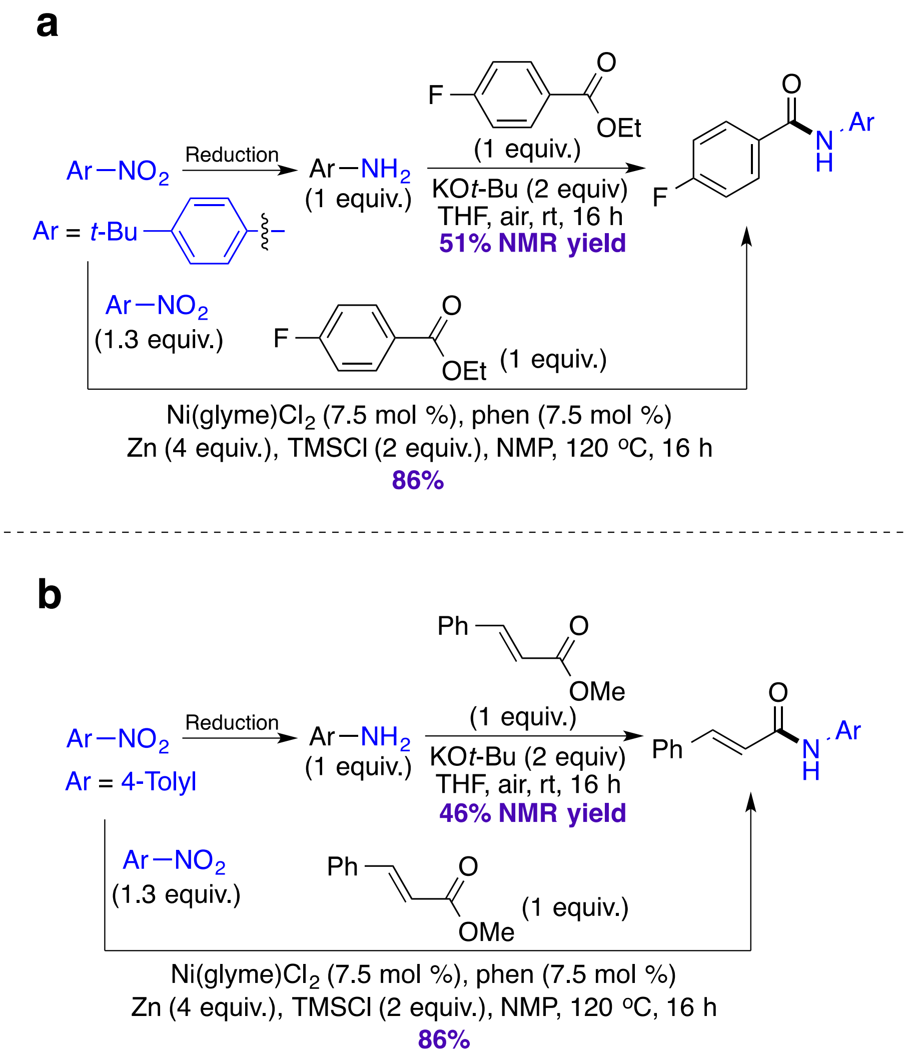

Supplementary Figure 1 (a) and (b). Comparison of nickel-catalyzed reductive coupling method and base-mediated amidation of esters with anilines. Et = ethyl; Me = methyl; THF = tetrahydrofuran; KO*t*-Bu = potassium *tert*-butoxide.

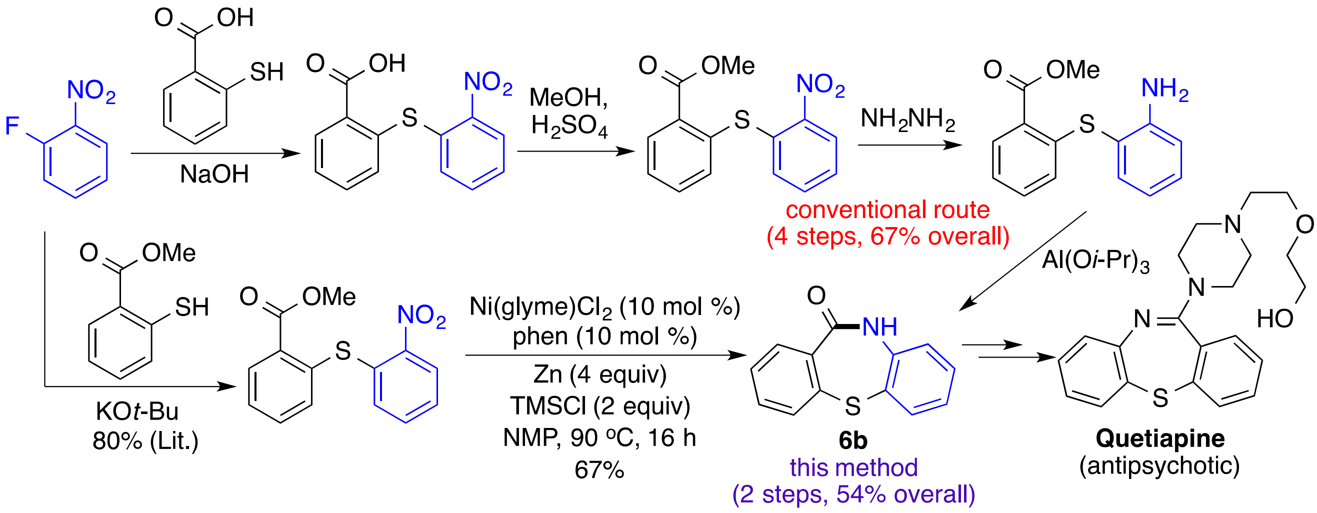

Supplementary Figure 2. Application in synthesis of the key intermediate of an antipsychotic drug. Lit. = reported yield from literature; Me = methyl; Al(O*i*-Pr)$_3$ = aluminum isopropoxide. EDC = *N*-ethyl-*N′*-(3-dimethylaminopropyl)carbodiimide; HOBt = 1-hydroxybenzotriazole.

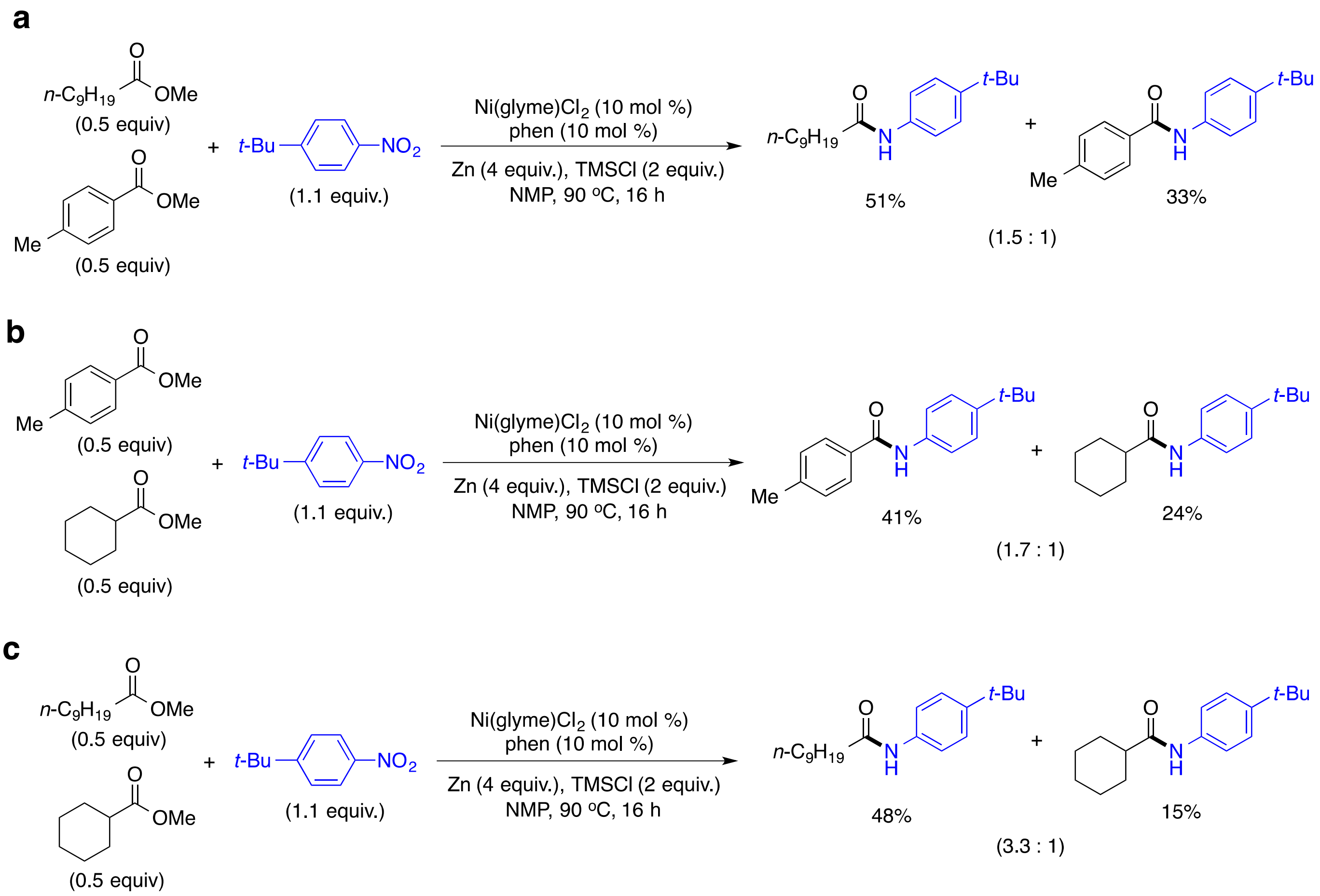

Supplementary Figure 3(a)-(c). Study of relationship between steric bulkiness of esters and relative reactivity of amidation. ^{1}H NMR yields were shown. The ratios of amide products were determined by ^{1}H NMR spectrometry. Me = methyl; *t*-Bu = *tert*-butyl.

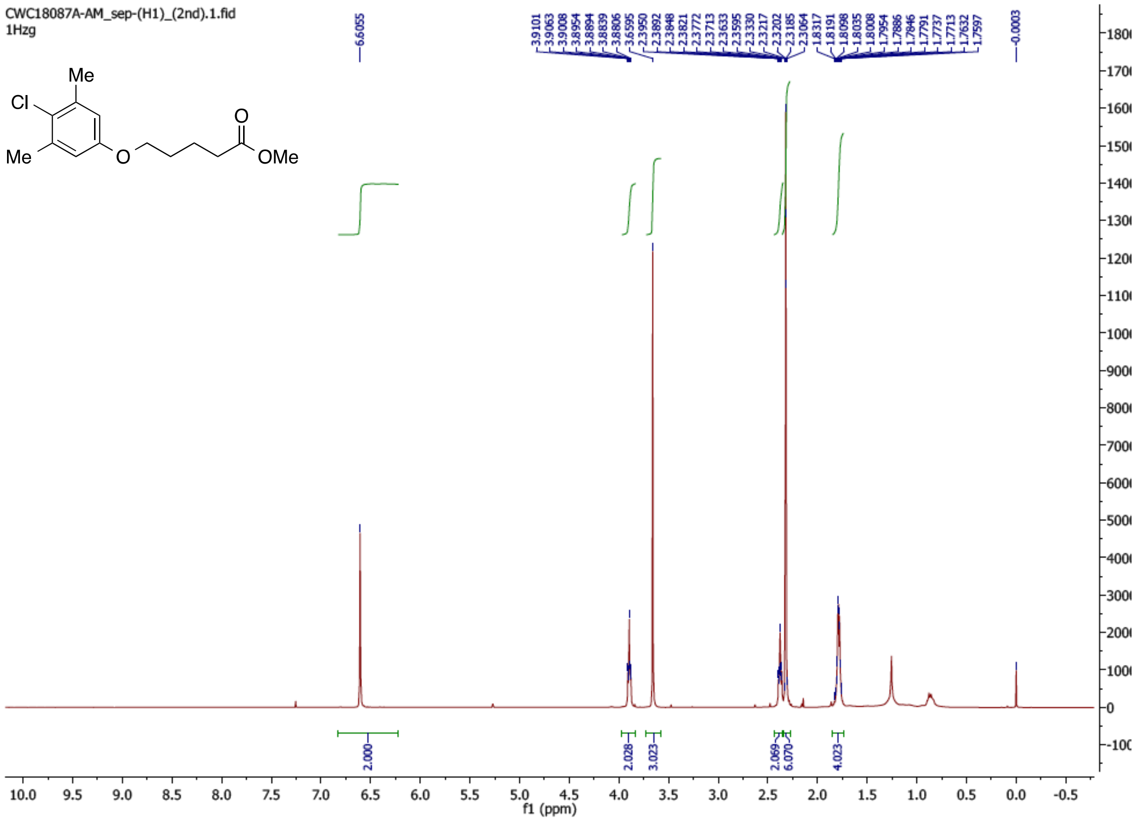

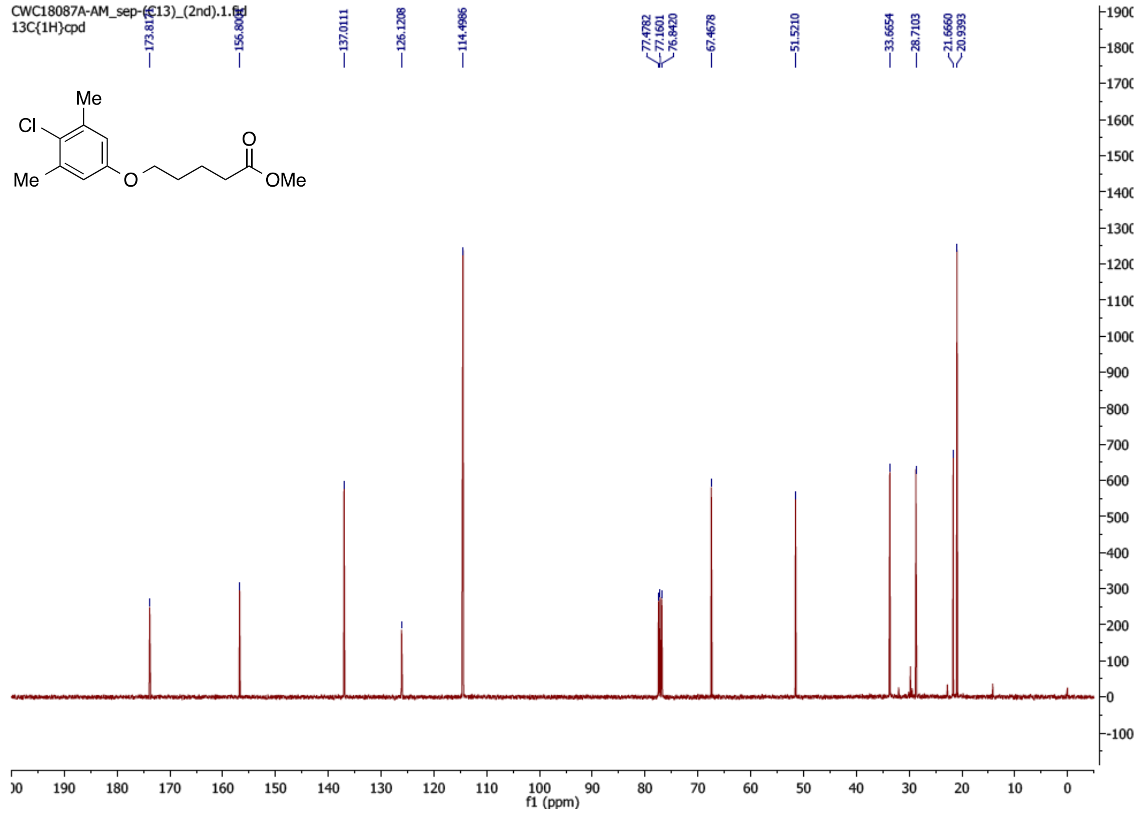

Supplementary Figure 4. ^{1}**H and** ^{13}**C NMR spectra of Methyl 5-(4-Chloro-3,5-dimethylphenoxy)pentanoate (S1)**

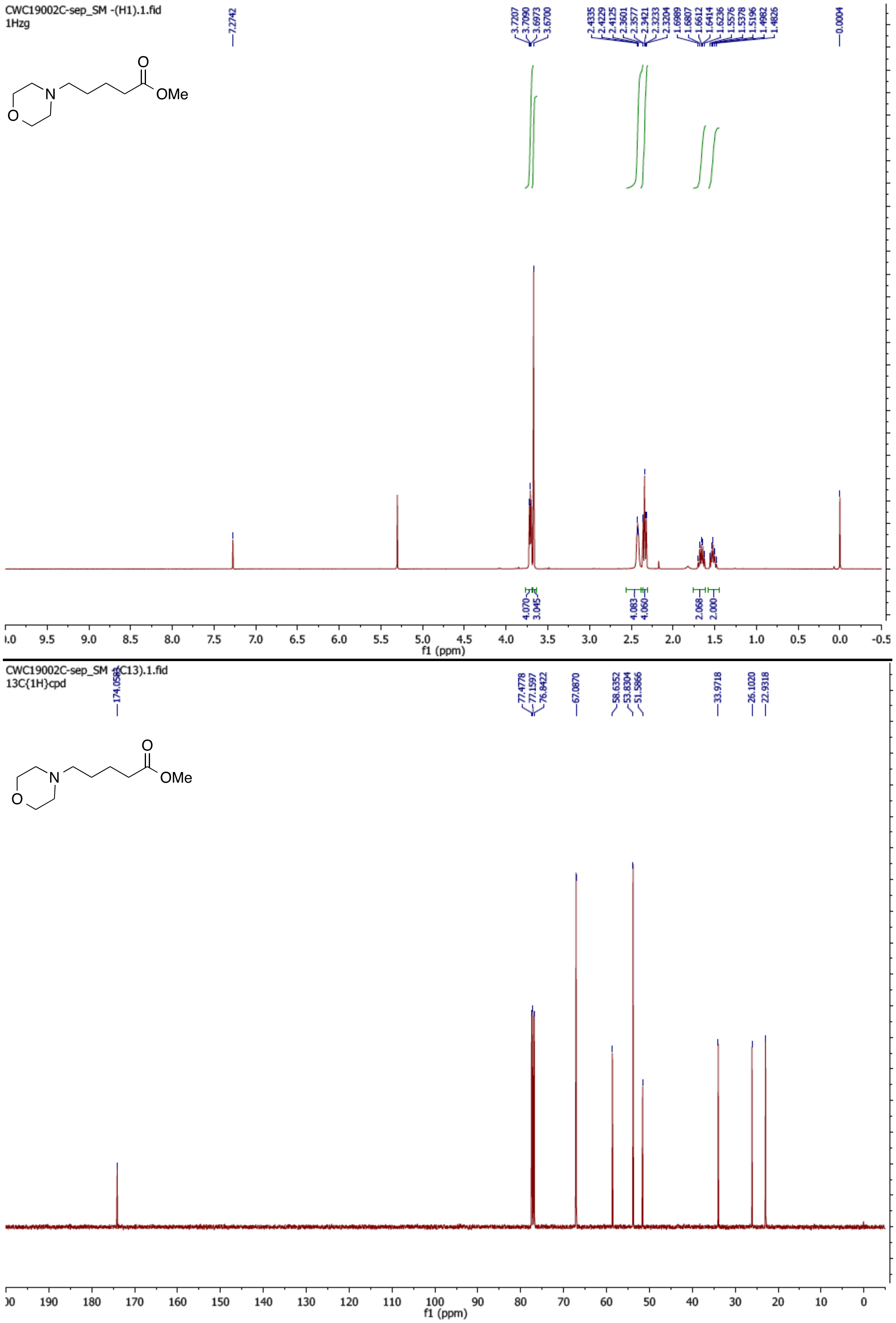

Supplementary Figure 5. ^{1}H and ^{13}C NMR spectra of Methyl 5-Morpholinopentanoate (S2)

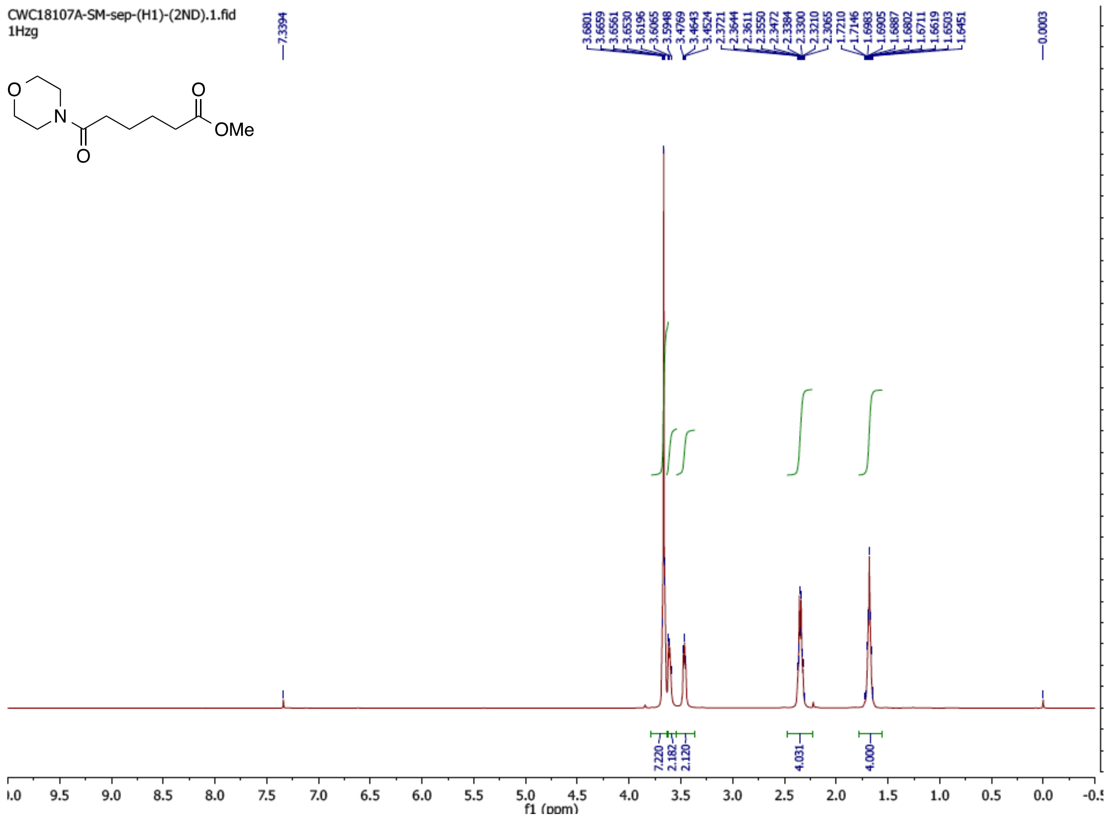

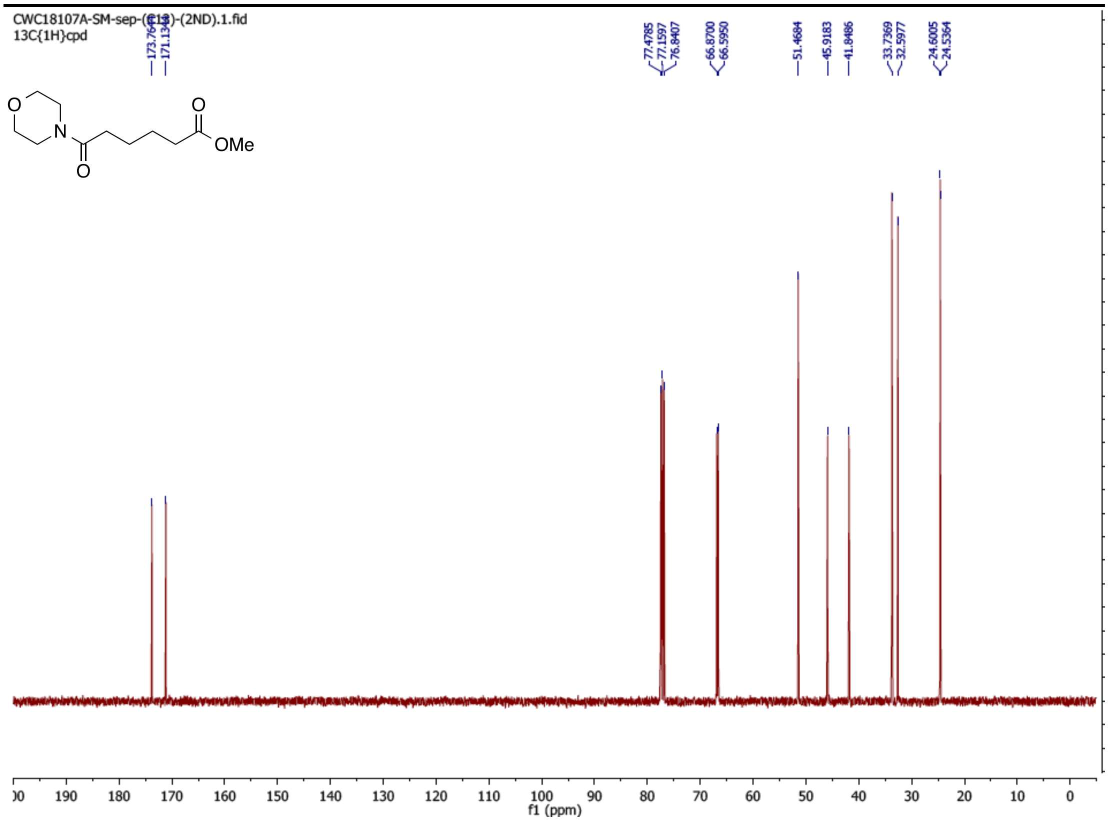

Supplementary Figure 6. ¹H and ¹³C NMR spectra of Methyl 6-Morpholino-6-oxohexanoate (S3)

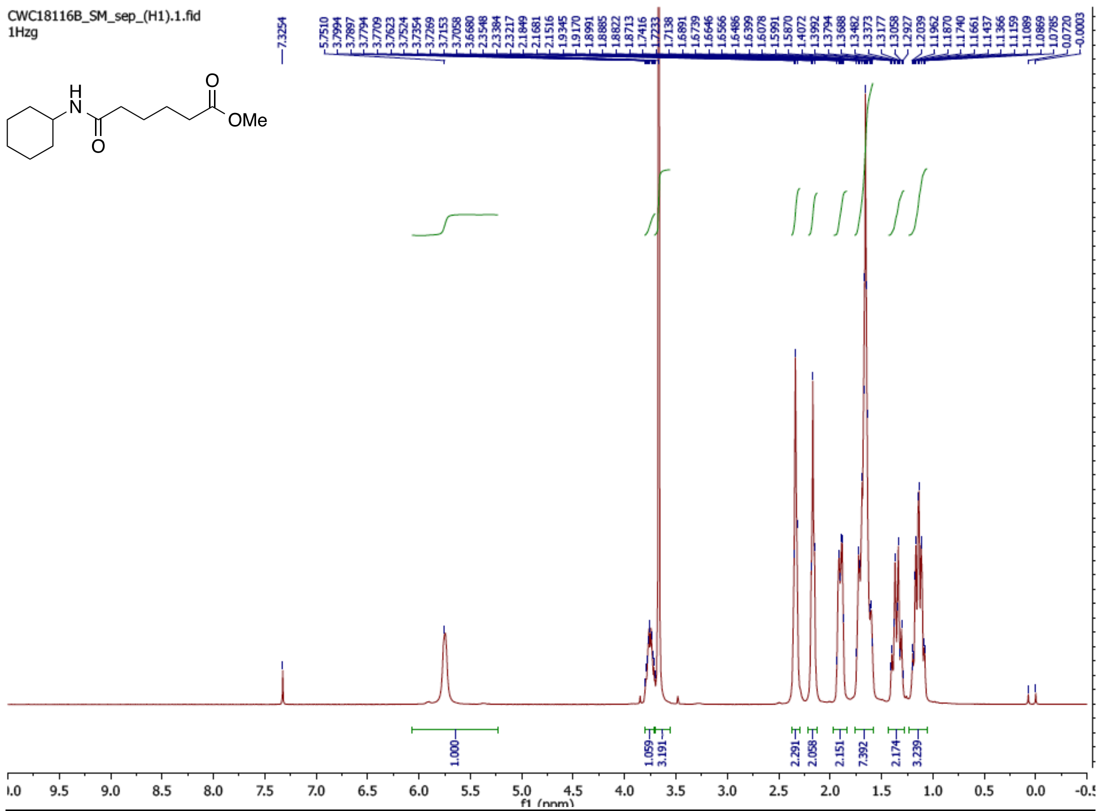

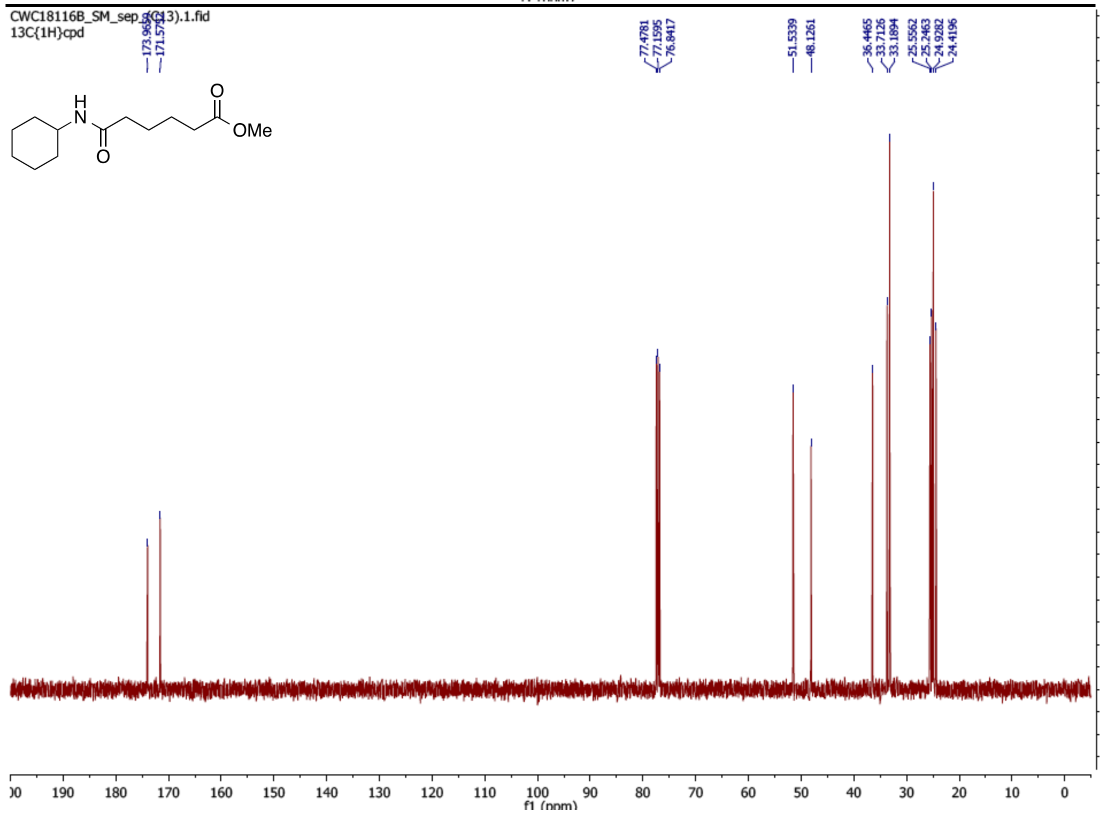

Supplementary Figure 7. ^{1}H and ^{13}C NMR spectra of Methyl 6-(Cyclohexylamino)-6-oxohexanoate (S4)

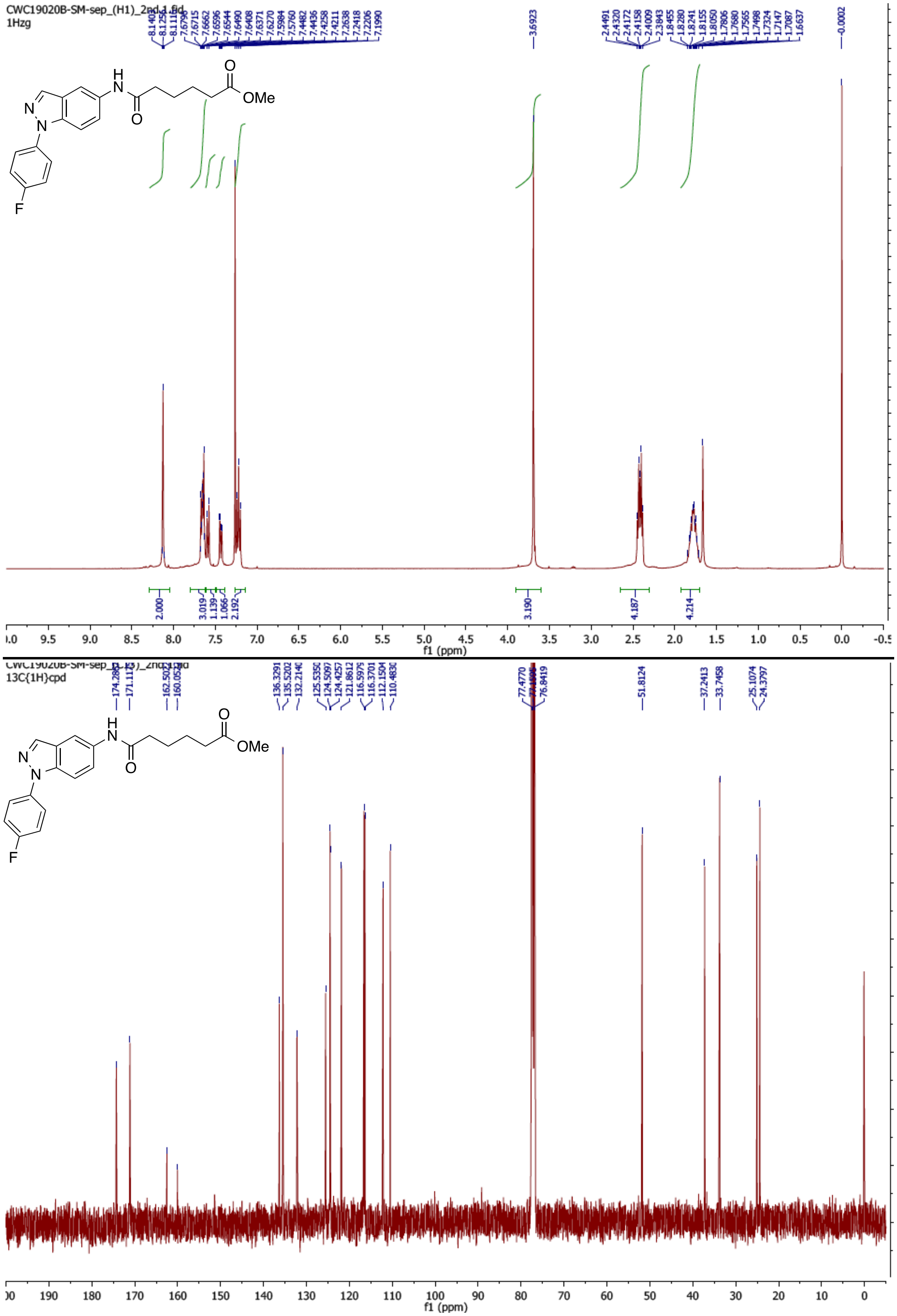

Supplementary Figure 8. ^{1}**H and** ^{13}**C NMR spectra of Methyl 6-((1-(4-Fluorophenyl)-1*H*-indazol-5-yl)amino)-6-oxohexanoate (S5)**

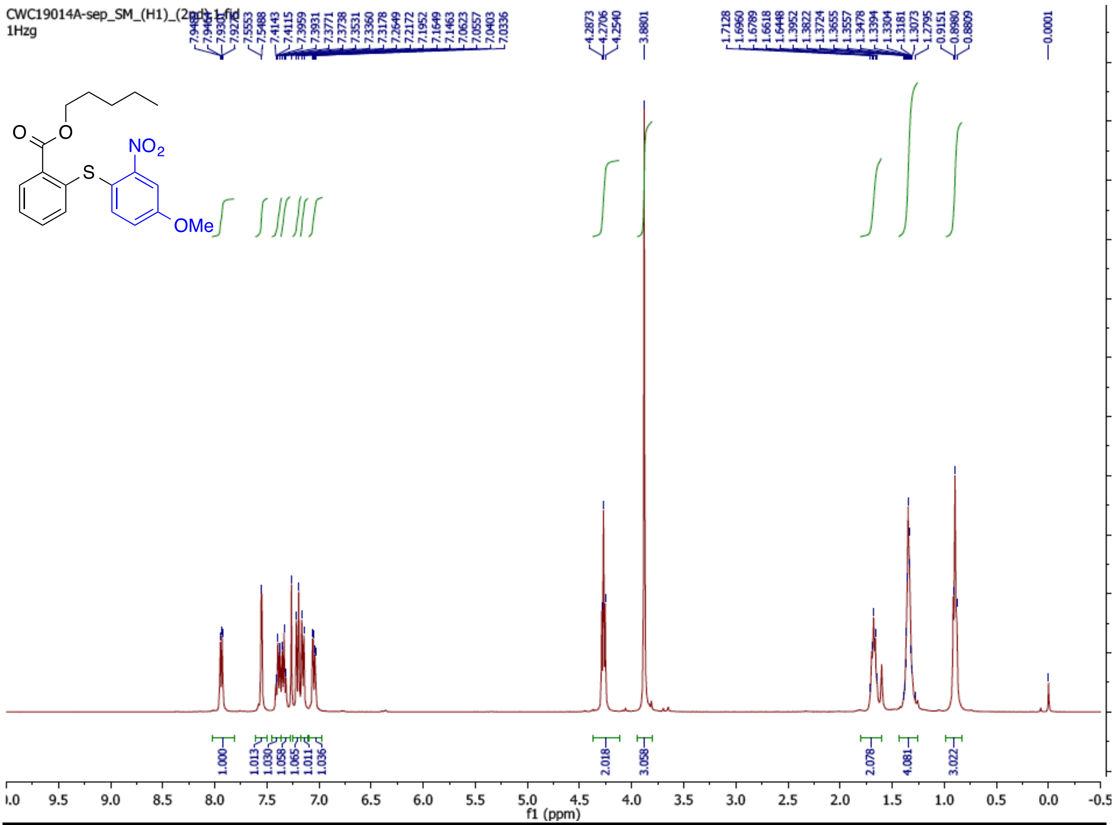

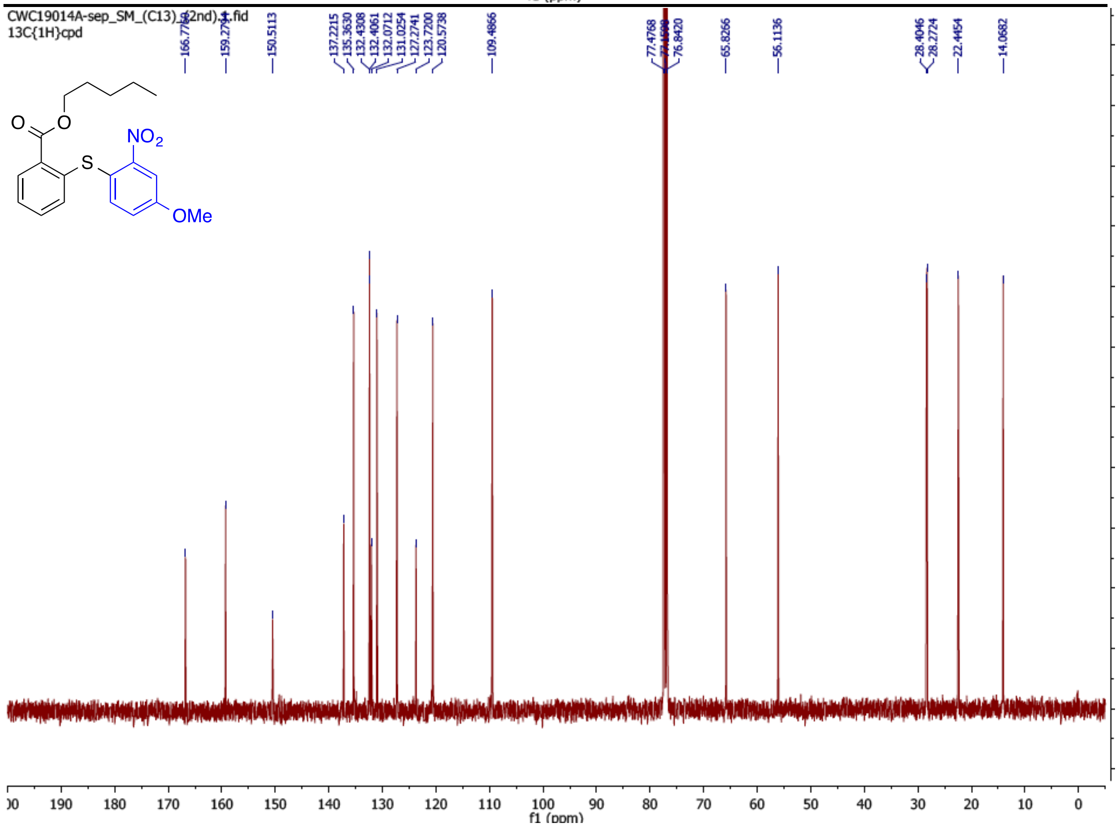

Supplementary Figure 9. ^{1}H and ^{13}C NMR spectra of Pentyl 2-((4-Methoxy-2-nitrophenyl)thio)benzoate (S6)

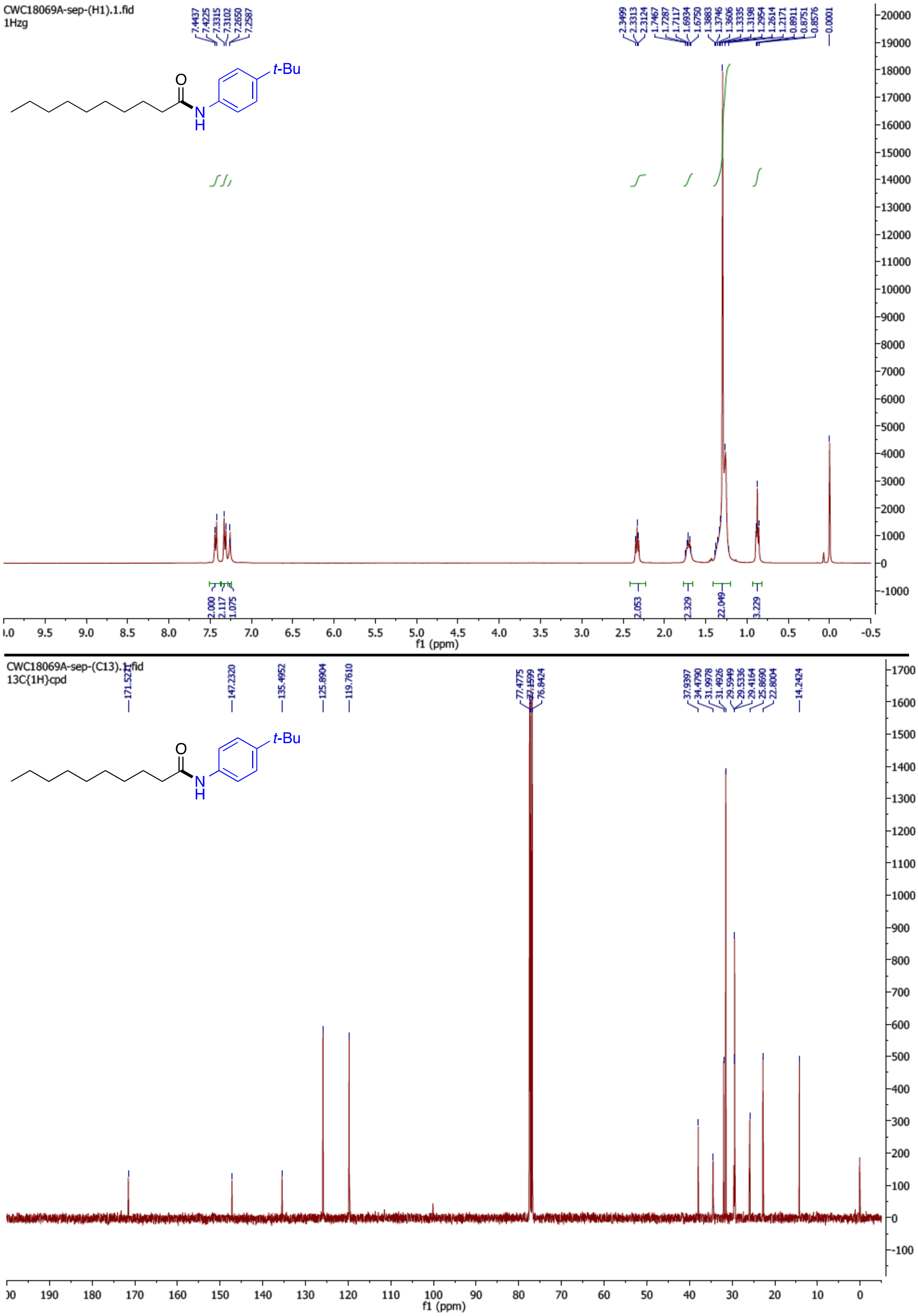

Supplementary Figure 10. ^{1}**H and** ^{13}**C NMR spectra of** ***N*****-(4-(*****tert*****-Butyl)phenyl)decanamide (3a)**

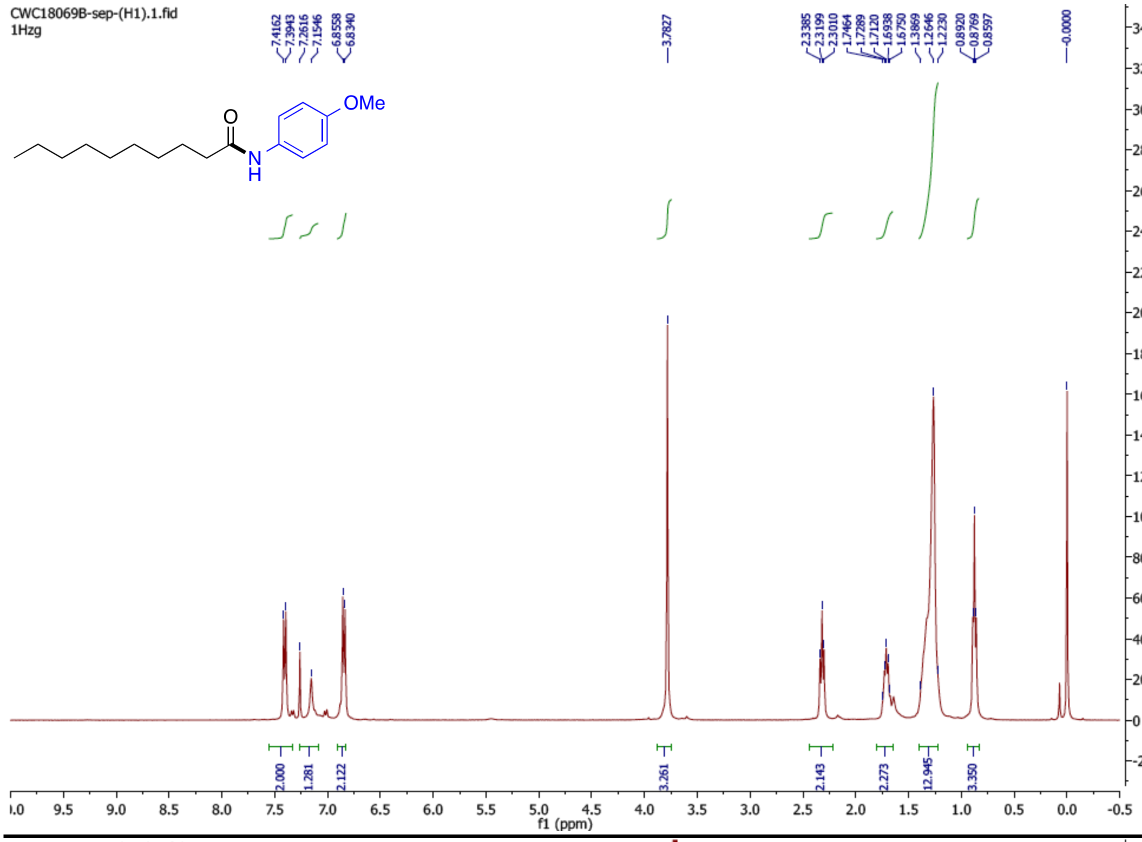

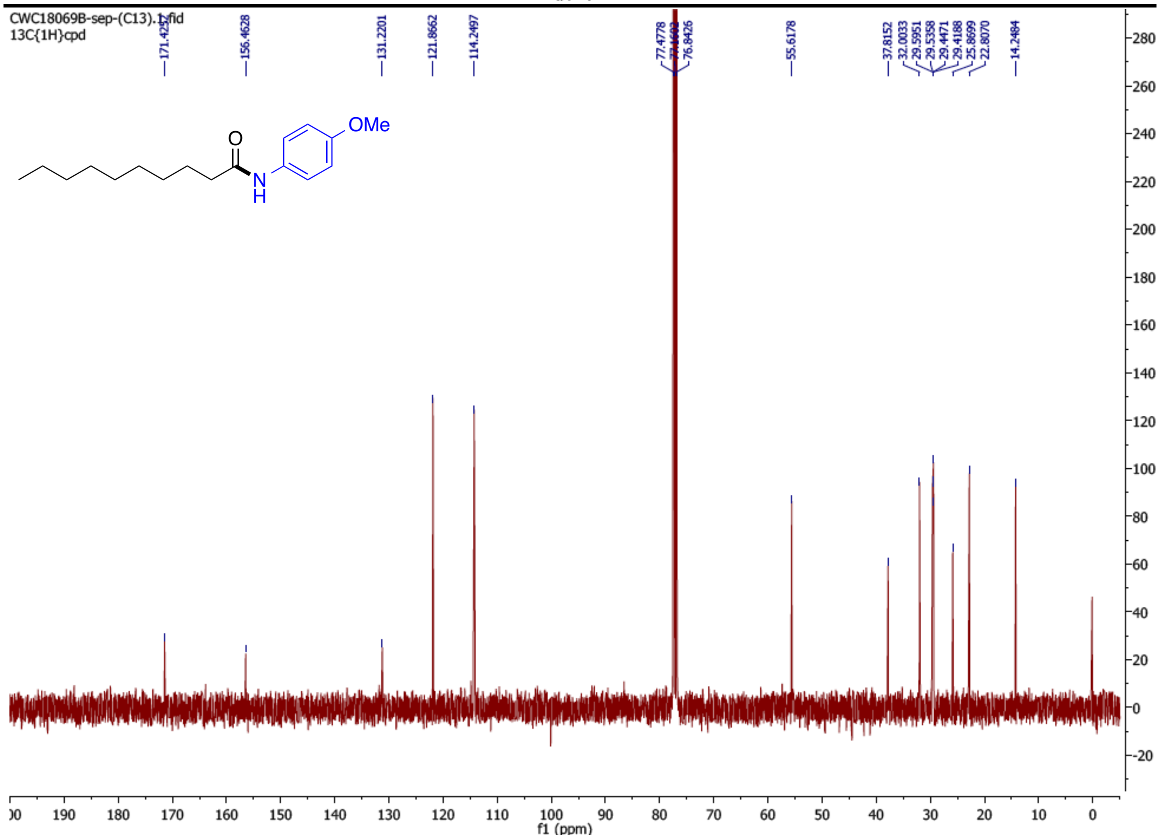

Supplementary Figure 11. **^{1}H and ^{13}C NMR spectra of *N*-(4-Methoxyphenyl)decanamide (3b)**

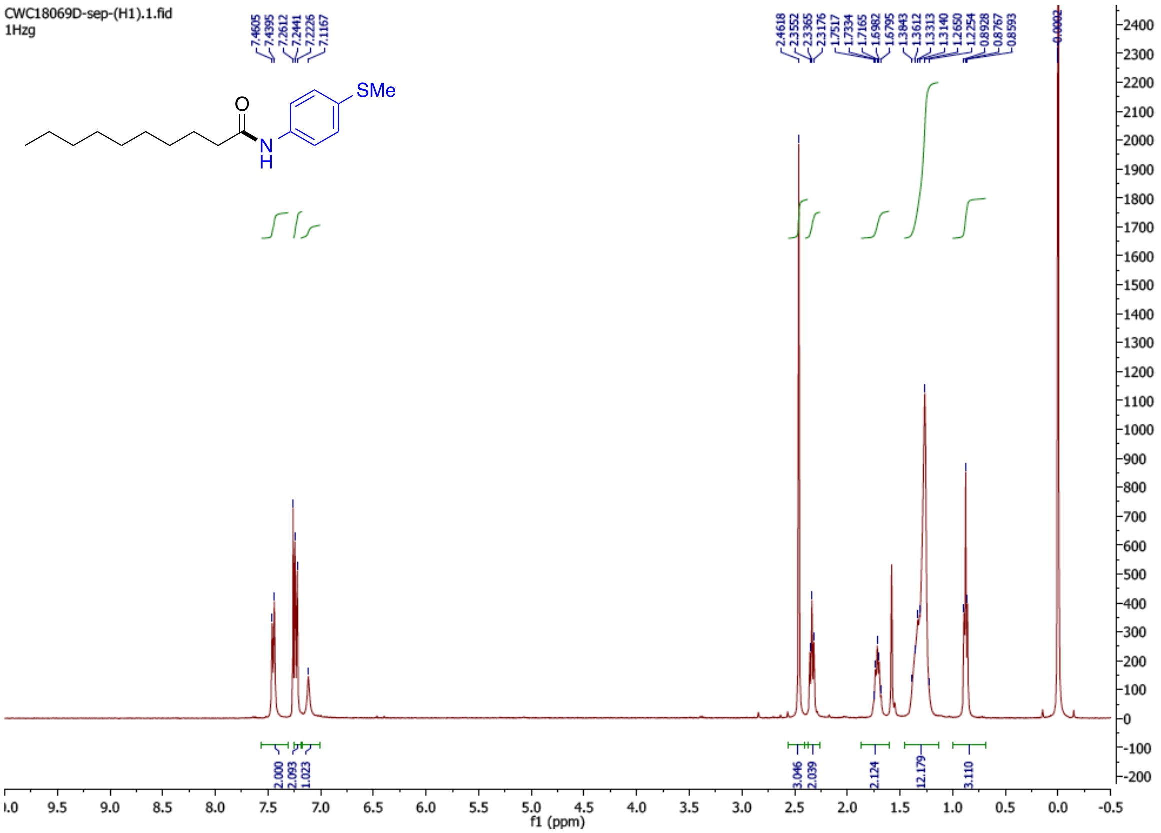

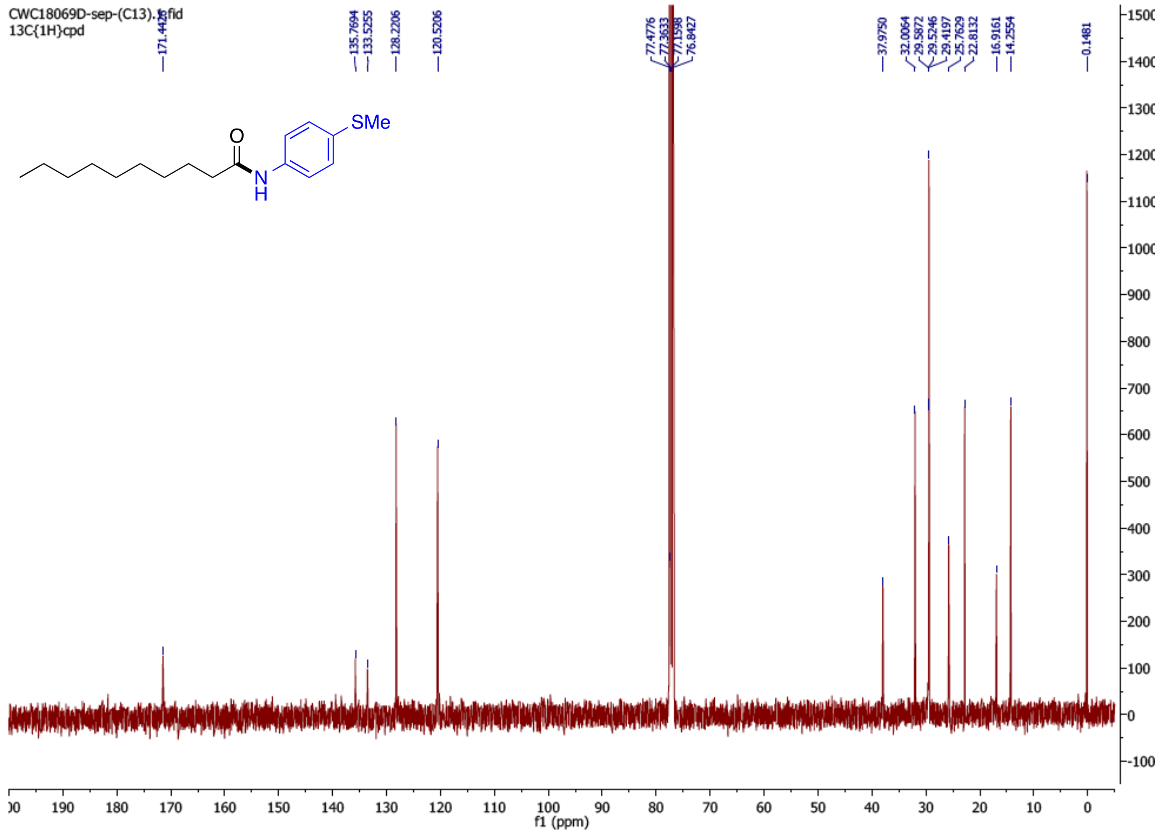

Supplementary Figure 12. ^{1}**H and** ^{13}**C NMR spectra of** ***N*****-(4-(Methylthio)phenyl)decanamide (3c)**

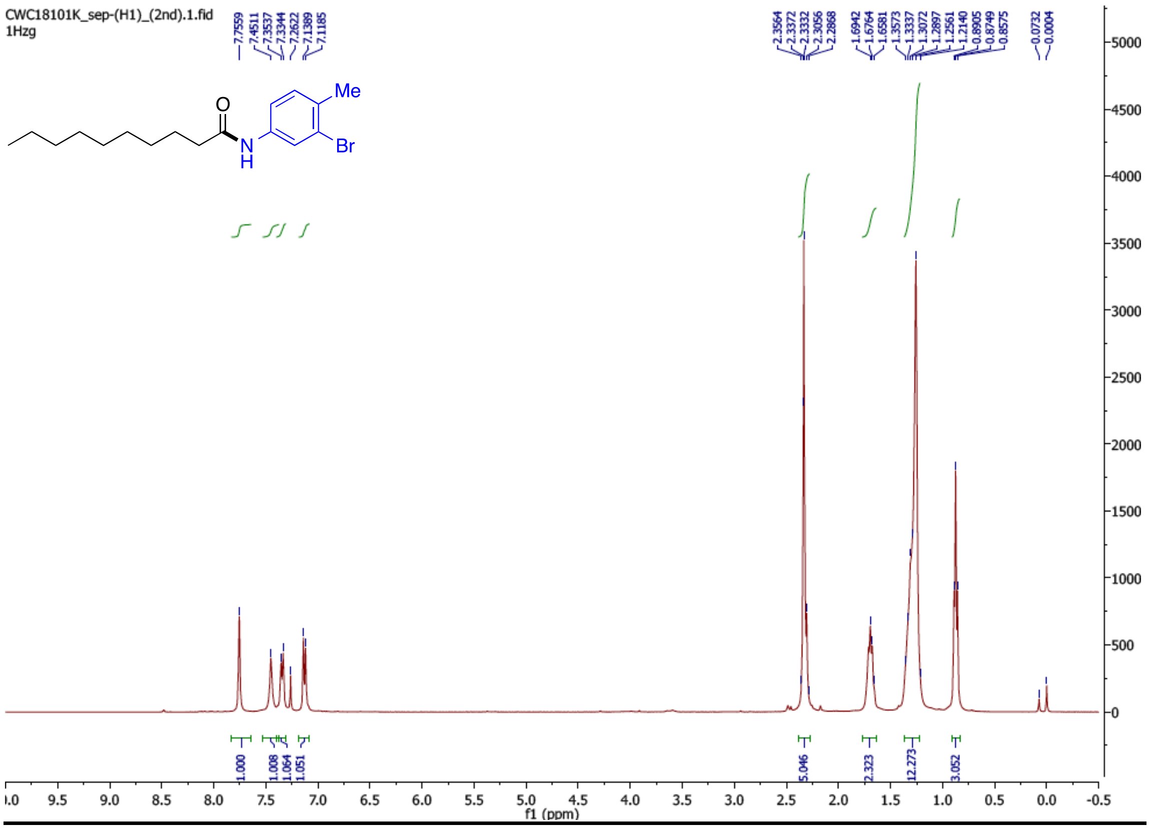

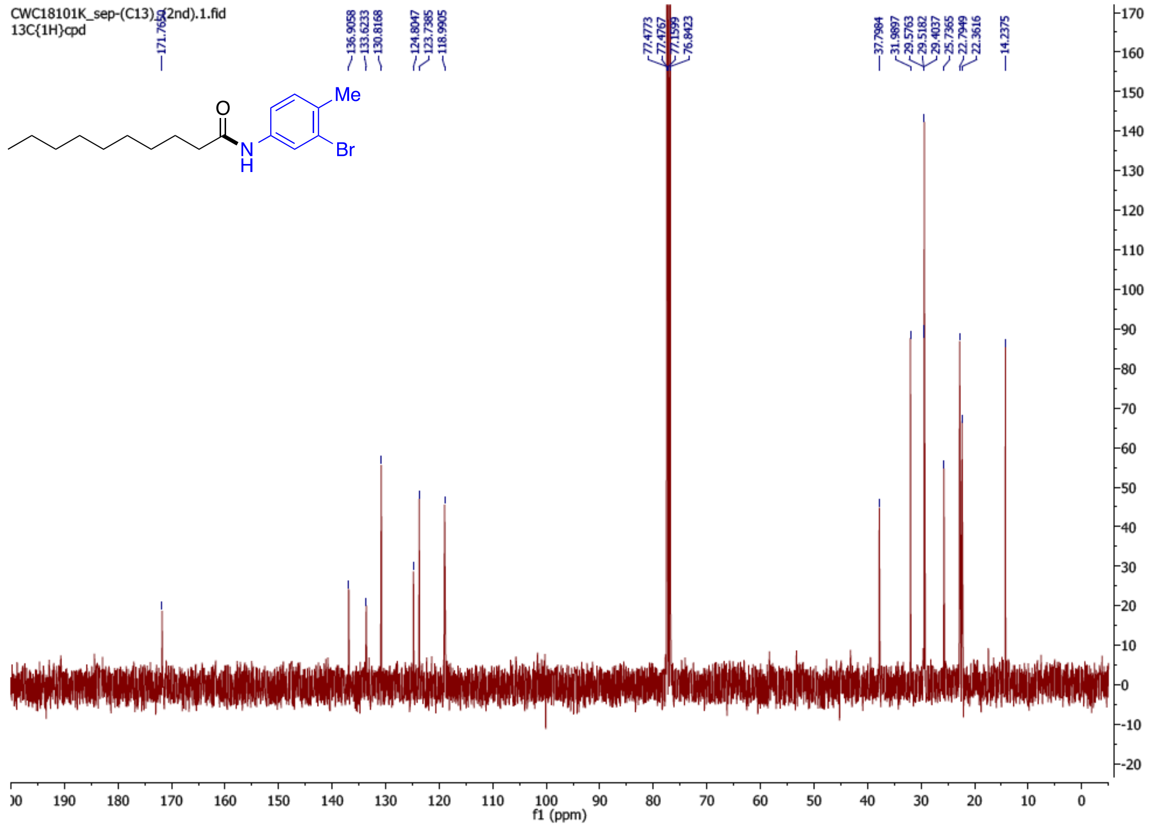

Supplementary Figure 13. ^{1}H and ^{13}C NMR spectra of *N*-(3-Bromo-4-methylphenyl)decanamide (3d)

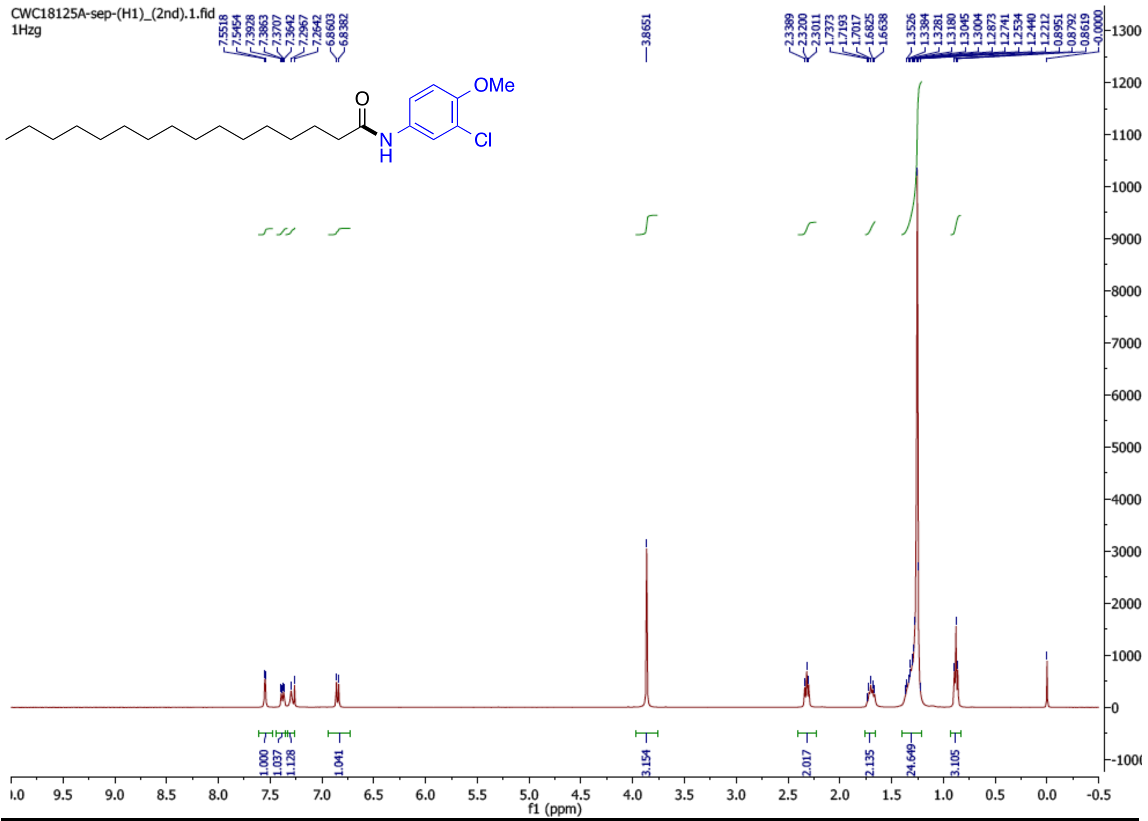

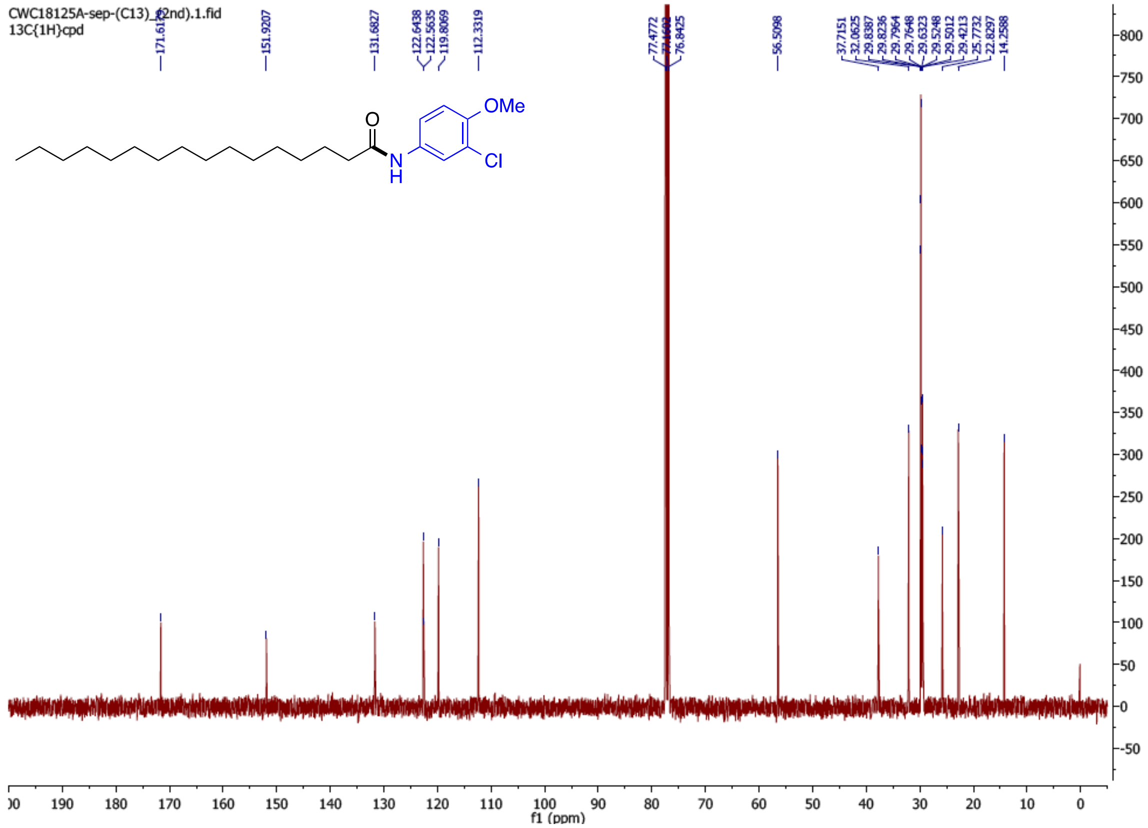

Supplementary Figure 14. ^{1}H and ^{13}C NMR spectra of *N*-(3-Chloro-4-methoxyphenyl)palmitamide (3e)

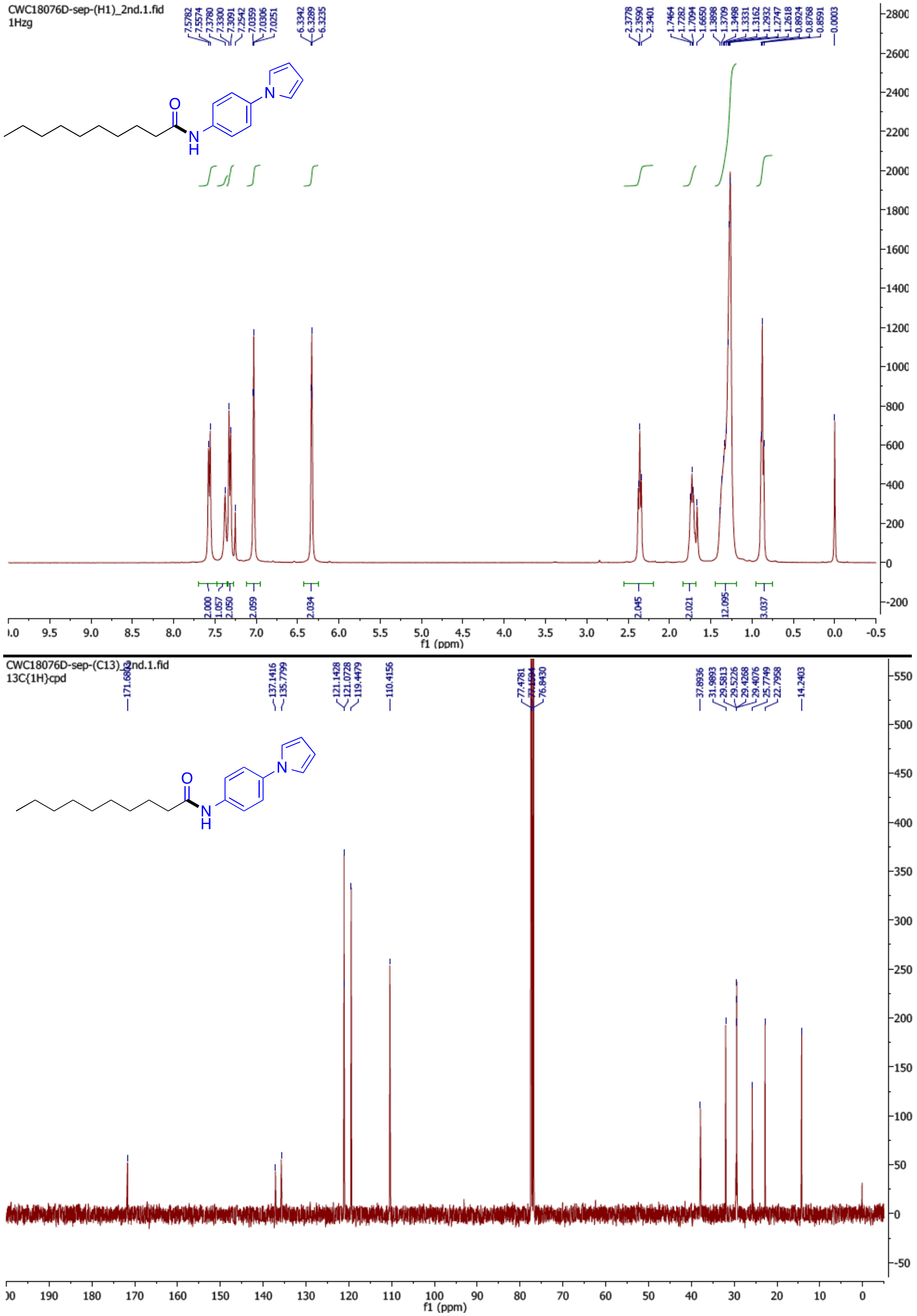

Supplementary Figure 15. **^{1}H and ^{13}C NMR spectra of *N*-(4-(1*H*-Pyrrol-1-yl)phenyl)decanamide (3f)**

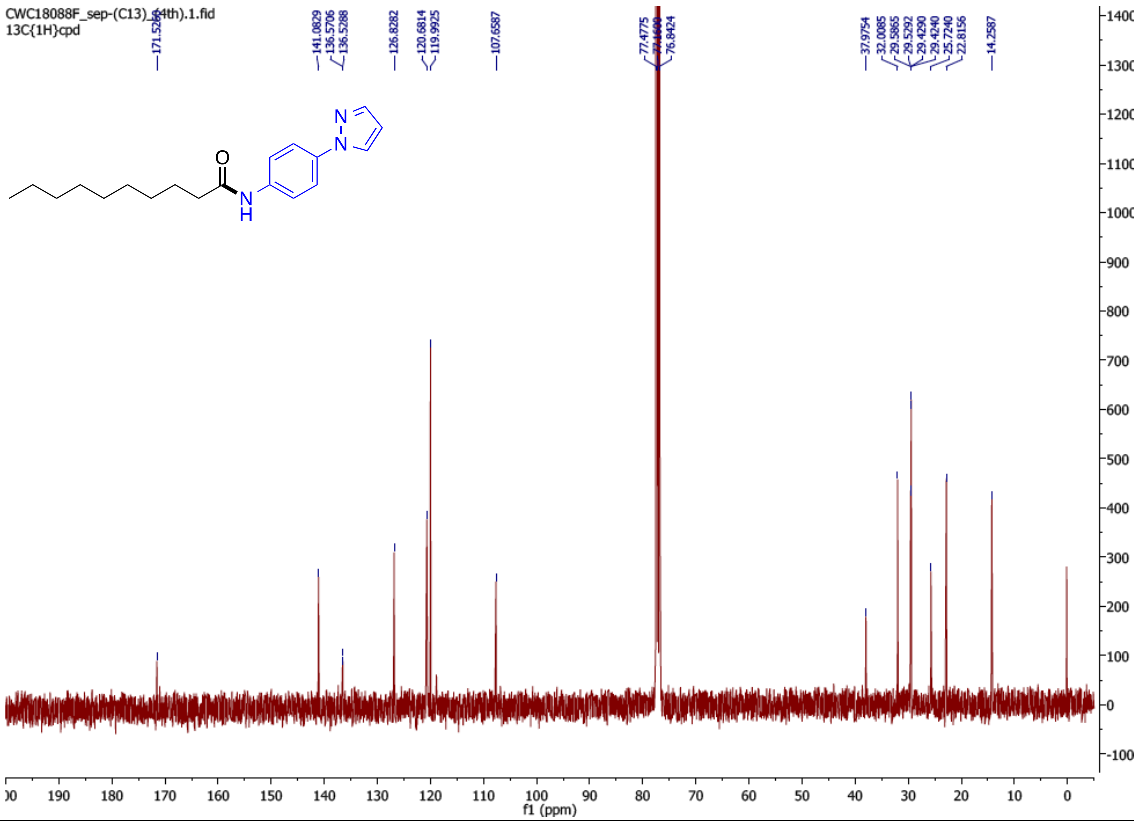

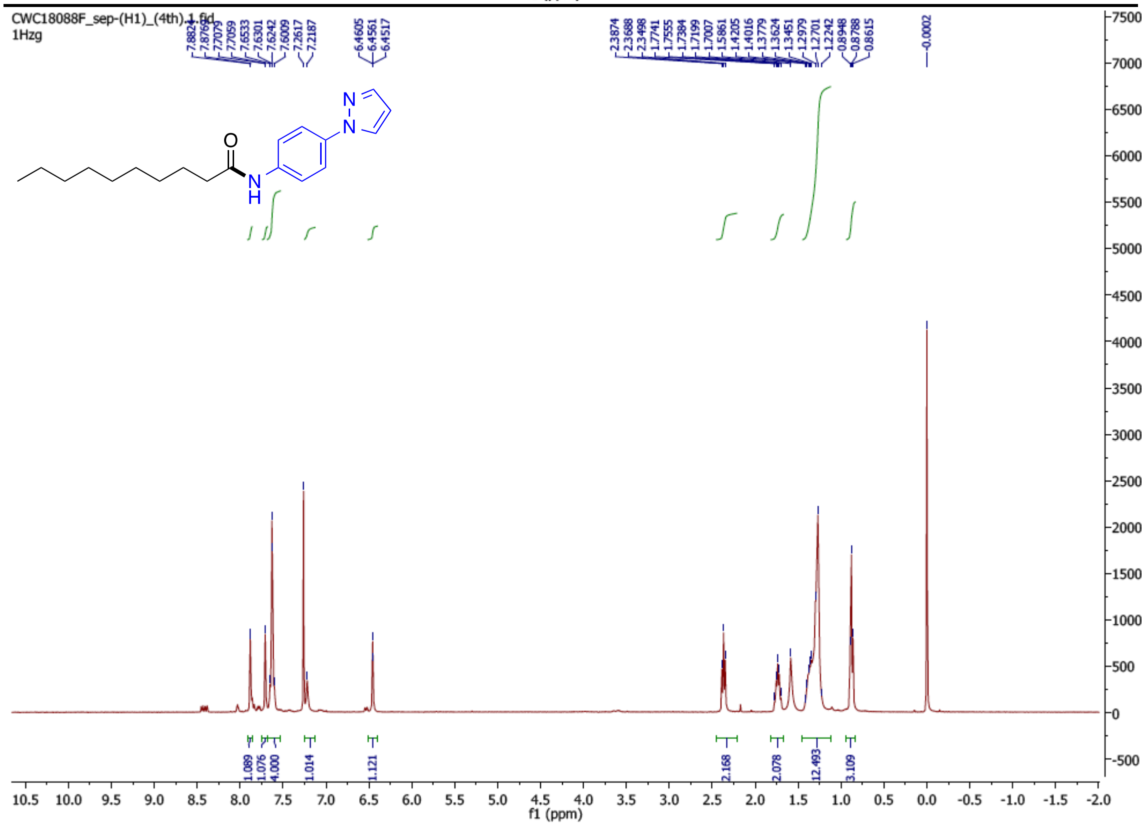

Supplementary Figure 16. ^{1}H and ^{13}C NMR spectra of *N*-(4-(1*H*-Pyrazol-1-yl)phenyl)decanamide (3g)

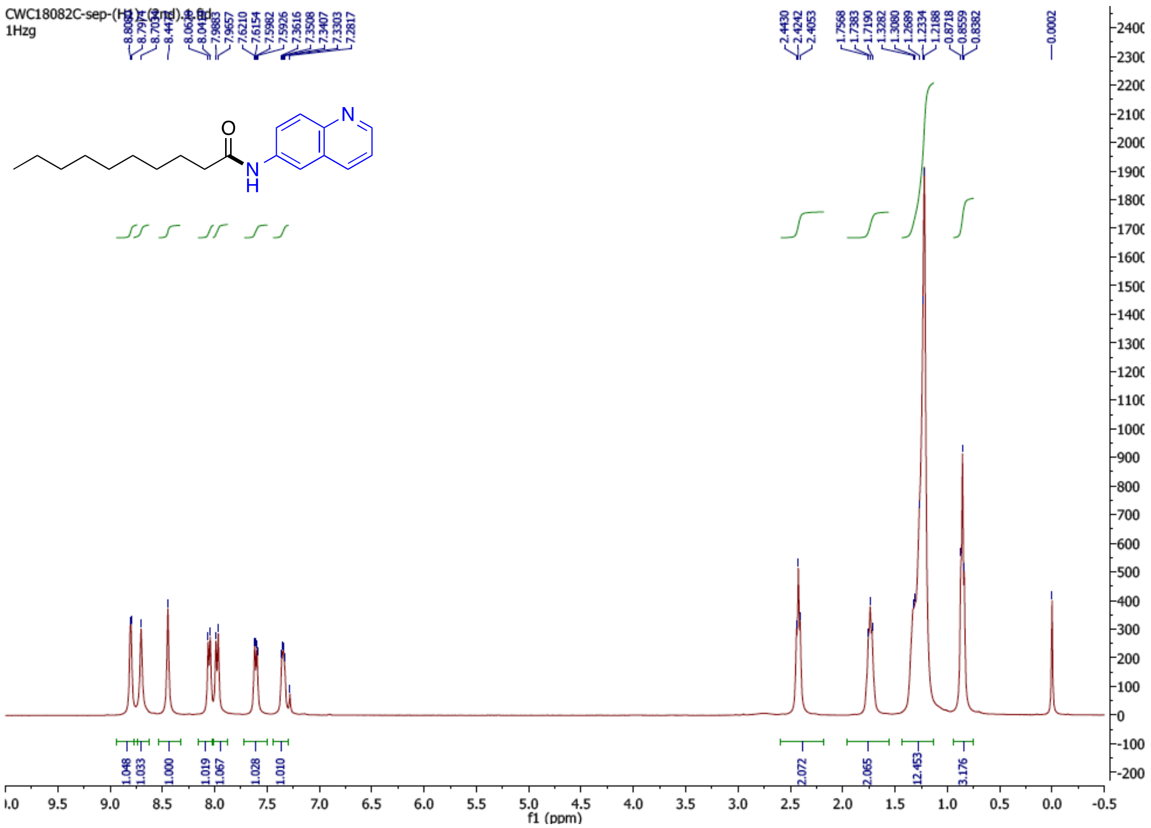

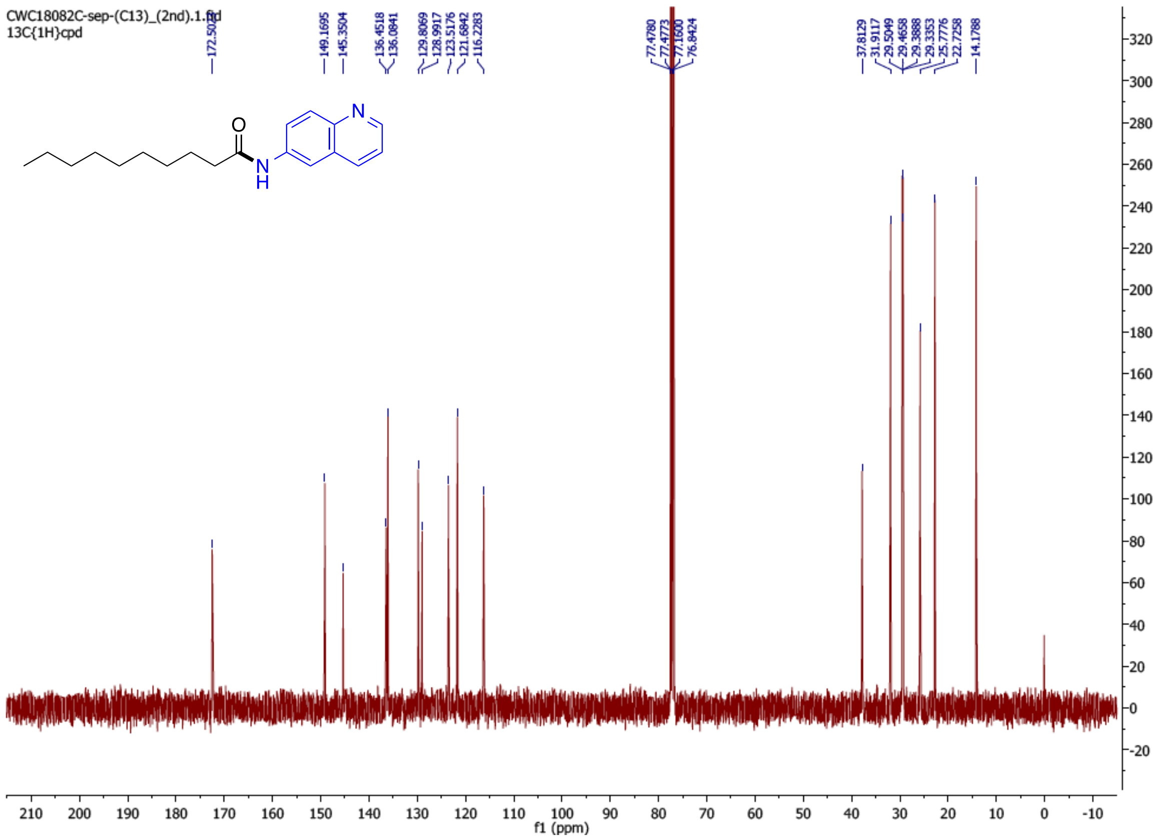

Supplementary Figure 17. ^{1}H and ^{13}C NMR spectra of *N*-(Quinolin-6-yl)decanamide (3h)

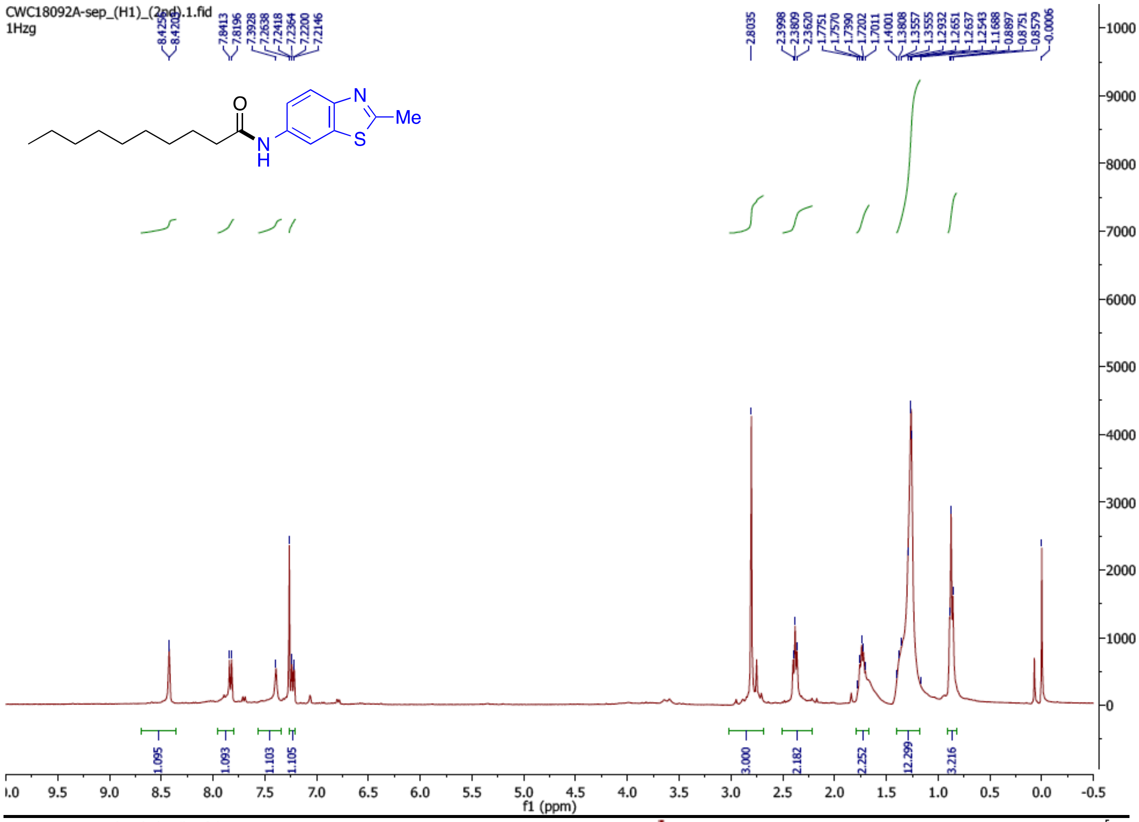

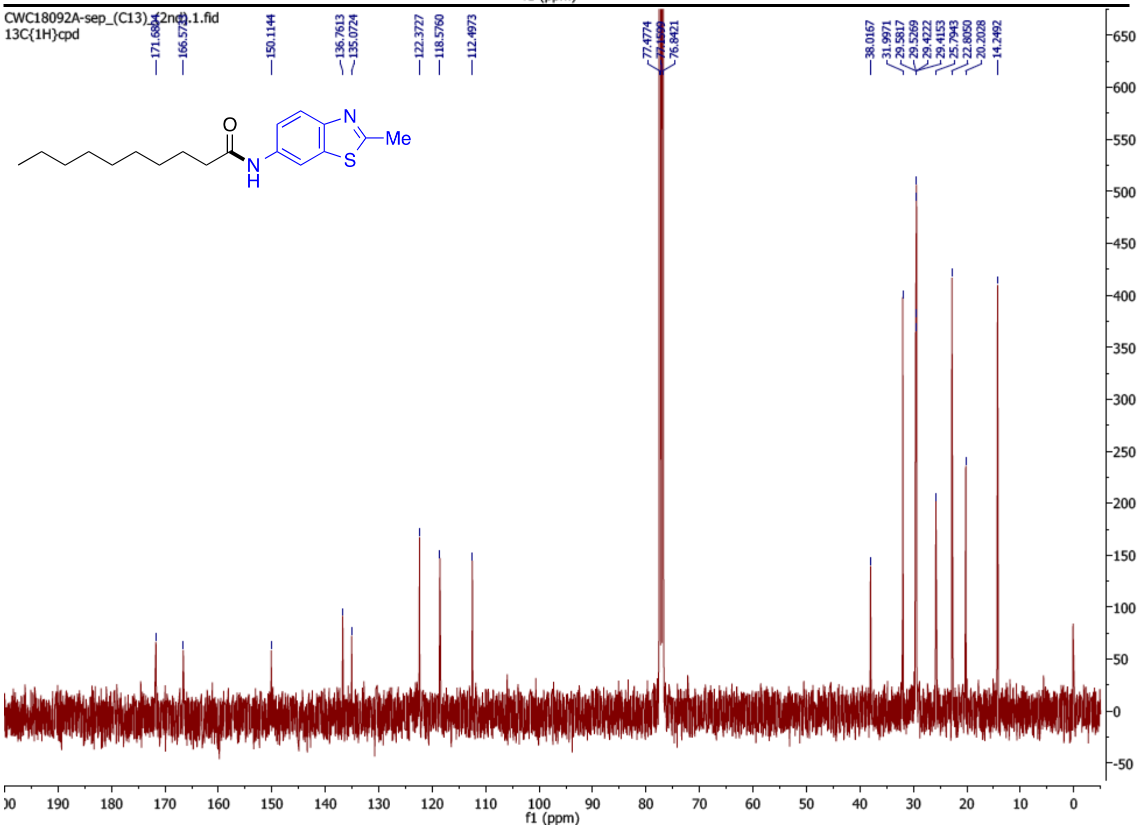

Supplementary Figure 18. ^{1}H and ^{13}C NMR spectra of *N*-(2-Methylbenzo[*d*]thiazol-6-yl)decanamide (3i)

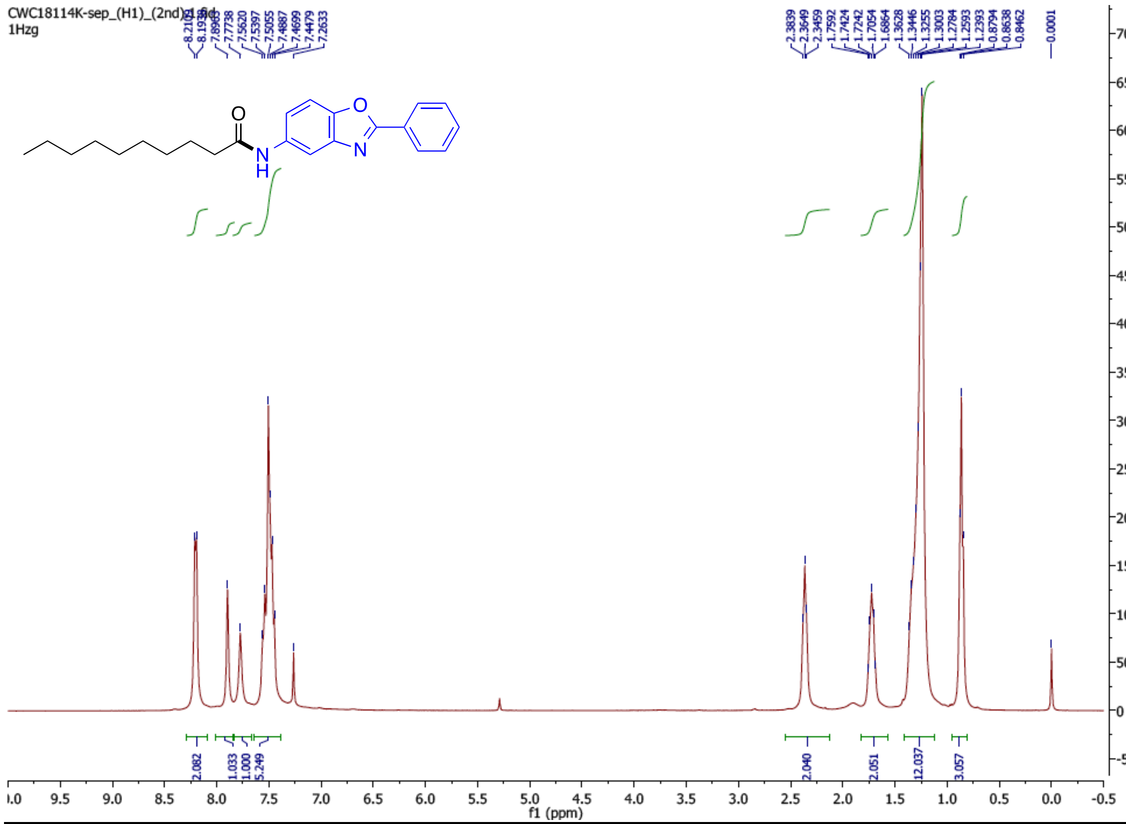

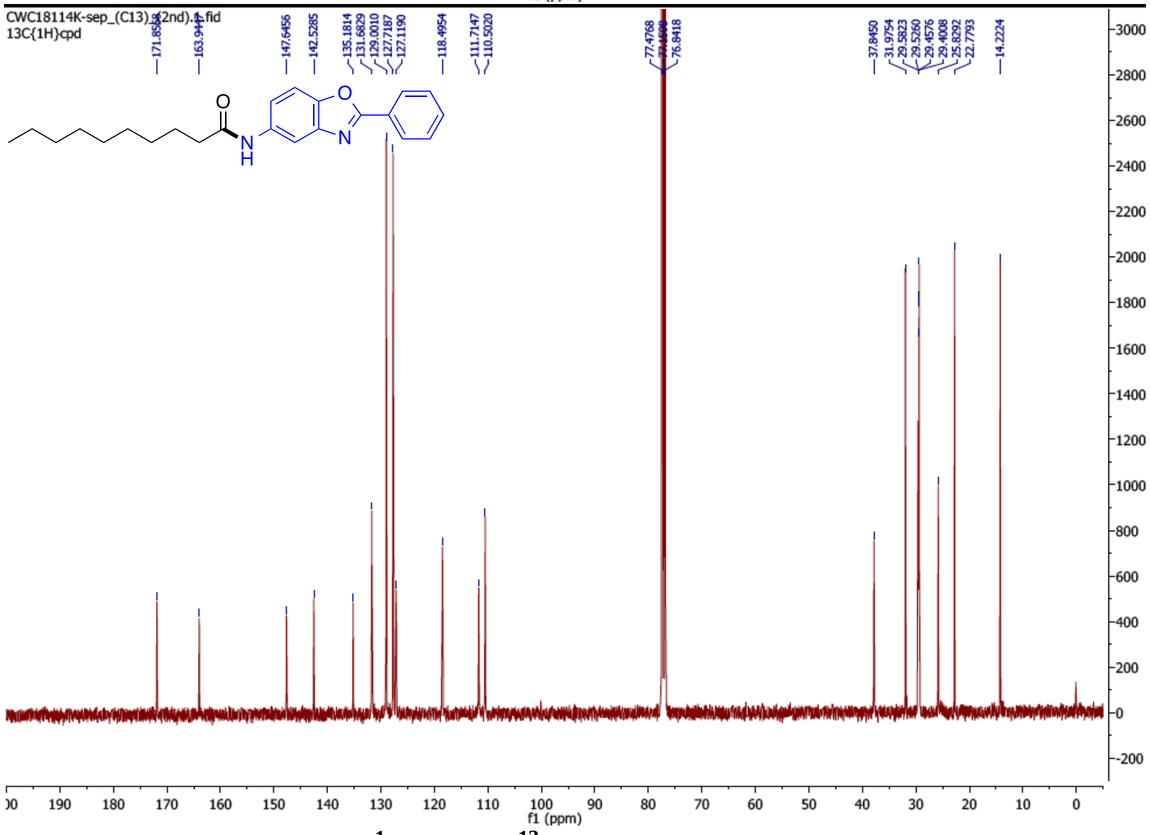

Supplementary Figure 19. ^{1}**H and** ^{13}**C NMR spectra of *N*-(2-Phenylbenzo[*d*]oxazol-5-yl)decanamide (3j)**

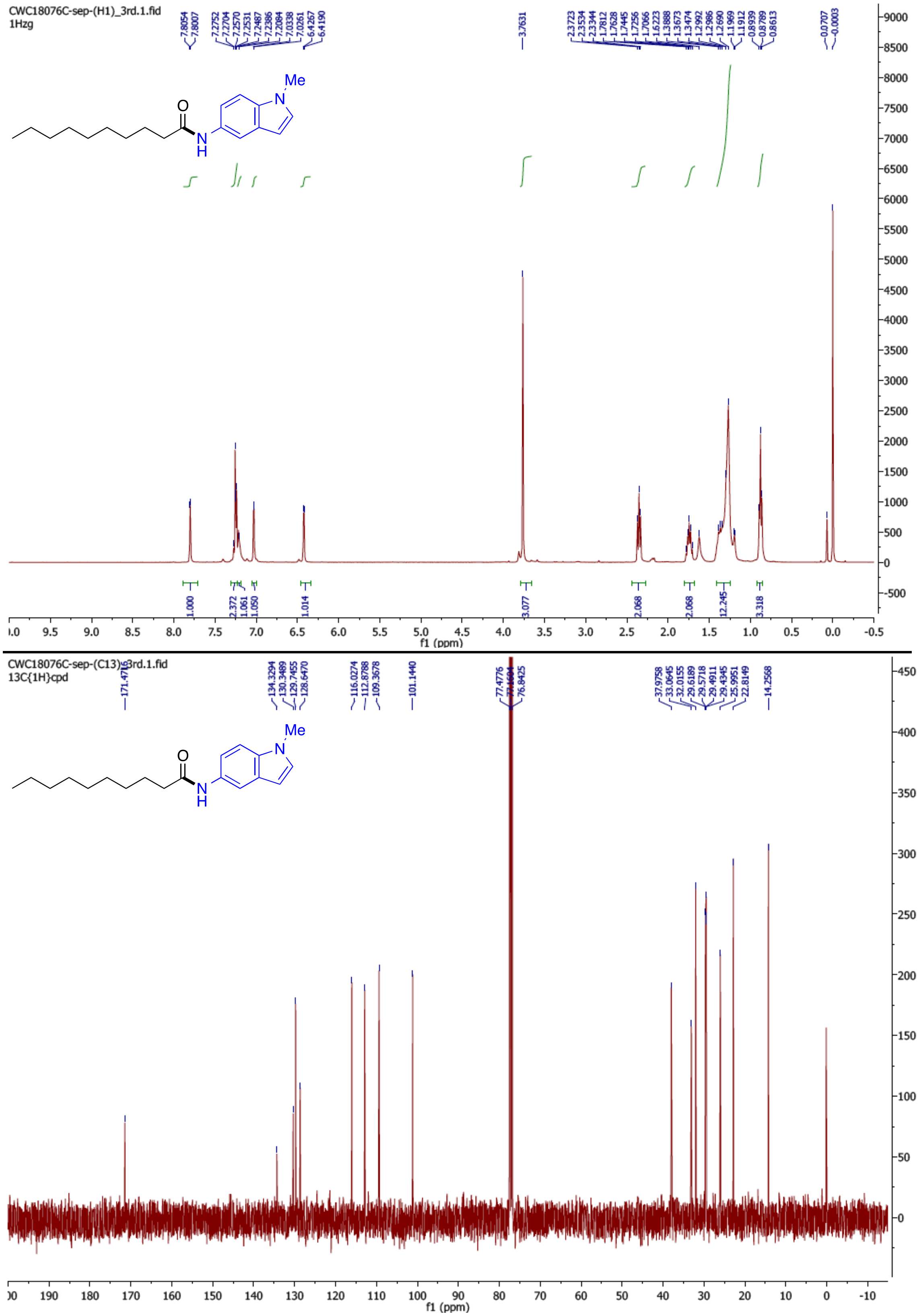

Supplementary Figure 20. ^{1}H and ^{13}C NMR spectra of *N*-(1-Methyl-1*H*-indol-5-yl)decanamide (3k)

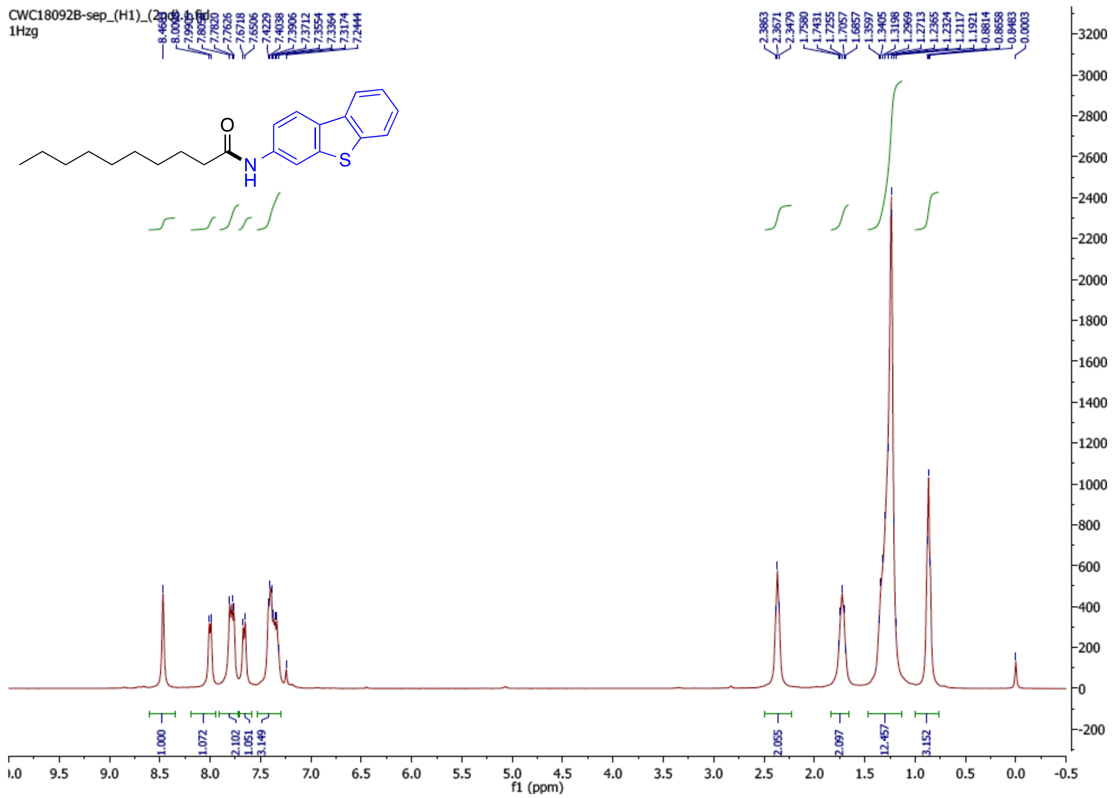

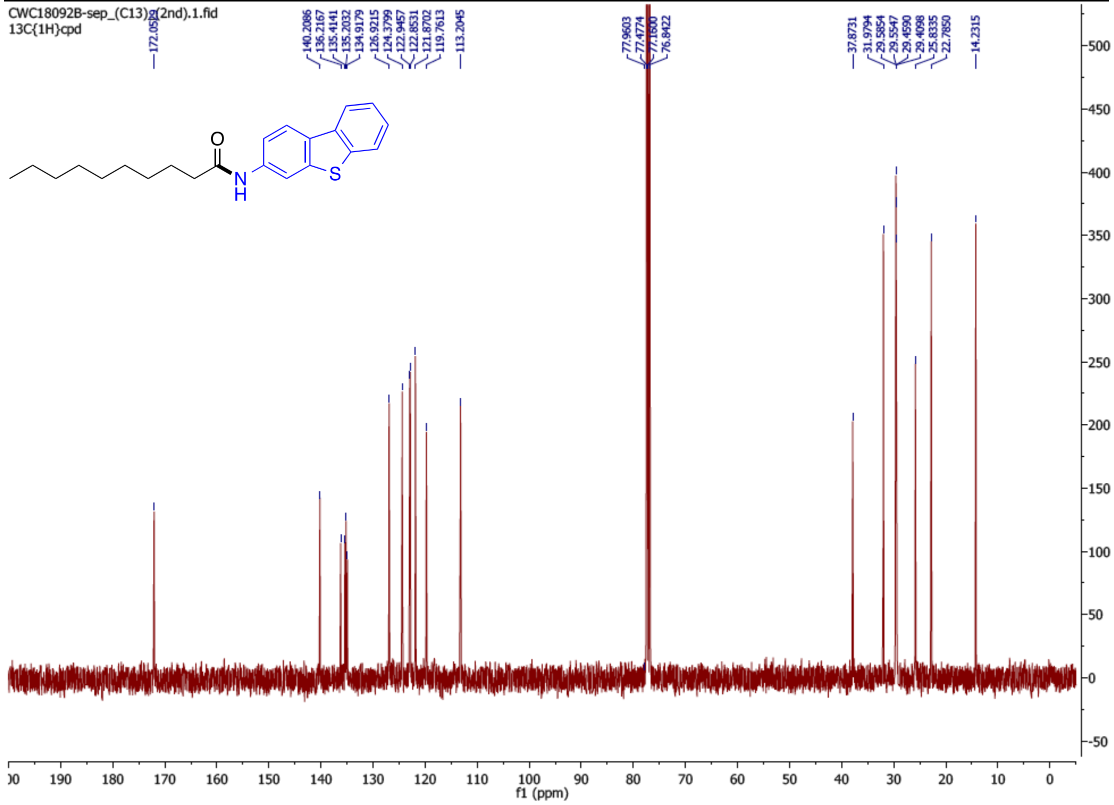

Supplementary Figure 21. ^{1}H and ^{13}C NMR spectra of *N*-(Dibenzo[*b*,*d*]thiophen-3-yl)decanamide (3l)

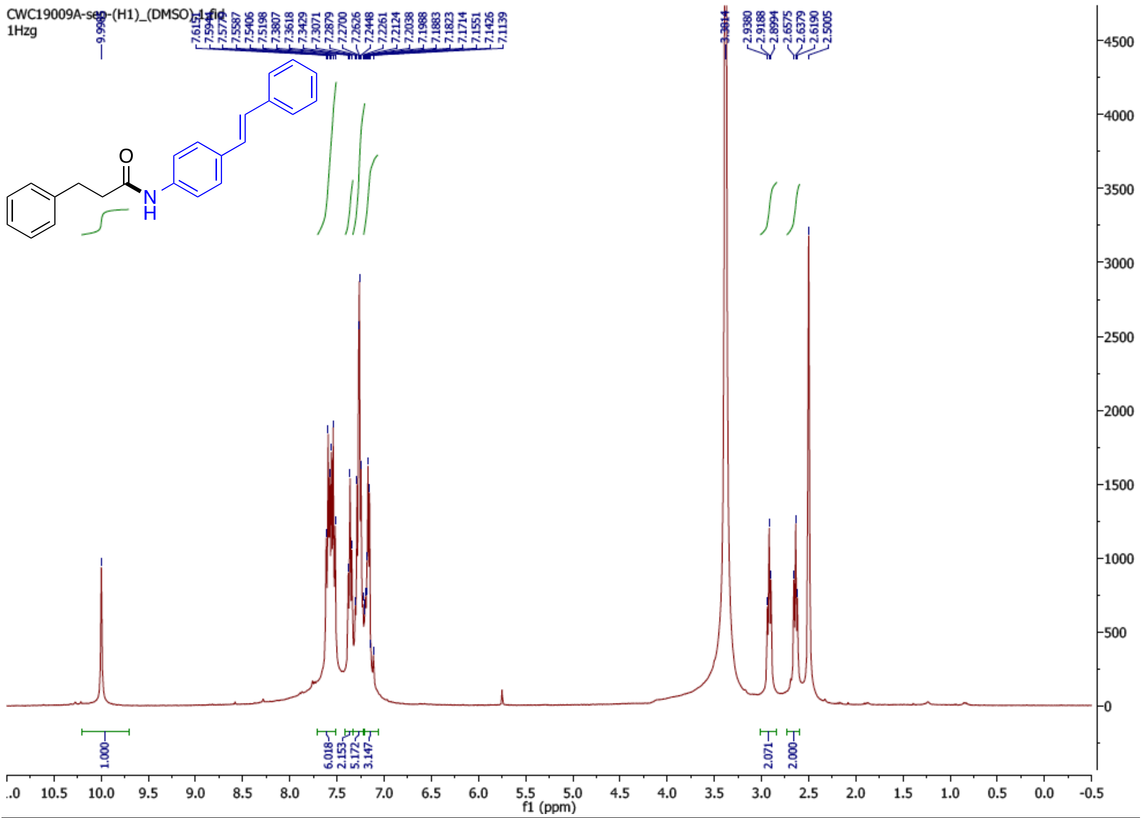

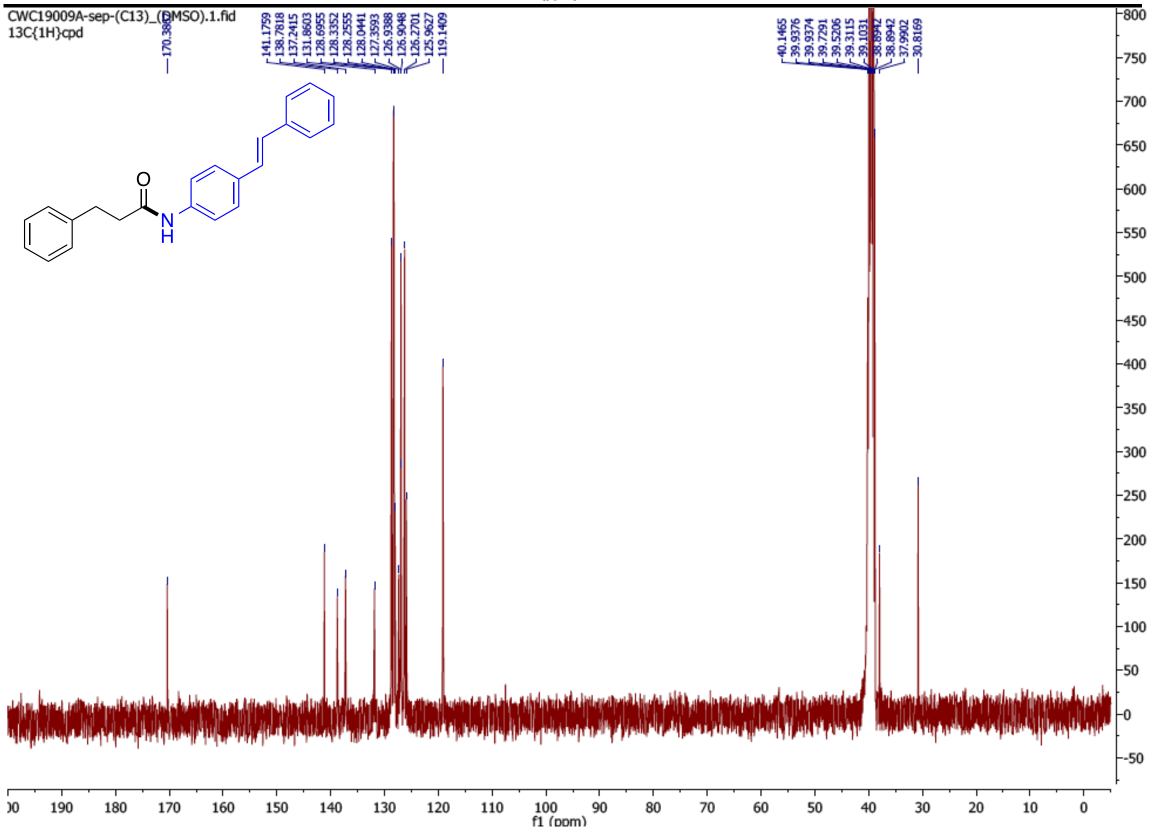

Supplementary Figure 22. ^{1}H and ^{13}C NMR spectra of (*E*)-3-Phenyl-*N*-(4-styrylphenyl)propanamide (3m)

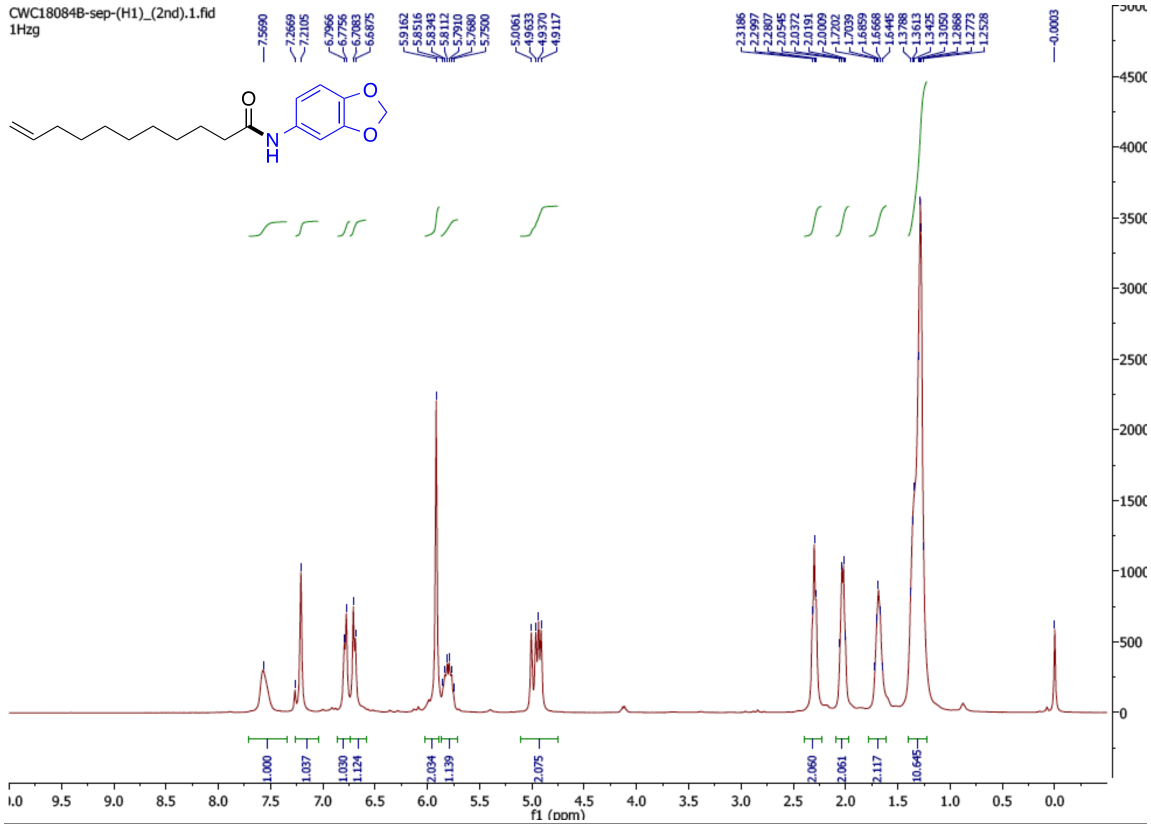

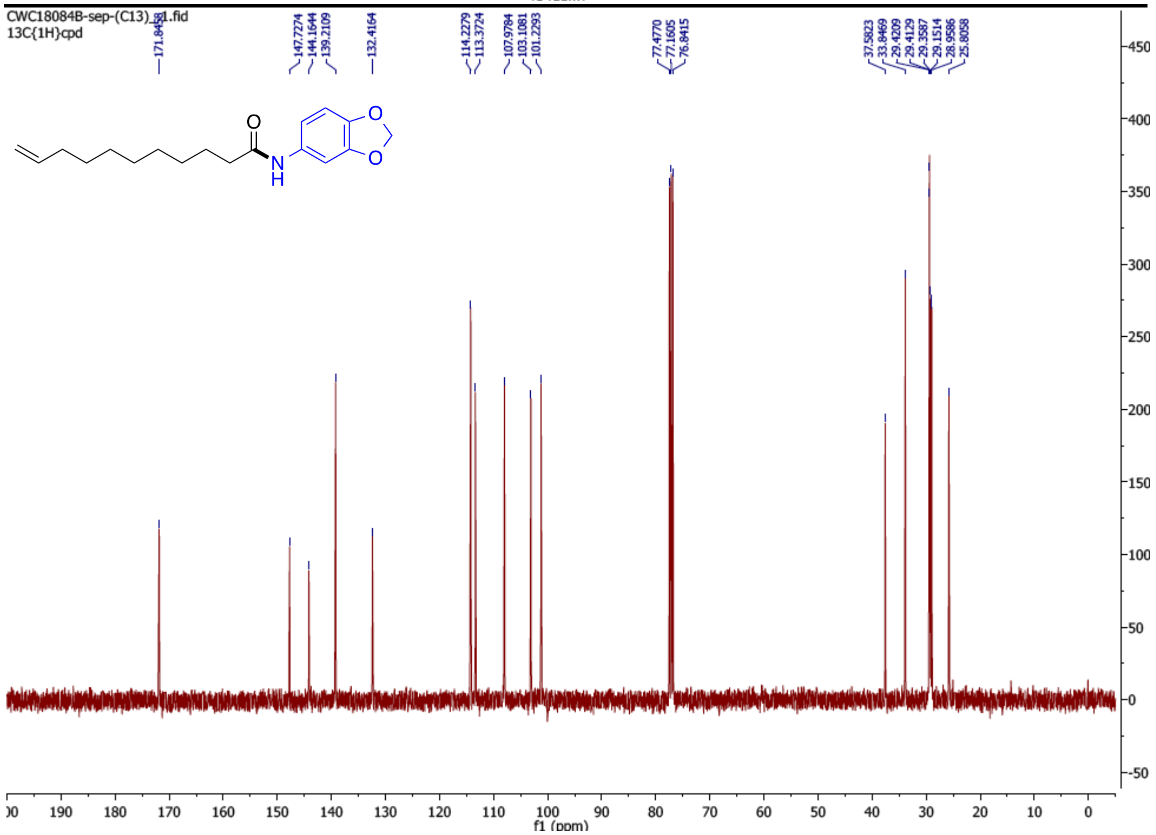

Supplementary Figure 23. ^{1}H and ^{13}C NMR spectra of *N*-(Benzo[*d*][1,3]dioxol-5-yl)undec-10-enamide (3n)

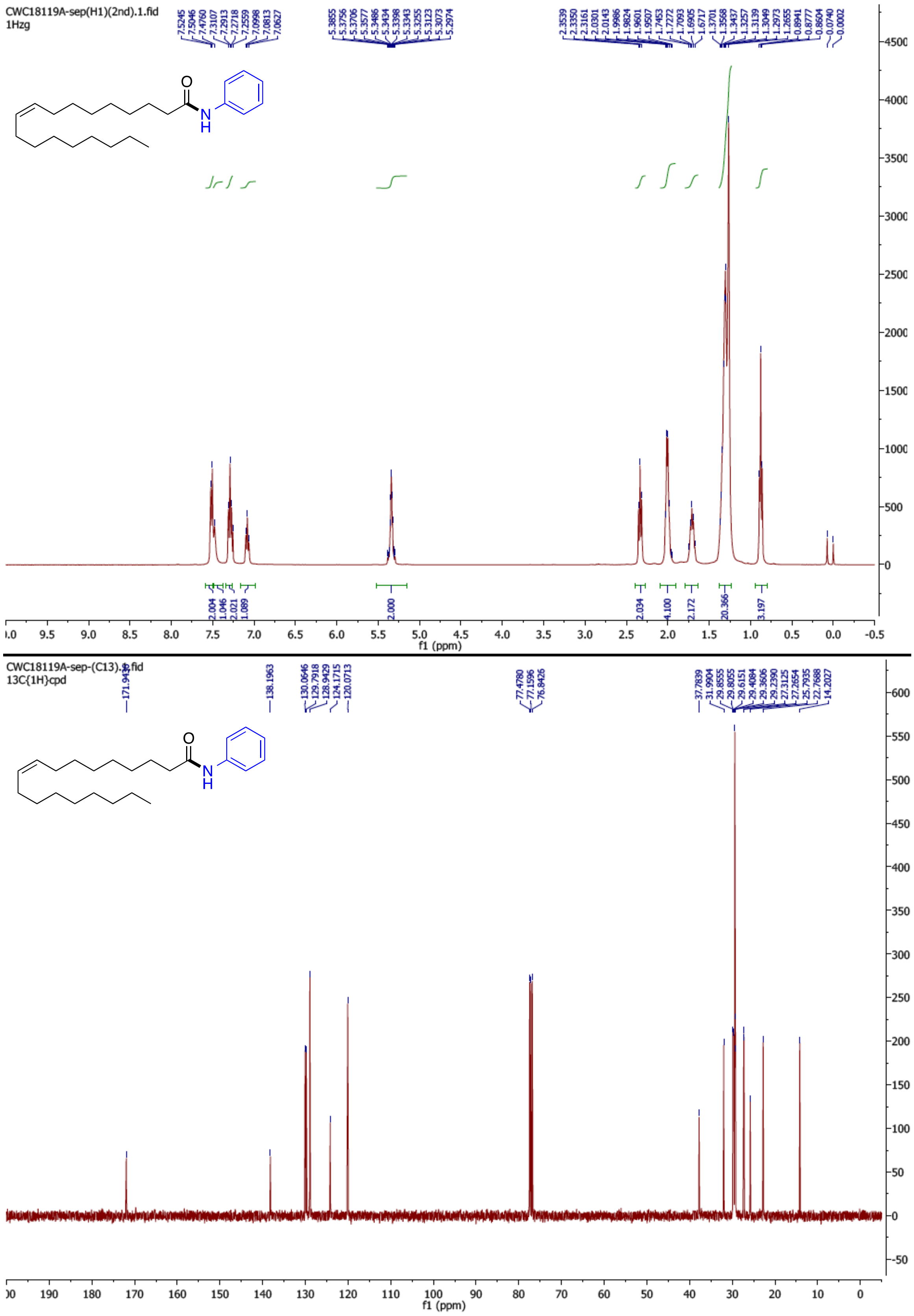

Supplementary Figure 24. ^{1}H and ^{13}C NMR spectra of *N*-Phenyloleamide (3o)

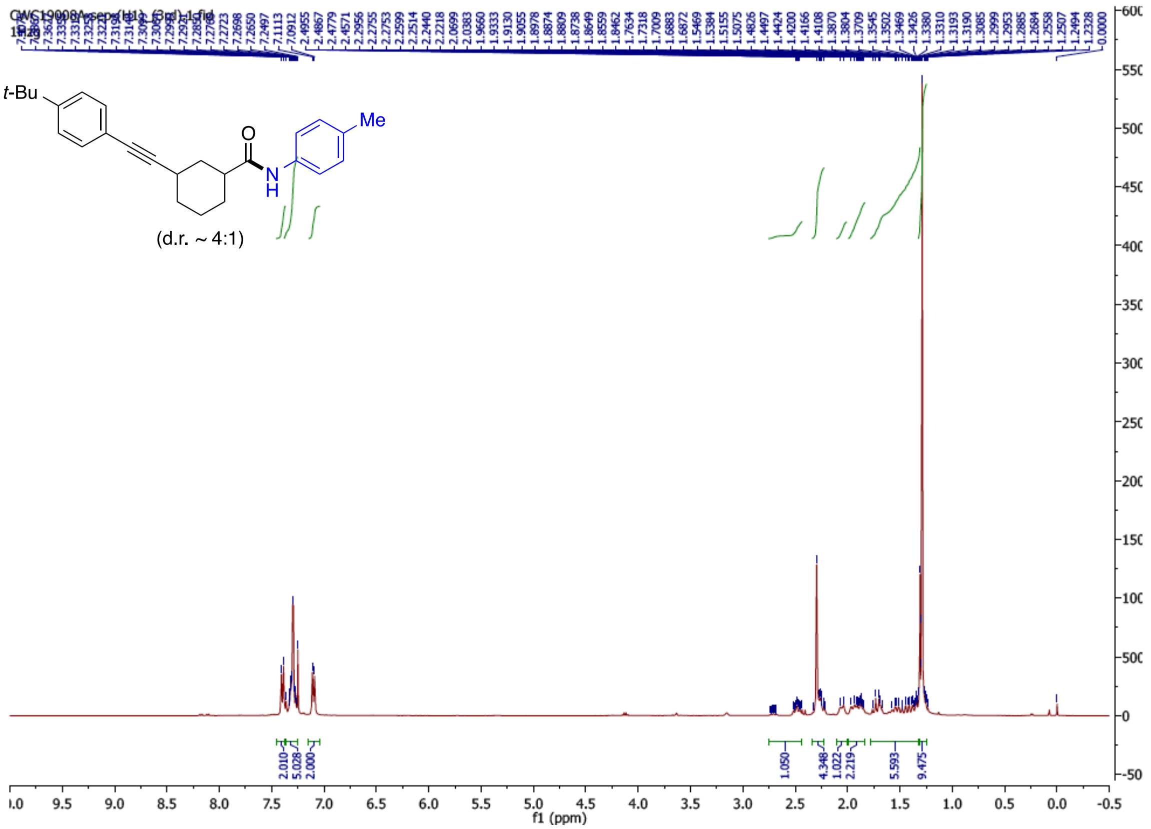

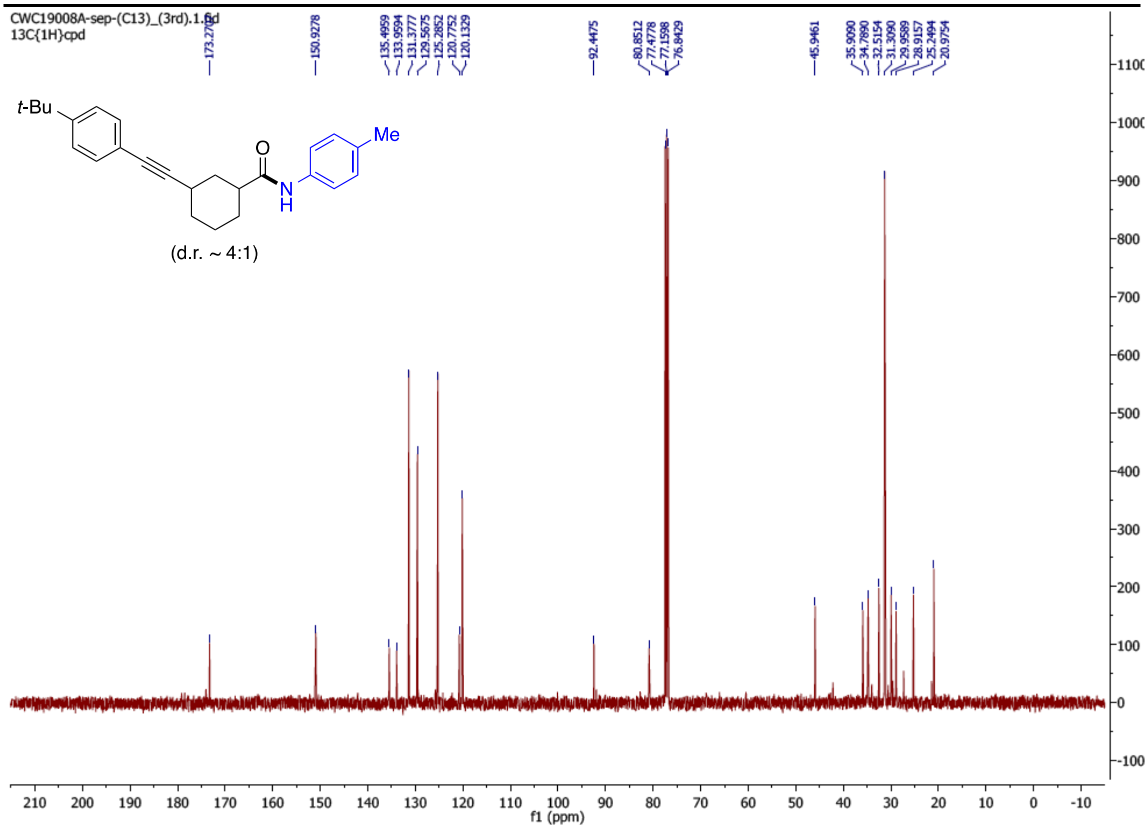

Supplementary Figure 25. **^{1}H and ^{13}C NMR spectra of 3-((4-(*tert*-Butyl)phenyl)ethynyl)-*N*-(*p*-tolyl)cyclohexane-1-carboxamide (3p)**

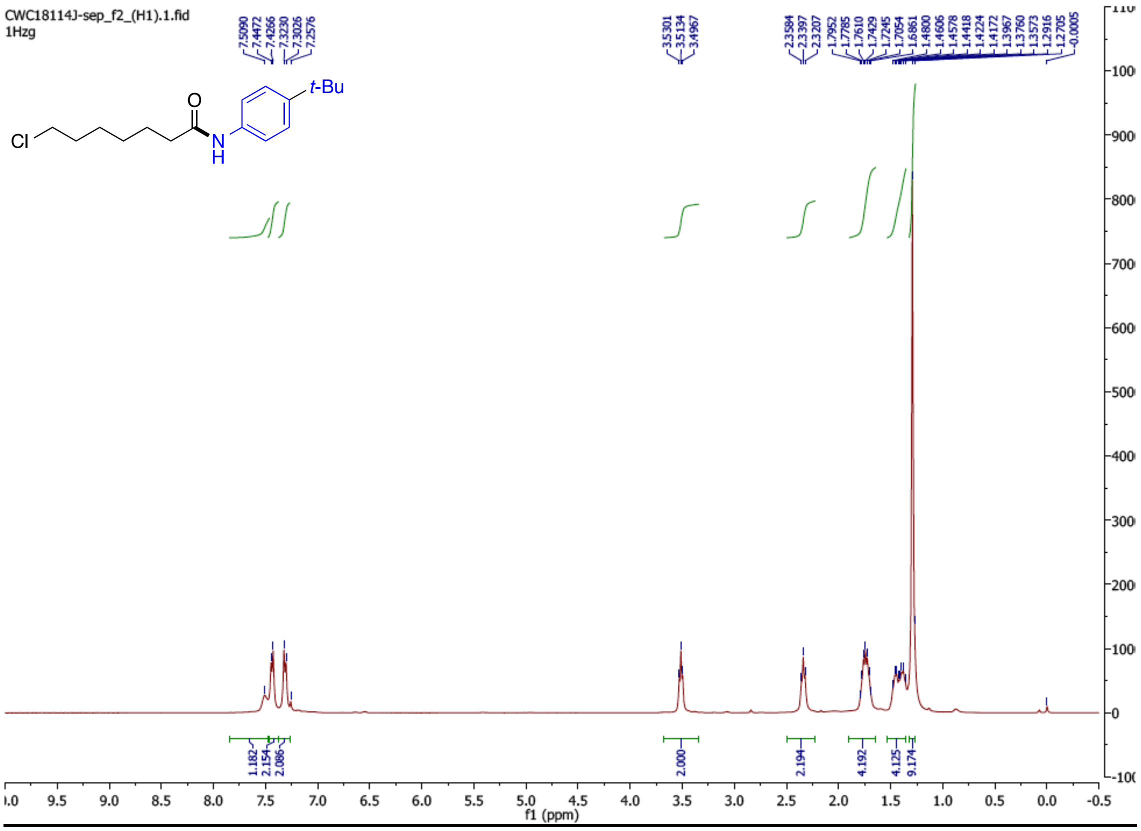

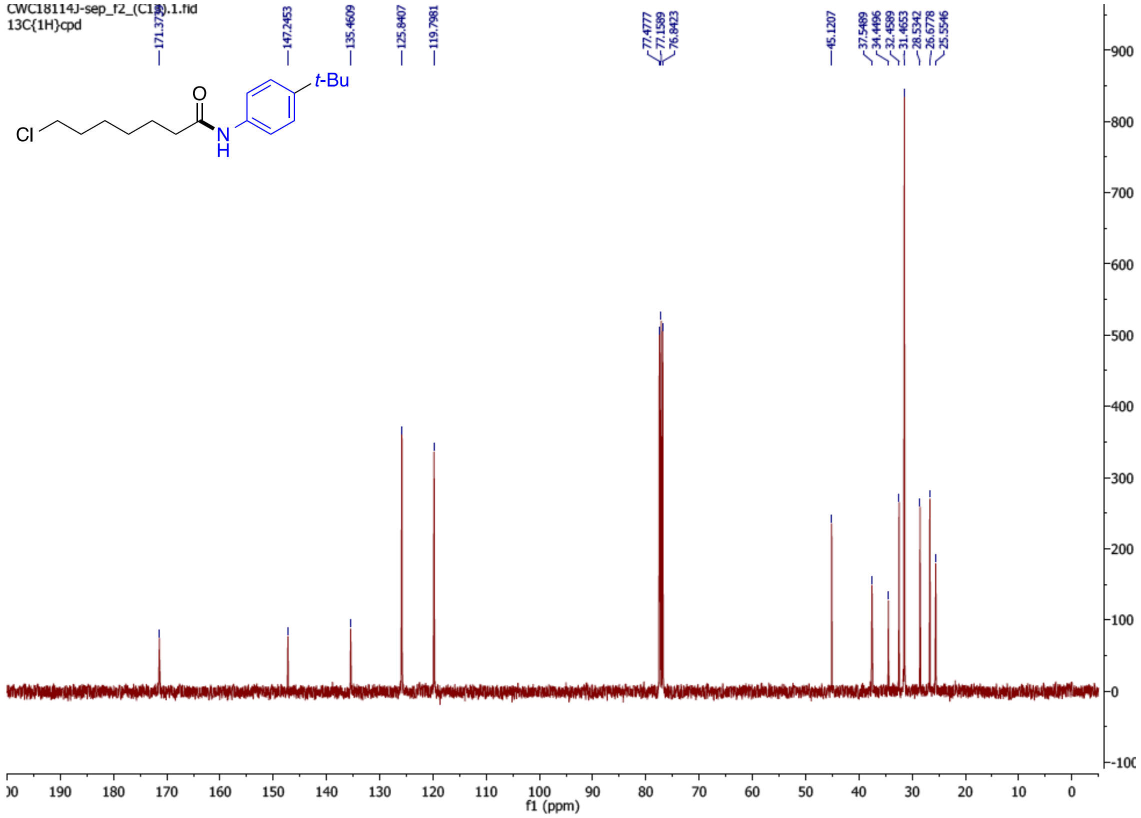

Supplementary Figure 26. ^{1}**H and** ^{13}**C NMR spectra of** ***N*****-(4-(*****tert*****-Butyl)phenyl)-7-chloroheptanamide (3q)**

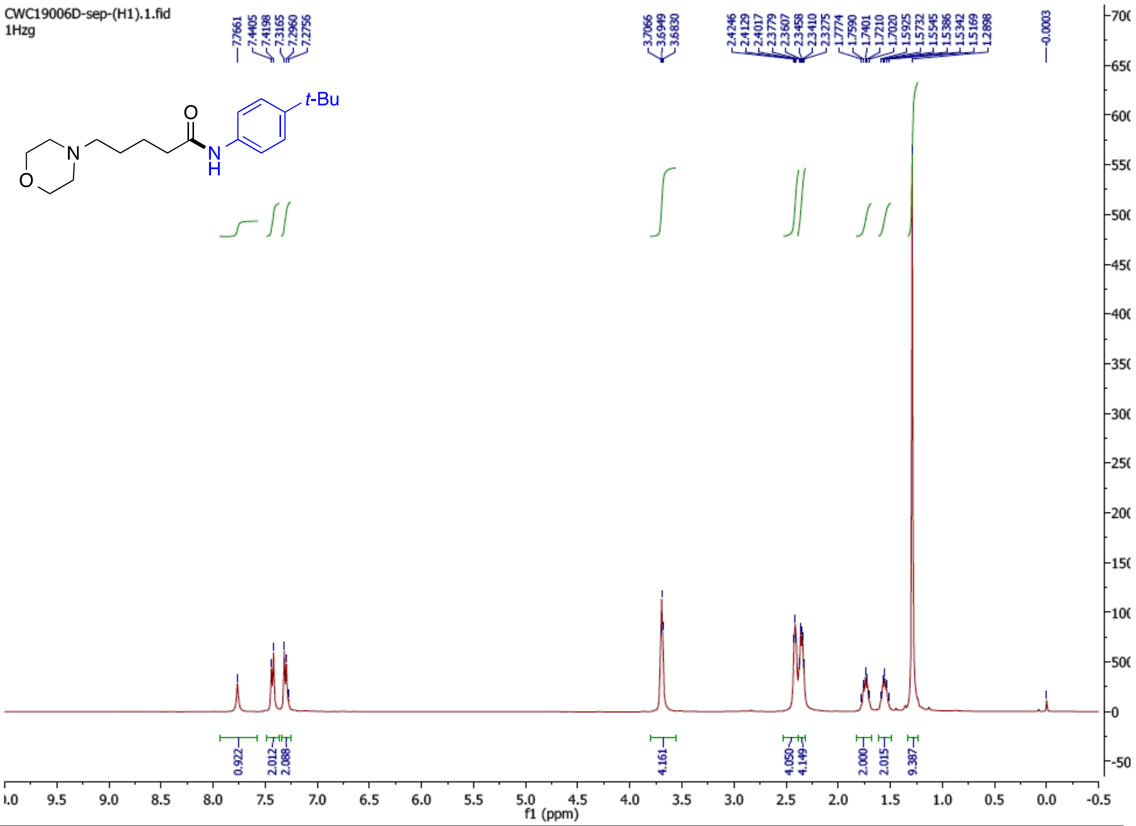

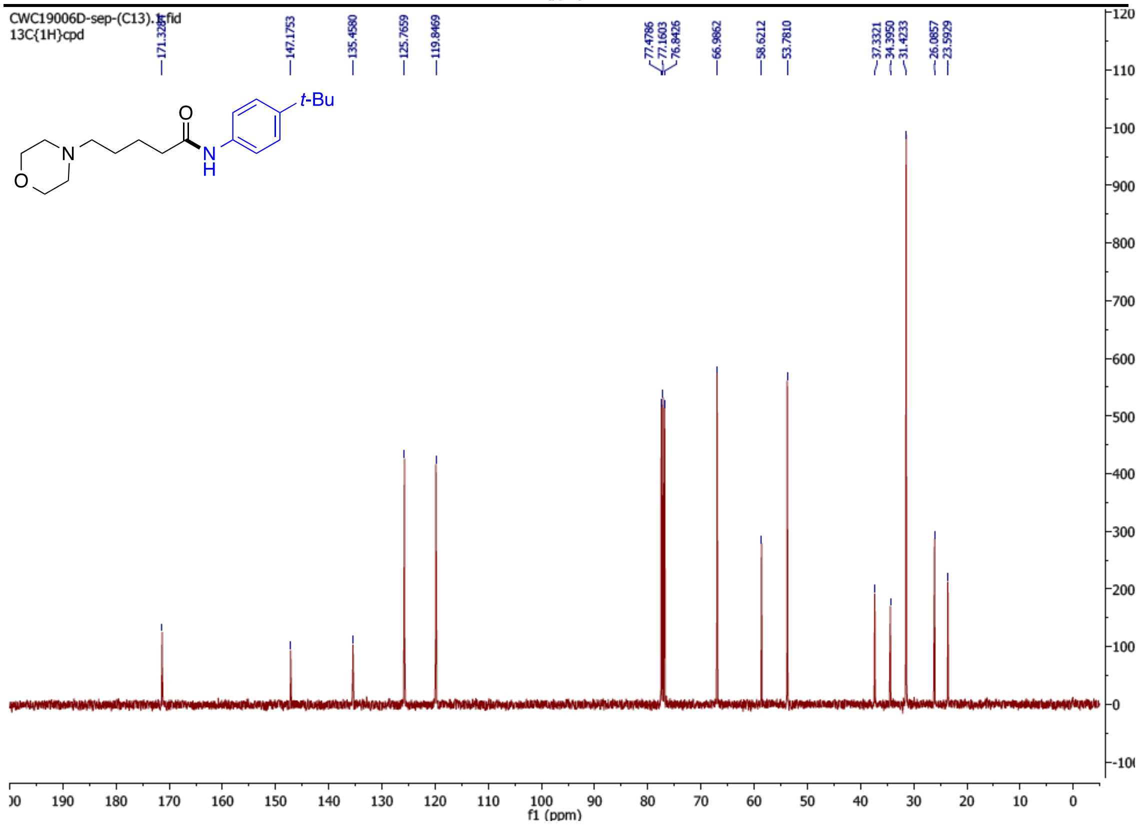

Supplementary Figure 27. ^{1}H and ^{13}C NMR spectra of *N*-(4-(*tert*-Butyl)phenyl)-5-morpholinopentanamide (3r)

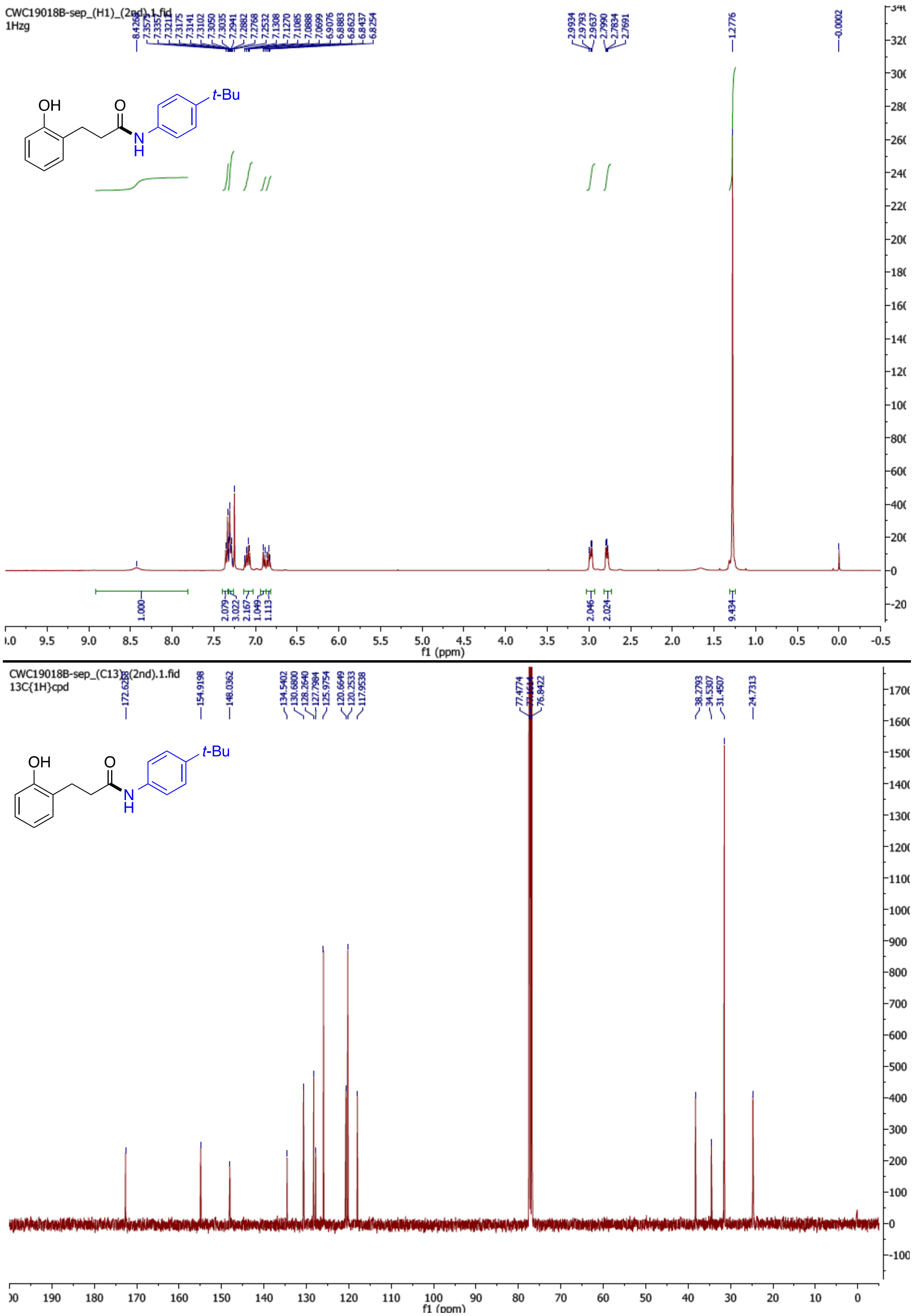

Supplementary Figure 28. ^{1}**H and** ^{13}**C NMR spectra of *N*-(4-(*tert*-Butyl)phenyl)-3-(2-hydroxyphenyl)propanamide (3s)**

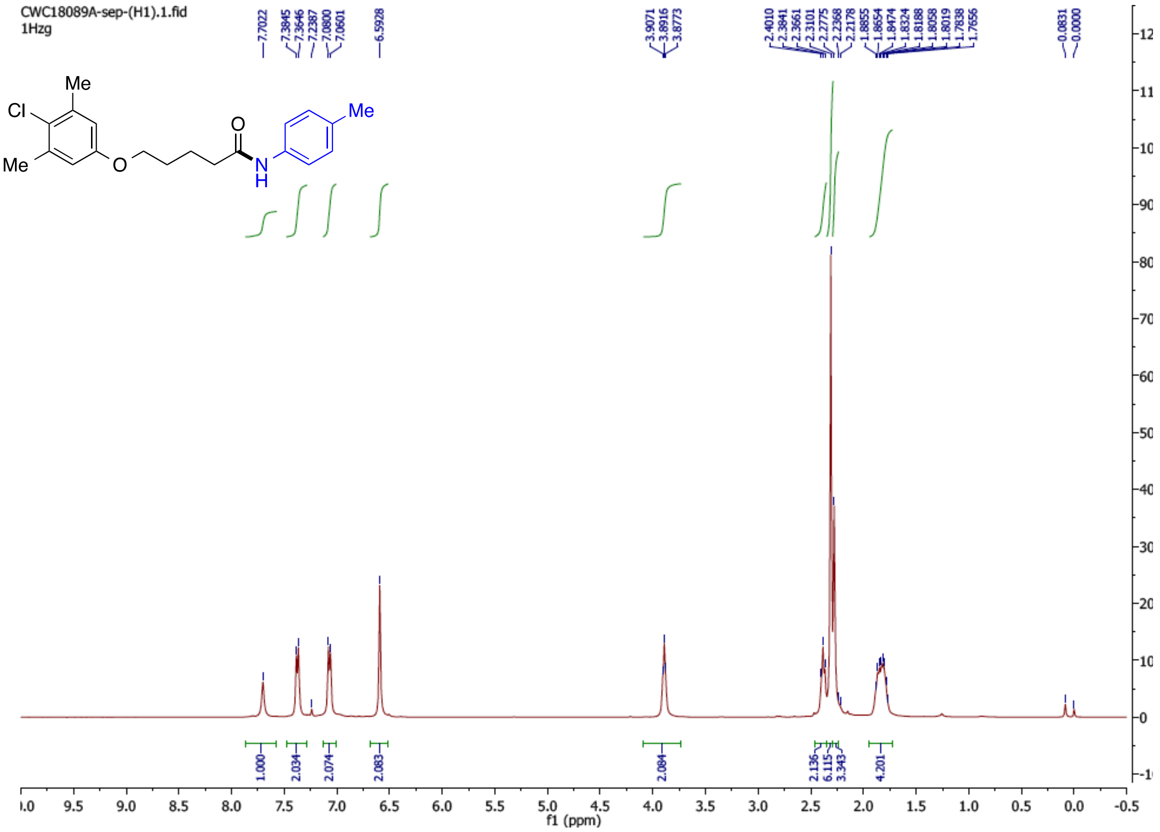

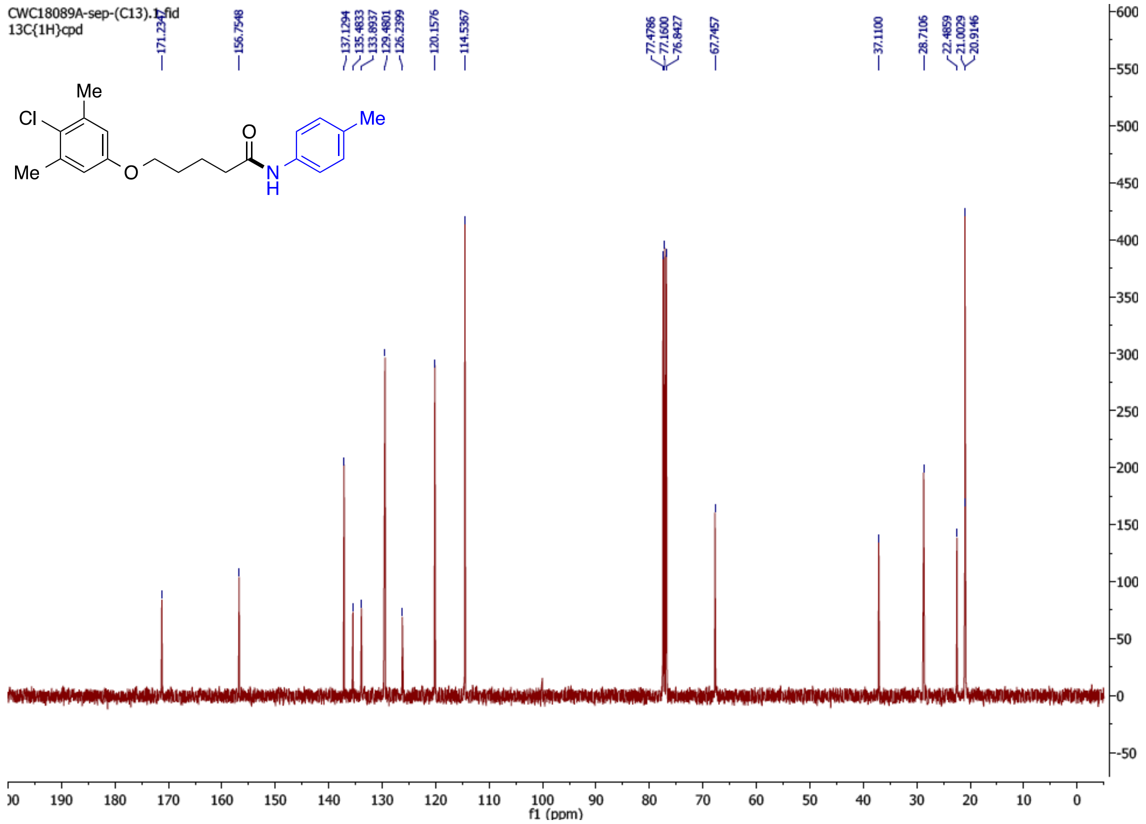

Supplementary Figure 29. ^{1}H and ^{13}C NMR spectra of 5-(4-Chloro-3,5-dimethylphenoxy)-*N*-(*p*-tolyl)pentanamide (3t)

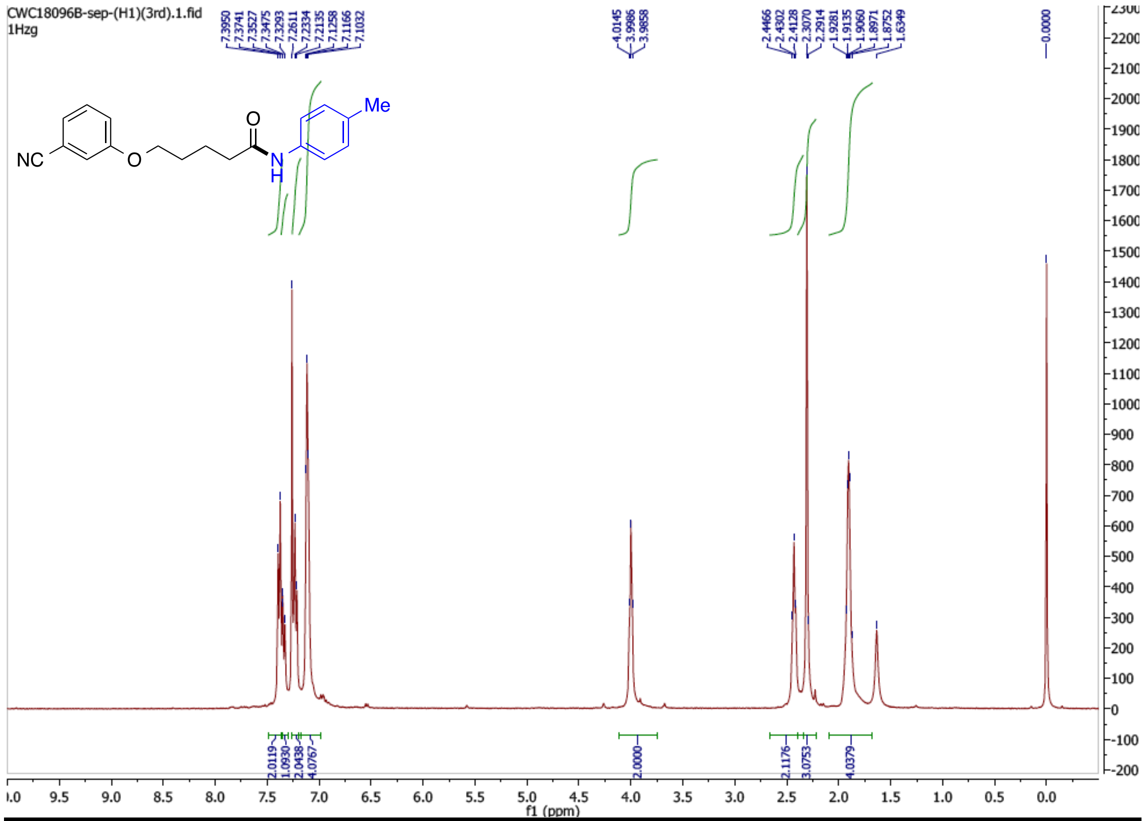

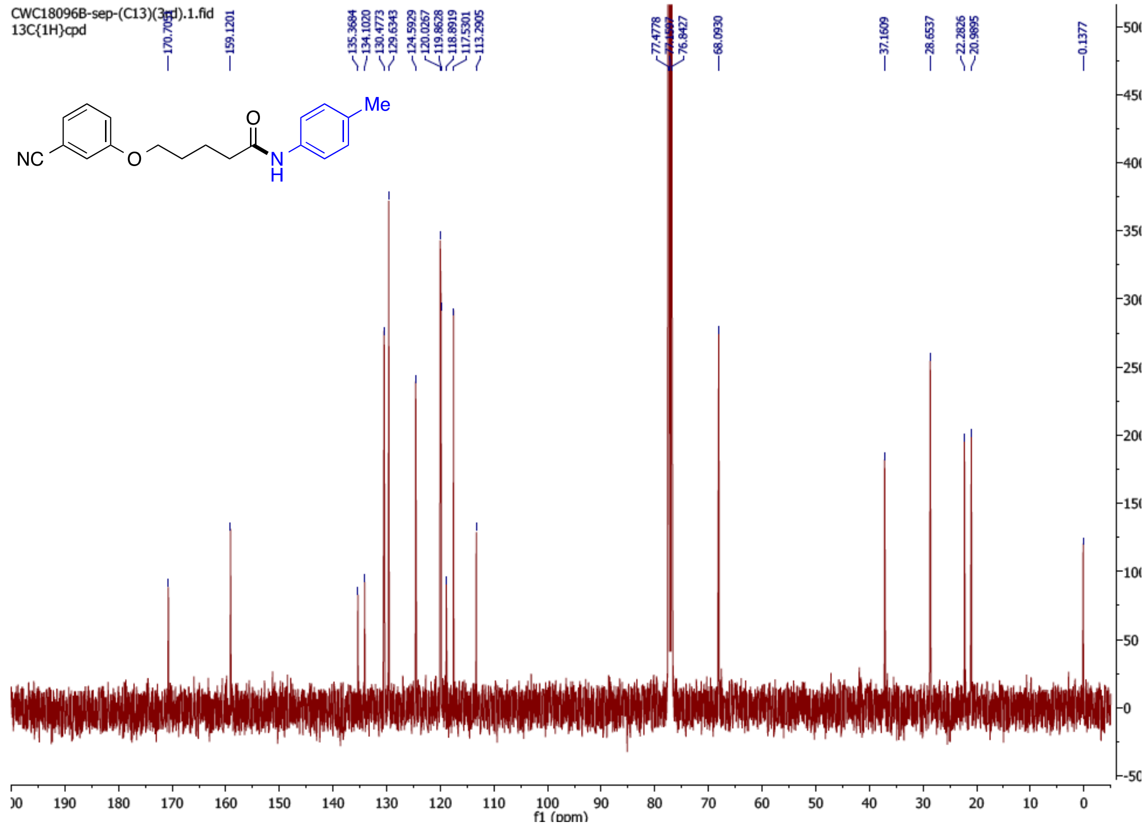

Supplementary Figure 30. ^{1}H and ^{13}C NMR spectra of 5-(3-Cyanophenoxy)-*N*-(*p*-tolyl)pentanamide (3u)

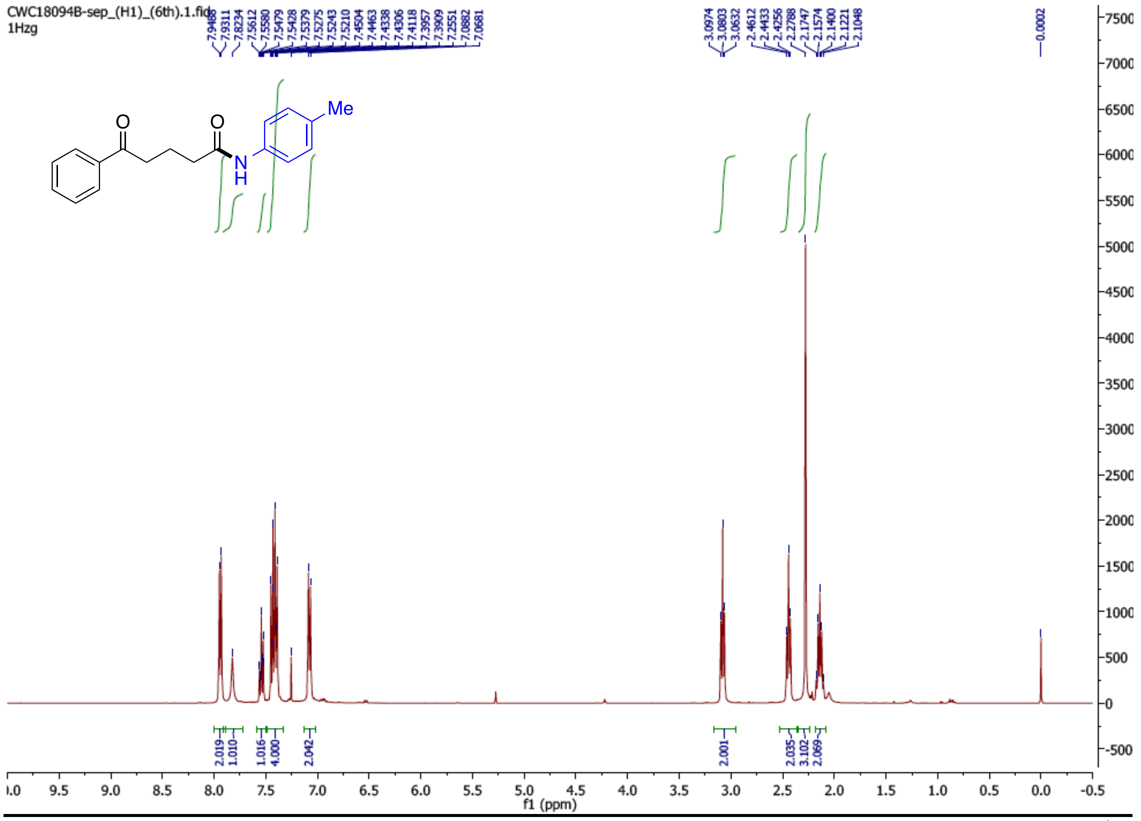

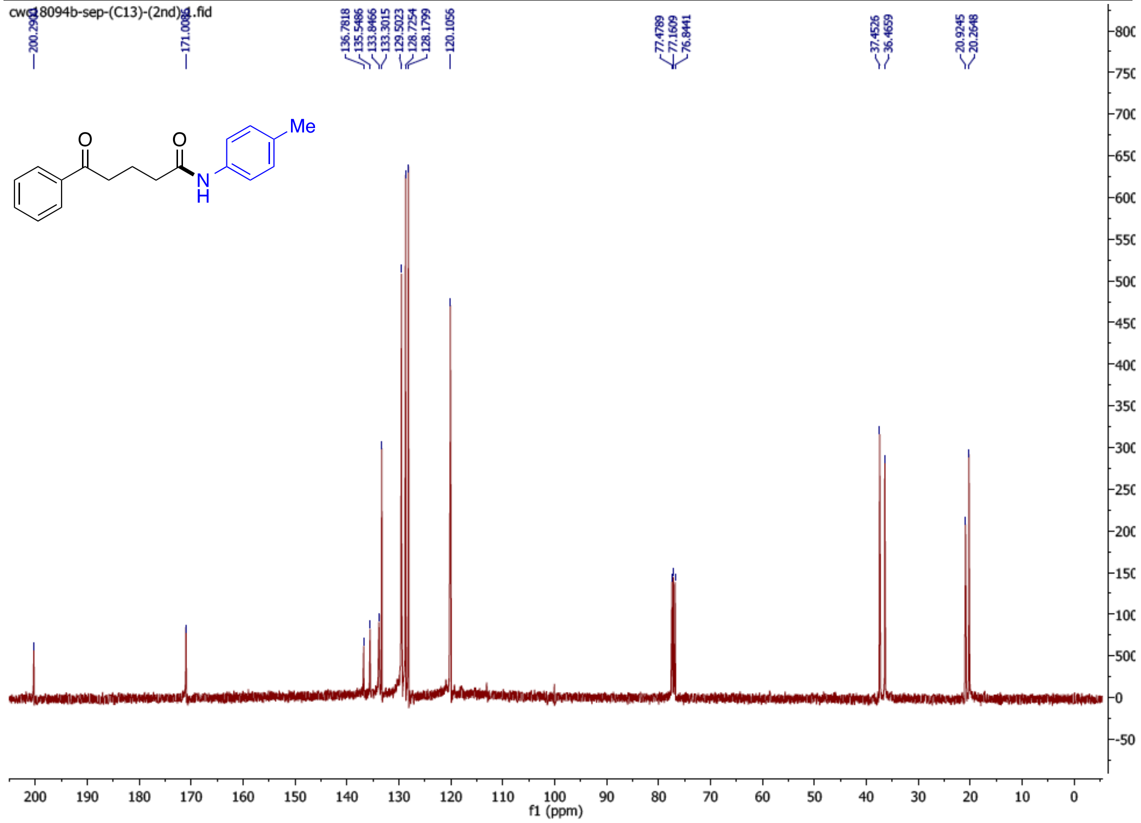

Supplementary Figure 31. ^{1}H and ^{13}C NMR spectra of 5-Oxo-5-phenyl-*N*-(*p*-tolyl)pentanamide (3v)

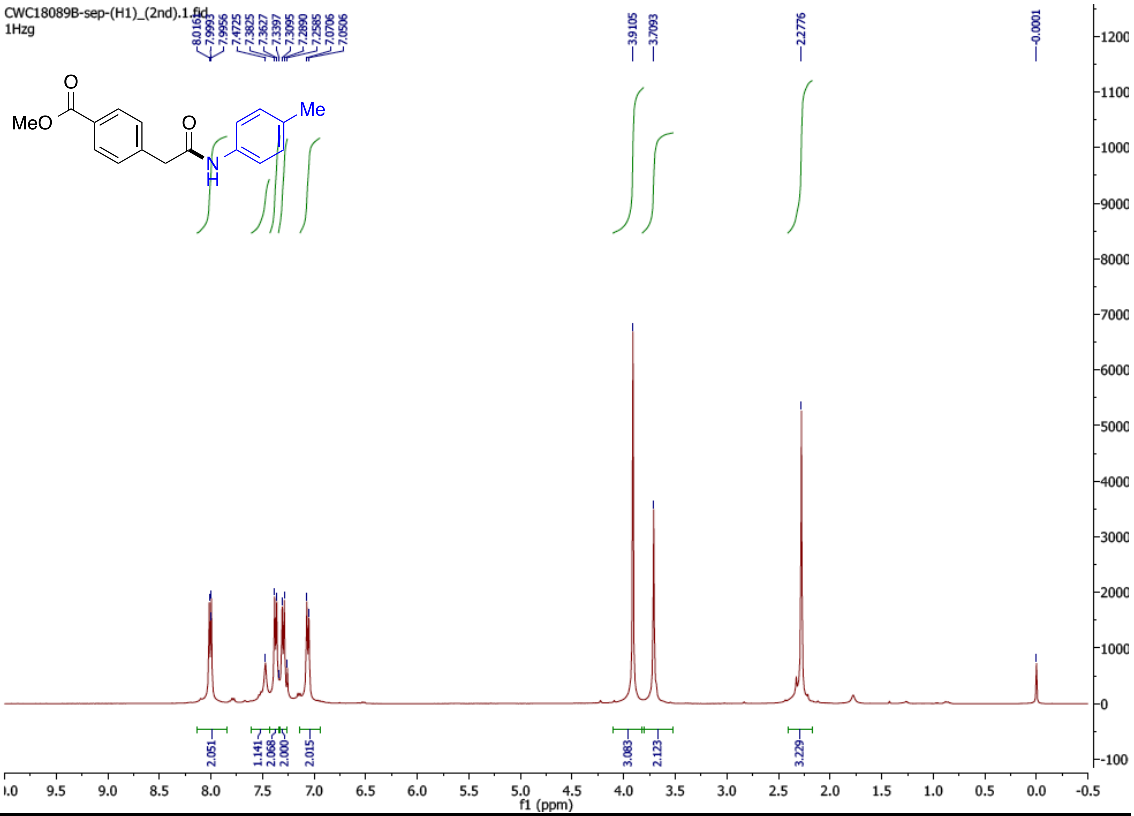

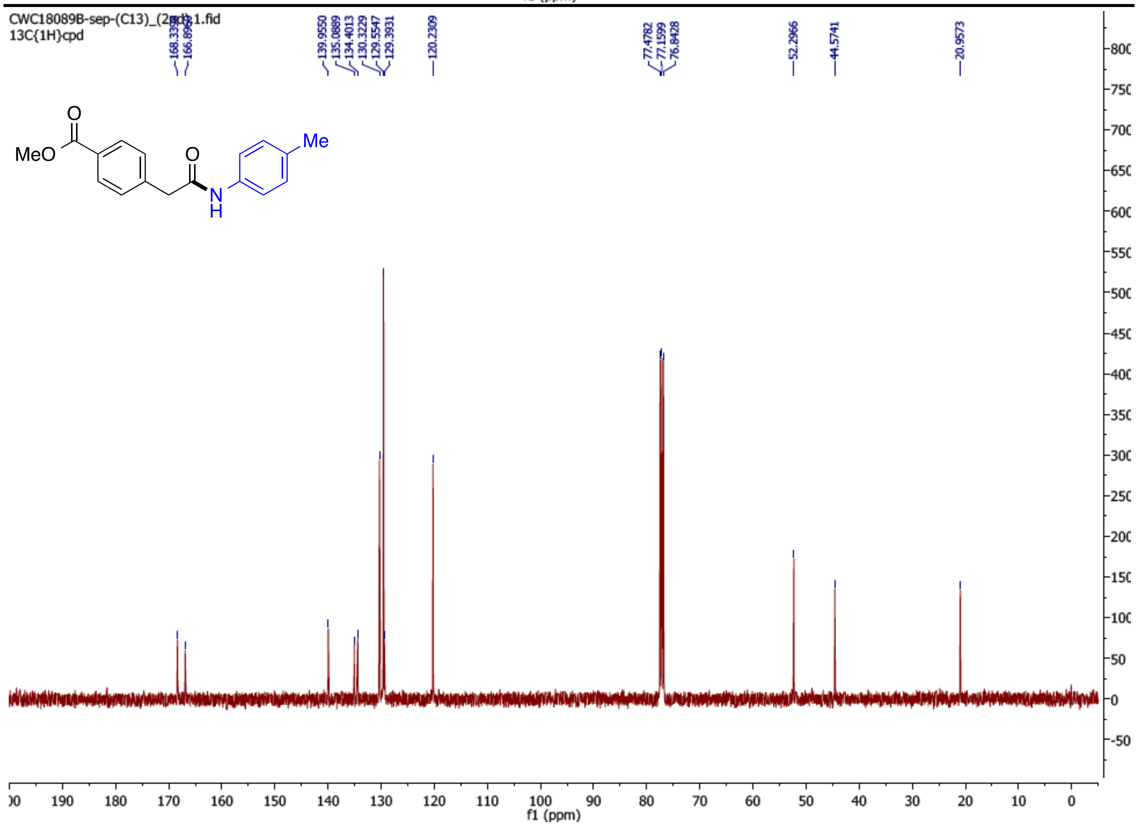

Supplementary Figure 32. **^{1}H and ^{13}C NMR spectra of Methyl 4-(2-Oxo-2-(*p*-tolylamino)ethyl)benzoate (3w)**

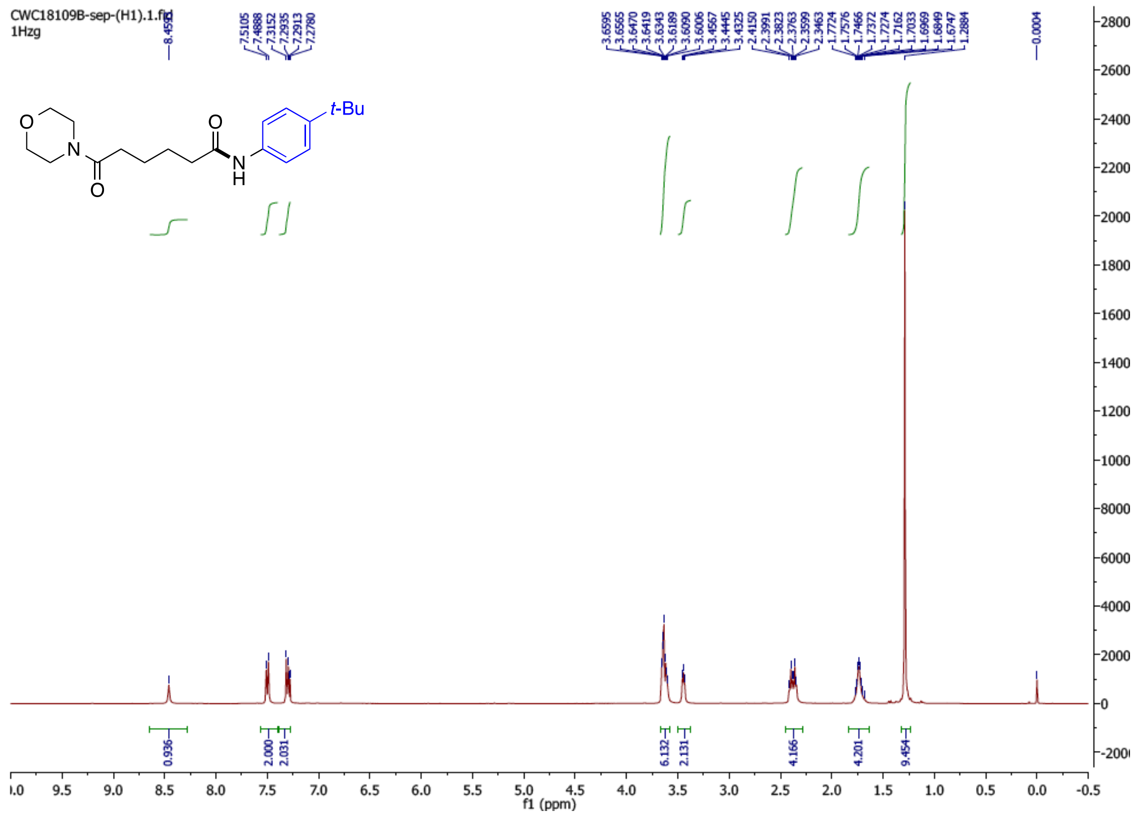

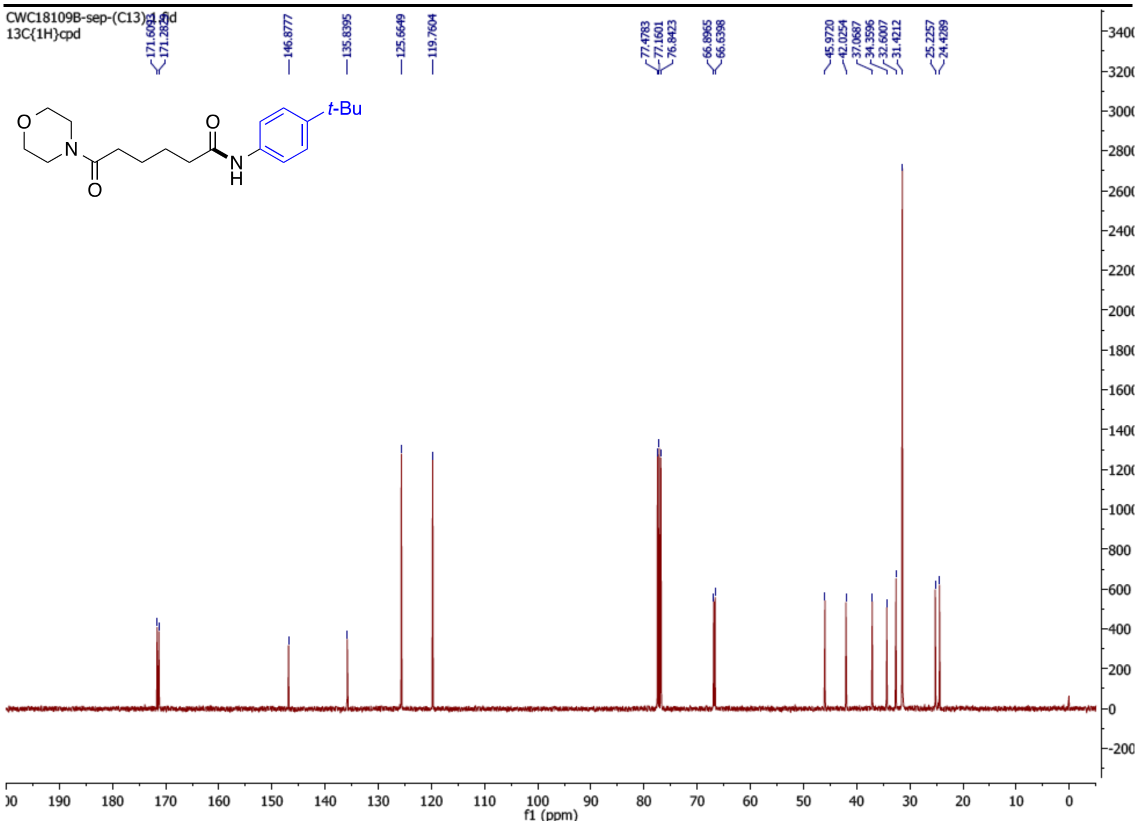

Supplementary Figure 33. ^{1}H and ^{13}C NMR spectra of *N*-(4-(*tert*-Butyl)phenyl)-6-morpholino-6-oxohexanamide (3x)

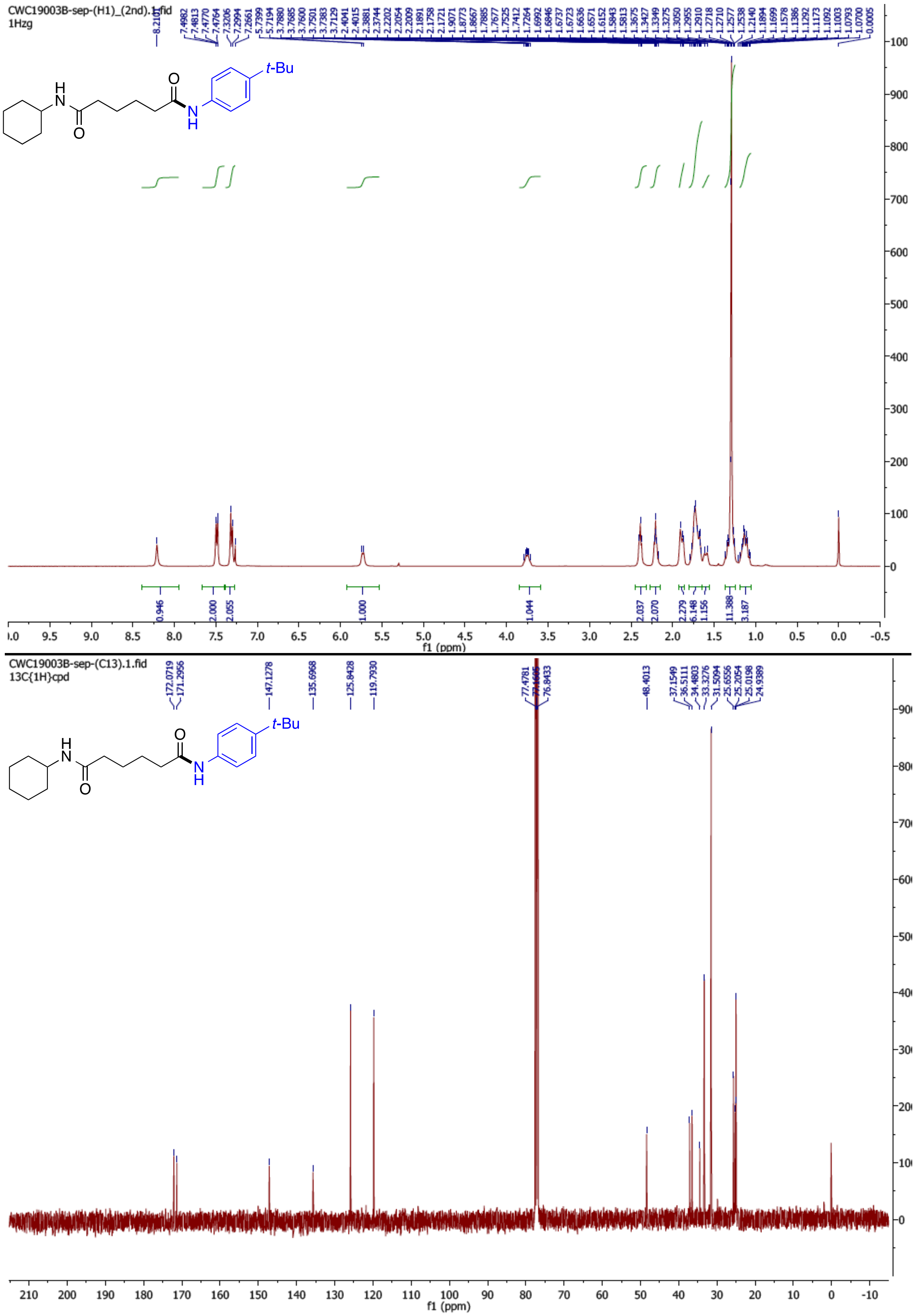

Supplementary Figure 34. ^{1}**H and** ^{13}**C NMR spectra of** ***N***^{1}**-(4-(*****tert*****-Butyl)phenyl)-*****N***^{6}**-cyclohexyladipamide (3y)**

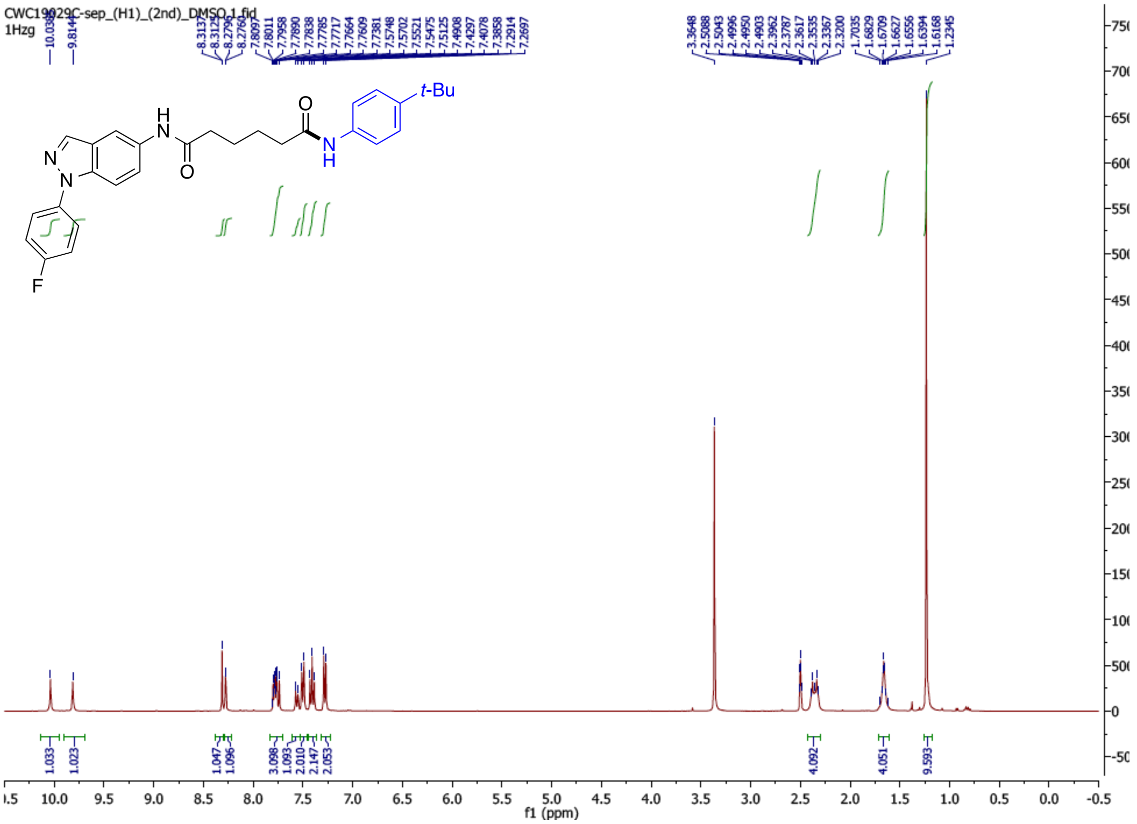

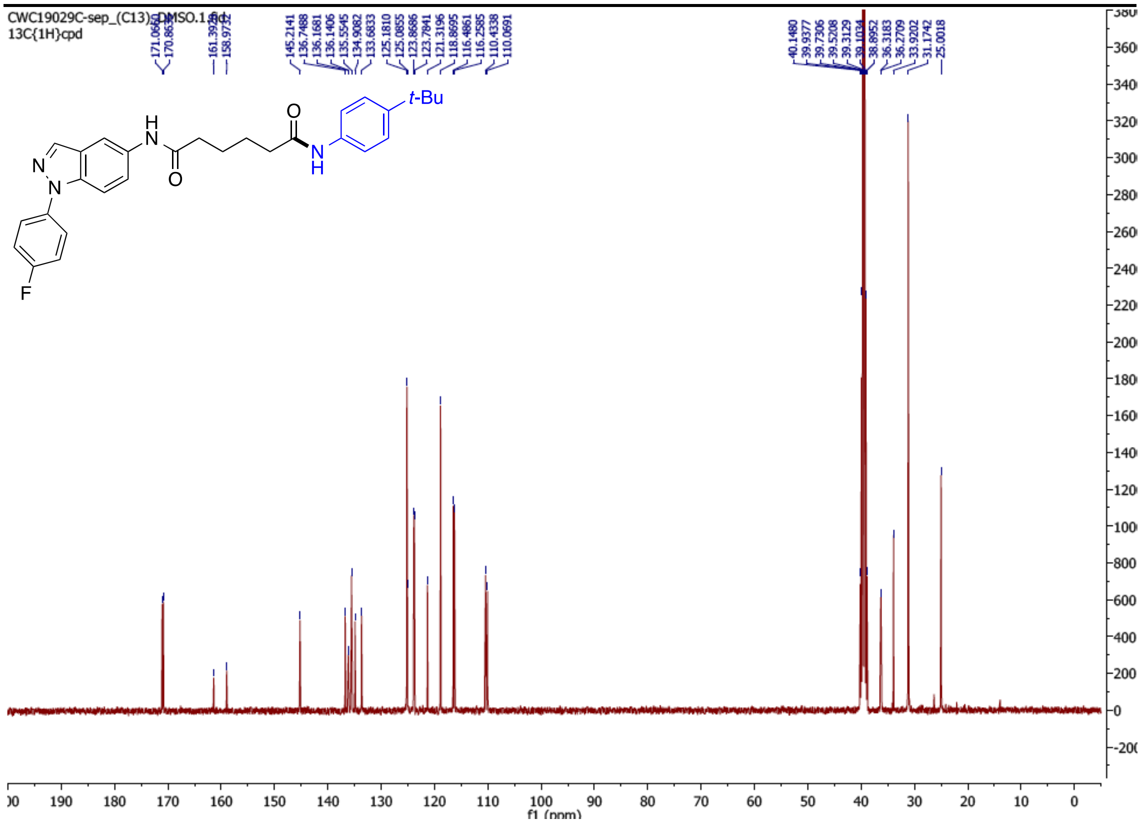

Supplementary Figure 35. ^{1}H and ^{13}C NMR spectra of *N*^{1}-(4-(*tert*-Butyl)phenyl)-*N*^{6}-(1-(4-fluorophenyl)-1*H*-indazol-5-yl)adipamide (3z)

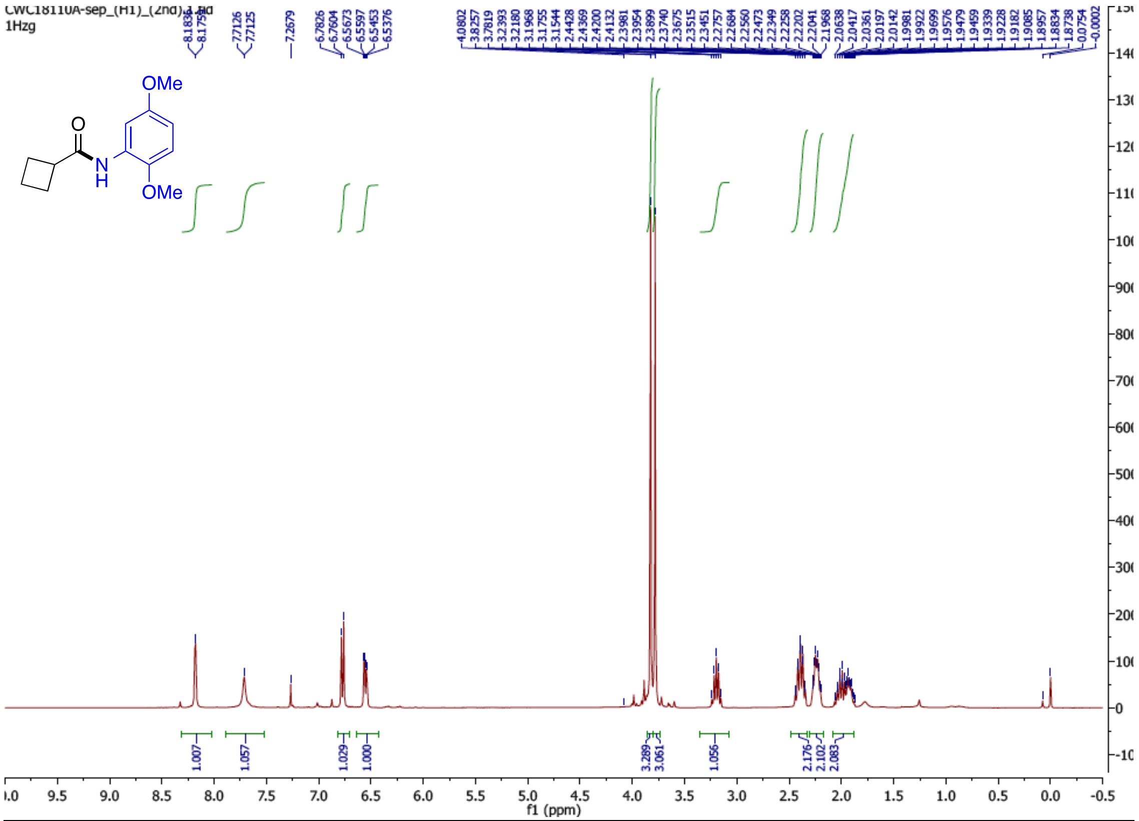

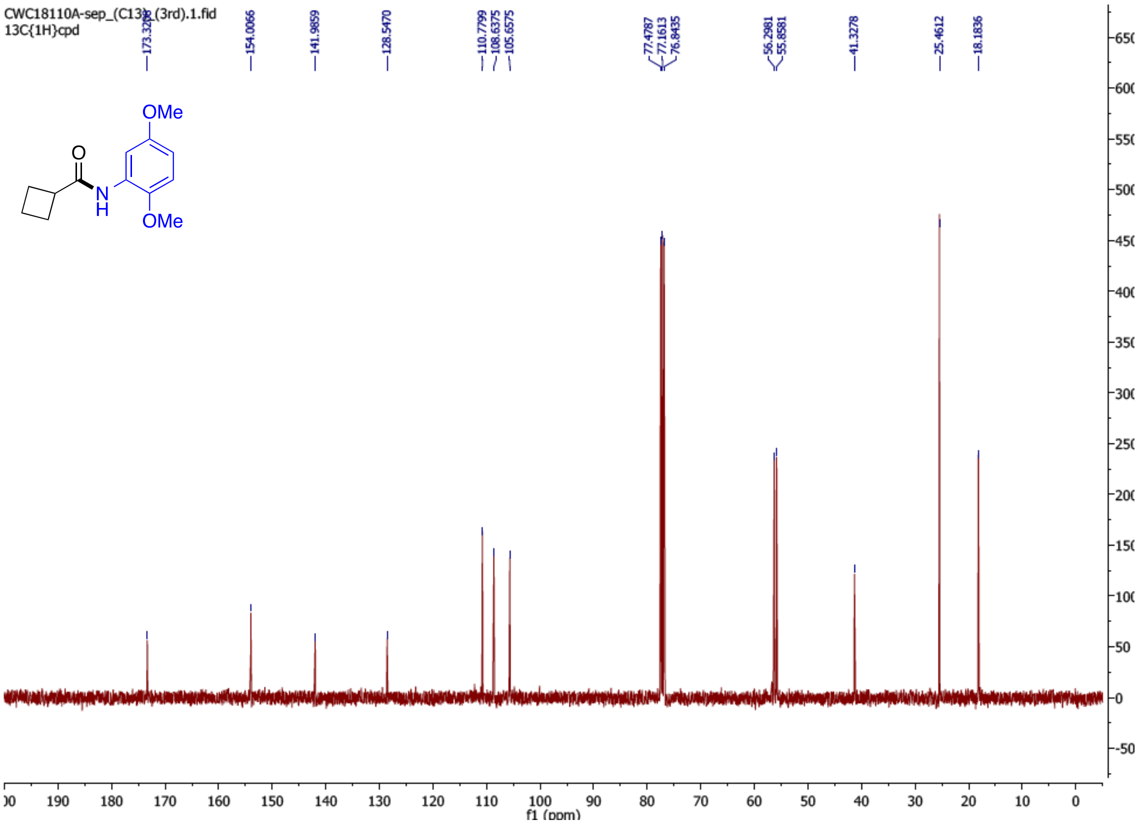

Supplementary Figure 36. ^{1}H and ^{13}C NMR spectra of *N*-(2,5-Dimethoxyphenyl)cyclobutanecarboxamide (4a)

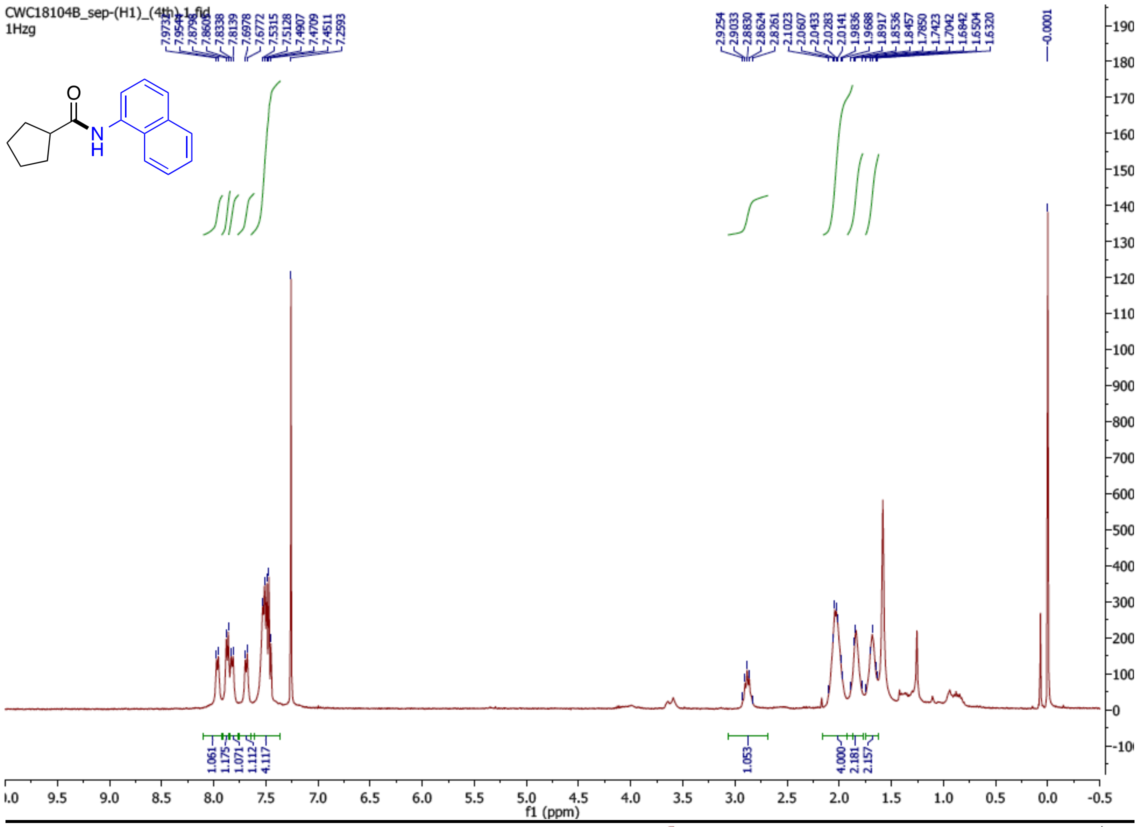

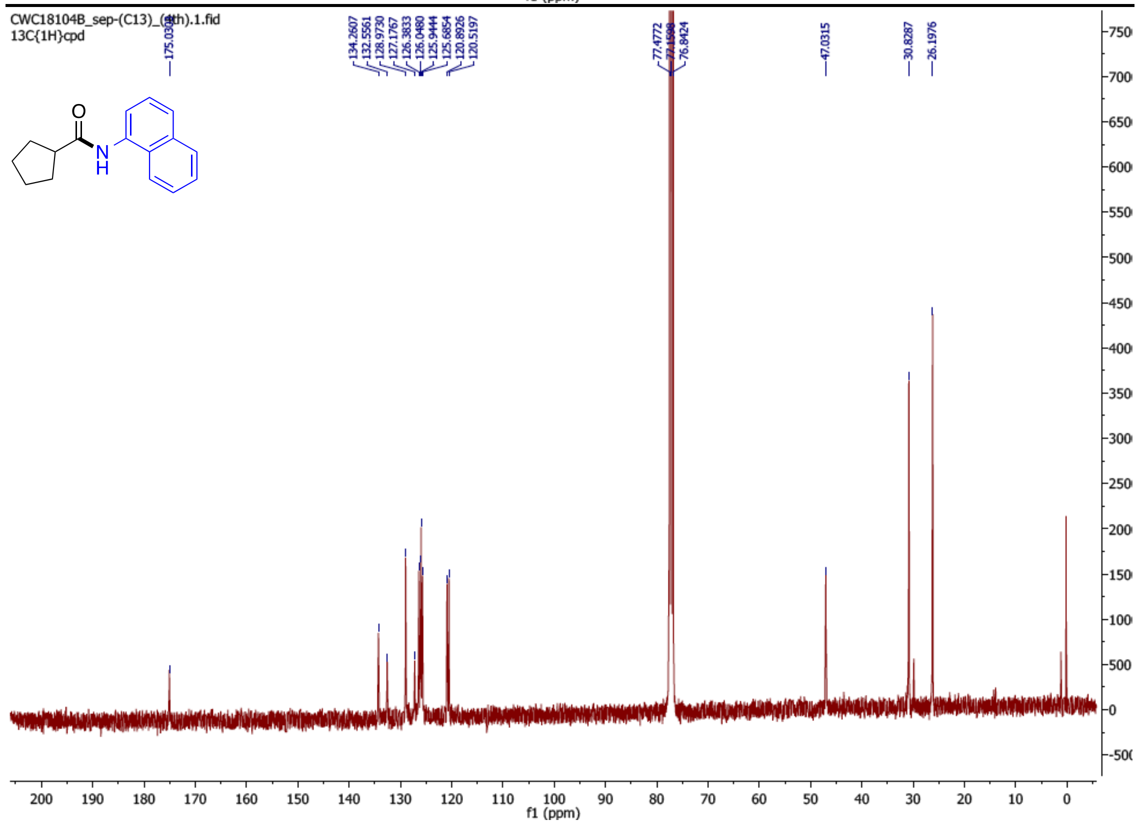

Supplementary Figure 37. ^{1}H and ^{13}C NMR spectra of *N*-(Naphthalen-1-yl)cyclopentanecarboxamide (4b)

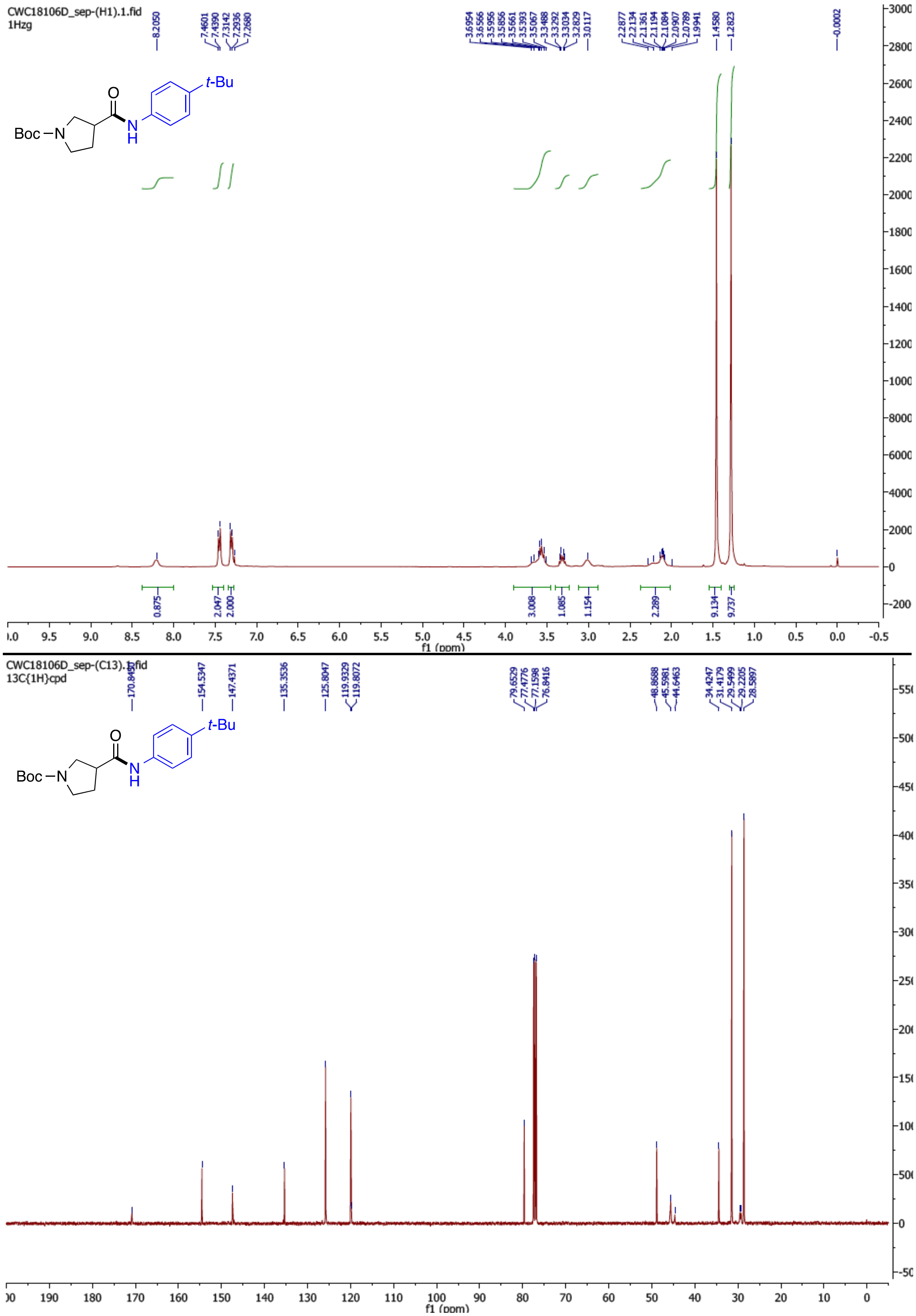

Supplementary Figure 38. **^{1}H and ^{13}C NMR spectra of *tert*-Butyl 3-((4-(*tert*-butyl)phenyl)carbamoyl)pyrrolidine-1-carboxylate (4c)**

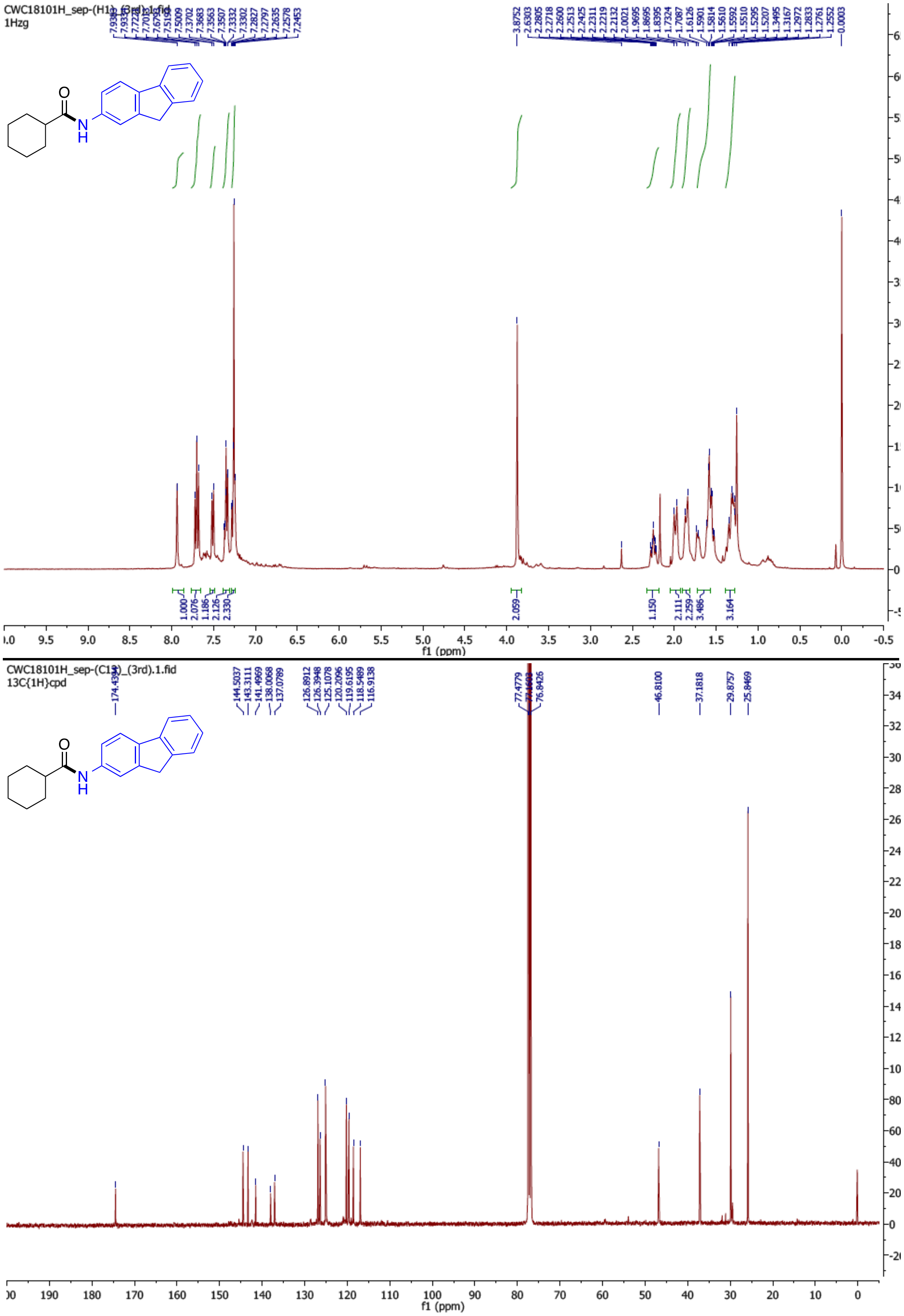

Supplementary Figure 39. **^{1}H and ^{13}C NMR spectra of *N*-(9*H*-Fluoren-2-yl)cyclohexanecarboxamide (4d)**

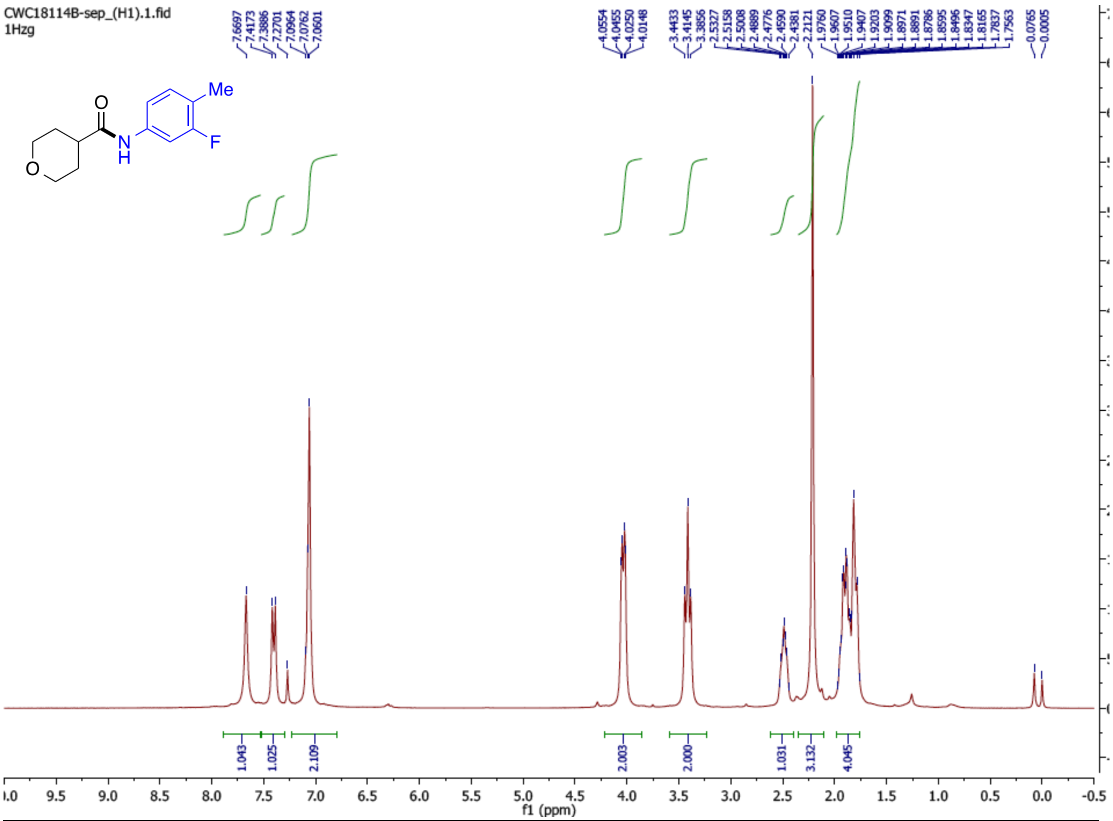

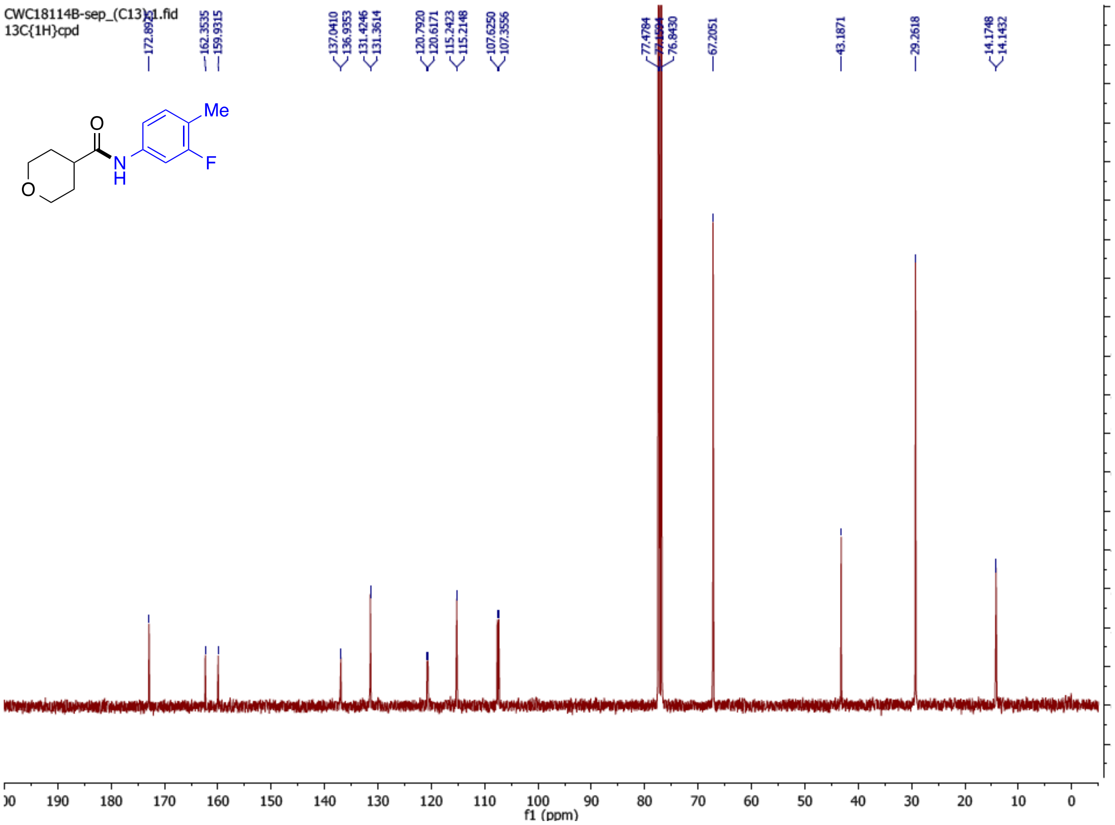

Supplementary Figure 40. ¹H and ¹³C NMR spectra of *N*-(3-Fluoro-4-methylphenyl)tetrahydro-2*H*-pyran-4-carboxamide (4e)

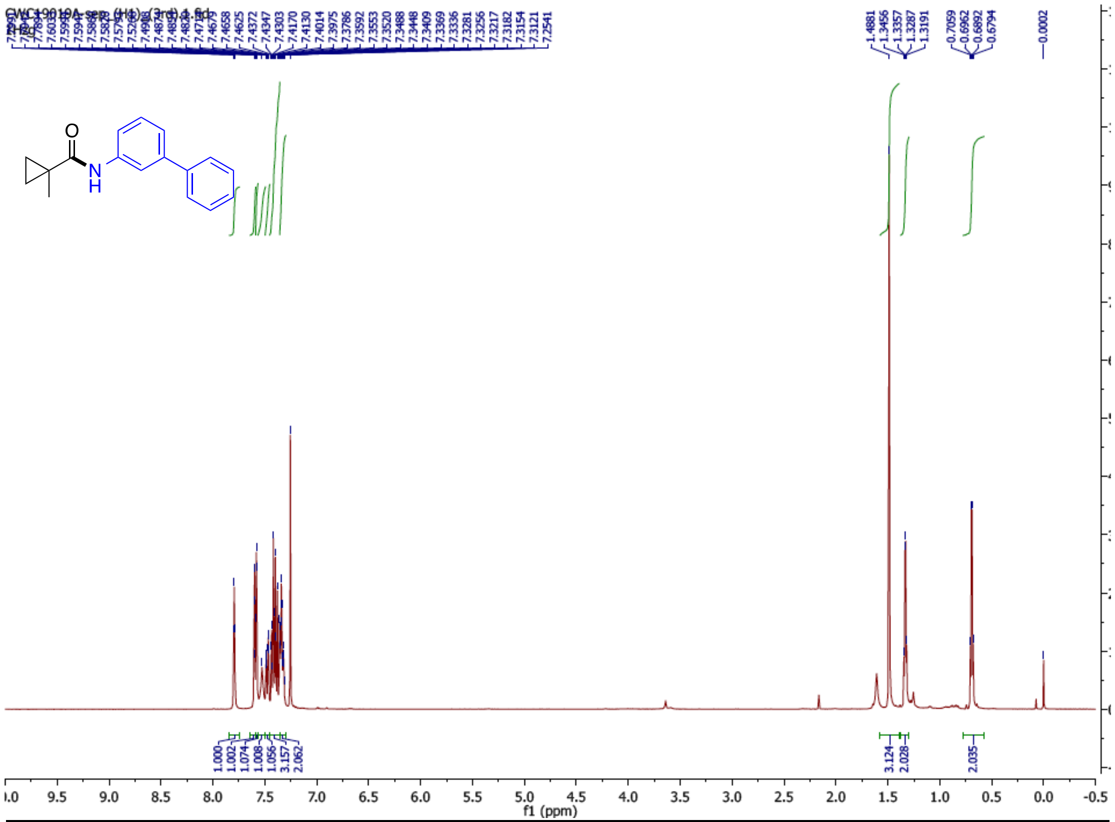

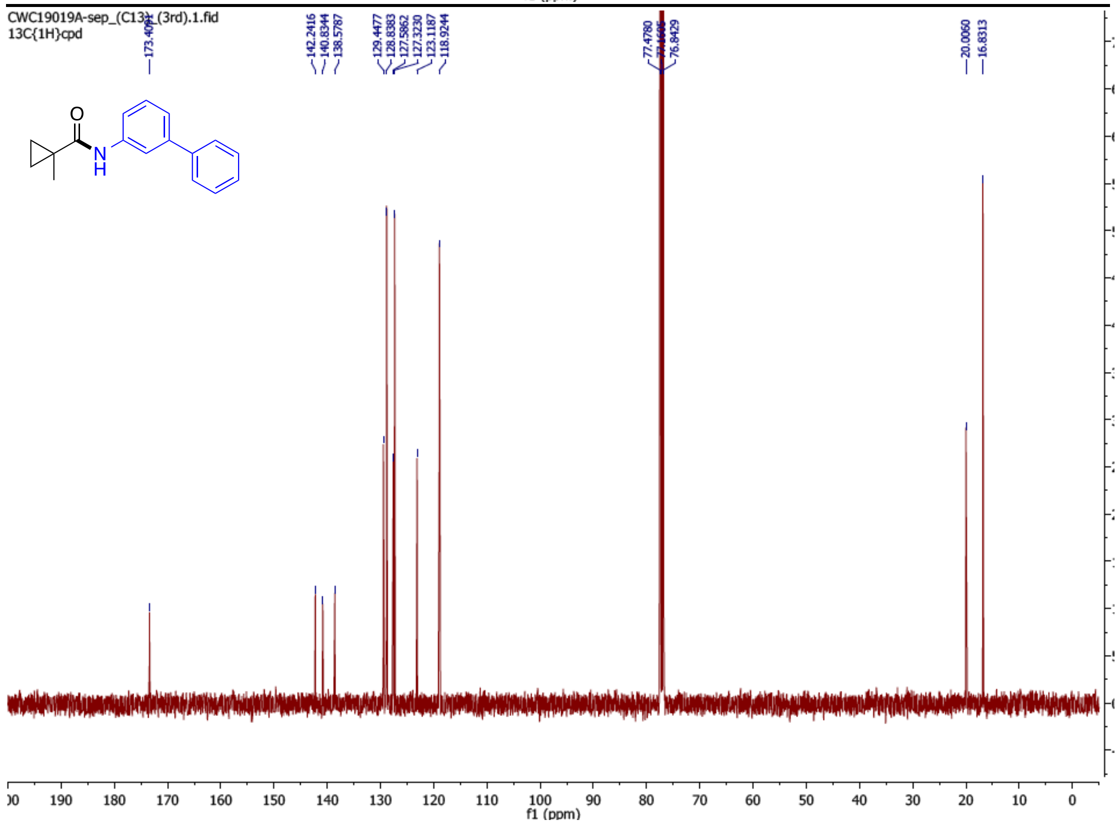

Supplementary Figure 41. ^{1}H and ^{13}C NMR spectra of *N*-([1,1'-Biphenyl]-3-yl)-1-methylcyclopropane-1-carboxamide (4f)

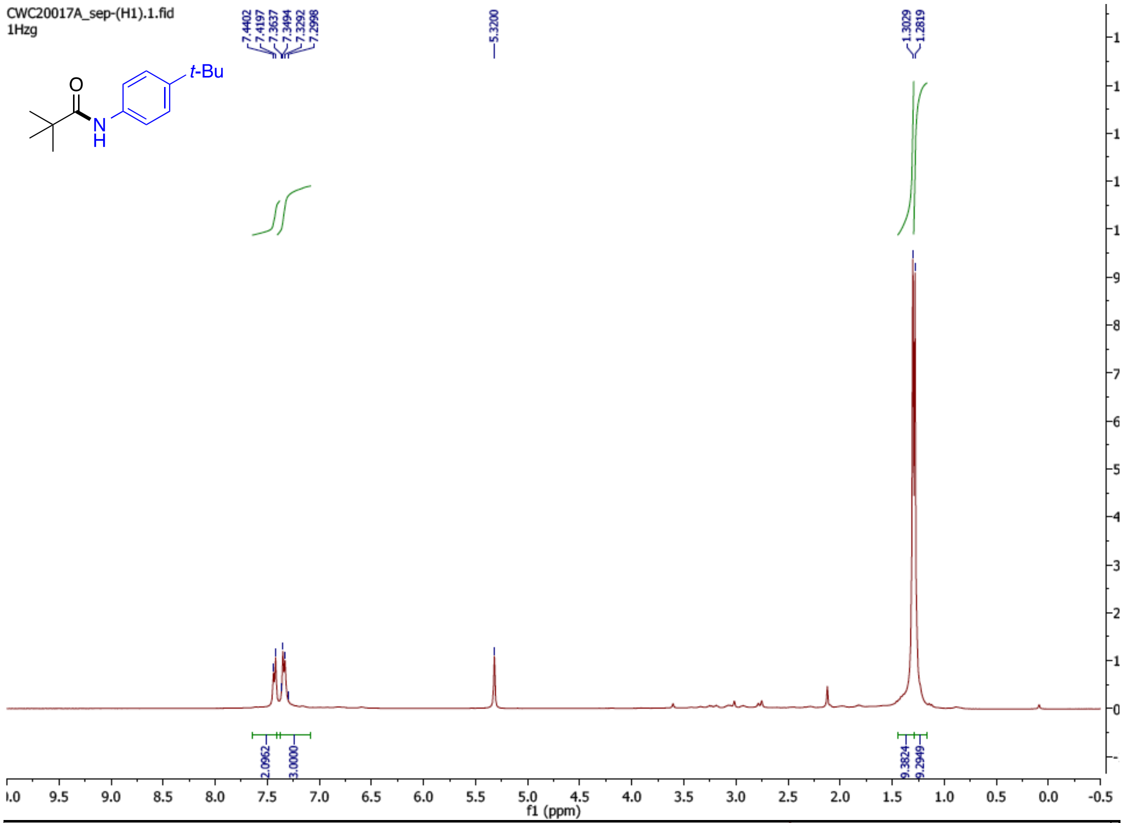

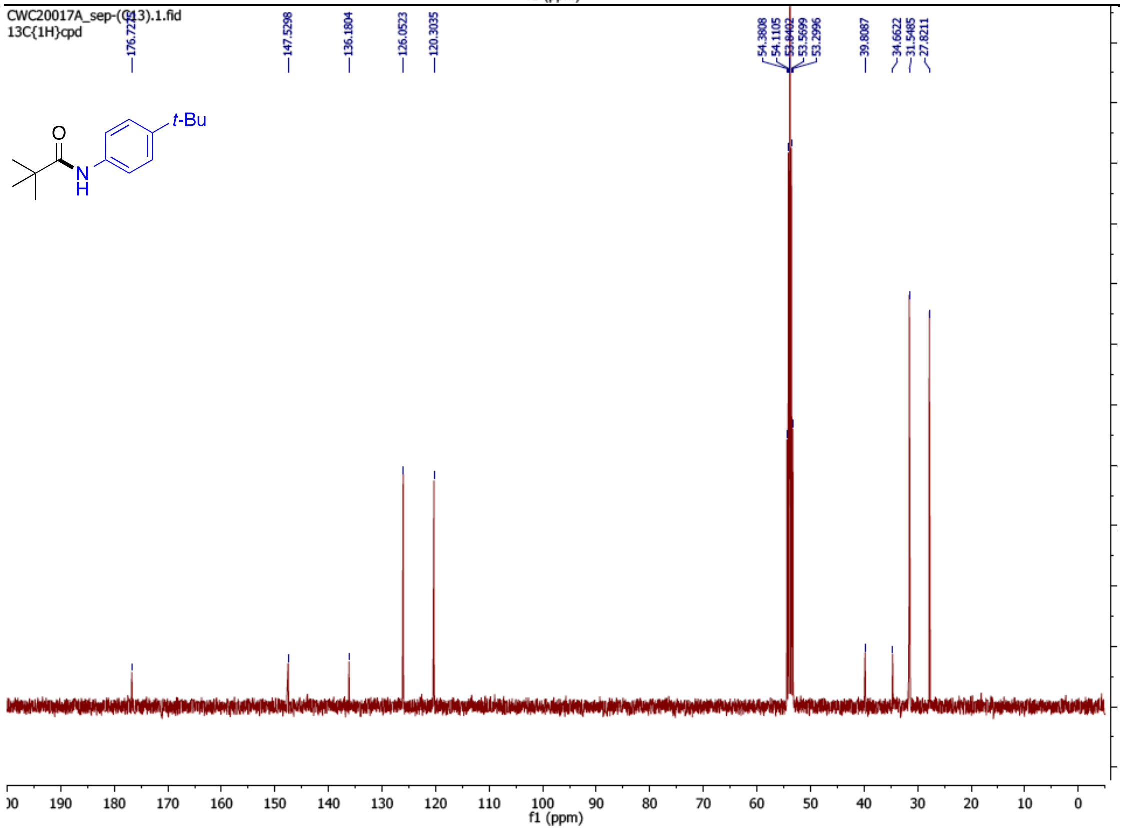

Supplementary Figure 42. ^{1}H and ^{13}C NMR spectra of *N*-(4-(*tert*-butyl)phenyl)pivalamide (4g)

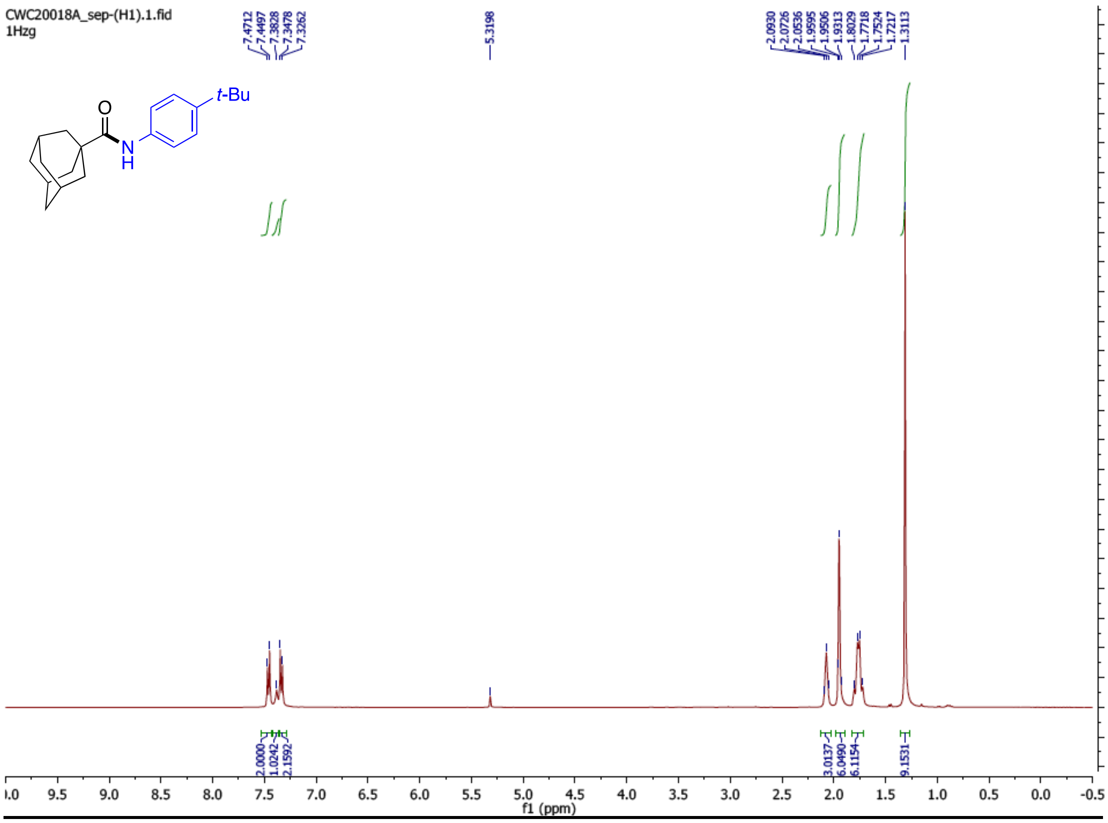

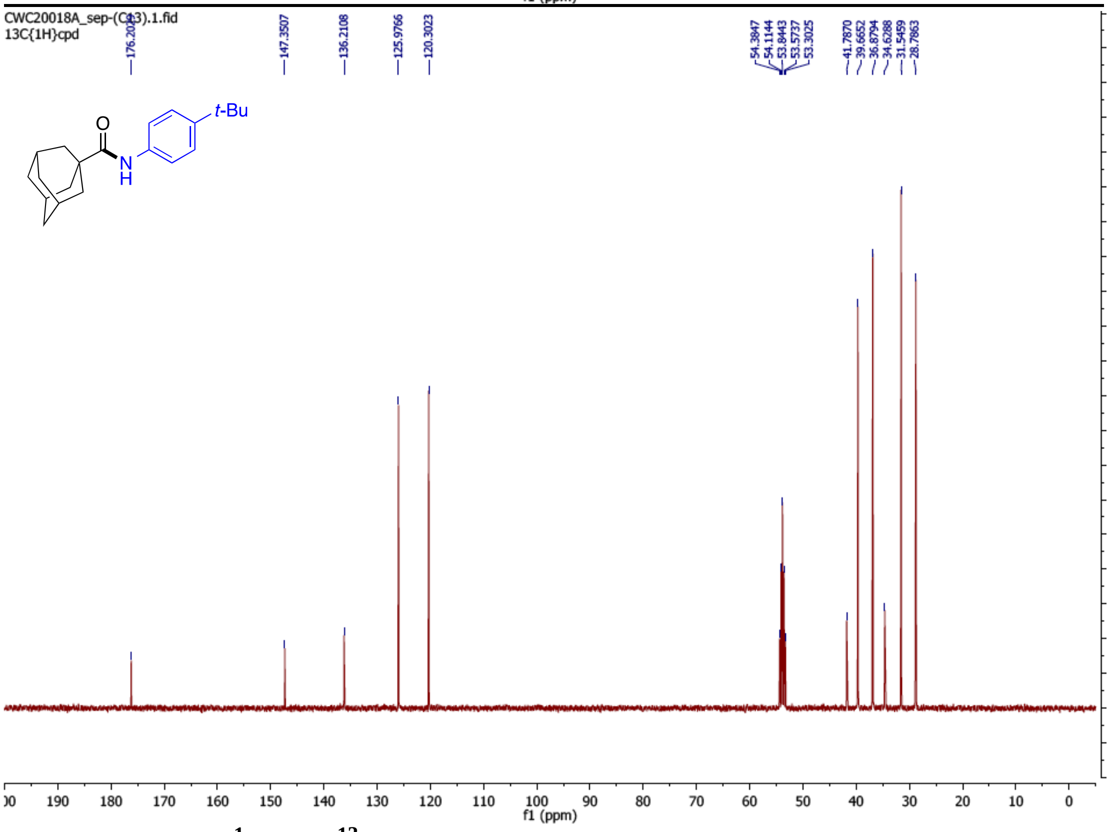

Supplementary Figure 43. ^{1}H and ^{13}C NMR spectra of *N*-(4-(*tert*-butyl)phenyl)adamantane-1-carboxamide (4h)

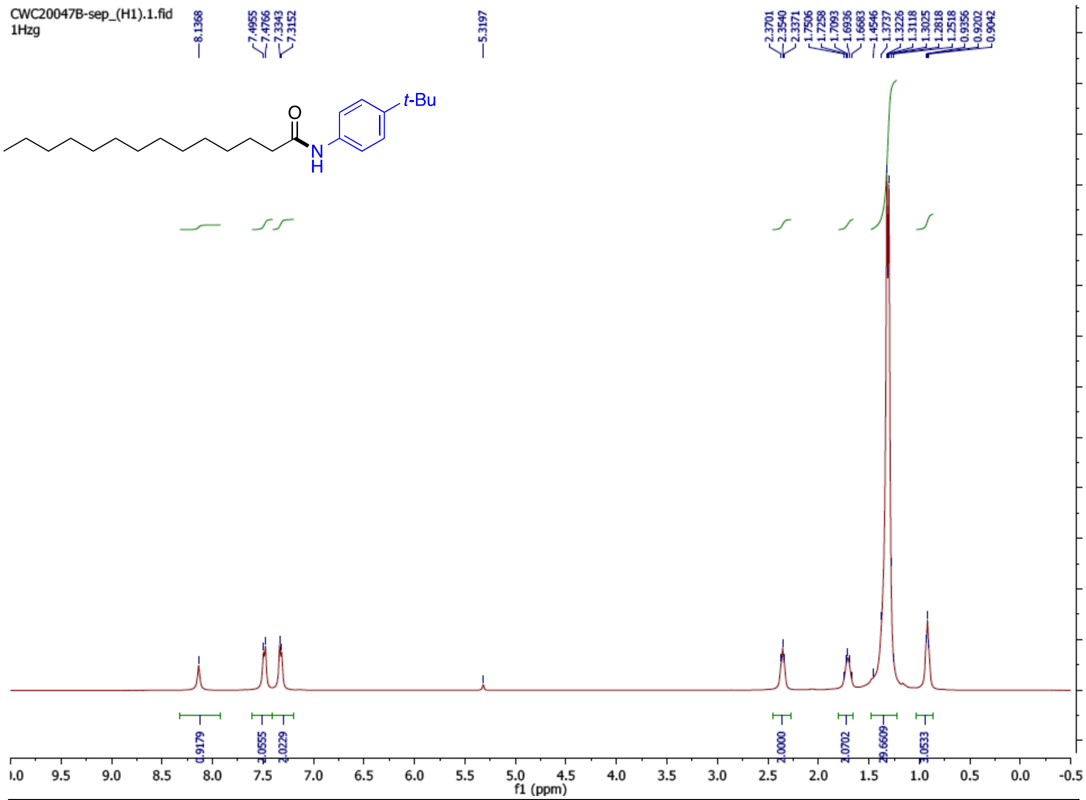

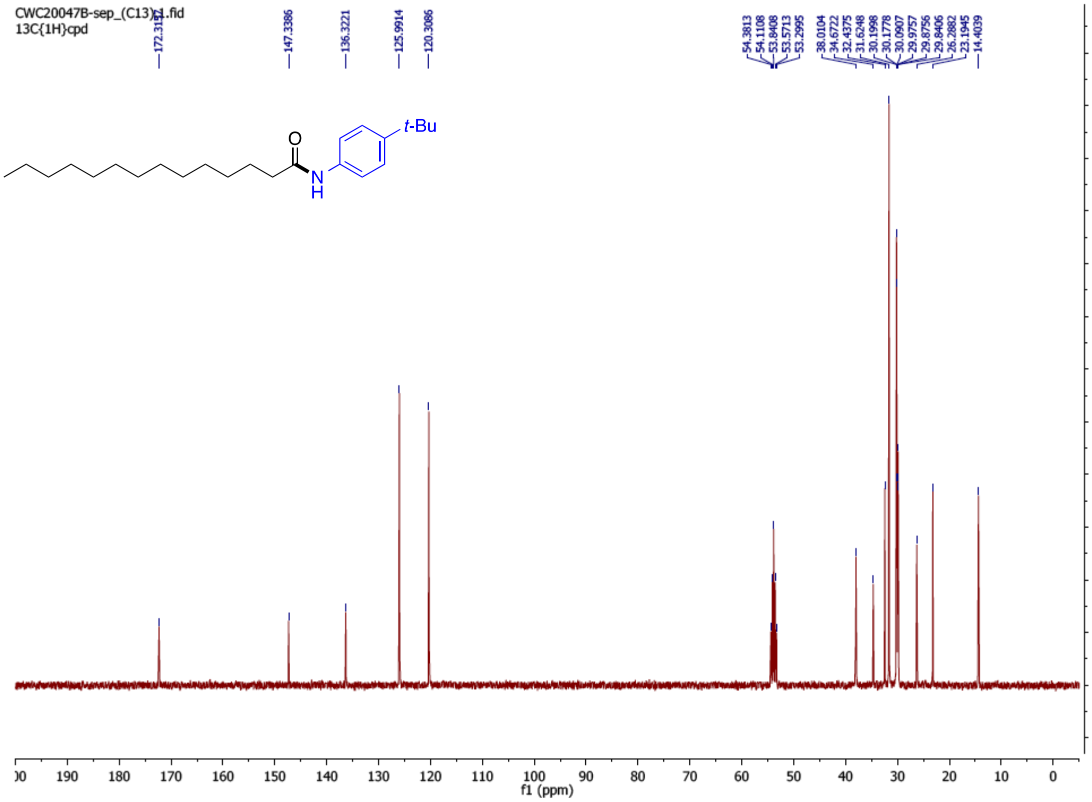

Supplementary Figure 44. ^{1}**H and** ^{13}**C NMR spectra of *N*-(4-(*tert*-butyl)phenyl)tetradecanamide (4i)**

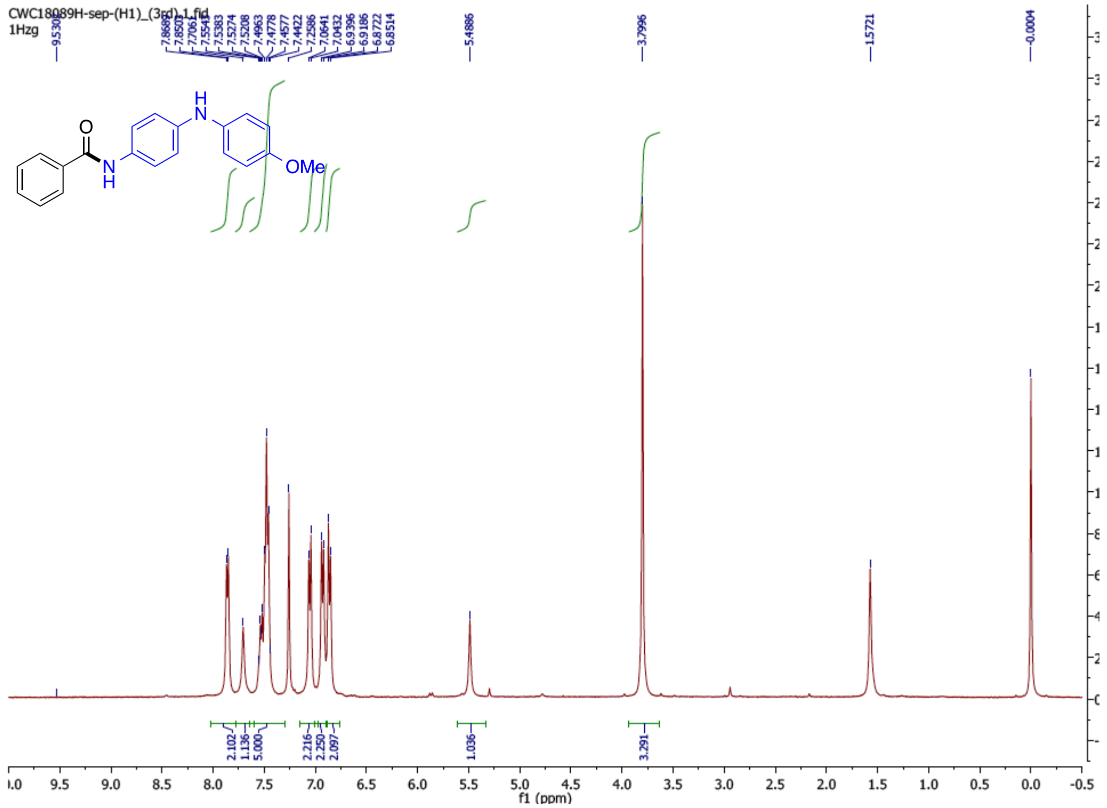

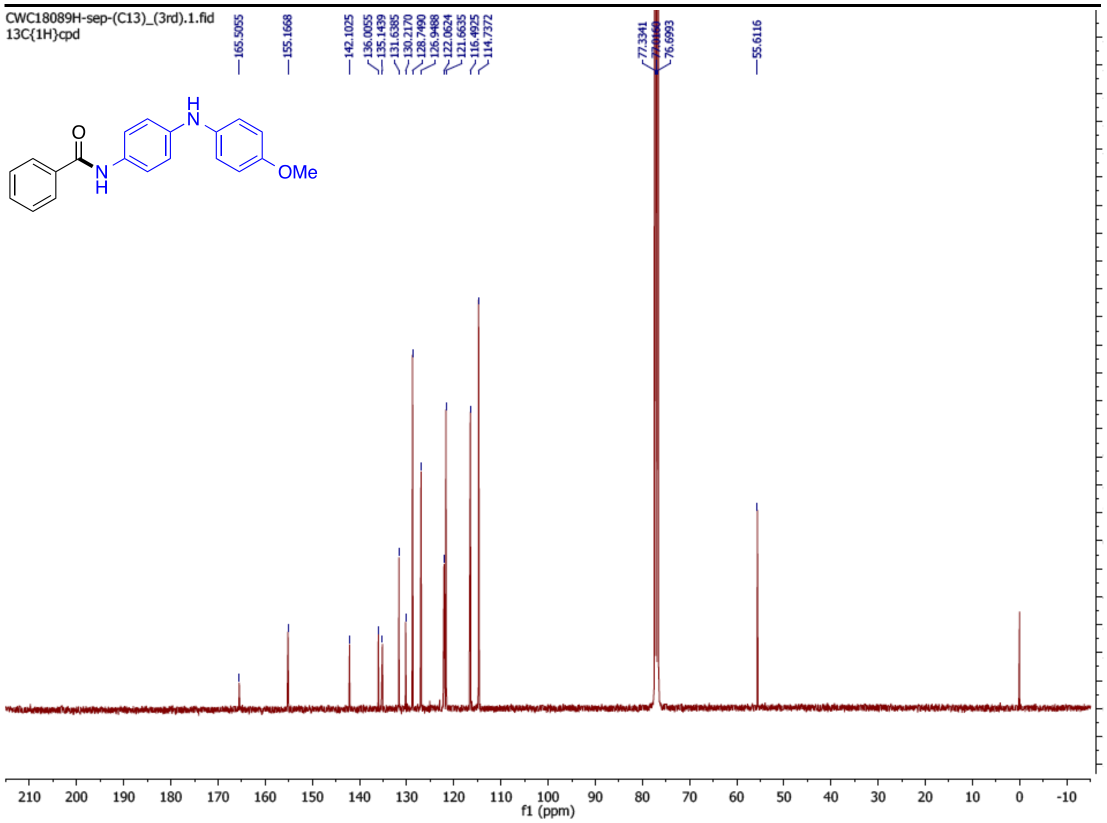

Supplementary Figure 45. ^{1}H **and** ^{13}C **NMR spectra of** ***N*****-(4-((4-Methoxyphenyl)amino)phenyl)benzamide (5a)**

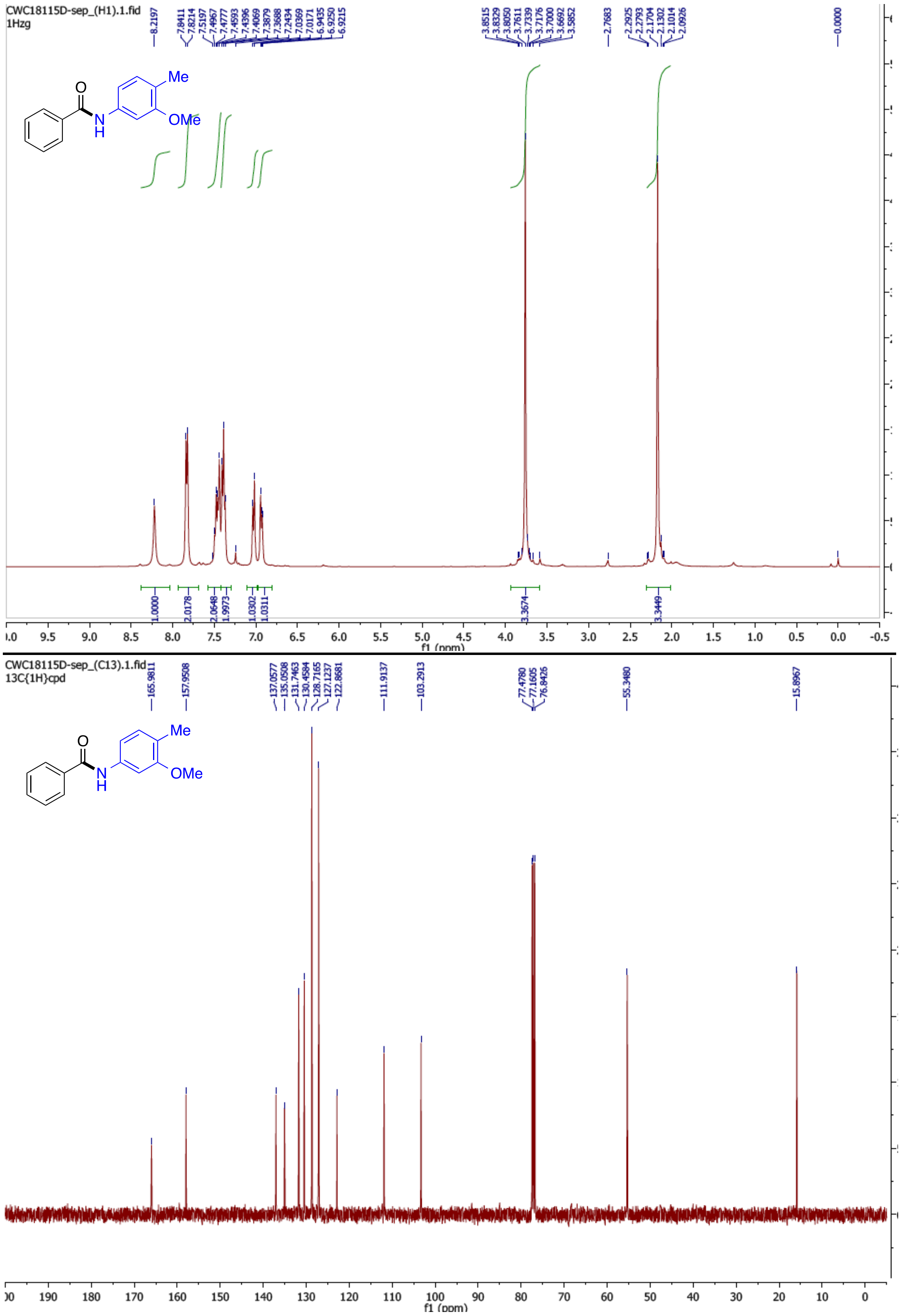

Supplementary Figure 46. ^{1}H and ^{13}C NMR spectra of *N*-(3-Methoxy-4-methylphenyl)benzamide (5b)

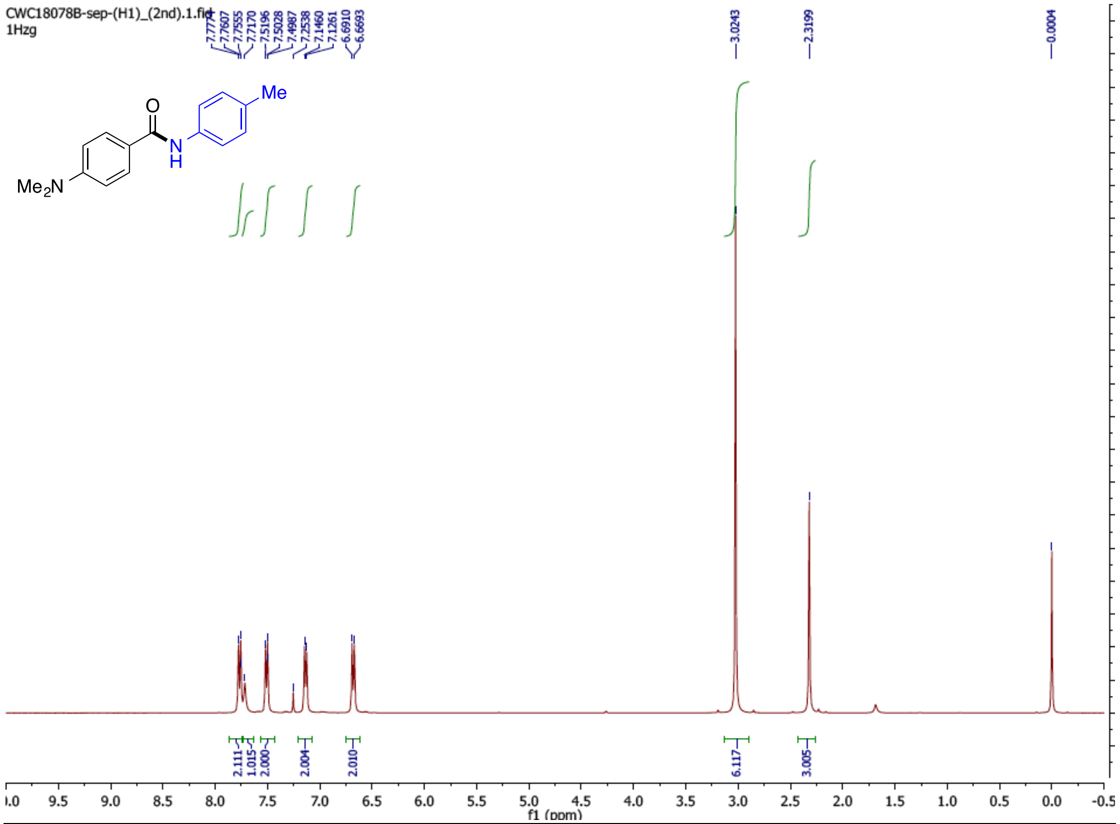

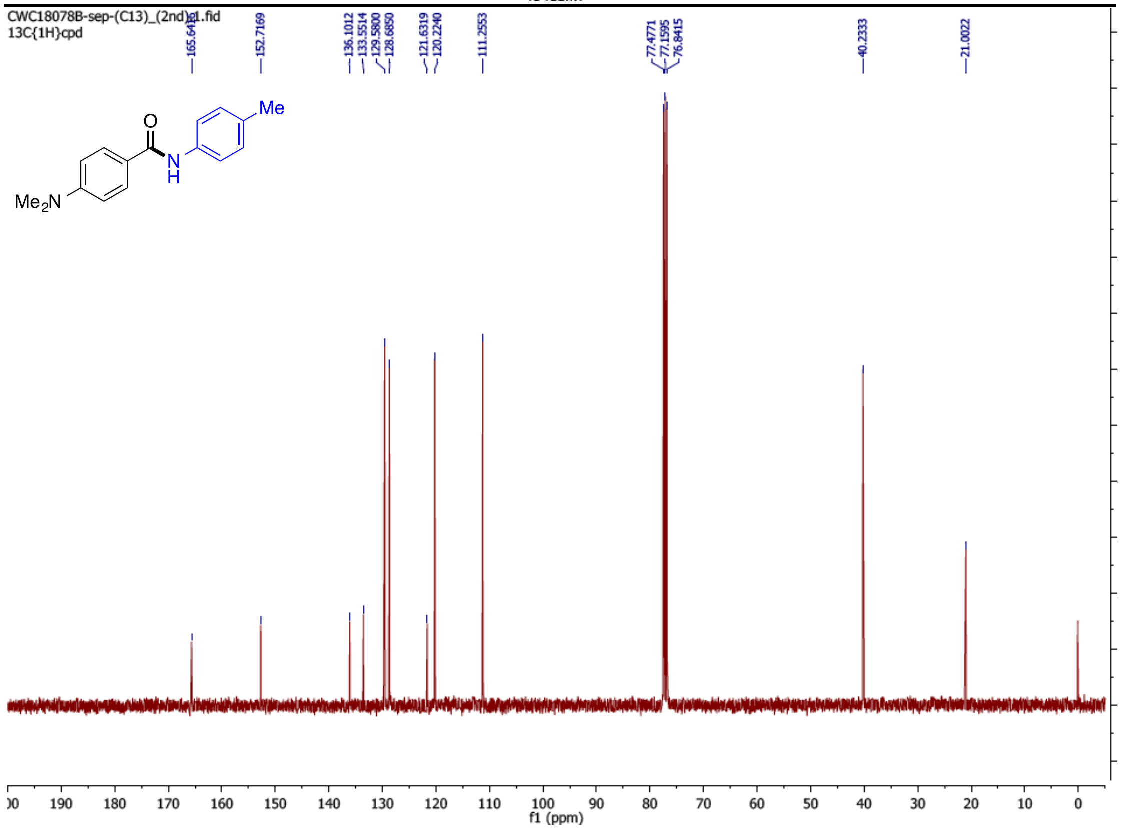

Supplementary Figure 47. ^{1}**H and** ^{13}**C NMR spectra of 4-(Dimethylamino)-*N*-(*p*-tolyl)benzamide (5c)**

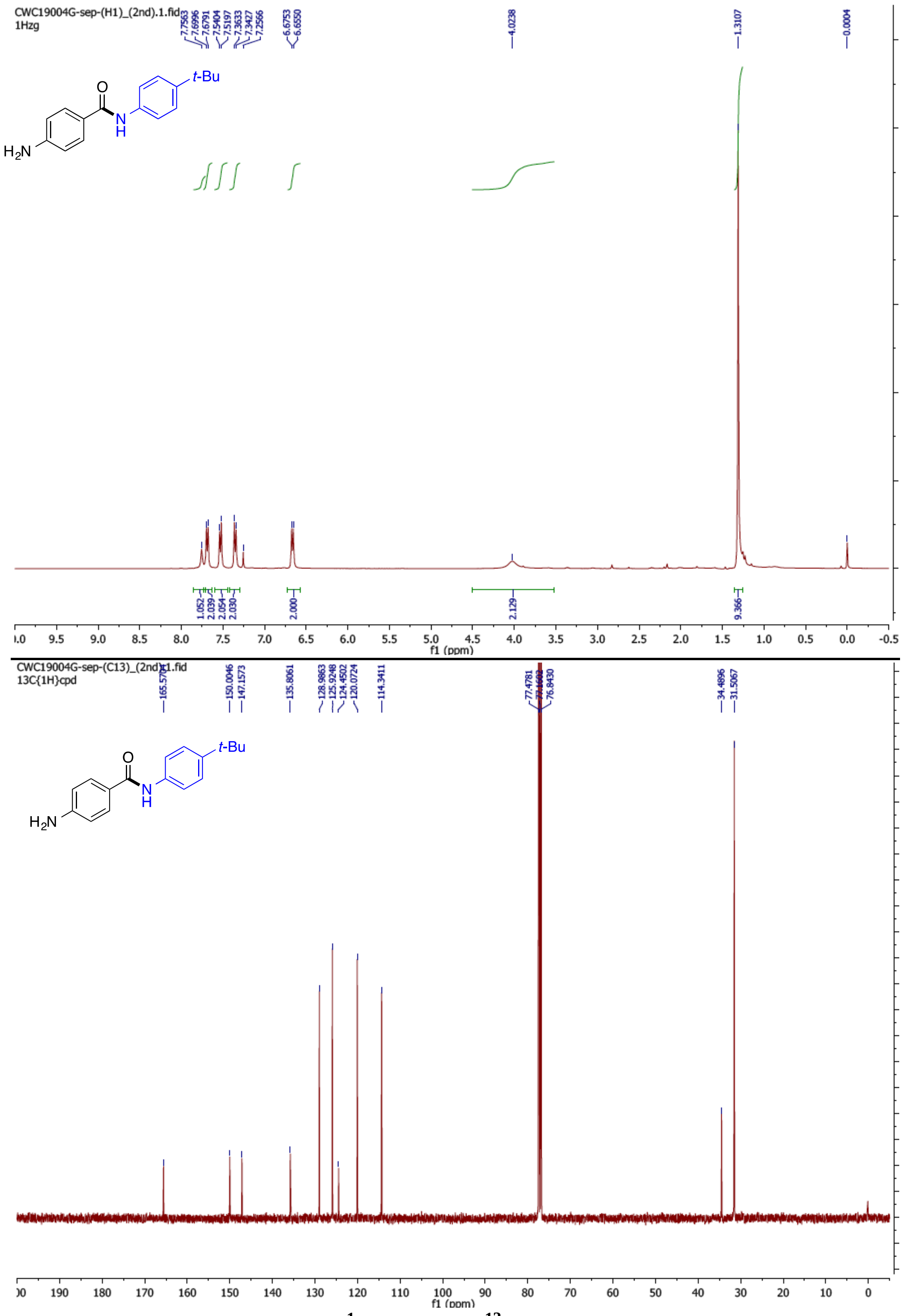

Supplementary Figure 48. **^{1}H and ^{13}C NMR spectra of 4-Amino-*N*-(4-(*tert*-butyl)phenyl)benzamide (5d)**

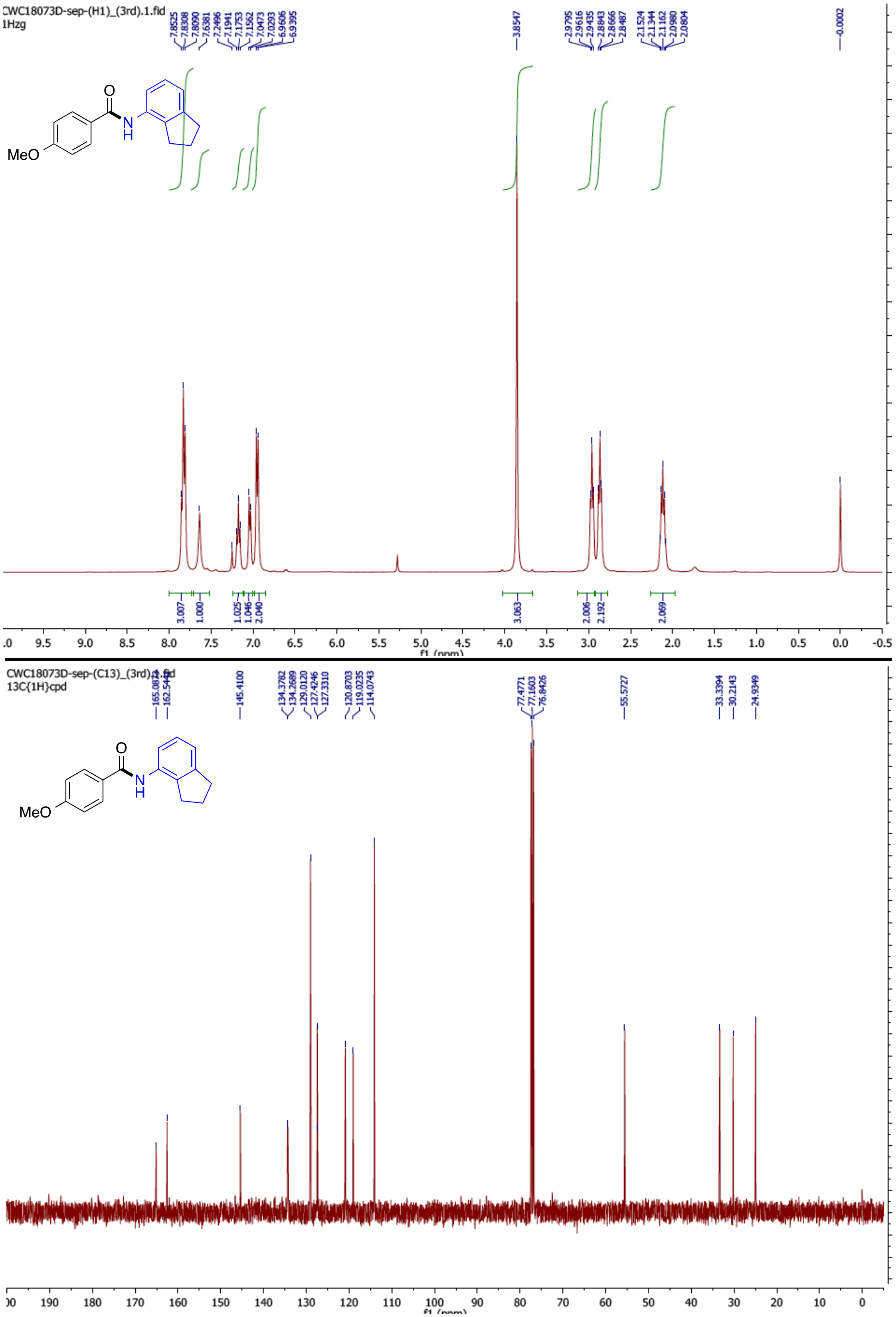

Supplementary Figure 49. ^{1}**H and** ^{13}**C NMR spectra of** ***N*****-(2,3-Dihydro-1*****H*****-inden-4-yl)-4-methoxybenzamide (5e)**

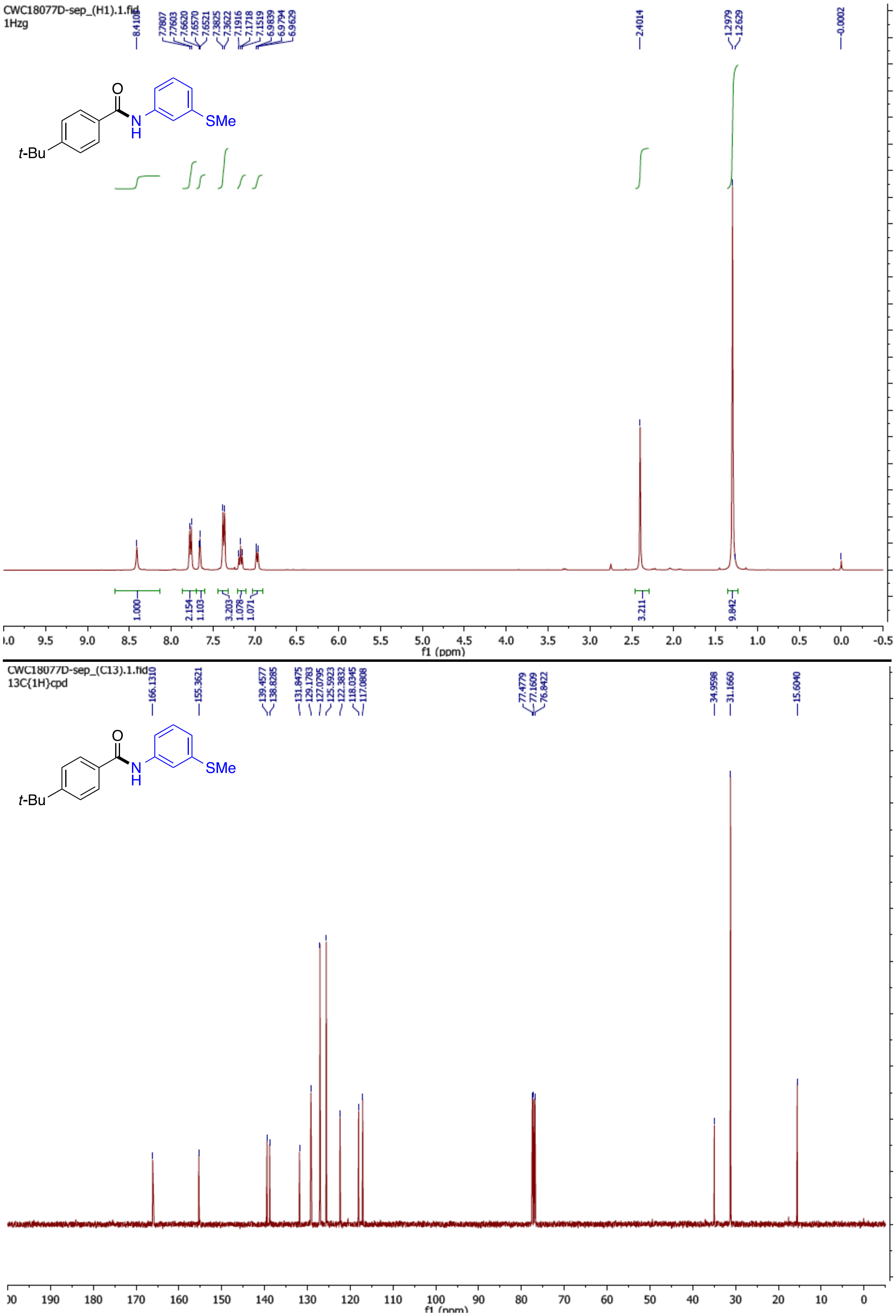

Supplementary Figure 50. ^{1}**H and** ^{13}**C NMR spectra of 4-(*tert*-Butyl)-*N*-(3-(methylthio)phenyl)benzamide (5f)**

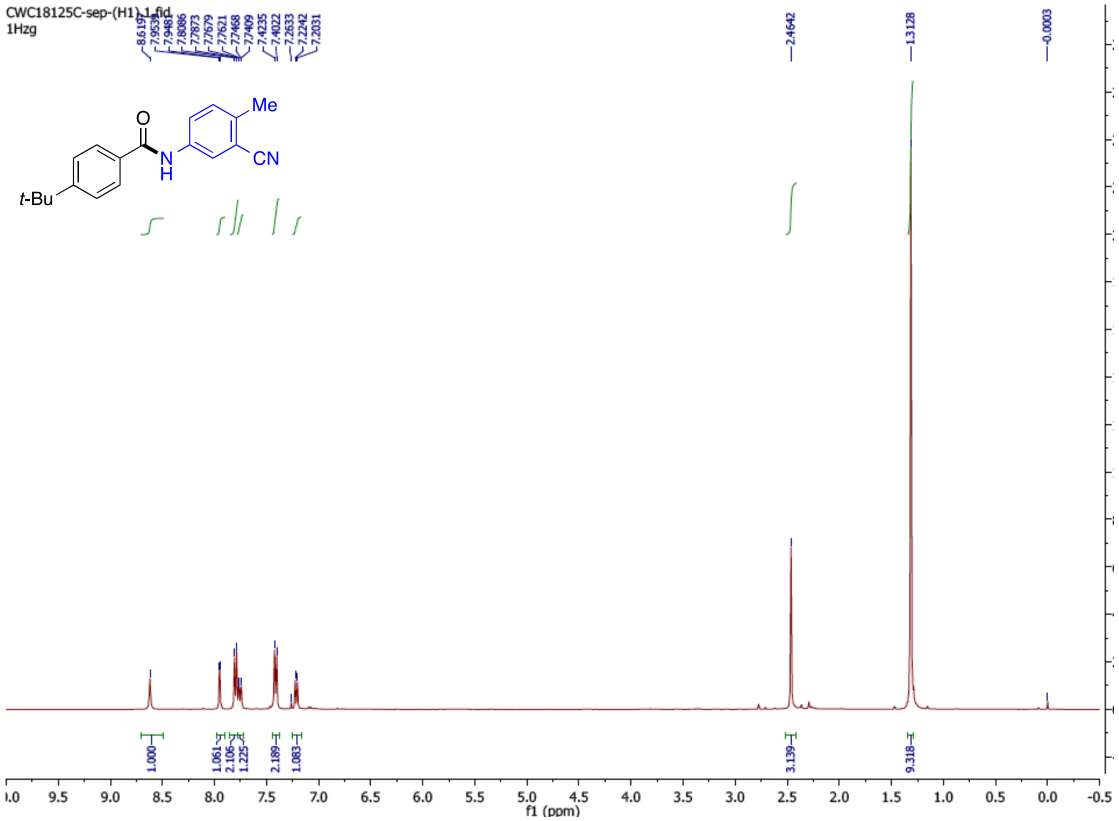

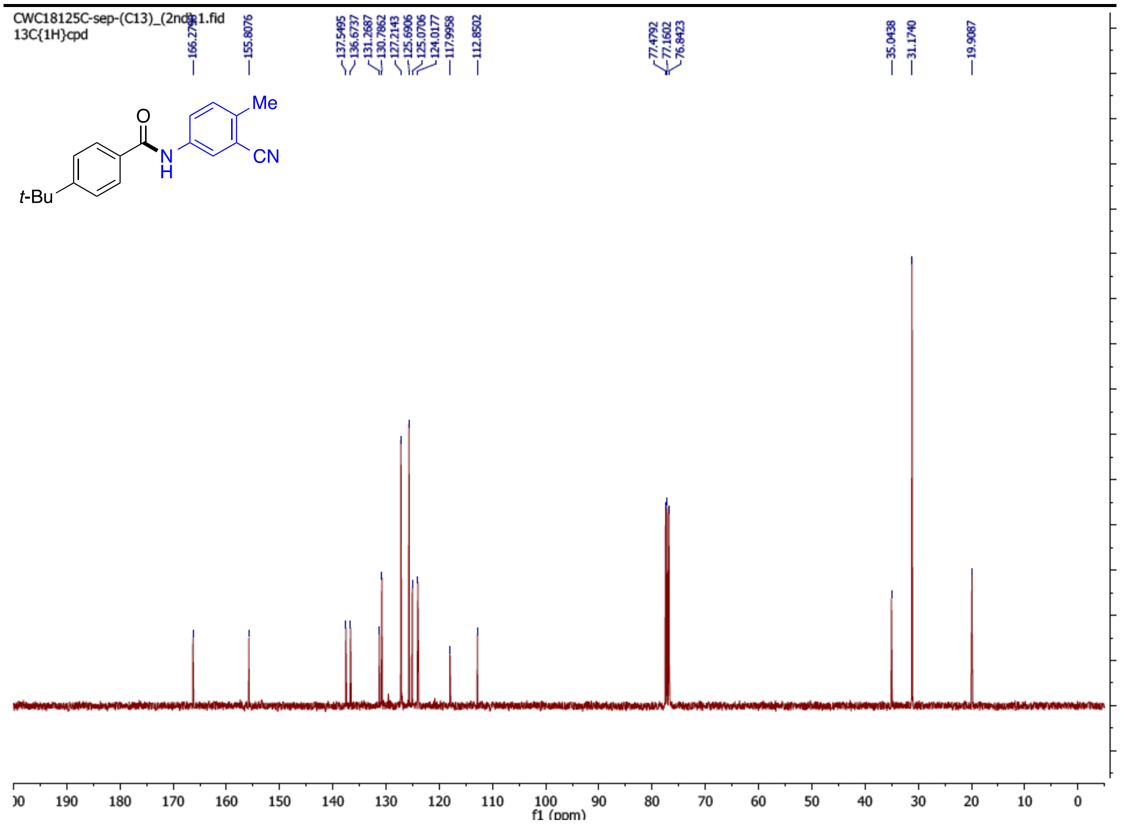

Supplementary Figure 51. **^{1}H and ^{13}C NMR spectra of 4-(*tert*-Butyl)-*N*-(3-cyano-4-methylphenyl)benzamide (5g)**

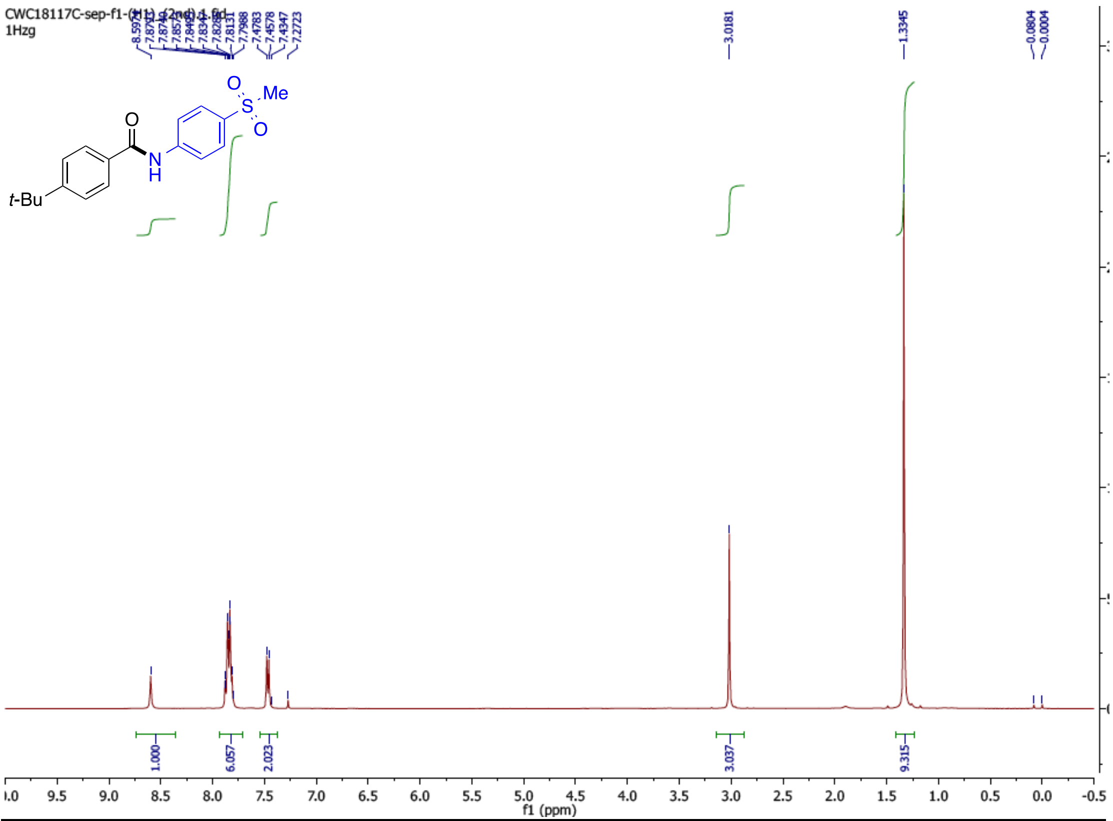

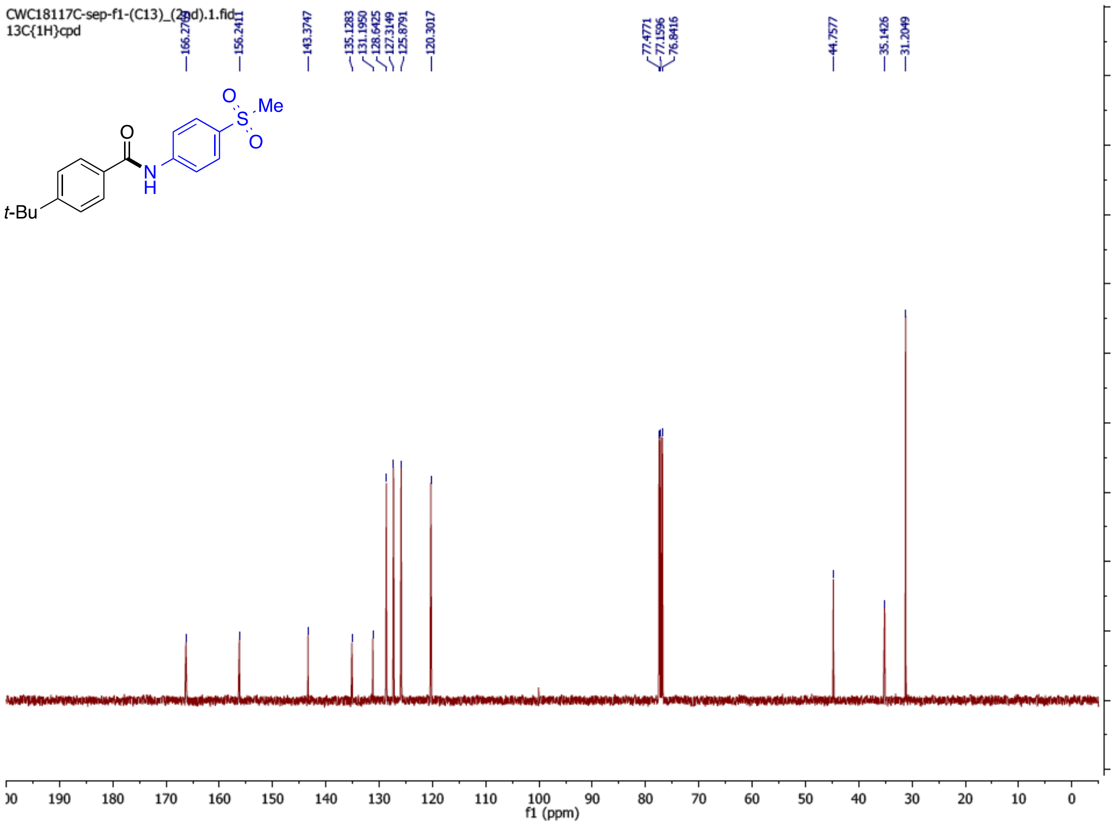

Supplementary Figure 52. ^{1}H and ^{13}C NMR spectra of 4-(*tert*-Butyl)-*N*-(4-(methylsulfonyl)phenyl)benzamide (5h)

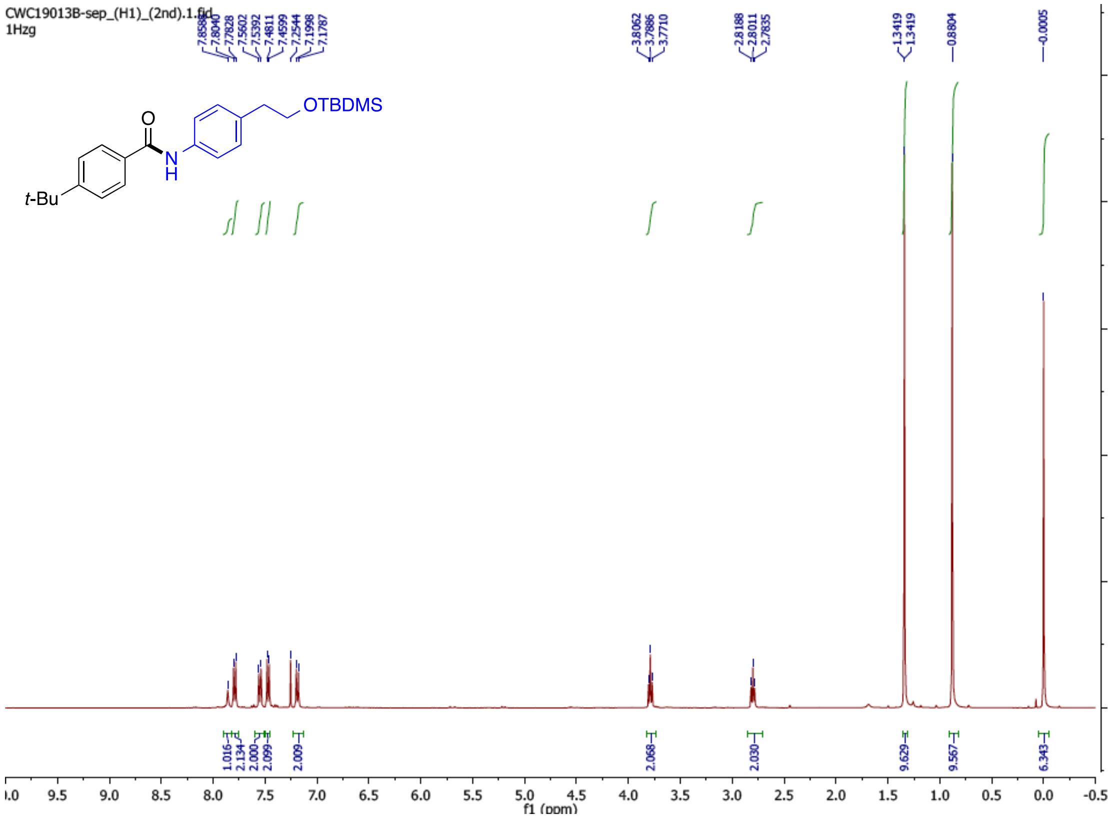

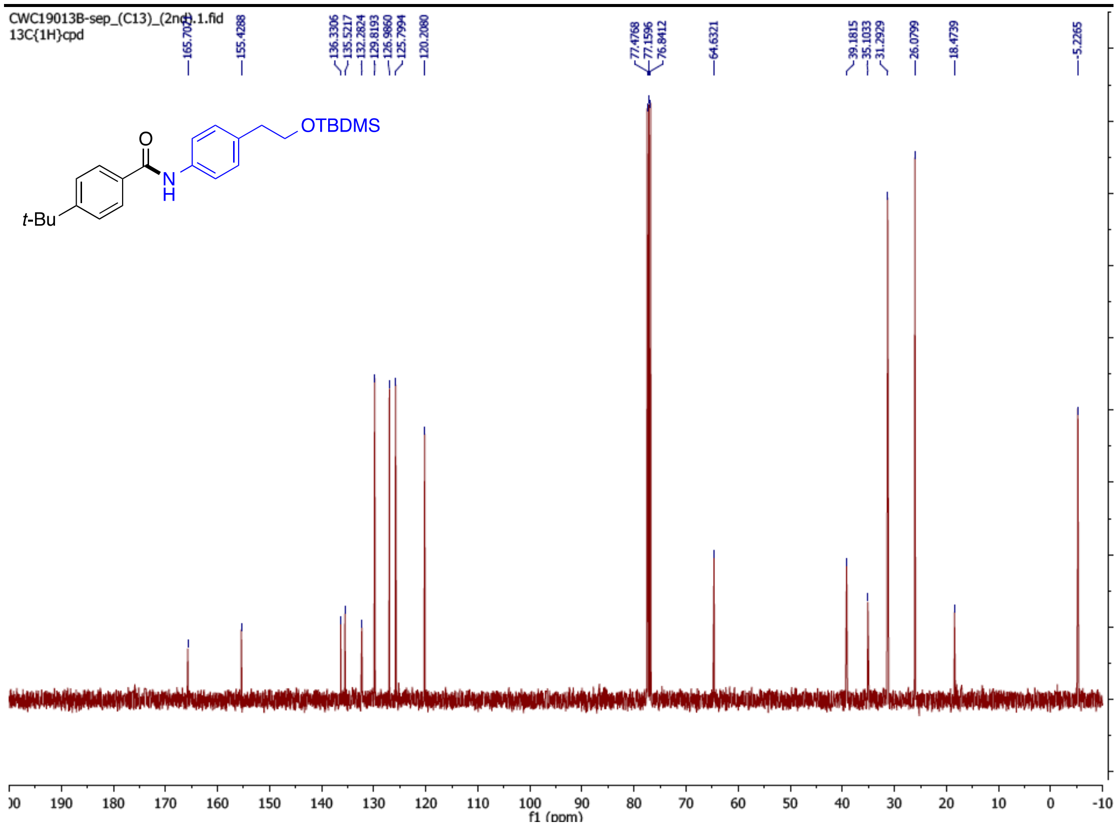

Supplementary Figure 53. ^{1}H and ^{13}C NMR spectra of 4-(*tert*-Butyl)-*N*-(4-(2-((*tert*-butyldimethylsilyl)oxy)ethyl)phenyl)benzamide (5i)

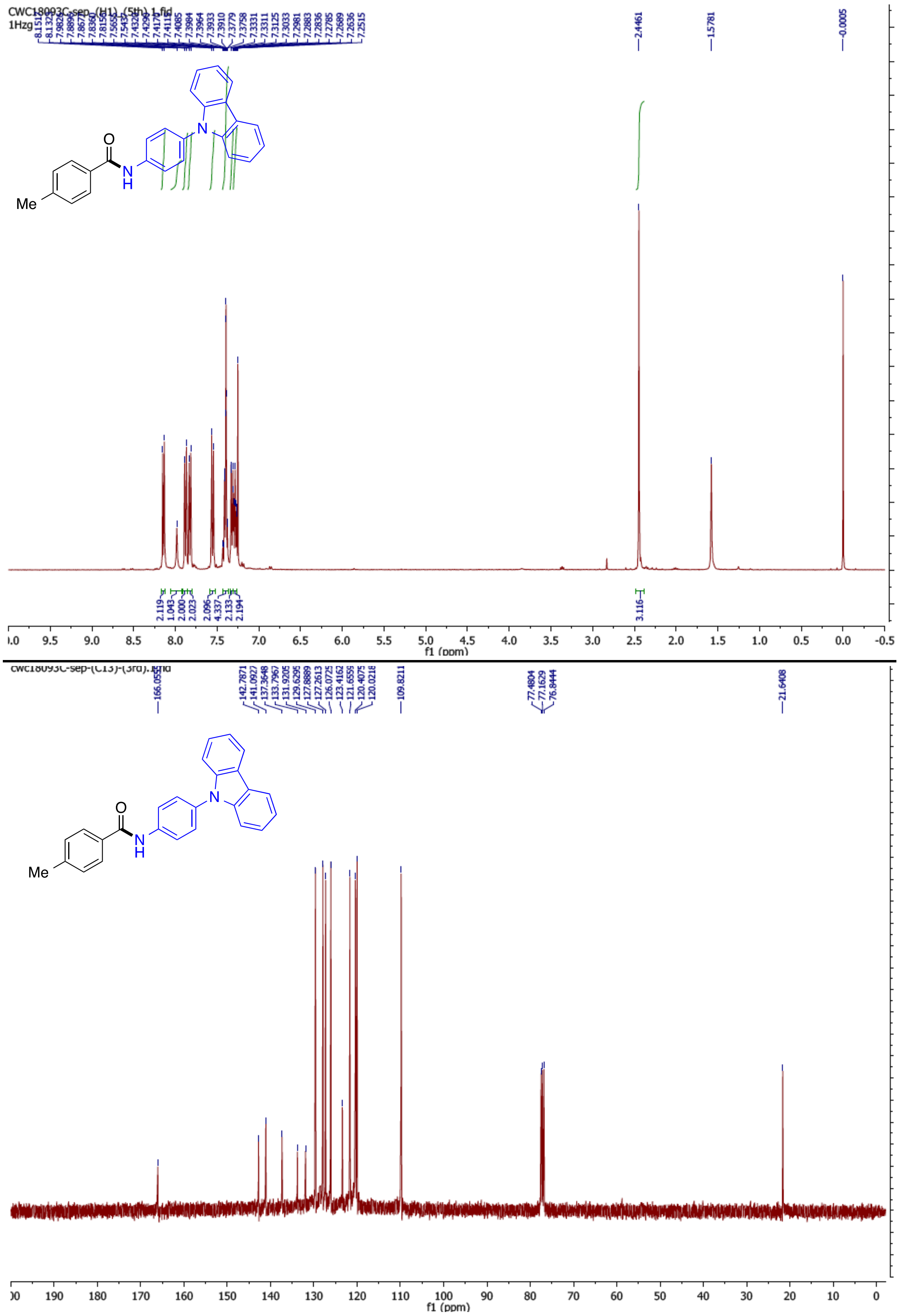

Supplementary Figure 54. ^{1}H and ^{13}C NMR spectra of *N*-(4-(9*H*-Carbazol-9-yl)phenyl)-4-methylbenzamide (5j)

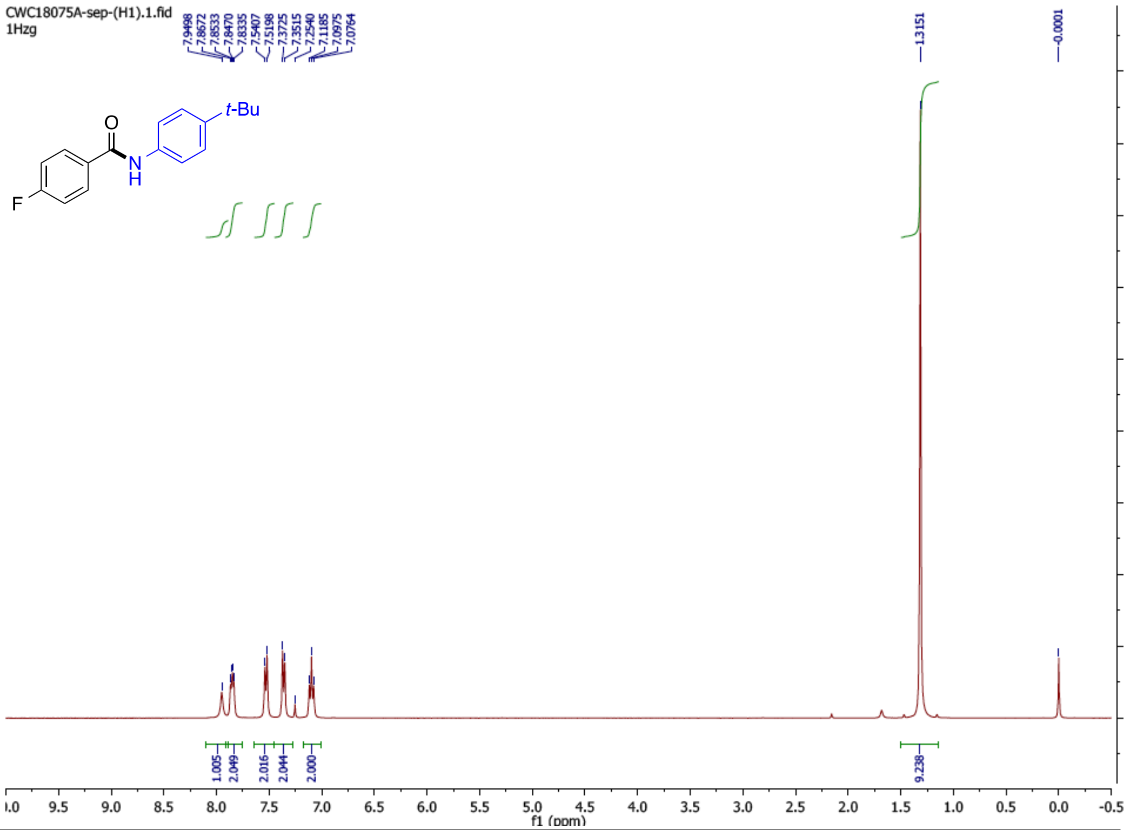

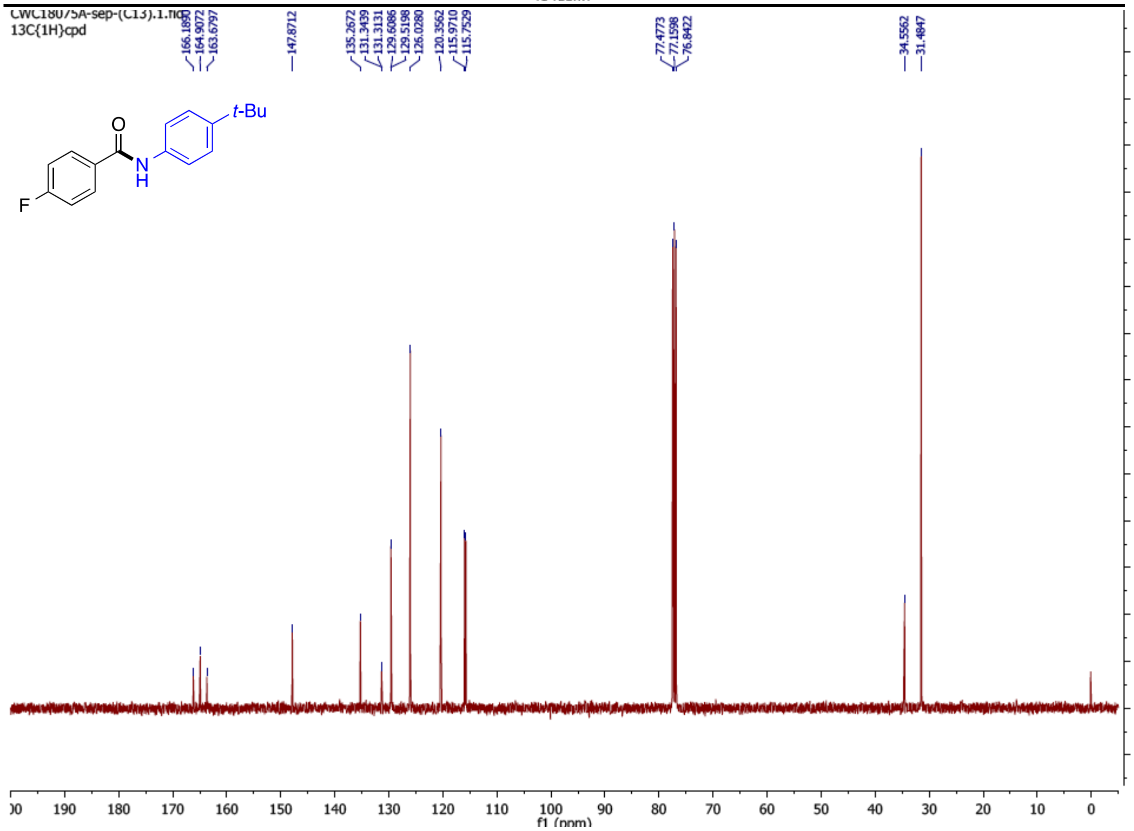

Supplementary Figure 55. ^{1}H **and** ^{13}C **NMR spectra of *N*-(4-(*tert*-Butyl)phenyl)-4-fluorobenzamide (5k)**

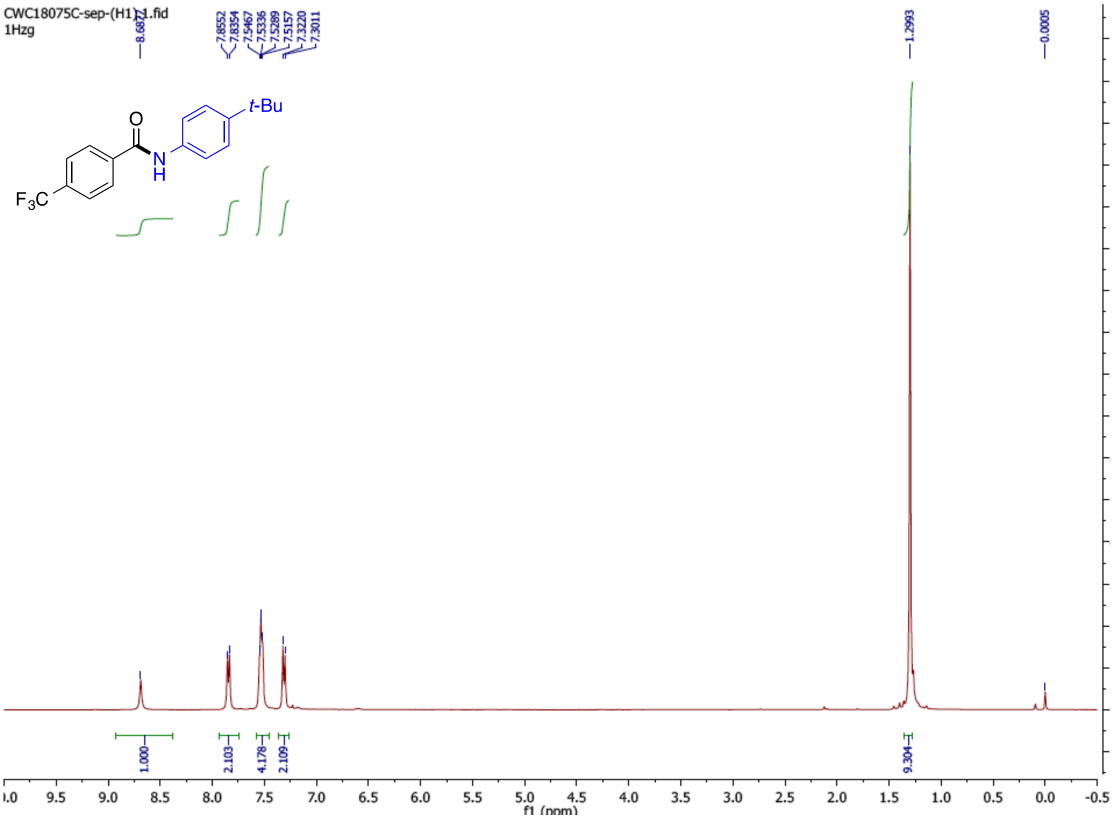

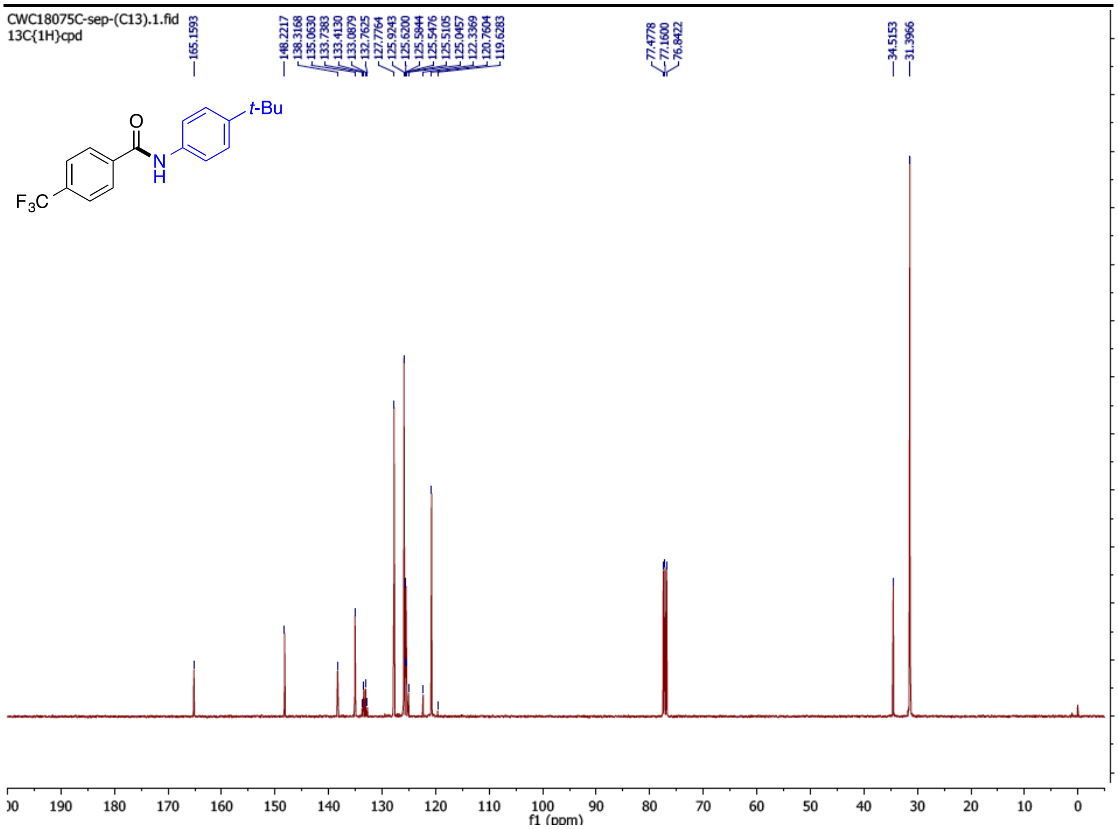

Supplementary Figure 56. ^{1}H **and** ^{13}C **NMR spectra of** ***N*-(4-(*tert*-Butyl)phenyl)-4-(trifluoromethyl)benzamide (5l)**

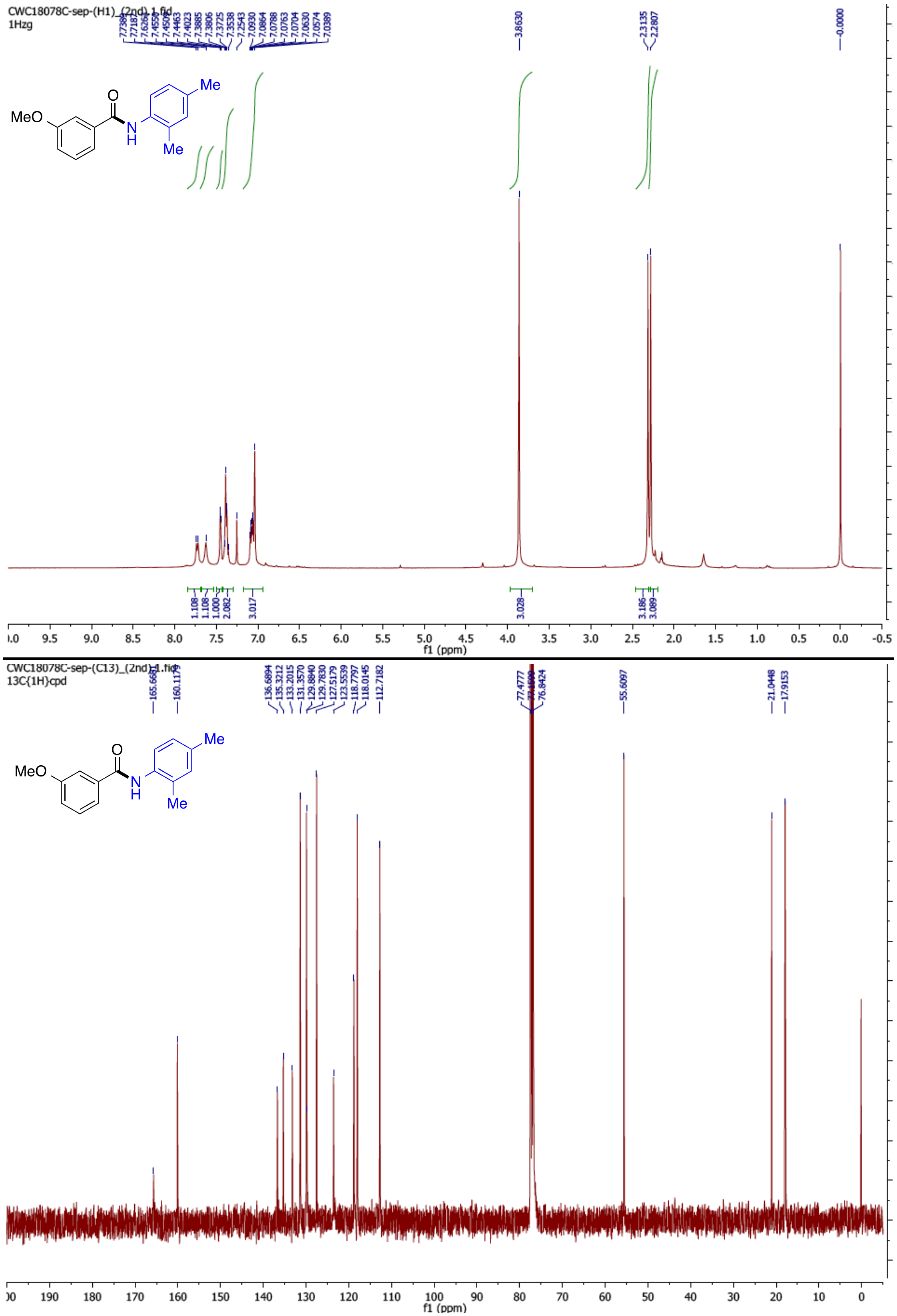

Supplementary Figure 57. ^{1}**H and** ^{13}**C NMR spectra of** ***N*-(2,4-Dimethylphenyl)-3-methoxybenzamide (5m)**

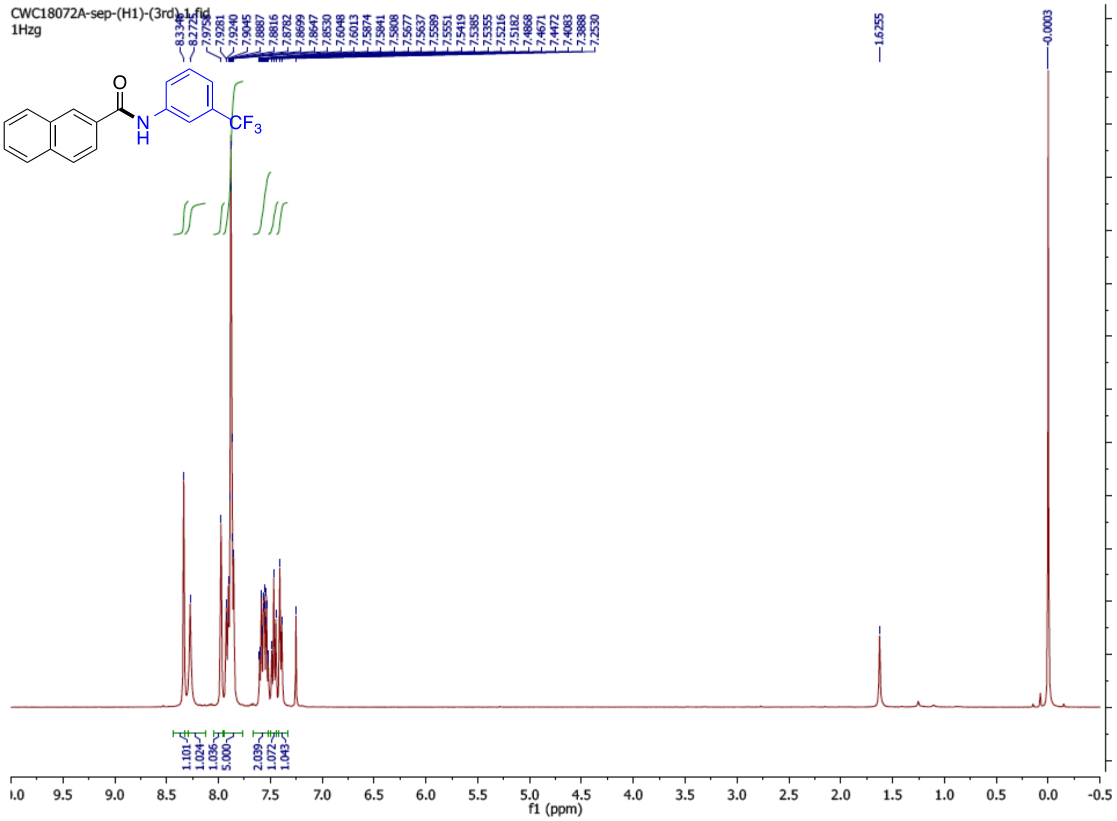

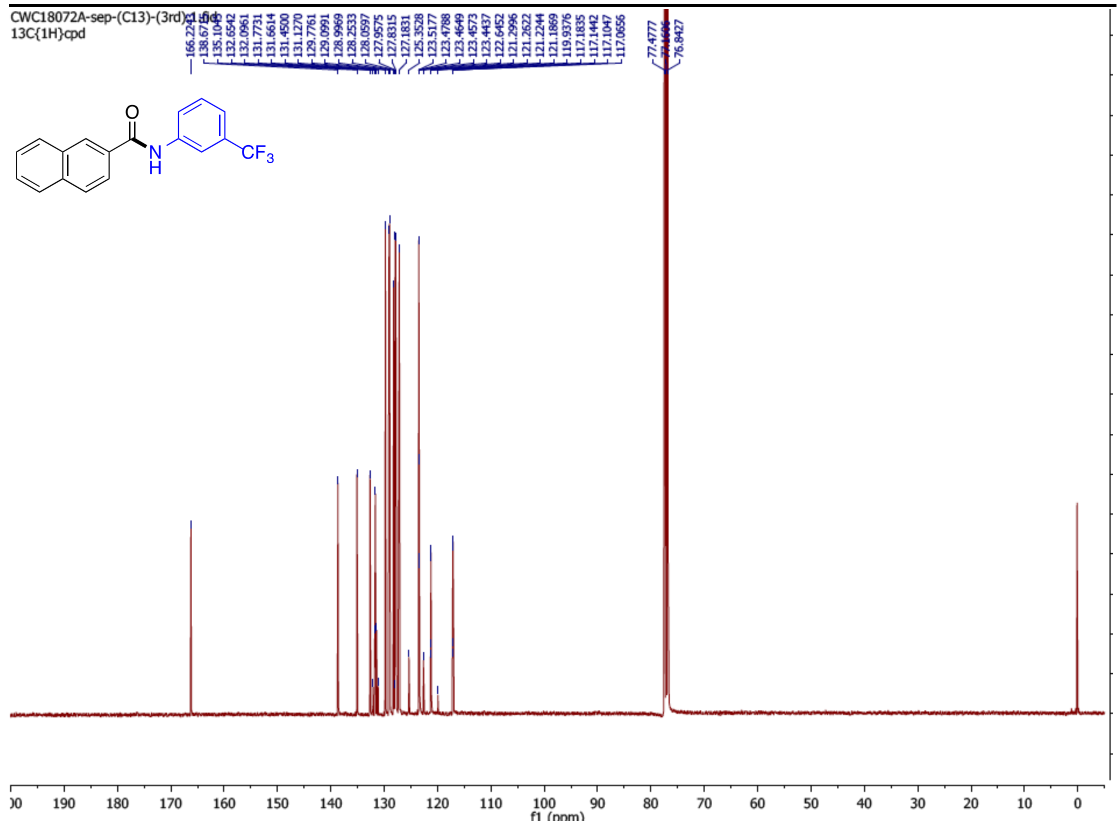

Supplementary Figure 58. ^{1}H and ^{13}C NMR spectra of *N*-(3-(Trifluoromethyl)phenyl)-2-naphthamide (5n)

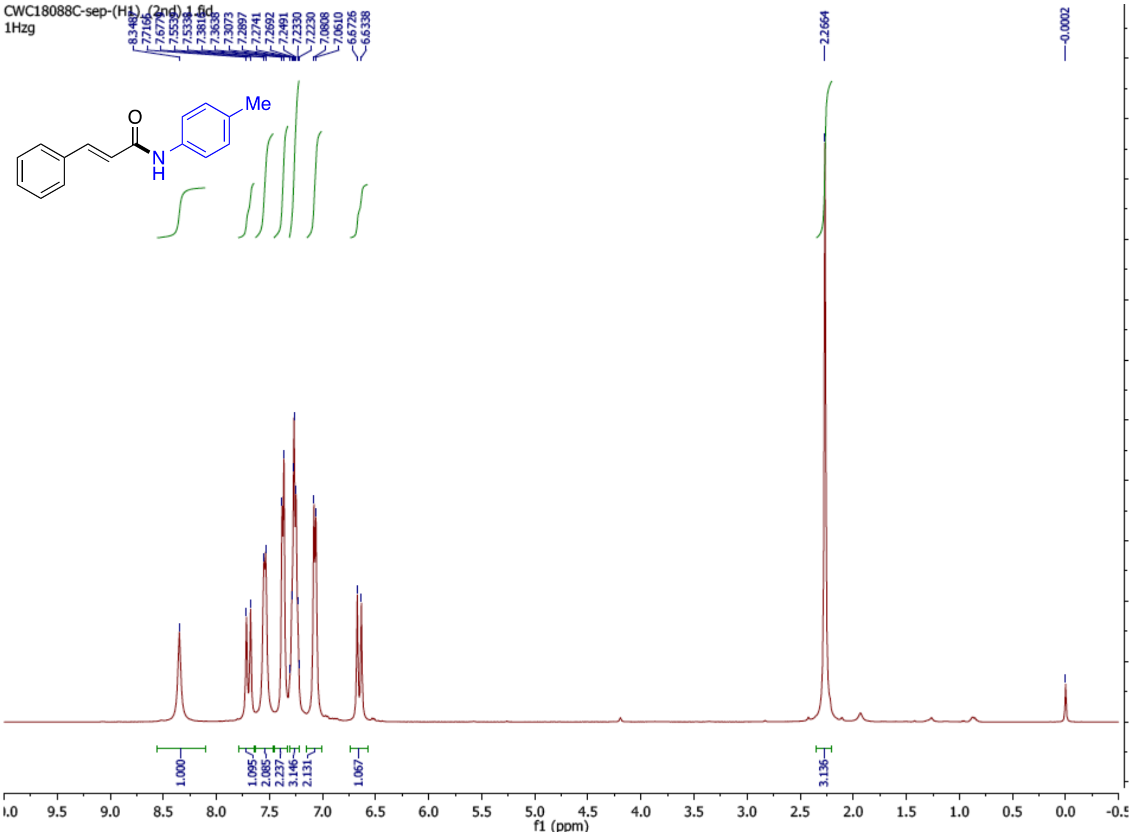

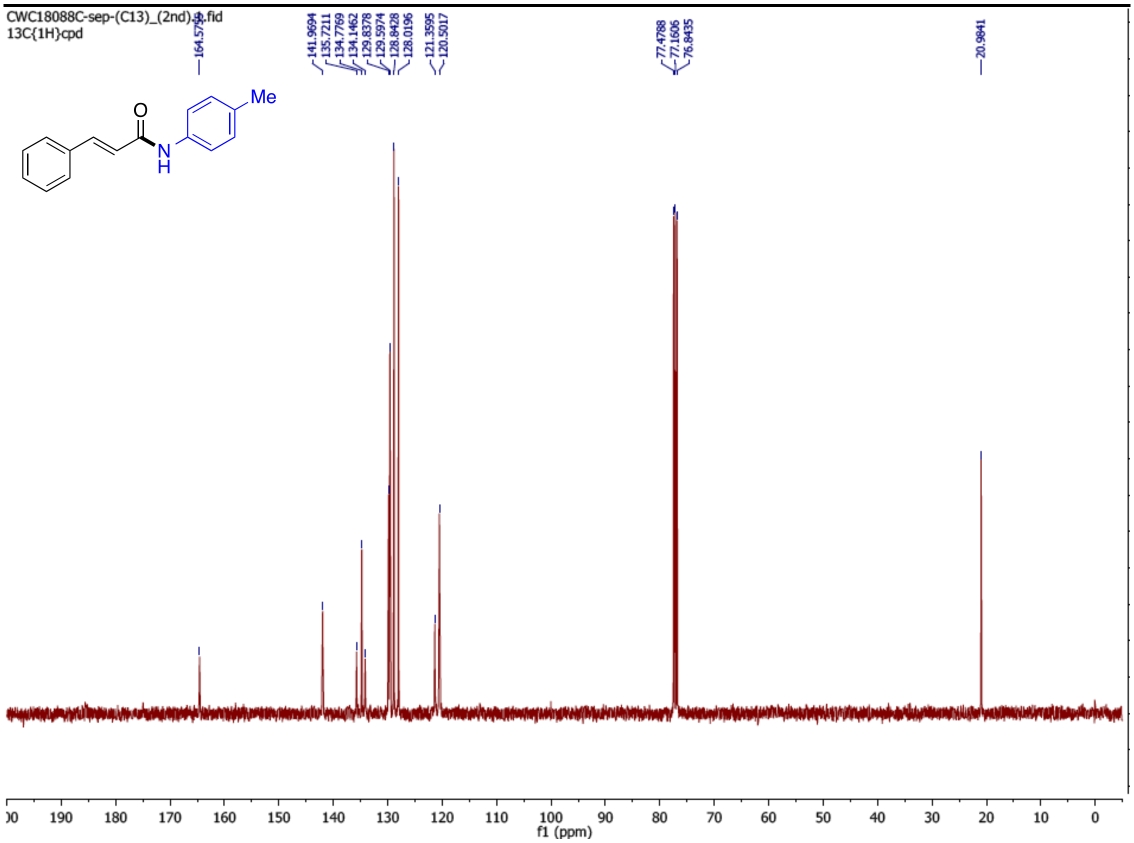

Supplementary Figure 59. ^{1}H and ^{13}C NMR spectra of *N*-(*p*-Tolyl)cinnamamide (5o)

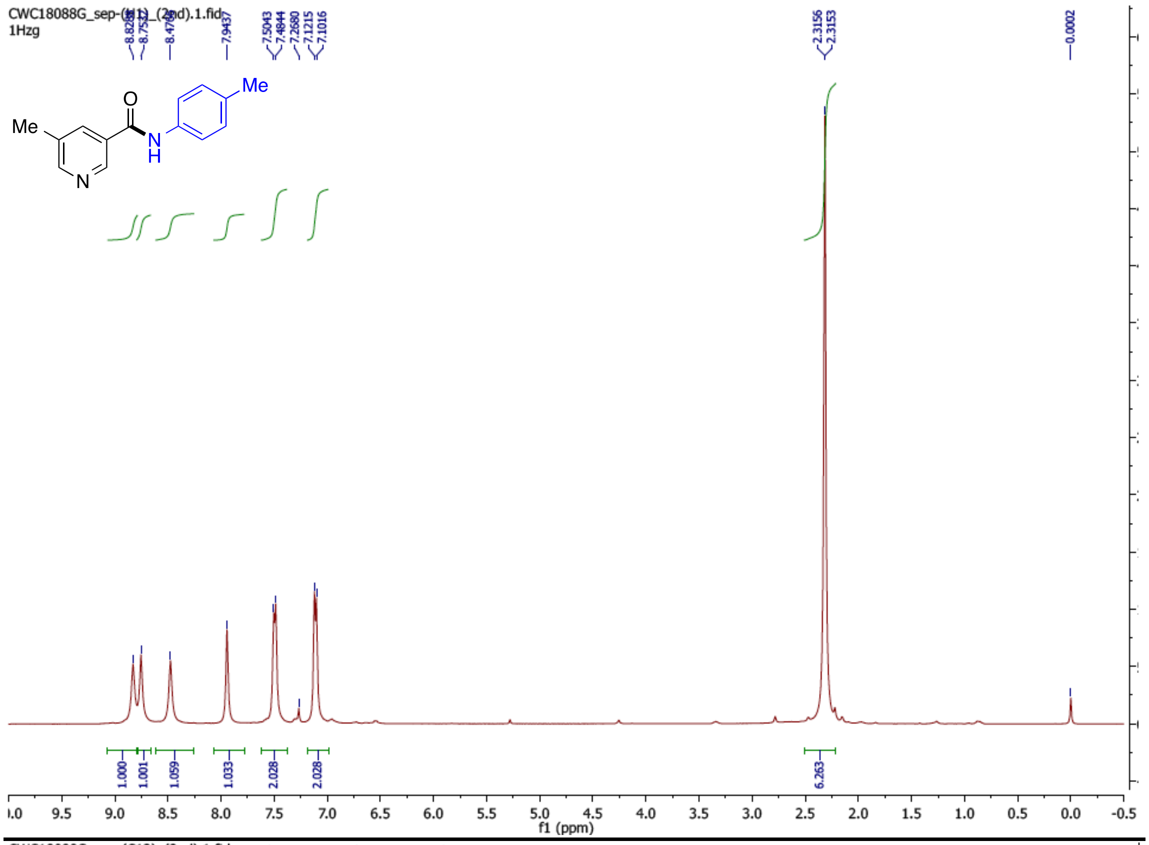

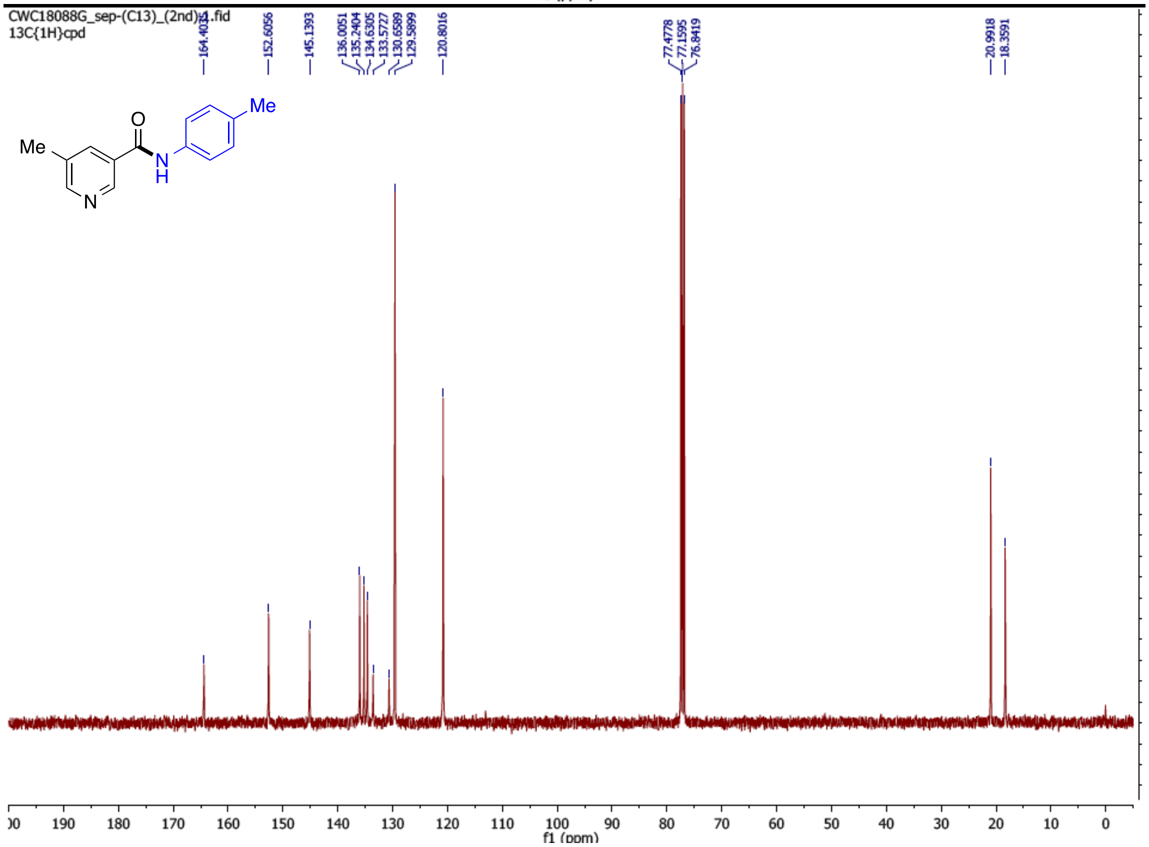

Supplementary Figure 60. ^{1}H and ^{13}C NMR spectra of 5-Methyl-*N*-(*p*-tolyl)nicotinamide (5p)

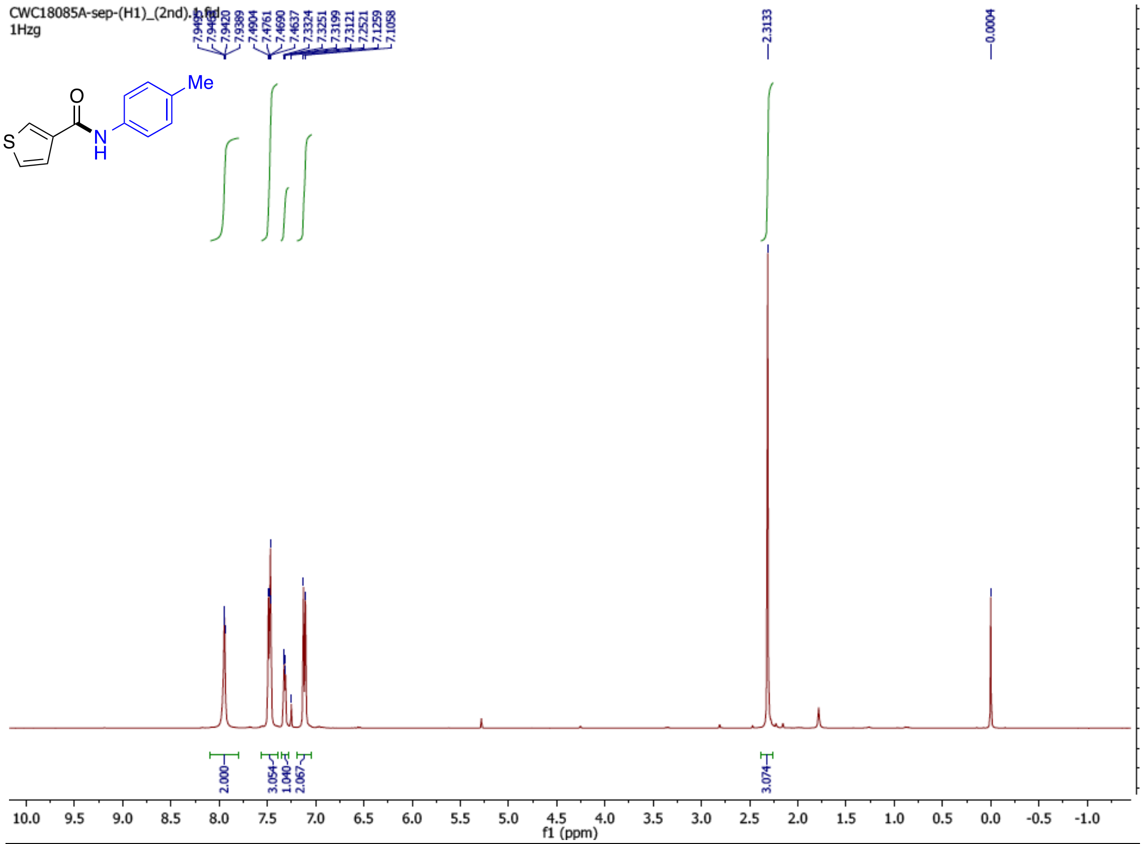

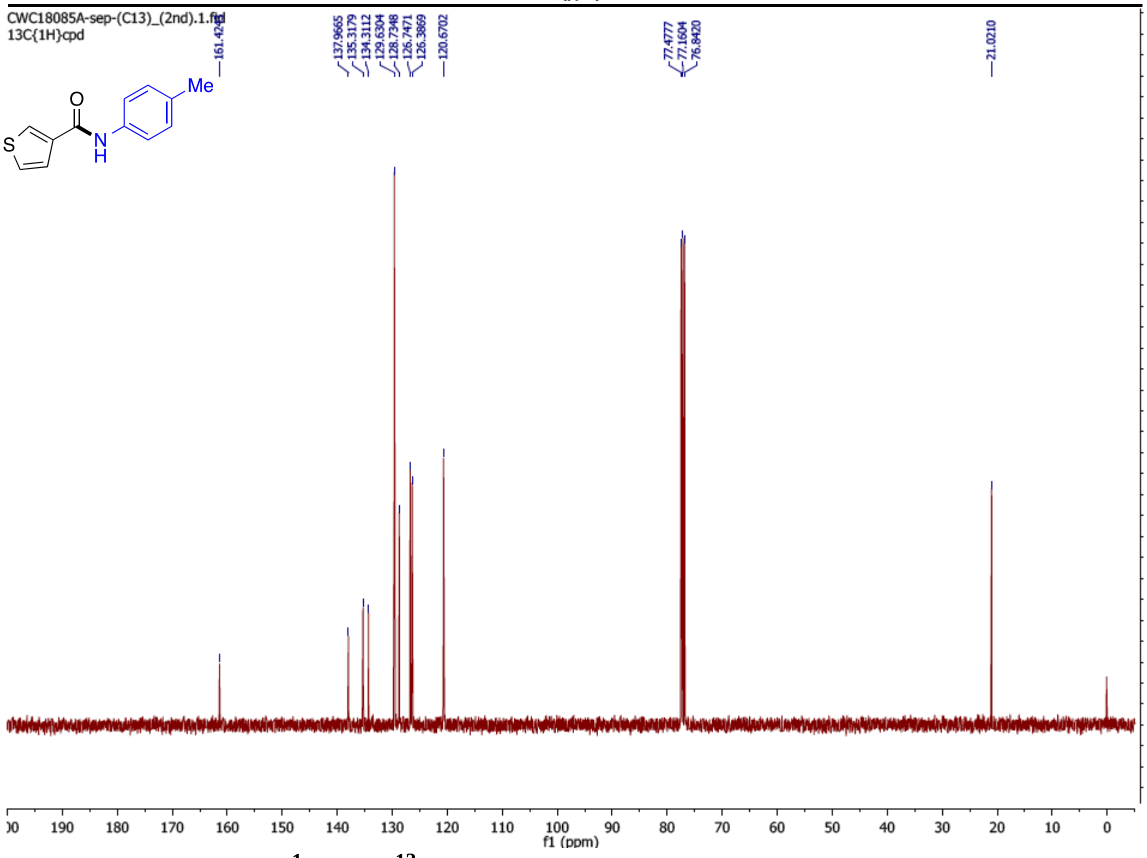

Supplementary Figure 61. ^{1}**H and** ^{13}**C NMR spectra of *N*-(*p*-Tolyl)thiophene-3-carboxamide (5q)**

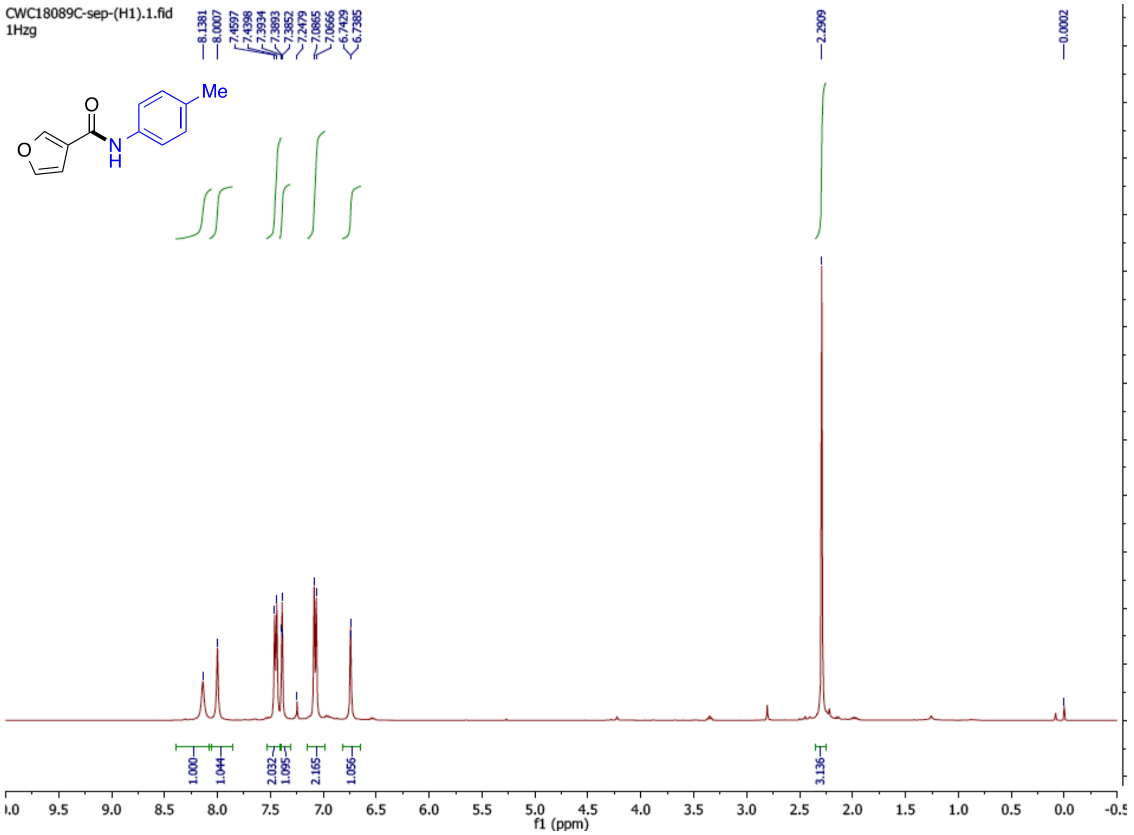

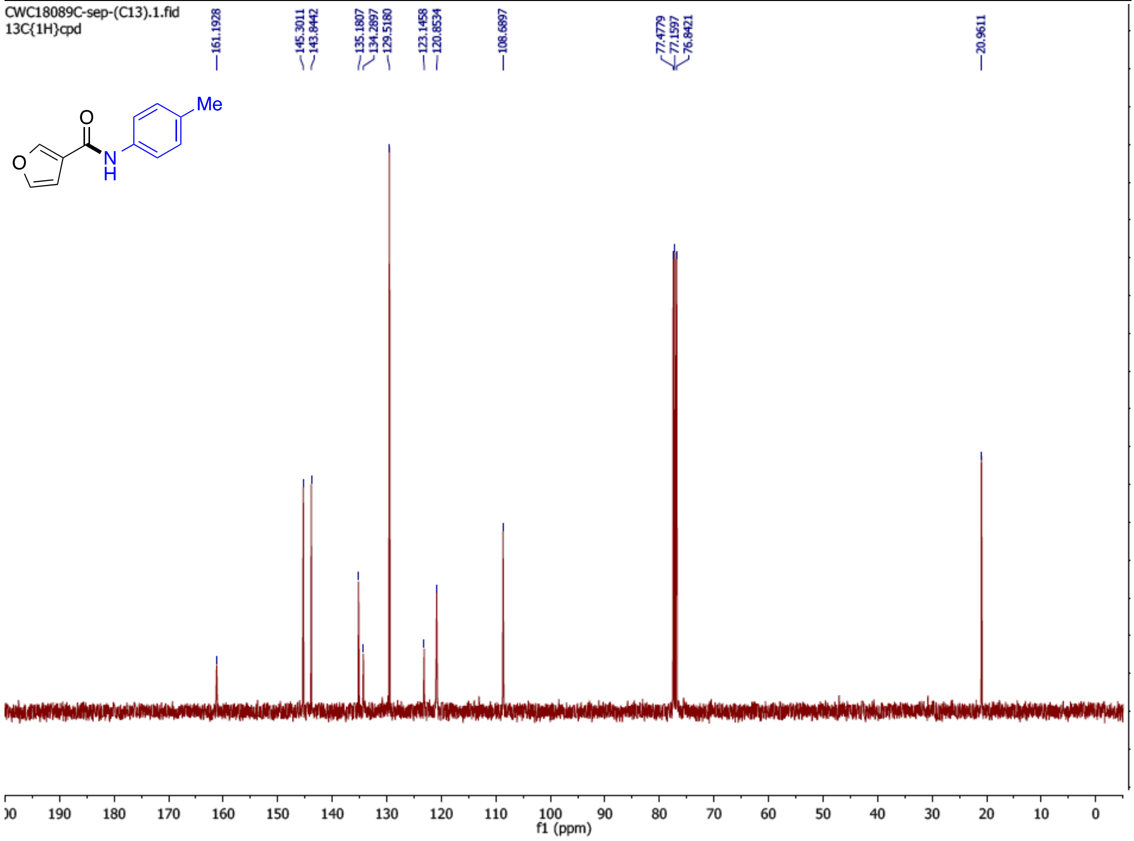

Supplementary Figure 62. ^{1}H and ^{13}C NMR spectra of *N*-(*p*-Tolyl)furan-3-carboxamide (5r)

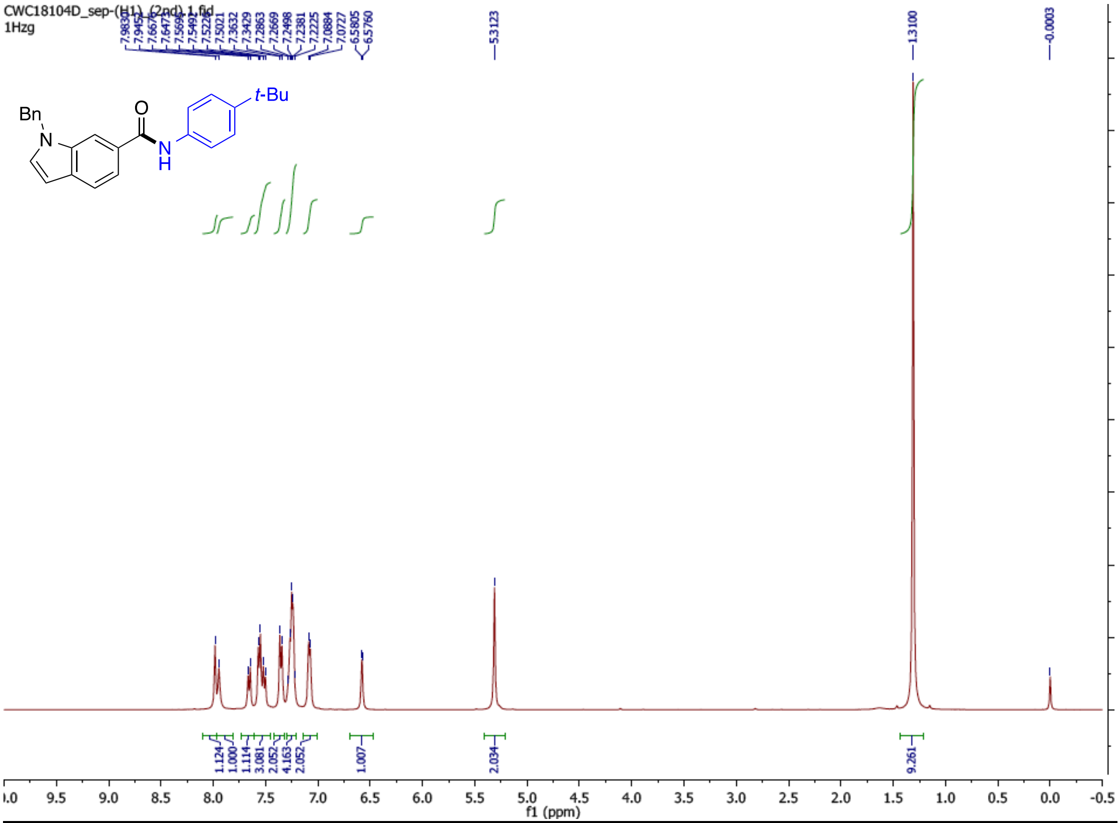

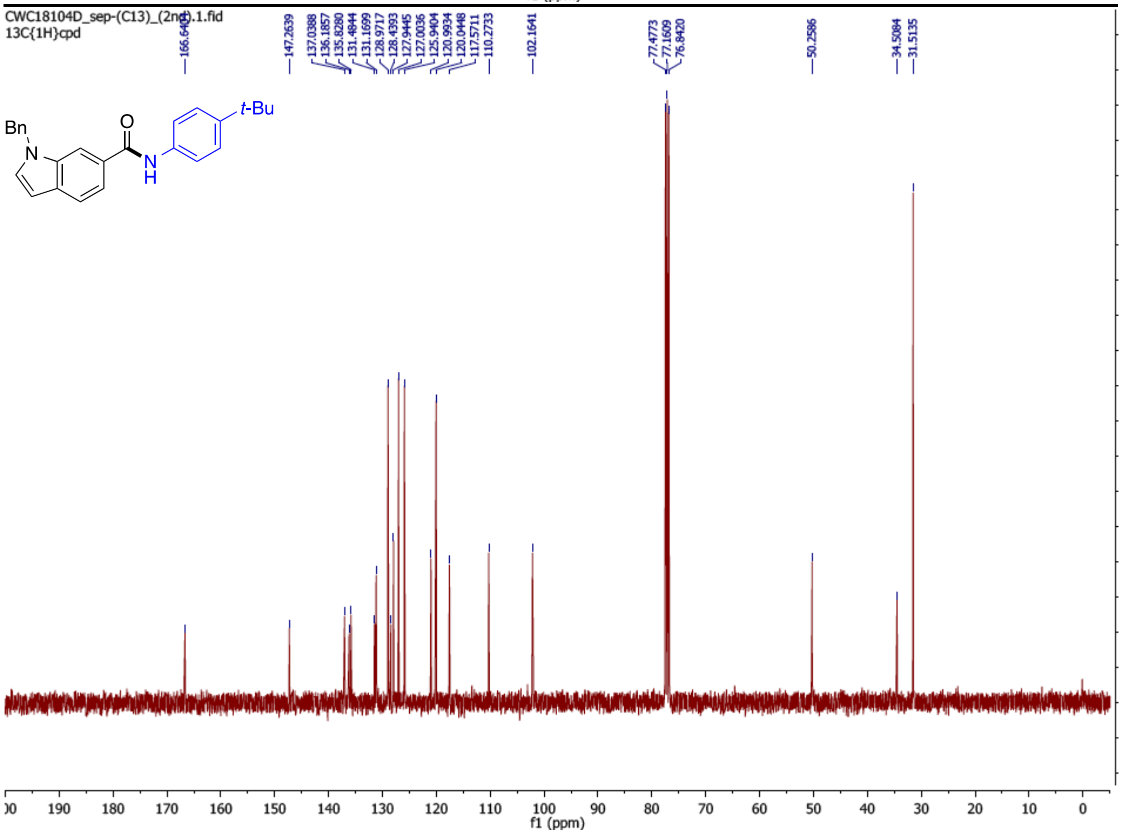

Supplementary Figure 63. ^{1}H and ^{13}C NMR spectra of 1-Benzyl-*N*-(4-(*tert*-butyl)phenyl)-1*H*-indole-6-carboxamide (5s)

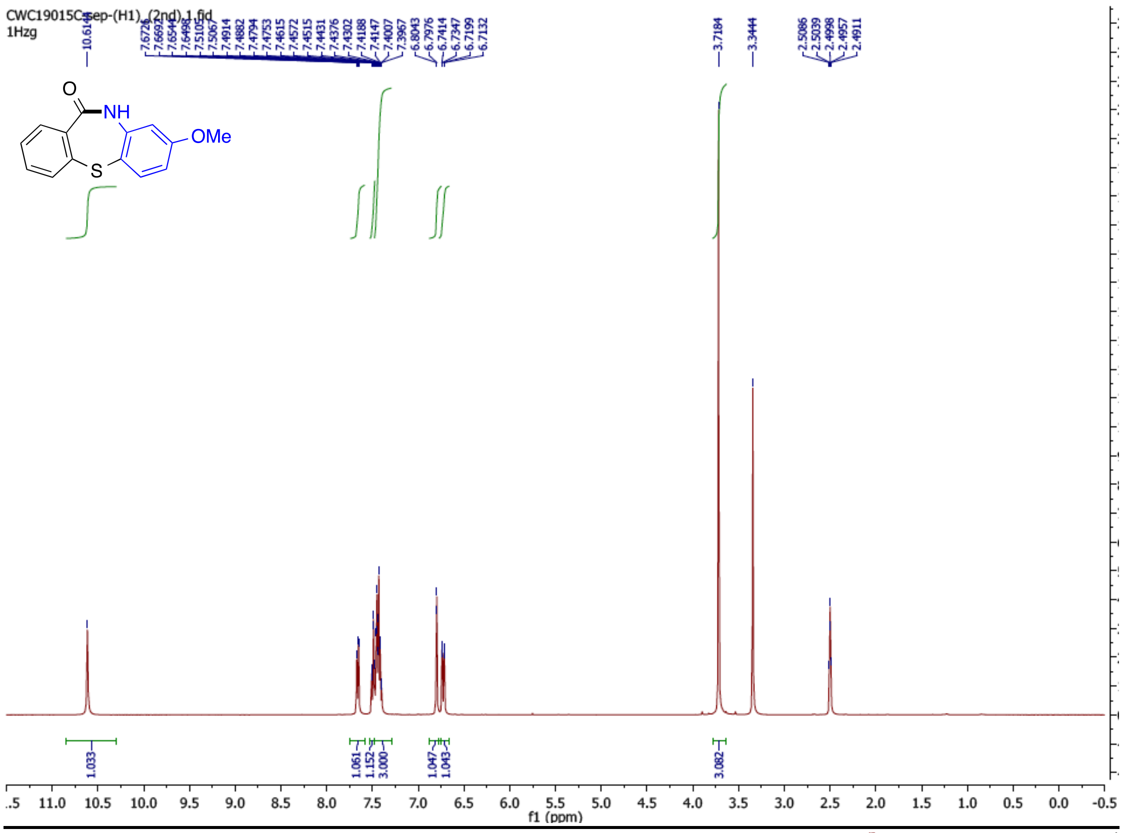

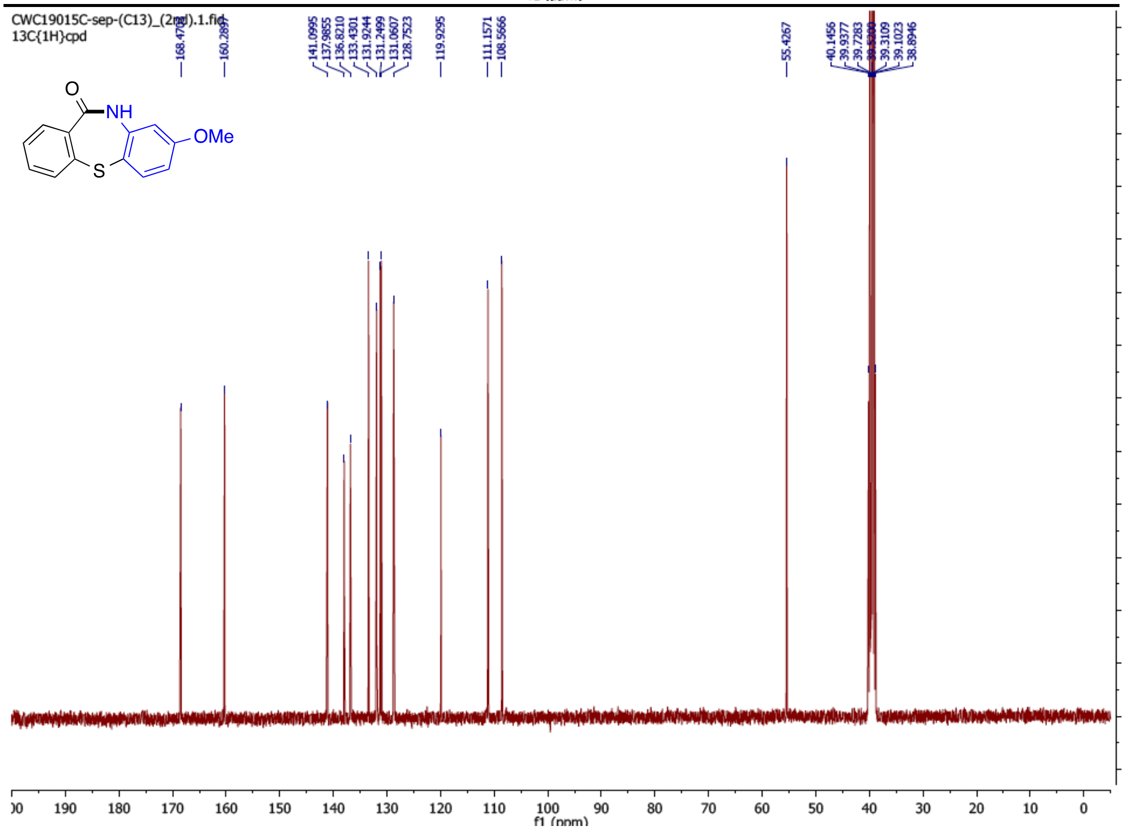

Supplementary Figure 64. **^{1}H and ^{13}C NMR spectra of 8-Methoxydibenzo[*b*,*f*][1,4]thiazepin-11(10*H*)-one (5t)**

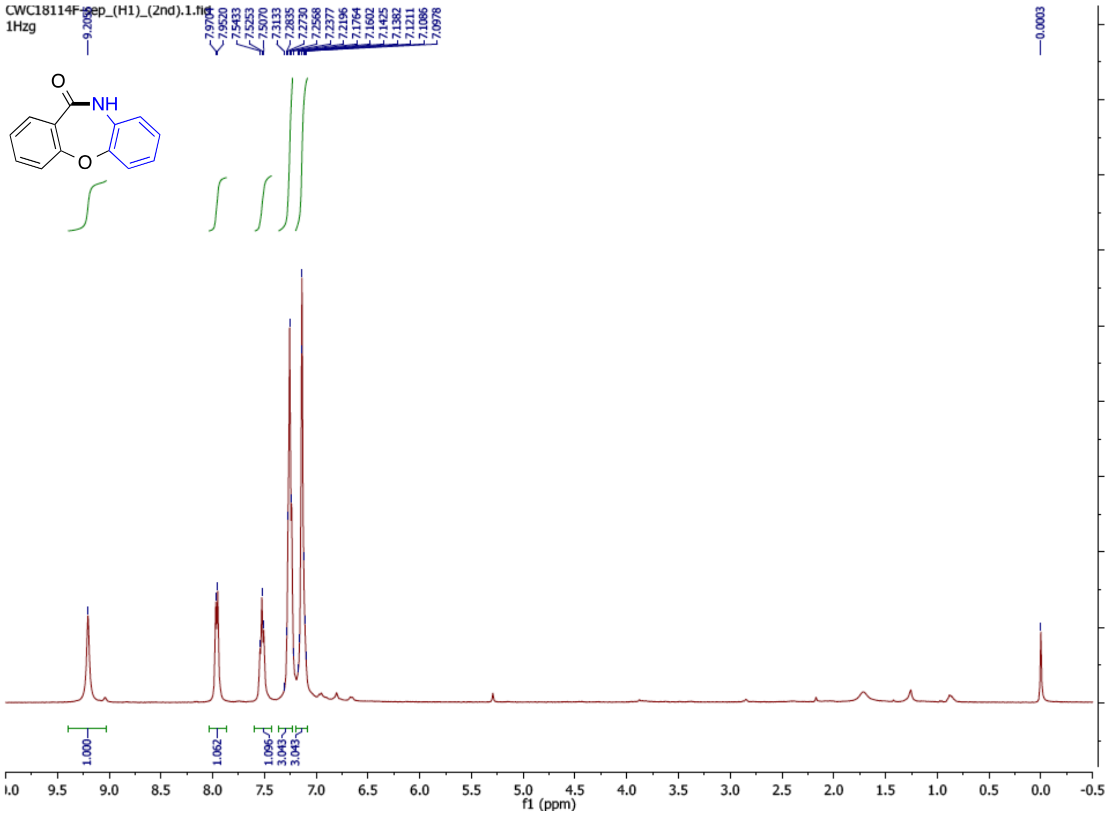

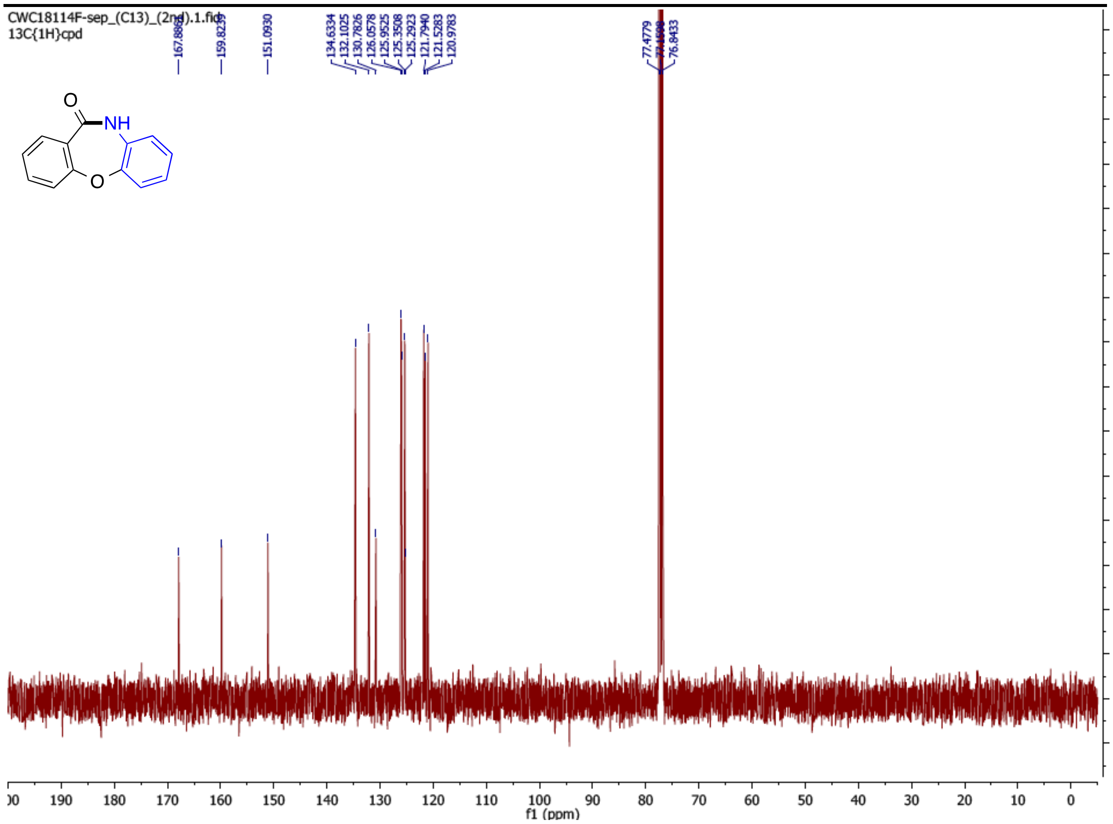

Supplementary Figure 65. ^{1}H and ^{13}C NMR spectra of Dibenzo[*b*,*f*][1,4]oxazepin-11(10*H*)-one (5u)

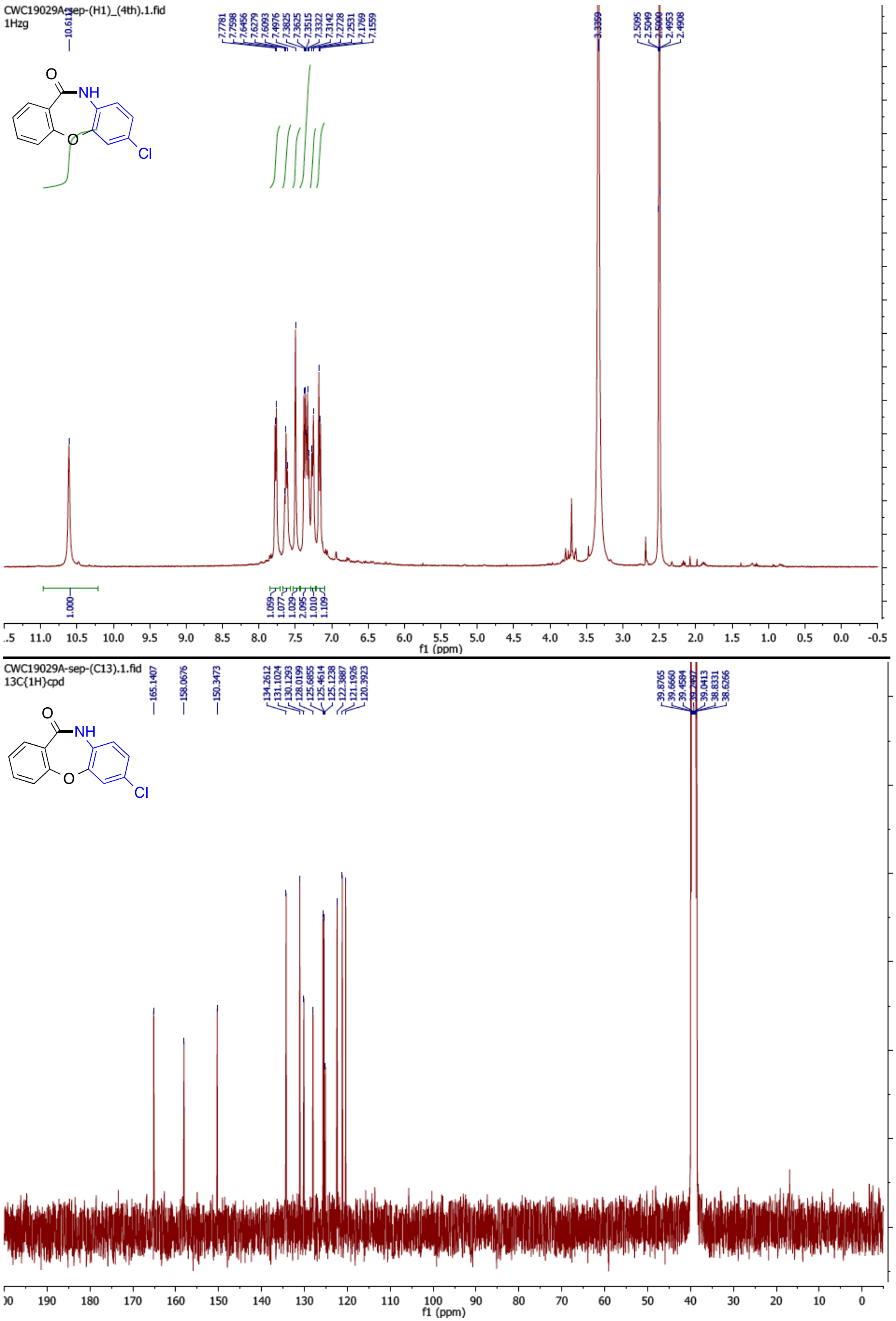

Supplementary Figure 66. ^{1}H **and** ^{13}C **NMR spectra of 7-Chlorodibenzo[*b*,*f*][1,4]oxazepin-11(10*H*)-one (5v)**

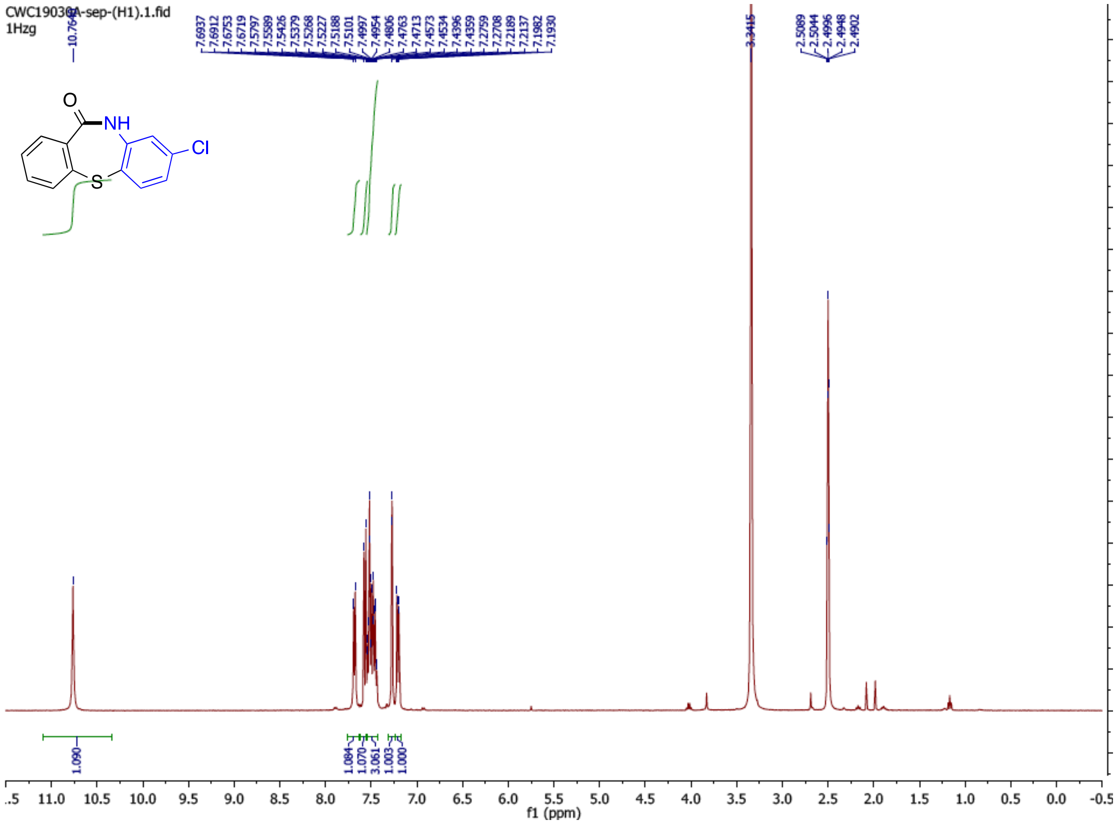

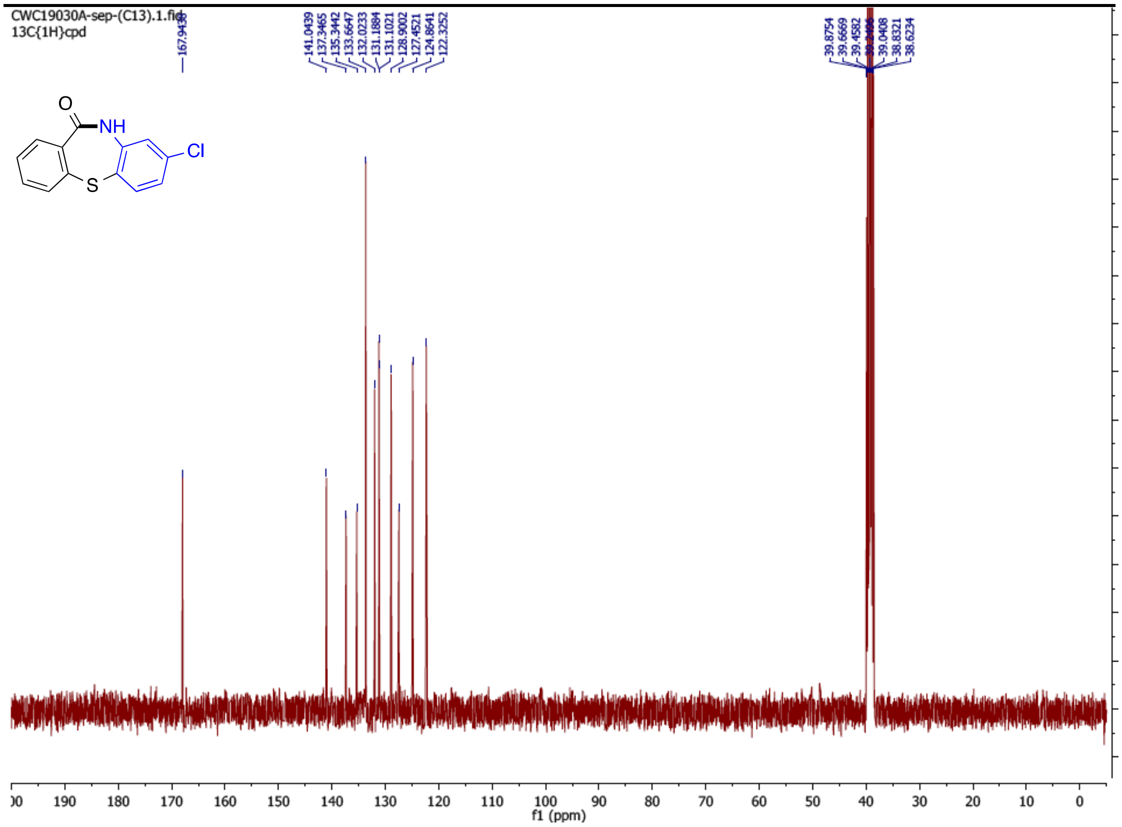

Supplementary Figure 67. ^{1}H and ^{13}C NMR spectra of 8-Chlorodibenzo[*b*,*f*][1,4]thiazepin-11(10*H*)-one (6a)

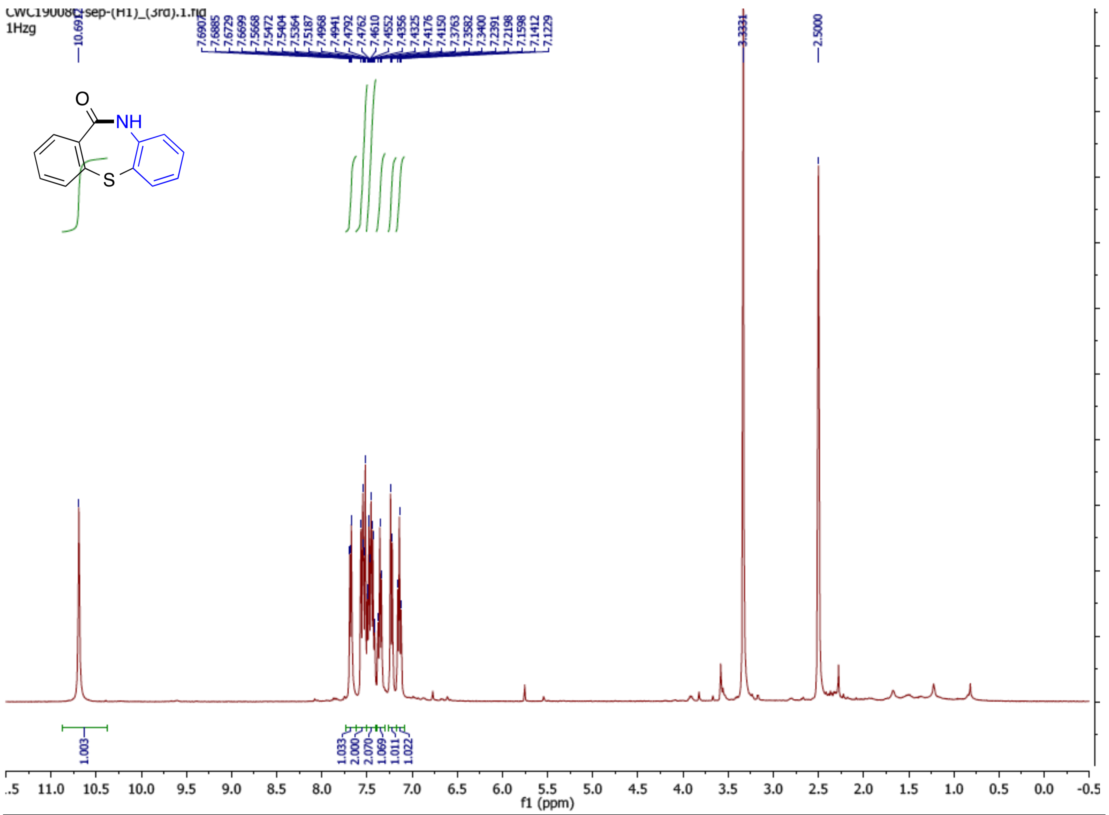

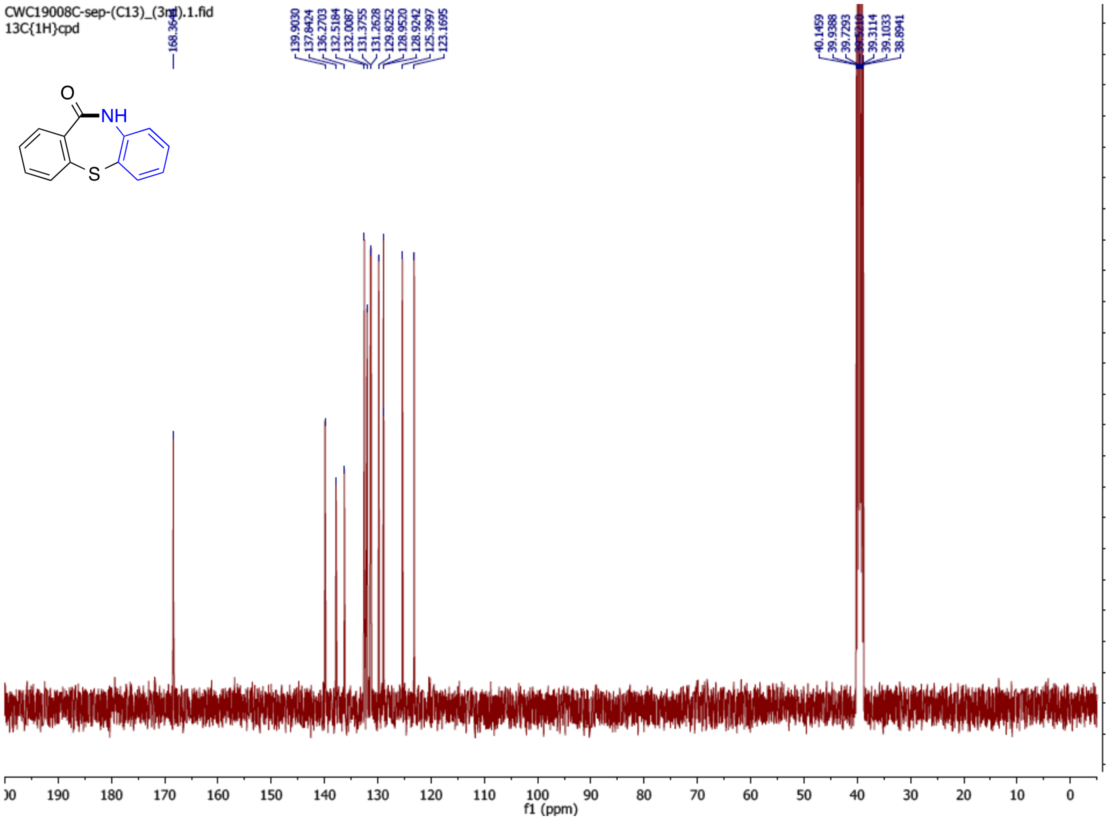

Supplementary Figure 68. ^{1}H and ^{13}C NMR spectra of Dibenzo[*b*,*f*][1,4]thiazepin-11(10*H*)-one (6b)

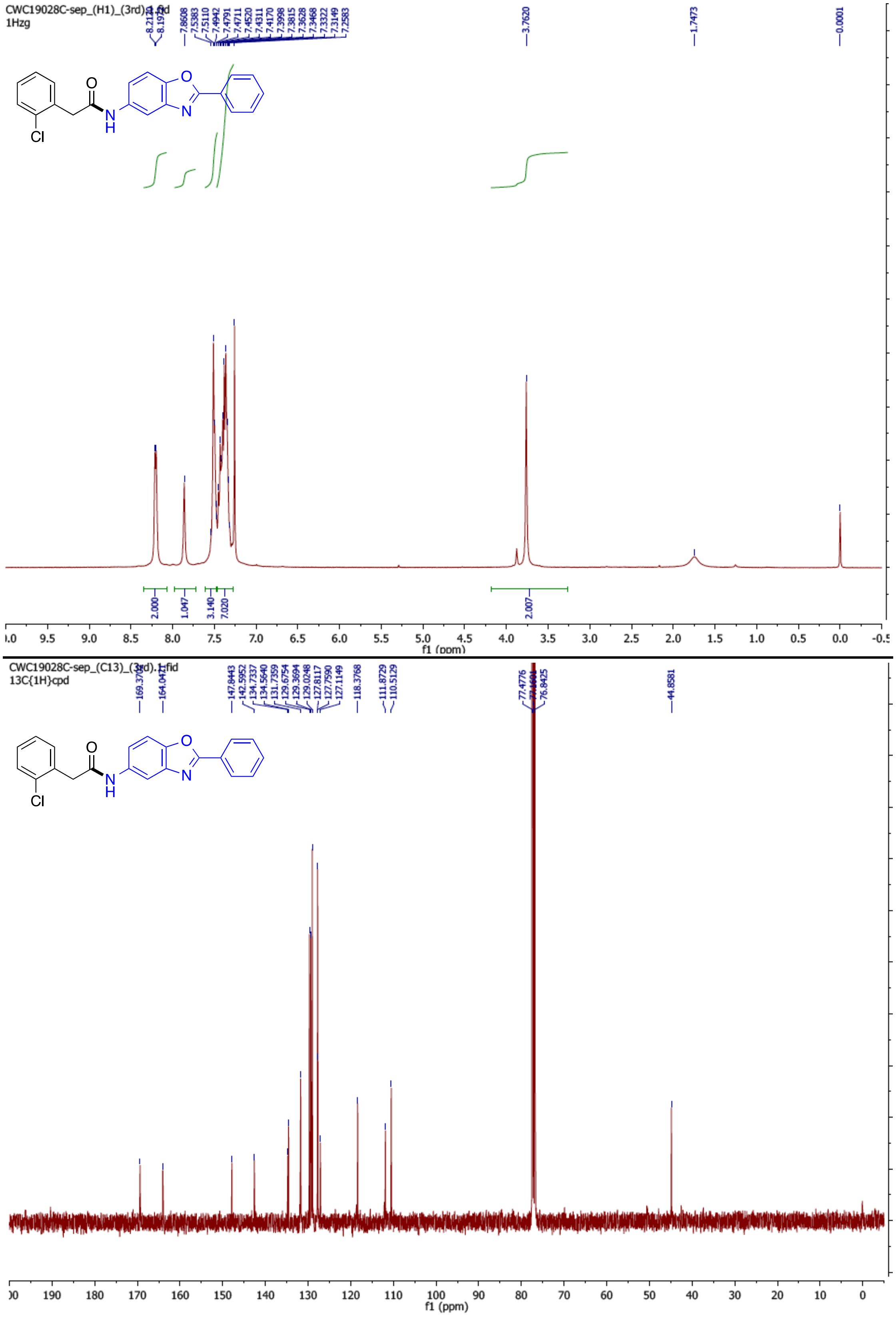

Supplementary Figure 69. ^{1}H and ^{13}C NMR spectra of 2-(2-Chlorophenyl)-*N*-(2-phenylbenzo[*d*]oxazol-5-yl)acetamide (6c)

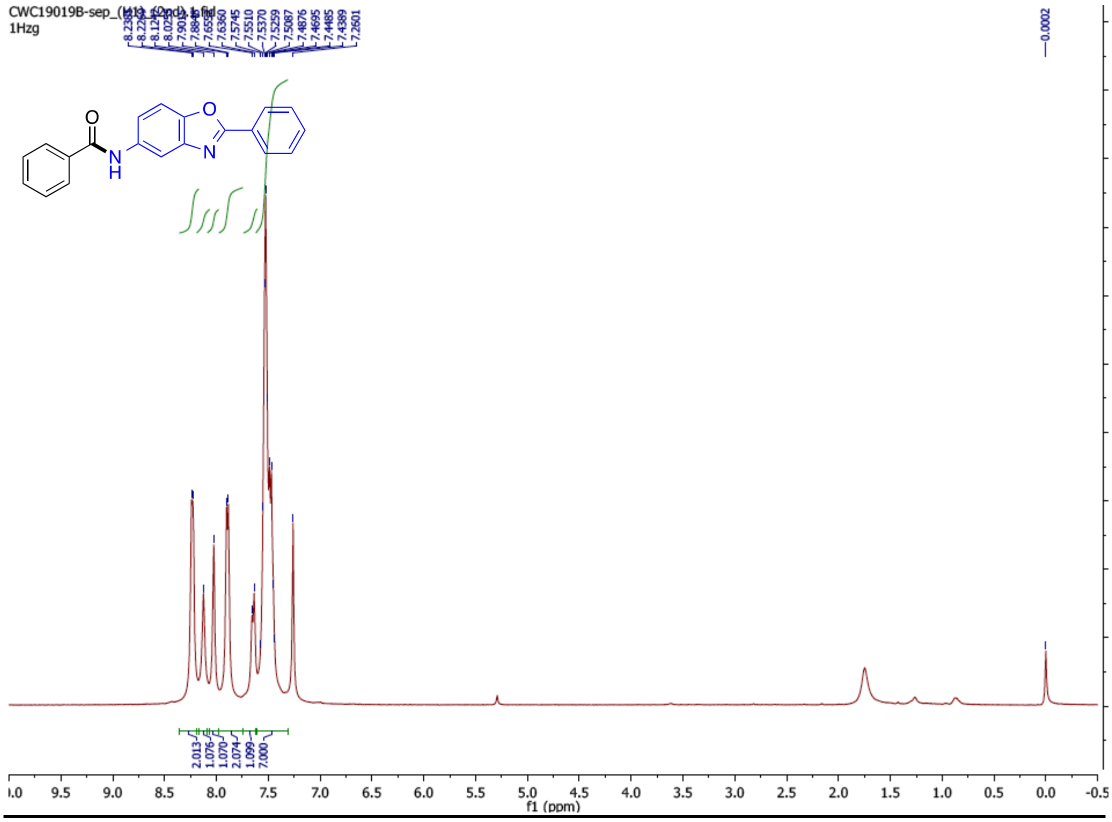

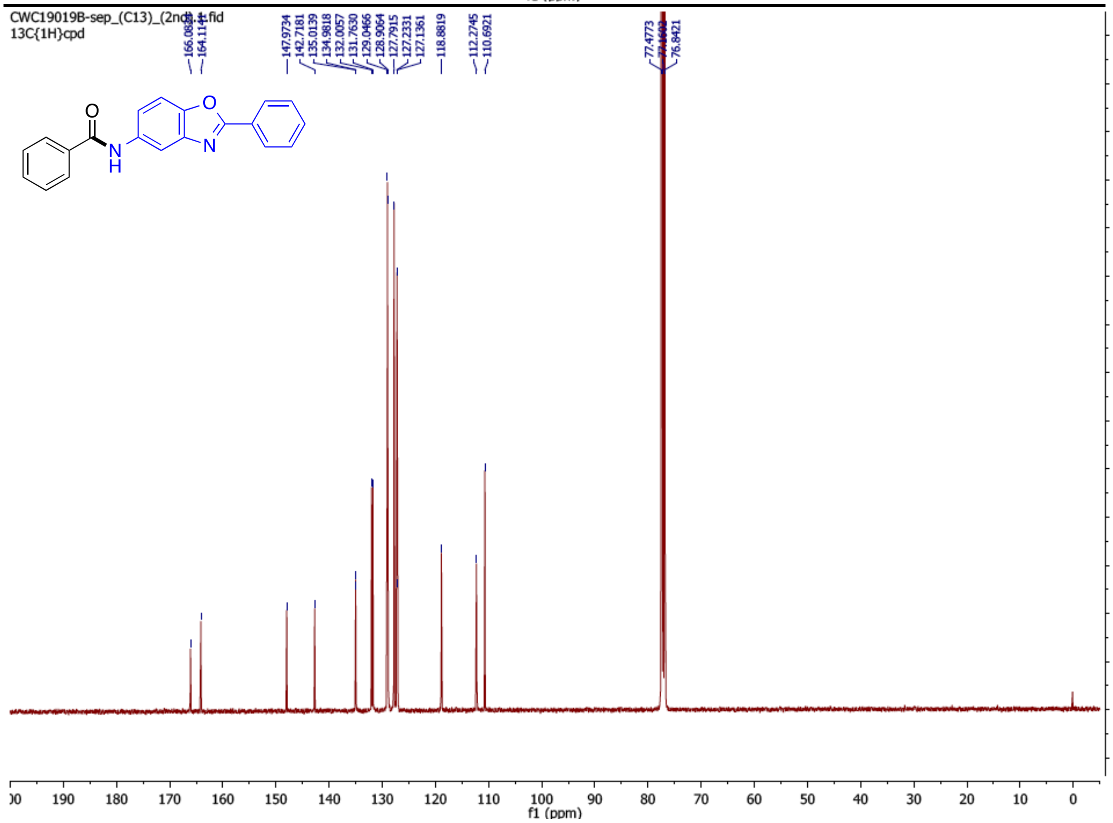

Supplementary Figure 70. **^{1}H and ^{13}C NMR spectra of *N*-(2-Phenylbenzo[*d*]oxazol-5-yl)benzamide (6d)**

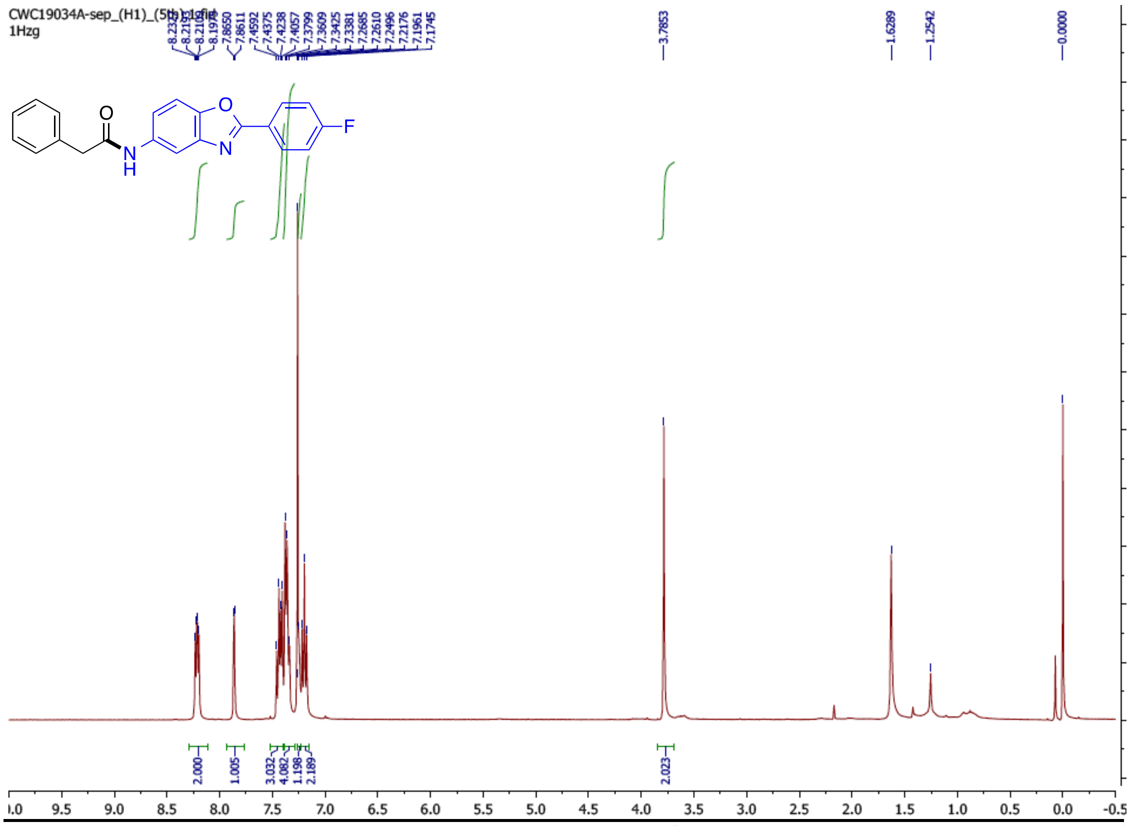

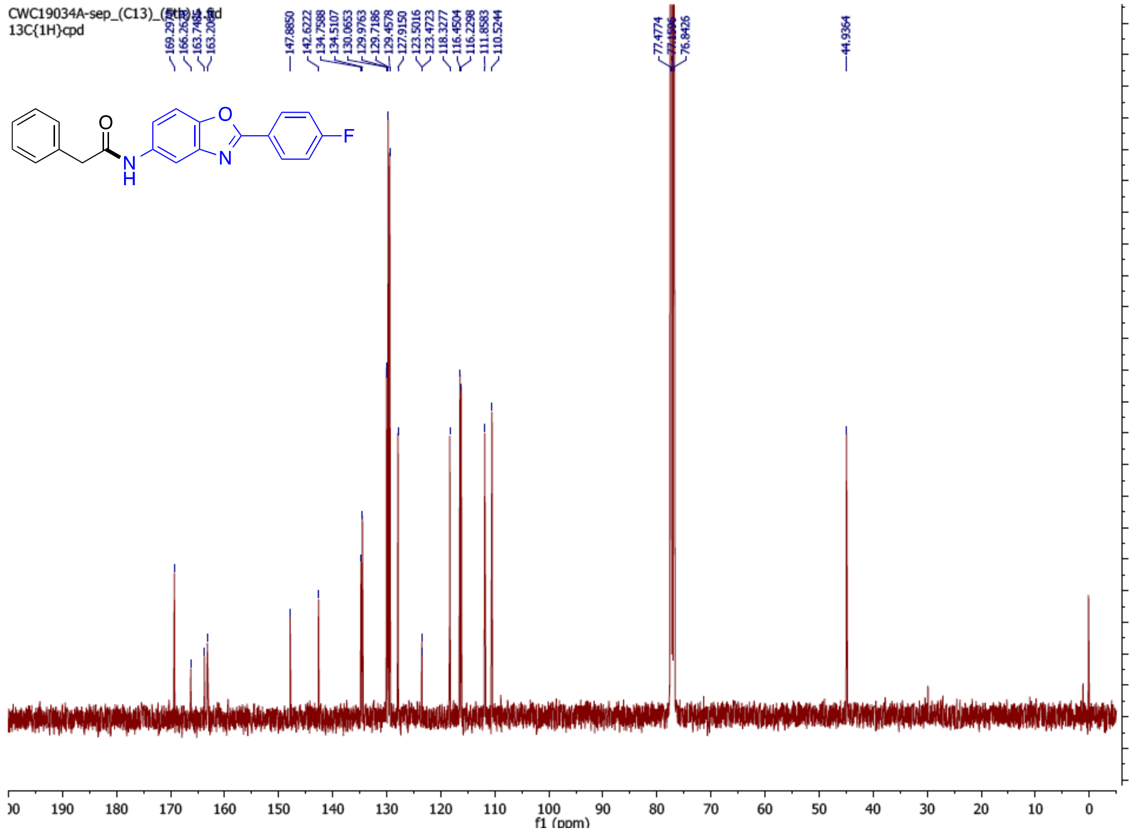

Supplementary Figure 71. ^{1}H and ^{13}C NMR spectra of *N*-(2-(4-Fluorophenyl)benzo[*d*]oxazol-5-yl)-2-phenylacetamide (6e)

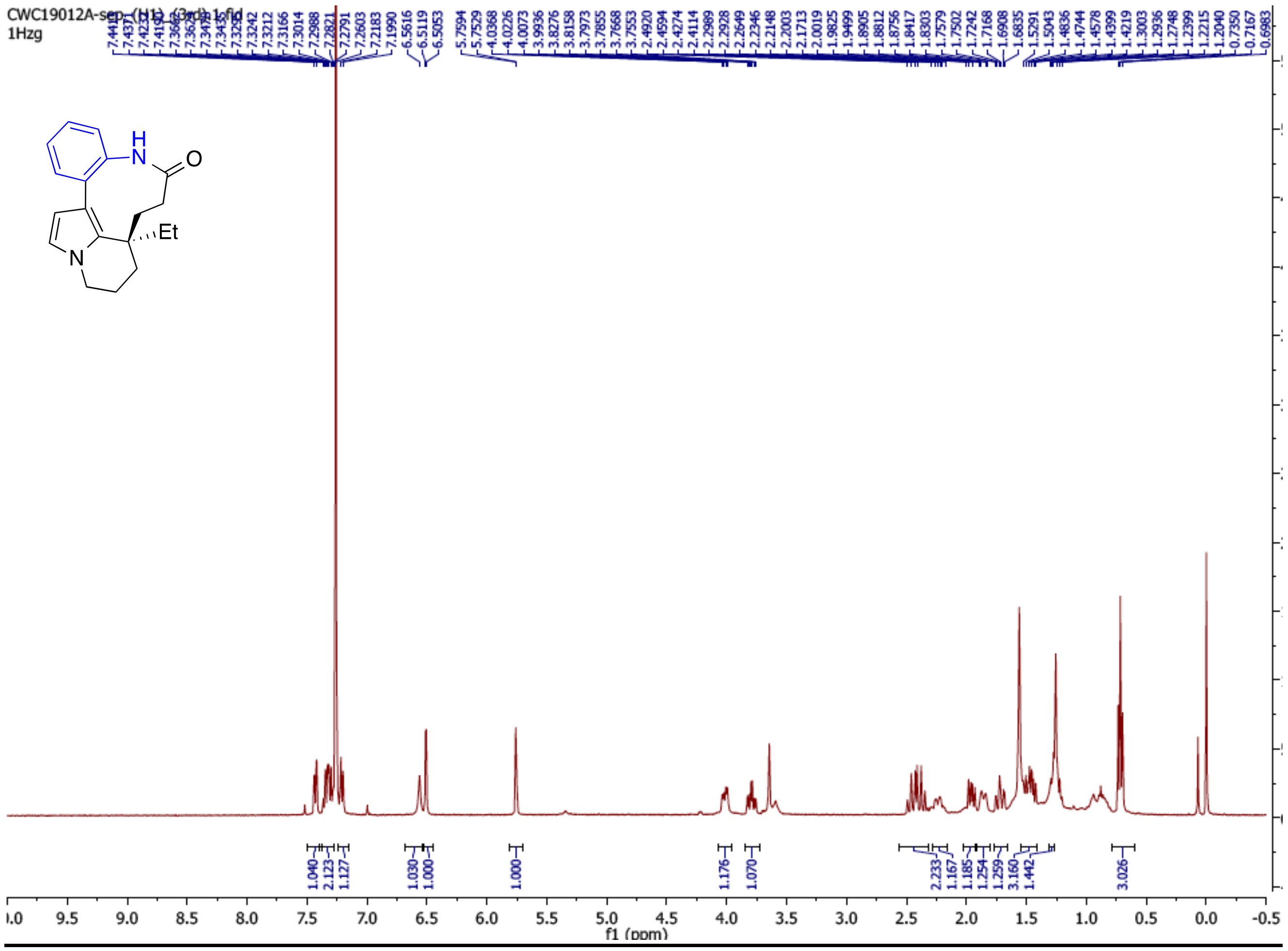

Supplementary Figure 72. ^{1}H and ^{13}C NMR spectra of (*R*)-3a-Ethyl-2,3,3a,4,5,7-hexahydrobenzo[2,3]azonino[6,5,4-*hi*]indolizin-6(1*H*)-one ((-)-Rhazinilam, 6f)

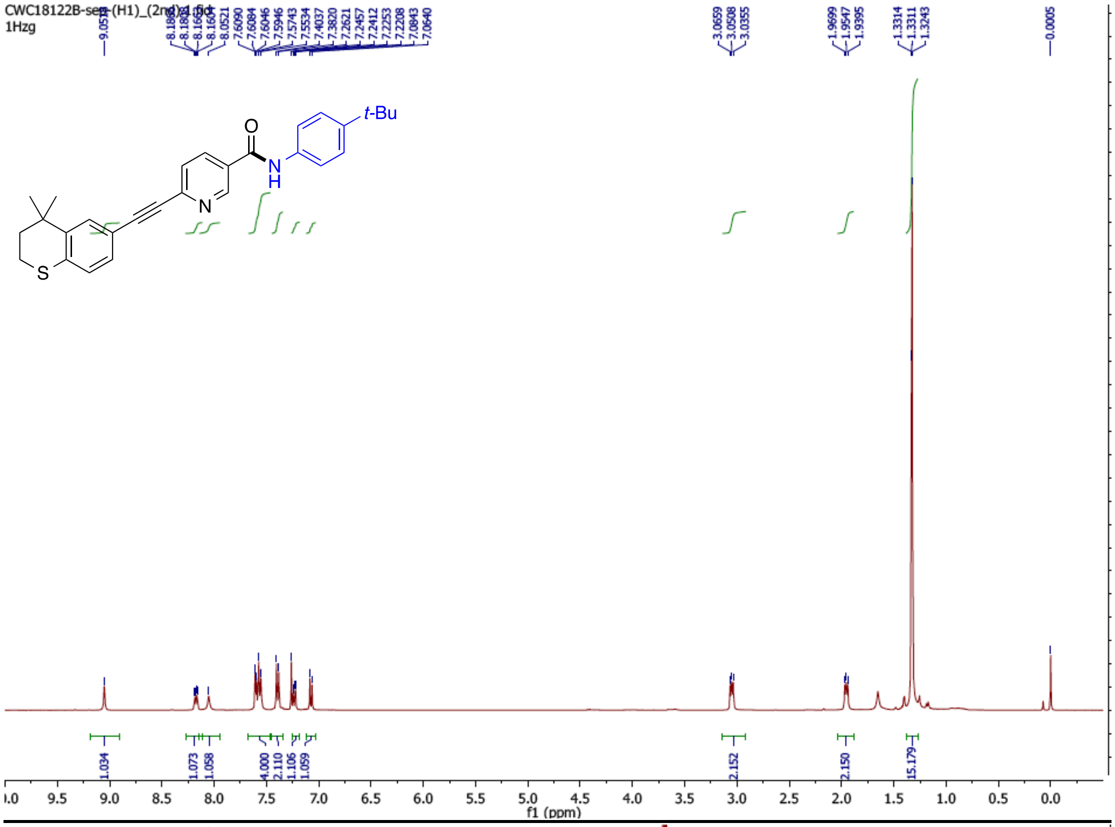

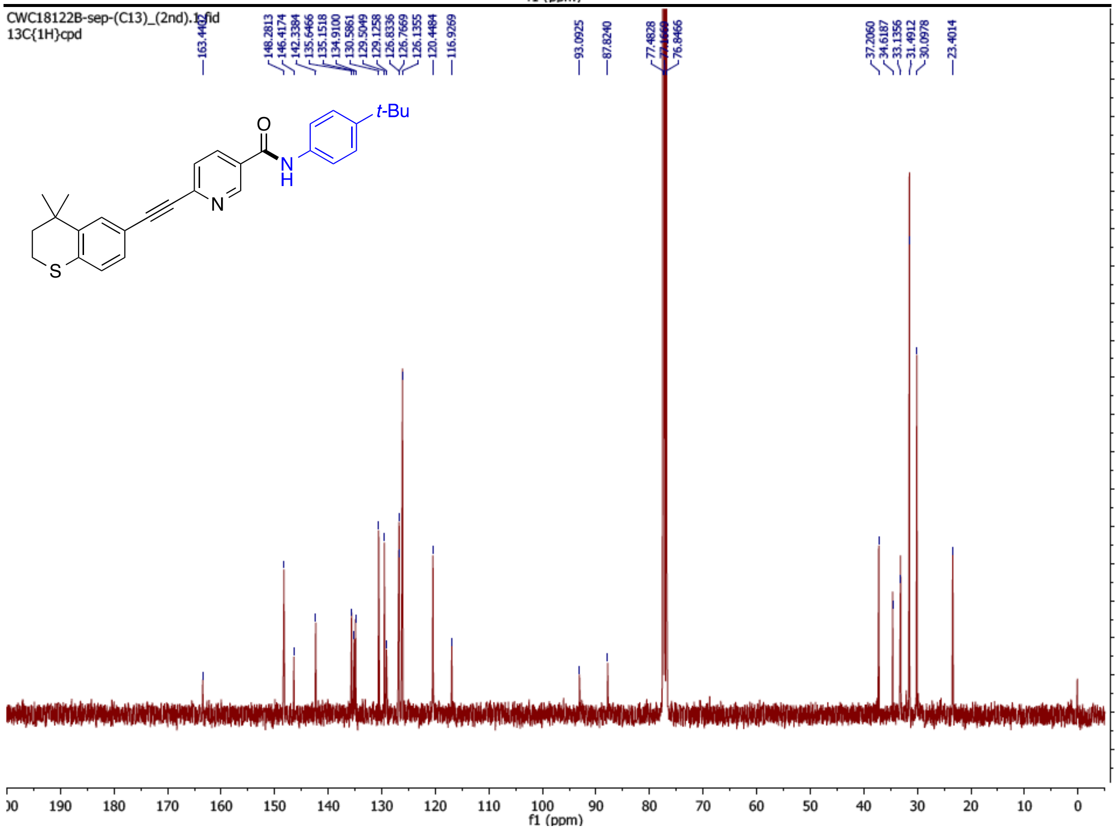

Supplementary Figure 73. ^{1}H **and** ^{13}C **NMR spectra of** ***N*****-(4-(*****tert*****-Butyl)phenyl)-6-((4,4-dimethylthiochroman-6-yl)ethynyl)nicotinamide (6g)**

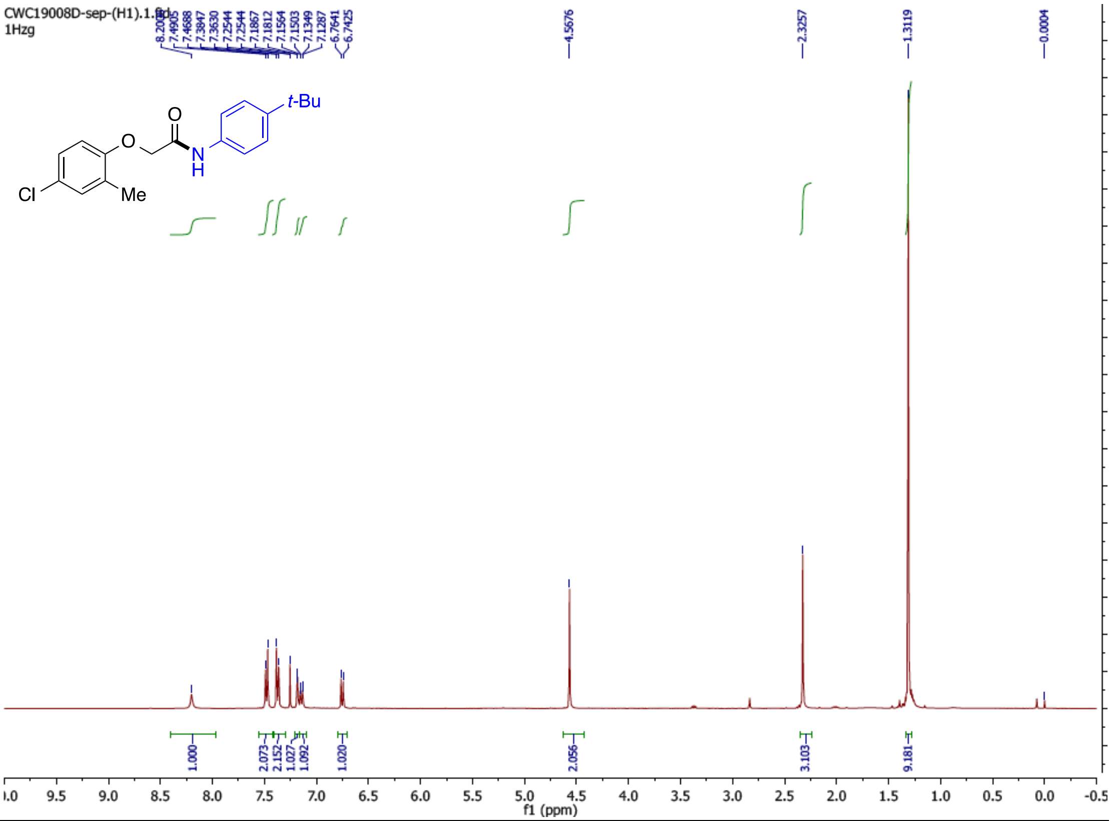

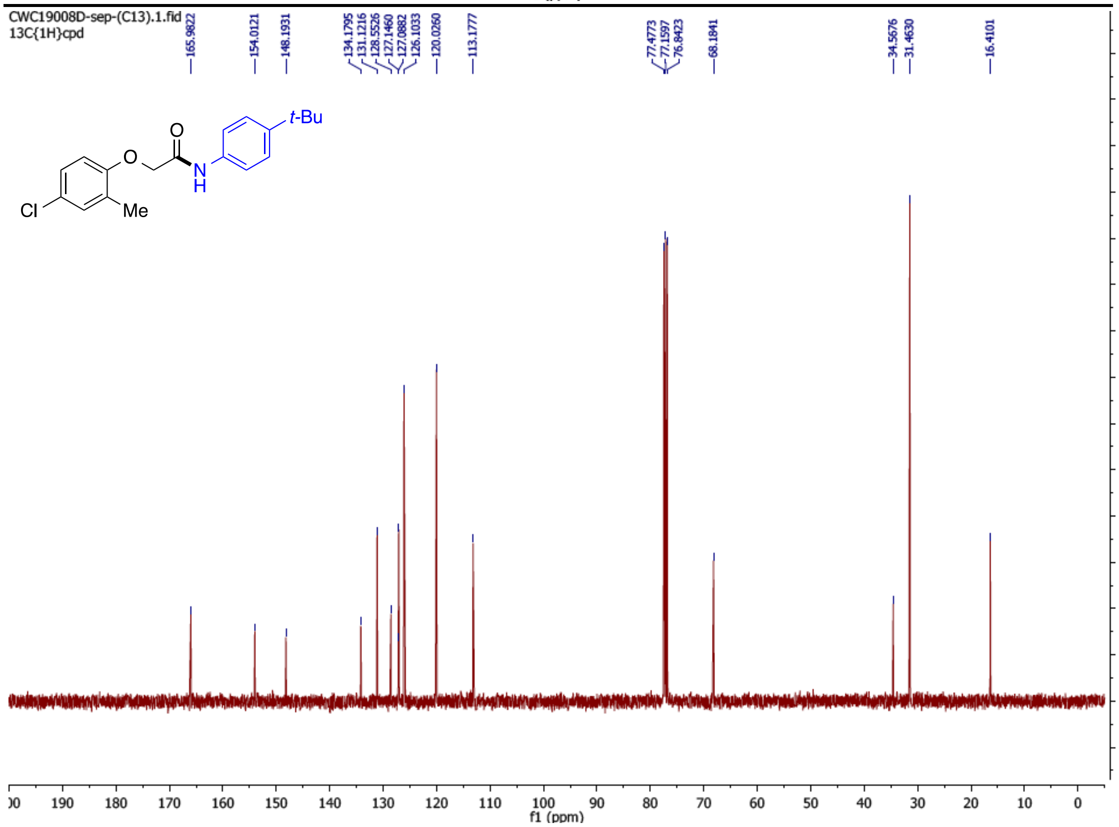

Supplementary Figure 74. ^{1}**H and** ^{13}**C NMR spectra of** ***N*-(4-(*tert*-Butyl)phenyl)-2-(4-chloro-2-methylphenoxy)acetamide (6h)**

Supplementary Tables

Supplementary Table 1. Optimization of Ligand in Nickel-Catalyzed Reductive Coupling of Methyl Benzoate with Nitrobenzene.

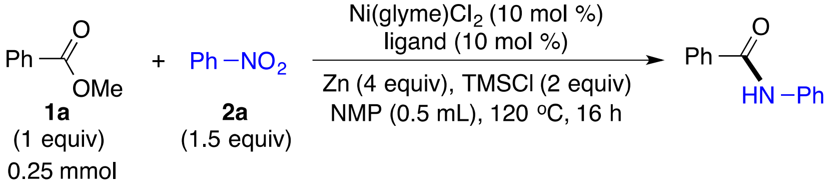

entry	ligand	yield (%)[a]
1	1,10-phenanthroline	85
2	2,2'-dipyridyl	69
3	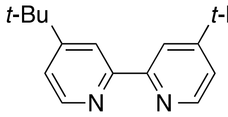	43
4	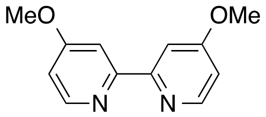	61
5	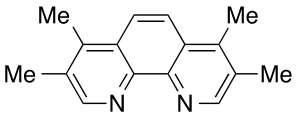	34
6	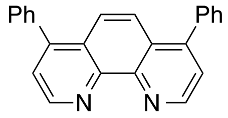	61
7	PCy_3	82[b]
8	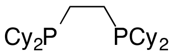	85
9	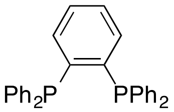	62
10	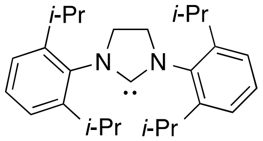	82

(a) GC yield using *n*-dodecane as an internal standard. (b) 20 mol % of ligand was used.

Supplementary Table 2. Optimization of Loading of Nitrobenzene in Nickel-Catalyzed Reductive Coupling of Methyl Benzoate with Nitrobenzene.

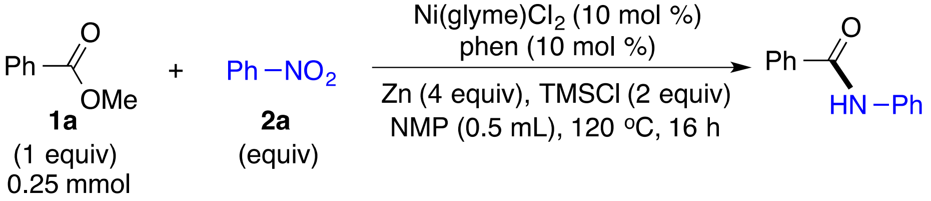

entry	$PhNO_2$ (equiv)	yield (%)[a]
1	1.5	81
2	1.7	73
3	1.3	93
4	1.2	81

(a) GC yield using *n*-dodecane as an internal standard.

Supplementary Table 3. Optimization of Loadings of Zinc and Chlorotrimethylsilane in Nickel-Catalyzed Reductive Coupling of Methyl Benzoate with Nitrobenzene.

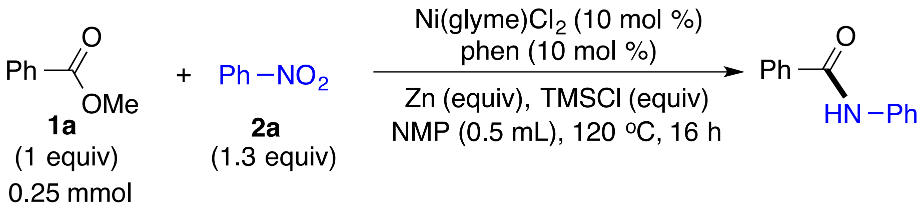

entry	Zn (equiv)	TMSCl (equiv)	yield (%)[a]
1	4	2	93
2	3.5	2	62
3	4	1.5	61
4	3.5	1.5	71
5	4.5	1.5	88

(a) GC yield using *n*-dodecane as an internal standard.

Supplementary Table 4. Optimization of Loadings of Ni(glyme)Cl_2 and 1,10-phenanthroline in Nickel-Catalyzed Reductive Coupling of Methyl Benzoate with Nitrobenzene.

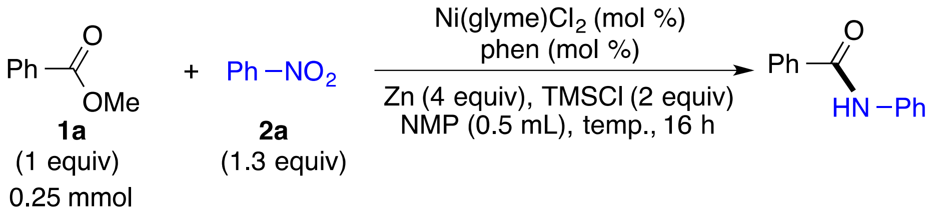

entry	Ni(glyme)Cl_2 (mol %)	phen (mol %)	yield (%)[a]
1	10	10	93
2	7.5	7.5	94
3	5	5	85
4	5	10	85
5	7.5	15	82

(a) GC yield using *n*-dodecane as an internal standard.

Supplementary Table 5. Optimization of temperature in Nickel-Catalyzed Reductive Coupling of Methyl Benzoate with Nitrobenzene.

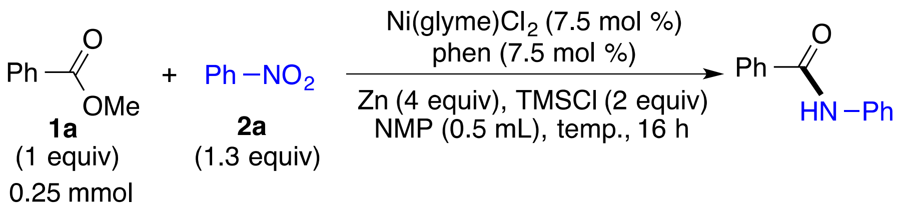

entry	temp. (°C)	yield (%)[a]
1	120	94
2	130	83
3	110	85
4	100	84

(a) GC yield using *n*-dodecane as an internal standard.

Supplementary Table 6. Optimization of Solvent in Nickel-Catalyzed Reductive Coupling of Methyl Benzoate with Nitrobenzene

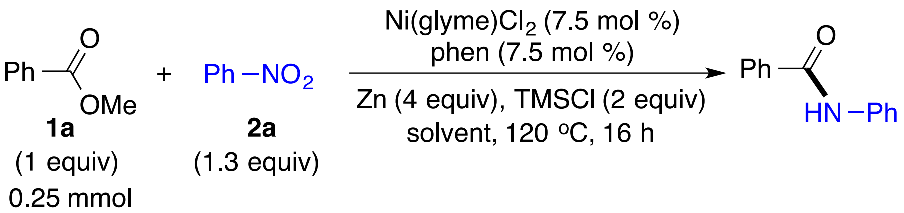

entry	solvent (mL)	yield (%)[a]
1	NMP (0.5)	94
2	DMA (0.5)	88
3	DMF (0.5)	59
4	DMSO (0.5)	6
5	1,4-dioxane (0.5)	36
6	NMP (1)	57

(a) GC yield using *n*-dodecane as an internal standard.

Supplementary Table 7. Optimization of Reductant in Nickel-Catalyzed Reductive Coupling of Methyl Benzoate with Nitrobenzene.

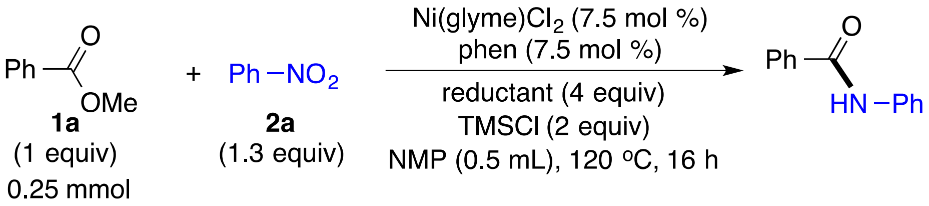

entry	reductant	yield (%)[a]
1	Zn	93
2	Mn	75

(a) GC yield using *n*-dodecane as an internal standard.

Supplementary Table 8. Optimization of Halotrimethylsilane Additive in Nickel-Catalyzed Reductive Coupling of Methyl Benzoate with Nitrobenzene.

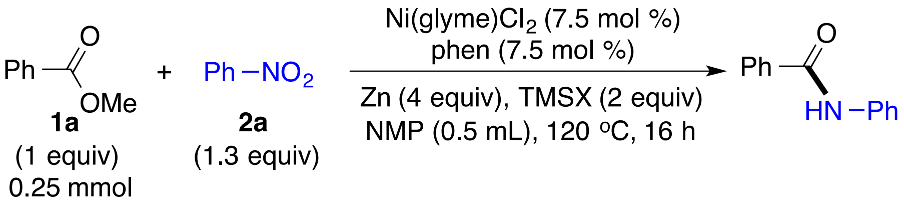

entry	TMSX	yield (%)[a]
1	TMSCl	94
2	TMSBr	84
3	TMSI	58

(a) GC yield using *n*-dodecane as an internal standard.

Supplementary Table 9. Optimization of Transition Metal Catalyst in Nickel-Catalyzed Reductive Coupling of Methyl Benzoate with Nitrobenzene.

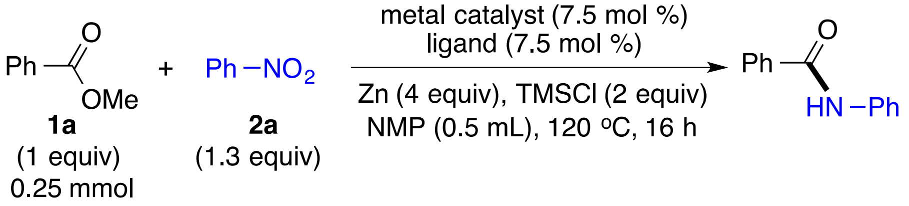

entry	metal catalyst	yield (%)[a]
1	$Ni(glyme)Cl_2$	94
2	$Ni(glyme)Br_2$	77
3	$Ni(diglyme)Br_2$	91
4	$NiBr_2$	87
5	$NiCl_2$	87
6	$Ni(COD)_2$	82
7	$FeCl_2 \cdot 4H_2O$	12
8	$FeBr_2$	19
9	$CoCl_2$	16
10	$Mn(OAc)_2 \cdot 4H_2O$	12
11	CuI	13

(a) GC yield using *n*-dodecane as an internal standard.

Supplementary Table 10. Control Experiments in Nickel-Catalyzed Reductive Coupling of Methyl Benzoate with Nitrobenzene.

"standard conditions":

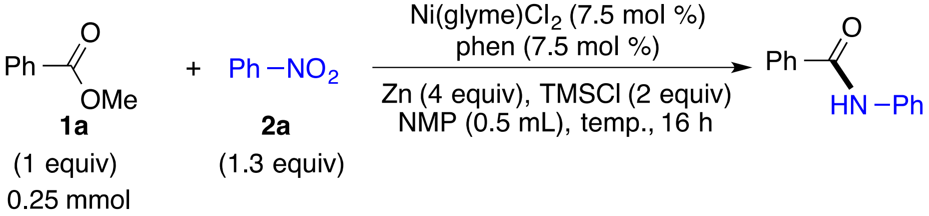

entry	variations from standard conditions	yield (%)[a]
1	----	94
2	no metal catalyst	7 (30% conv.)
3	no ligand	67
4	no TMSCl	0 (7% conv.)
5	no Zn	0 (0% conv.)

(a) GC yield using *n*-dodecane as an internal standard.

Supplementary Table 11. Optimizations of Nickel-Catalyzed Reductive Coupling of Methyl Decanoate with Nitrobenzene.

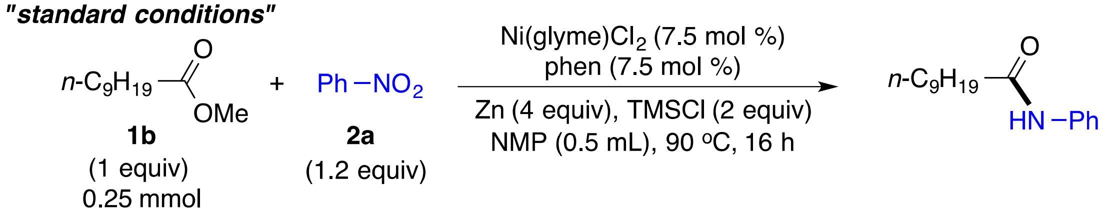

entry	variations from standard conditions	yield (%)[a]
1	----	100
2	$Ni(glyme)Br_2$ instead of $Ni(glyme)Cl_2$	90
3	$Ni(diglyme)Br_2$ instead of $Ni(glyme)Cl_2$	90
4	$NiCl_2$ instead of $Ni(glyme)Cl_2$	85
5	$NiBr_2$ instead of $Ni(glyme)Cl_2$	87
6	$NiCl_2 \cdot 6H_2O$ instead of $Ni(glyme)Cl_2$	7 (93% conv.)
7	$FeCl_2 \cdot 4H_2O$ instead of $Ni(glyme)Cl_2$	25 (34% conv.)
8	$FeBr_2$ instead of $NiCl_2(glyme)$	24 (48% conv.)
9	$CoCl_2$ instead of $NiCl_2(glyme)$	20 (34% conv.)
10	CuI instead of $NiCl_2(glyme)$	3 (10% conv.)
11	2,2'-dipyridyl instead of phen	94
12	4,4'-di-*tert*-butyl-2,2'-dipyridyl instead of phen	94
13	3,4,7,8-tetramethyl-1,10-phenanthroline instead of phen	95
14	bathophenanthroline instead of phen	69 (83% conv.)
15	Mn instead of Zn	84
16	$Ni(glyme)Cl_2$ (5 mol %)/phen (5 mol %) instead of $Ni(glyme)Cl_2$ (7.5 mol %)/phen (7.5 mol %)	82
17	$Ni(glyme)Cl_2$ (10 mol %)/phen (10 mol %) instead of $Ni(glyme)Cl_2$ (7.5 mol %)/phen (7.5 mol %)	97
18	Zn (3.5 equiv) instead of (4 equiv)	55
19	TMSCl (1.5 equiv) instead of (2 equiv)	84
20	Zn (3.5 equiv)/TMSCl (1.5 equiv) instead of Zn (4 equiv)/TMSCl (2 equiv)	69
21	$PhNO_2$ (1.1 equiv) instead of (1.2 equiv)	86
22	70 °C instead of 90 °C	61 (70% conv.)
control experiments:		
23	no metal catalyst	2 (87% conv.)
24	no ligand	77
25	no TMSCl	0 (4% conv.)
26	no Zn	0 (7% conv.)

(a) GC yield using *n*-dodecane as an internal standard.

Supplementary Table 12. Optimization of Zn and TMSCl loading for the reaction between nitrosobenzene and methyl decanoate.

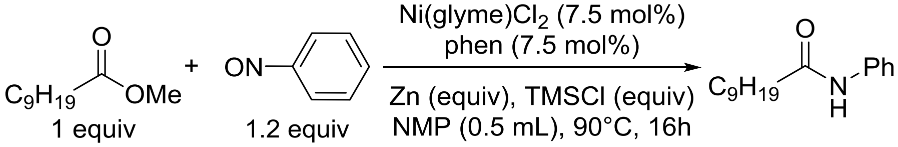

entry	Zn (equiv)	TMSCl (equiv)	conversion (%)	yield (%)
1	4	2	100	100
2	3	2	100	100
3	3	1	91	87
4	2	1	22	12

Supplementary Table 13. Optimizaion of Zn and TMSCl loading for the reaction between *N*-phenylhydroxyamine and methyl decanoate.

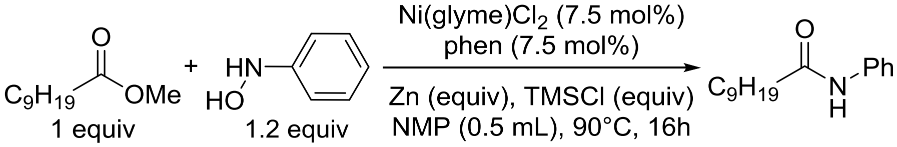

entry	Zn (equiv)	TMSCl (equiv)	conversion (%)	yield (%)
1	4	2	78	74
2	3	2	60	59
3	2	2	17	15
4	2	1	11	4

Supplementary Table 14. Screening of conditions for the reaction between aniline and methyl decanoate.

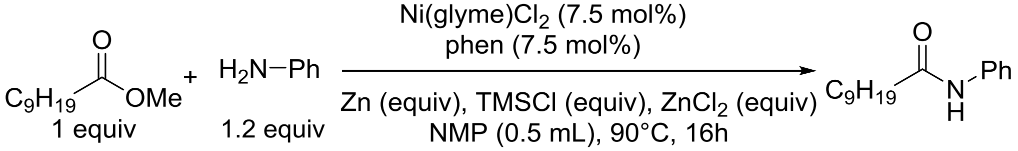

entry	Zn (equiv)	TMSCl (equiv)	$ZnCl_2$ (equiv)	conversion (%)	yield (%)
1	4	2	0	14	12
2	2	1	0	27	28
3	2	0	0	0	0
4	1	1	0	20	17
5	1	0	0	0	0
6	4	2	2	18	3
7	2	1	2	25	19
8	2	0	2	45	44
9	1	1	2	21	20
10	1	0	2	44	39
11	0	0	2	0	0

Supplementary Table 15. Optimizaion of Zn and TMSCl loading for the reaction between azoxybenzene and methyl decanoate

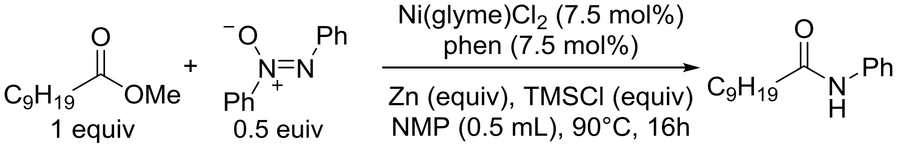

entry	Zn (equiv)	TMSCl (equiv)	conversion (%)	yield (%)
1	4	1	85	81
2	4	0.5	95	93
3	4	0.091	2	6
4	3	0.5	96	99
5	2	0.5	91	81
6	1	0.5	53	45

Supplementary Methods

(A) General Analytical Information.

Nuclear Magnetic Resonance spectra were recorded on a Bruker Avance 400 MHz instruments at ambient temperature. All ^{1}H NMR spectra were measured in part per million (ppm) relative to the signal of tetramethylsilane (TMS) added into the deuterated chloroform ($CDCl_3$, 0.00 ppm), the signal of residual dichloromethane in deuterated dichloromethane (CD_2Cl_2, 5.32 ppm), or the signal of residual dimethyl sulfoxide in deuterated dimethyl sulfoxide (DMSO-d_6, 2.50 ppm).[1] Data for ^{1}H NMR were reported as follows: chemical shift, multiplicity (s = singlet, d = doublet, t = triplet, q = quartet, qu = quintet, m = multiplet, ovrlp = overlap, br = broad), coupling constants, and integration. All ^{13}C NMR spectra were reported in ppm relative to $CDCl_3$ (77.16 ppm), CD_2Cl_2 (53.84 ppm), or DMSO-d_6 (39.52 ppm)[1] and were obtained with complete ^{1}H decoupling. The ^{19}F NMR spectra were obtained with complete ^{1}H decoupling as well. α,α,α-Trifluorotoluene was used as the internal standard, with a chemical shift of -63.73 ppm relative to $CFCl_3$. All GC analyses were performed on a Perkin-Elmer Clarus 400 GC system with a FID detector. All GC-MS analyses were performed on an Agilent Technologies 7890A GC system equipped with a 5975C MS detector. High-resolution mass spectra (HRMS) by electrospray ionization (ESI) method were performed at the EPFL ISIC Mass Spectroscopy Service with a Micro Mass QTOF Ultima spectrometer.

(B) General Reagent Information.

Unless otherwise noted, all chemicals were used as received without further purifications. Anhydrous *N*-methylpyrrolidone (NMP) (99.8% purity, in Sure-Seal bottle), zinc powder (Zn, >98% purity), manganese powder (Mn, 99.99% purity), chlorotrimethylsilane (TMSCl, ≥98% purity), and nickel(II) chloride ethylene glycol dimethyl ether complex (Ni(glyme)Cl_2) were purchased from Aldrich Chemical Co.. 1,10-Phenanthroline (phen, ≥99% purity) was purchased from Acros Chemicals. Iodotrimethylsilane (TMSI, ≥95% purity) was purchased from TCI Chemicals. The following known starting materials (esters and nitroarenes) were prepared according to the literature procedures:[2-16]

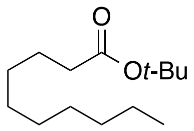

tert-butyl decanoate[2]

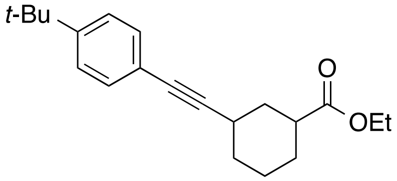

(d.r. ~ 4.4:1)
ethyl 3-((4-(*tert*-butyl)phenyl)ethynyl)-cyclohexane-1-carboxylate[3]

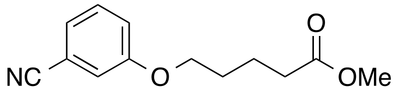

methyl 5-(3-cyanophenoxy)pentanoate[4]

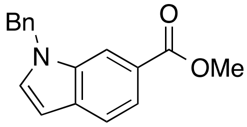

methyl 1-benzyl-1*H*-indole-6-carboxylate[5]

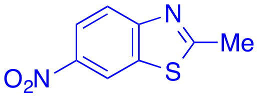

2-methyl-6-nitrobenzo[*d*]thiazole[6]

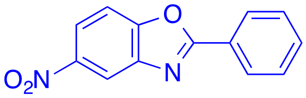

5-nitro-2-phenylbenzo[*d*]oxazole[7]

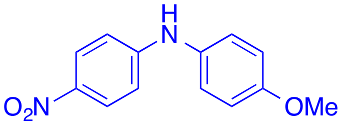

4-methoxy-*N*-(4-nitrophenyl)aniline[8]

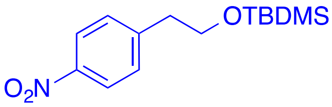

tert-butyldimethyl(4-nitrophenethoxy)silane[9]

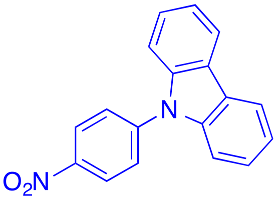

9-(4-nitrophenyl)-9*H*-carbazole[10]

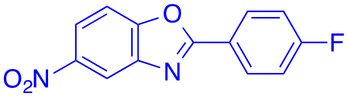

2-(4-fluorophenyl)-5-nitrobenzo[*d*]oxazole[11]

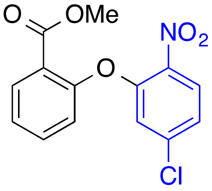

methyl 2-(5-chloro-2-nitrophenoxy)-benzoate[12]

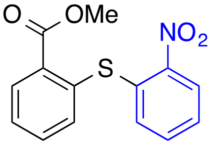

methyl 2-((2-nitrophenyl)thio)-benzoate[13]

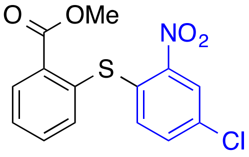

methyl 2-((4-chloro-2-nitrophenyl)thio)-benzoate[14]

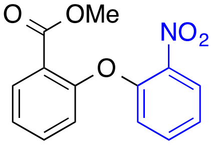

methyl 2-(2-nitrophenoxy)benzoate[15]

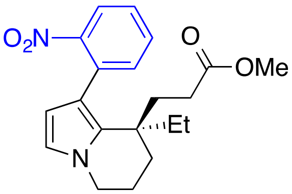

methyl (*R*)-3-(8-ethyl-1-(2-nitrophenyl)-5,6,7,8-tetrahydroindolizin-8-yl)-propanoate[16]

(C) General Manipulation Considerations.

All manipulations for the nickel-catalyzed reductive coupling reactions of nitroarenes with esters were set up in a 30 mL Teflon-screw capped test tubes (unless otherwise noted) under an inert nitrogen (N_2) atmosphere using glove-box techniques. The test tubes were then sealed with airtight electrical tapes and the reaction mixtures were stirred in a preheated oil-bath. Flash column chromatography was performed using silica gel (Silicycle, ultra pure grade). Preparative thin-layer chromatography (preparative TLC) was performed using preparative TLC plate (Merck Millipore, TLC Silica gel 60 F_{254}, 20 x 20 cm, catalogue number: 1.05715.0001) in a developing tank. Notably, the TLC plates used for the purification of amide products were washed with hexanes/triethylamine solution (volume ratio ~20:1) prior to the use in order to minimize the product loss. The eluents for column chromatography and preparative TLC were presented as ratios of solvent volumes. Yields reported in the publication are of isolated materials unless otherwise noted. All new starting materials and all amide products were characterized by ^{1}H and ^{13}C NMR spectroscopies and high-resolution mass spectrometry (HRMS).

Synthesis of Starting Materials:

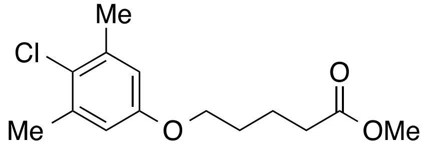

Methyl 5-(4-Chloro-3,5-dimethylphenoxy)pentanoate (S1). An oven-dried 100 mL round-bottom flask equipped with a Teflon-coated magnetic stir bar was sequentially charged with 4-chloro-3,5-dimethylphenol (1 equiv, 1.81 g, 11.6 mmol), DMF (anhydrous, 30 mL), and sodium hydride (60% in paraffin oil, 1.5 equiv, 696 mg, 17.4 mmol). The resulting mixture was stirred at room temperature for 30 min. Sodium iodide (40 mol %, 697 mg, 4.64 mmol) and methyl 5-chloropentanoate (1 equiv, 1.67 mL, 11.6 mmol) were then added, and the reaction mixture was stirred at 90 ^{o}C in a preheated oil bath overnight. After the reaction, the crude product was quenched with water (~20 mL) and then washed with saturated NaOH solution (~200 mL) and EtOAc (~100 mL). The organic fraction was concentrated *in vacuo* with the aid of a rotary evaporator, and the residue was purified by flash chromatography with silica gel (without prior washing with Et_3N/hexanes) using a mixture of hexanes/EtOAc (5:1) as an eluent to afford the title compound (**S1**) as yellow oil (1.72 g, 55%). **^{1}H NMR** (400 MHz, $CDCl_3$): δ 6.61 (s, 2 H), 3.90 (t, *J* = 6.0 Hz, 2 H), 3.66 (s, 3 H), 2.38 (t, *J* = 7.1 Hz, 2 H), 2.32 (s, 6 H), 1.81-1.76 (ovrlp, 4 H). **^{13}C NMR** (100 MHz, $CDCl_3$): δ 173.8, 156.8, 137.0, 126.1, 114.5, 67.5, 51.5, 33.7, 28.7, 21.7, 20.9. **HRMS** (ESI): Calcd for $C_{14}H_{20}ClO_3$ [M+H]: 271.1095; Found: 271.1097.

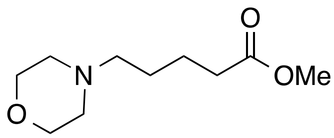

Methyl 5-Morpholinopentanoate (S2). An oven-dried 100 mL round-bottom flask equipped with a Teflon-coated magnetic stir bar was sequentially charged with morpholine (5 equiv, 8.6 mL, 99.5 mmol), methyl 5-chloropentanoate (1 equiv, 3.00 g, 19.9 mmol), sodium iodide (1 equiv, 1.50 g, 9.95 mmol), and DMF (anhydrous, 30 mL), and the reaction mixture was stirred at 90 ^{o}C in a preheated oil bath overnight. After the reaction, the crude product was washed with water (~300 mL) and EtOAc (~100 mL). The organic fraction was concentrated *in vacuo* with the aid of a rotary evaporator, and the residue was purified by flash chromatography with silica gel (without prior washing with Et_3N/hexanes) using a mixture of hexanes/EtOAc (3:1) as an eluent to afford the title compound (**S2**) as brown oil (1.23 g, 31%). **^{1}H NMR** (400 MHz, $CDCl_3$): δ 3.71 (t, *J* = 4.7 Hz, 4 H), 3.67 (s, 3 H), 2.42 (t, *J* = 4.2 Hz, 4 H), 2.36-2.32 (ovrlp, 4 H), 1.66 (qu, *J* = 7.8 Hz, 2 H), 1.52 (qu, *J* = 7.9 Hz, 2 H). **^{13}C NMR** (100 MHz, $CDCl_3$): δ 174.1, 67.1, 58.6, 53.8, 51.6, 34.0, 26.1, 22.9. **HRMS** (ESI): Calcd for $C_{10}H_{20}NO_3$ [M+H]: 202.1443; Found: 202.1448.

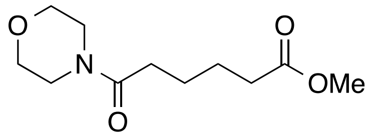

Methyl 6-Morpholino-6-oxohexanoate (S3). An oven-dried 250 mL round-bottom flask equipped with a Teflon-coated magnetic stir bar was sequentially charged with morpholine (1.2 equiv, 1.1 mL, 12.8 mmol), CH_2Cl_2 (100 mL), triethylamine (1.8 equiv, 2.7 mL, 19.3 mmol), *N*,*N*-dimethyl-4-aminopyridine (3 mol %, 0.321 mmol, 39 mg), and methyl 6-chloro-6-oxohexanoate (1 equiv, 1.91 g, 10.7 mmol), and the resulting mixture was stirred at room temperature overnight. After the reaction, the crude product was washed with dilute HCl solution (~1 M, ~100 mL). The aqueous fraction was removed, and the organic fraction was further neutralized with saturated $NaHCO_3$ solution (~100 mL). The organic fraction was concentrated *in vacuo* with the aid of a rotary evaporator, and the residue was purified by flash chromatography with silica gel (without prior washing with Et_3N/hexanes) using a mixture of hexanes/EtOAc (3:1) as an eluent to afford the title compound (**S3**) as brown oil (1.10 g, 45%). **^{1}H NMR** (400 MHz, $CDCl_3$): δ 3.68-3.65 (ovrlp, 7 H), 3.61 (t, *J* = 4.7 Hz, 2 H), 3.46 (t, *J* = 4.8 Hz, 2 H), 2.37-2.31 (ovrlp, 4 H), 1.72-1.65 (ovrlp, 4 H). **^{13}C NMR** (100 MHz, $CDCl_3$): δ 173.8, 171.1, 66.9, 66.6, 51.5, 45.9, 41.8, 33.7, 32.6, 24.6, 24.5. **HRMS** (ESI): Calcd for $C_{11}H_{20}NO_4$ [M+H]: 230.1387; Found: 230.1391.

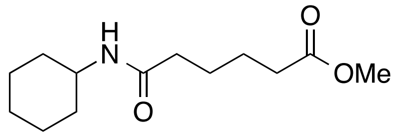

Methyl 6-(Cyclohexylamino)-6-oxohexanoate (S4). An oven-dried 250 mL round-bottom flask equipped with a Teflon-coated magnetic stir bar was sequentially charged with cyclohexylamine (2.5 equiv, 2.9 mL, 25.0 mmol), CH_2Cl_2 (100 mL), and methyl 6-chloro-6-oxohexanoate (1 equiv, 1.79 g, 10.0 mmol), and the resulting mixture was stirred at room temperature overnight. After the reaction, the crude product was washed with diluted HCl solution (~1 M, ~100 mL). The aqueous fraction was removed, and the organic fraction was further neutralized with saturated $NaHCO_3$ solution (~100 mL). The organic fraction was concentrated *in vacuo* with the aid of a rotary evaporator, and the residue was purified by recrystallized using CH_2Cl_2/hexanes as solvents to afford the title compound (**S4**) as white solid (1.93 g, 80%). **^{1}H NMR** (400 MHz, $CDCl_3$): δ 5.75 (s, 1 H), 3.80-3.71 (m, 1 H), 3.67 (s, 3 H), 2.34 (t, *J* = 6.6 Hz, 2 H), 2.17 (t, *J* = 6.6 Hz, 2 H), 1.90 (dd, *J* = 11.4 Hz, *J* = 2.5 Hz, 2 H), 1.74-1.59 (ovrlp, 7 H), 1.36 (qt, *J* = 12.2 Hz, *J* = 3.2 Hz, 2 H), 1.14 (qu/d, *J* = 12.2 Hz, *J* = 3.4 Hz, 2 H). **^{13}C NMR** (100 MHz, $CDCl_3$): δ 174.0, 171.6, 51.5, 48.1, 36.4, 33.7, 33.2, 25.6, 25.2, 24.9, 24.4. **HRMS** (ESI): Calcd for $C_{13}H_{24}NO_3$ [M+H]: 242.1756; Found: 242.1758.

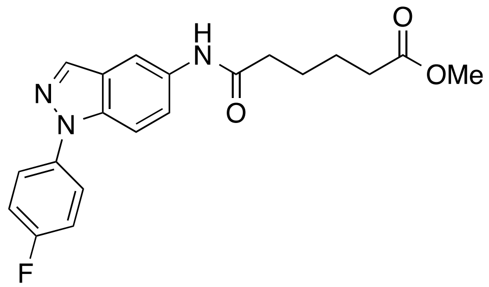

Methyl 6-((1-(4-Fluorophenyl)-1*H*-indazol-5-yl)amino)-6-oxohexanoate (S5). An oven-dried 250 mL round-bottom flask equipped with a Teflon-coated magnetic stir bar was sequentially charged with 1-(4-fluorophenyl)-1*H*-indazol-5-amine[17] (1 equiv, 327 mg, 1.44 mmol), CH_2Cl_2 (30 mL), triethylamine (1.5 equiv, 0.3 mL, 2.16 mmol), *N*,*N*-dimethyl-4-aminopyridine (10 mol %, 18 mg, 0.144 mmol), and methyl 6-chloro-6-oxohexanoate (1.1 equiv, 283 mg, 1.58 mmol), and the resulting mixture was stirred at room temperature overnight. After the reaction, the crude product was washed with dilute HCl solution (~1 M, ~30 mL). The aqueous fraction was removed, and the organic fraction was further neutralized with saturated $NaHCO_3$ solution (~50 mL). The organic fraction was concentrated *in vacuo* with the aid of a rotary evaporator, and the residue was purified by recrystallization using CH_2Cl_2/hexanes as solvents to afford the title compound (**S5**) as white solid (500 mg, 94%). **^{1}H NMR** (400 MHz, $CDCl_3$): δ 8.14-8.11 (ovrlp, 2 H), 7.68-7.58 (ovrlp, 3 H), 7.59 (d, J = 9.0 Hz, 1 H), 7.43 (dd, J = 9.0 Hz, J = 1.8 Hz, 1 H), 7.22 (dd, $^3J_{HH}$ = 8.5 Hz, $^3J_{HH}$ = 8.5 Hz, 1 H), 3.69 (s, 3 H), 2.45-2.38 (ovrlp, 4 H), 1.85-1.66 (ovrlp, 4 H). **^{13}C NMR** (100 MHz, $CDCl_3$): δ 174.3, 171.1, 161.3 (d, $^1J_{CF}$ = 245.0 Hz), 136.3, 135.5, 132.2, 125.5, 124.5 (d, $^3J_{CF}$ = 8.4 Hz), 121.9, 116.5 (d, $^2J_{CF}$ = 22.8 Hz), 112.2, 110.5, 51.8, 37.2, 33.7, 25.1, 24.4. **HRMS** (ESI): Calcd for $C_{20}H_{21}FN_3O_3$ [M+H]: 370.1567; Found: 370.1564.

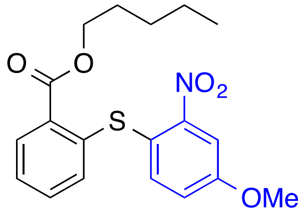

Pentyl 2-((4-Methoxy-2-nitrophenyl)thio)benzoate (S6). The title compound was synthesized according to the literature procedures with small variations.[18] In a nitrogen-filled glove box, an 30 mL Teflon-screw cap test tube equipped with a Teflon-coated magnetic stir bar was sequentially charged with 1-bromo-4-methoxy-2-nitrobenzene (1 equiv, 232 mg, 1.00 mmol), 2-mercaptobenzoic acid (1.2 equiv, 190 mg, 1.20 mmol), K_2CO_3 (1.5 equiv, 207 mg, 1.50 mmol), copper powder (20 mol %, 13 mg), and 1-pentanol (3 mL), and the resulting mixture was stirred at 140 ^oC in a preheated oil bath for 24 h. After the reaction, the crude product was washed with EtOAc (~100 mL) and saturated NaOH solution (~100 mL). The aqueous fraction was removed, and the organic fraction was further washed with water (~100 mL). The organic fraction was concentrated *in vacuo* with the aid of a rotary evaporator, and the residue was purified by flash chromatography with silica gel (without prior washing with Et_3N/hexanes) using a mixture of hexanes/EtOAc (4:1) as an eluent to afford the title compound (**S6**) as brown oil (296 mg, 75%). **^{1}H NMR** (400 MHz, $CDCl_3$): δ 7.94 (dd, J = 7.5 Hz, J = 1.0 Hz, 1 H), 7.55 (d, J = 2.6 Hz, 1 H), 7.39 (td, J = 7.4 Hz, J = 1.1 Hz, 1 H), 7.34 (d, J = 7.3 Hz, 1 H), 7.21 (d, J = 8.8 Hz, 1 H), 7.16 (d, J = 7.4 Hz, 1 H), 7.05 (dd, J = 8.8 Hz, J = 2.6 Hz, 1 H), 4.27 (t, J =

6.6 Hz, 2 H), 3.88 (s, 3 H), 1.68 (qu, J = 6.8 Hz, 2 H), 1.40-1.28 (ovrlp, 4 H), 0.90 (t, J = 6.8 Hz, 3 H). **^{13}C NMR** (100 MHz, $CDCl_3$): δ 166.8, 159.3, 150.5, 137.2, 135.4, 132.43, 132.41, 132.1, 131.0, 127.3, 123.7, 120.6, 109.5, 65.8, 56.1, 28.4, 28.3, 22.4, 14.1. **HRMS** (ESI): Calcd for $C_{19}H_{22}NO_5S$ [M+H]: 376.1213; Found: 376.1221.

Synthesis of Amides

Nickel-Catalyzed Reductive Coupling of Nitroarene with Alkyl Alkanoate (General Procedure A). An oven-dried 30 mL re-sealable screw-cap test tube equipped with a Teflon-coated magnetic stir bar was sequentially charged with zinc powder (Zn, 4 equiv, 2.0 mmol, 131 mg), ester (1 equiv, 0.50 mmol), nitroarene (1.2 equiv, 0.60 mmol), 1,10-phenanthroline (phen, 7.5 mol %, 6.8 mg), nickel(II) chloride ethylene glycol dimethyl ether complex (Ni(glyme)Cl_2, 7.5 mol %, 8.3 mg), *N*-methylpyrrolidone solvent (NMP, 1.0 mL), and chlorotrimethylsilane (TMSCl, 2 equiv, 1.0 mmol, 128 μL). The resulting mixture was stirred at 90 ^{o}C in a preheated oil bath for 16 h. After the reaction, the reaction mixture was cooled down to room temperature, and the crude product was acidified with saturated NH_4Cl solution (~5 mL) and then neutralized with saturated $NaHCO_3$ solution (~10 mL). The crude product in the aqueous fraction was extracted with EtOAc (~20 mL). The aqueous fraction was further washed with EtOAc (3 x ~10 mL). The combined organic fractions were concentrated *in vacuo* with the aid of a rotary evaporator. The crude product residue was purified by preparative thin-layer chromatography (TLC) using a solvent mixture (dichloromethane (CH_2Cl_2), hexanes, and/or ethyl acetate (EtOAc)) as an eluent to afford the purified amide product.

Nickel-Catalyzed Reductive Coupling of Nitroarene with Alkyl Arenoate (General Procedure B). An oven-dried 30 mL re-sealable screw-cap test tube equipped with a Teflon-coated magnetic stir bar was sequentially charged with zinc powder (Zn, 4 equiv, 2.0 mmol, 131 mg), ester (1 equiv, 0.50 mmol), nitroarene (1.3 equiv, 0.65 mmol), 1,10-phenanthroline (phen, 7.5 mol %, 6.8 mg), nickel(II) chloride ethylene glycol dimethyl ether complex (Ni(glyme)Cl_2, 7.5 mol %, 8.3 mg), *N*-methylpyrrolidone solvent (NMP, 1.0 mL), and chlorotrimethylsilane (TMSCl, 2 equiv, 1.0 mmol, 128 μL). The resulting mixture was stirred at 120 ^{o}C in a preheated oil bath for 16 h. After the reaction, the reaction mixture was cooled down to room temperature, and the crude product was acidified with saturated NH_4Cl solution (~5 mL) and then neutralized with saturated $NaHCO_3$ solution (~10 mL). The crude product in the aqueous fraction was extracted with EtOAc (~20 mL). The aqueous fraction was further washed with EtOAc (3 x ~10 mL). The combined organic fractions were concentrated *in vacuo* with the aid of a rotary evaporator. The crude product residue was purified by preparative thin-layer chromatography (TLC) using a solvent mixture (dichloromethane (CH_2Cl_2), hexanes, and/or ethyl acetate (EtOAc)) as an eluent to afford the purified amide product.

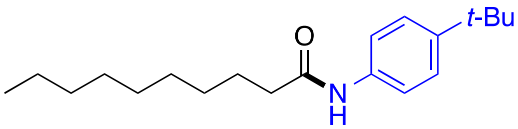

***N*-(4-(*tert*-Butyl)phenyl)decanamide (3a).**

(i) Synthesized from methyl decanoate: Following the general procedure A, the title compound was prepared using methyl decanoate (0.50 mmol, 93 mg) and 1-(*tert*-butyl)-4-nitrobenzene (0.60 mmol, 108 mg). The crude product was purified by preparative TLC using hexanes/CH_2Cl_2 (5:1) as an eluent to afford the title compound (**3a**) as pale brown solid (110 mg, 72%). **^{1}H NMR** (400 MHz, $CDCl_3$): δ 7.43 (d, *J* = 8.5 Hz, 2 H), 7.32 (d, *J* = 8.5 Hz, 2 H), 7.27 (s, 1 H), 2.33 (t, *J* = 7.6 Hz, 2 H), 1.72 (qu, *J* = 7.3 Hz, 2 H), 1.39-1.22 (ovrlp, 21 H), 0.88 (t, *J* = 7.0 Hz, 3 H). **^{13}C NMR** (100 MHz, $CDCl_3$): δ 171.5, 147.2, 135.5, 125.9, 119.8, 37.9, 34.5, 32.0, 31.5, 29.6, 29.5, 29.4, 25.9, 22.8, 14.2. **HRMS** (ESI): Calcd for $C_{20}H_{34}NO$ [M+H]: 304.2640; Found: 304.2646.

(ii) Synthesized from *tert*-butyl decanoate: Following the general procedure A, the title compound was prepared using *tert*-butyl decanoate (0.50 mmol, 114 mg), 1-(*tert*-butyl)-4-nit1robenzene (0.75 mmol, 134 mg), manganese (Mn, 5 equiv, 138 mg), iodotrimethylsilane (TMSI, 2 equiv, 143 μL), Ni(glyme)Cl_2 (15 mol %, 16.5 mg), and phen (15 mol %, 13.5 mg) at the reaction temperature of 140 ^{o}C. The crude product was purified by preparative TLC using hexanes/EtOAc (8:1) as an eluent to afford the title compound (**3a**) as pale brown solid (96 mg, 63%). Spectral and analytical data were identical to those reported for the same compound above.

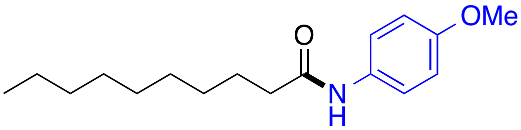

***N*-(4-Methoxyphenyl)decanamide (3b).** Following the general procedure A, the title compound was prepared using methyl decanoate (0.50 mmol, 93 mg) and 1-methoxy-4-nitrobenzene (0.60 mmol, 92 mg). The crude product was purified by preparative TLC using hexanes/CH_2Cl_2 (3:1) as an eluent to afford the title compound (**3b**) as pale brown solid (95 mg, 68%). **^{1}H NMR** (400 MHz, $CDCl_3$): δ 7.41 (d, *J* = 8.8 Hz, 2 H), 7.15 (s, 1 H), 6.84 (d, *J* = 8.7 Hz, 2 H), 3.78 (s, 3 H), 2.32 (t, *J* = 7.6 Hz, 2 H), 1.71 (qu, *J* = 7.3 Hz, 2 H), 1.39-1.21 (ovrlp, 12 H), 0.88 (t, *J* = 6.9 Hz, 3 H). **^{13}C NMR** (100 MHz, $CDCl_3$): δ 171.4, 156.5, 131.2, 121.9, 114.2, 55.6, 37.8, 32.0, 29.6, 29.5, 29.45, 29.42, 25.9, 22.8, 14.2. **HRMS** (ESI): Calcd for $C_{17}H_{28}NO_2$ [M+H]: 278.2120; Found: 278.2126.

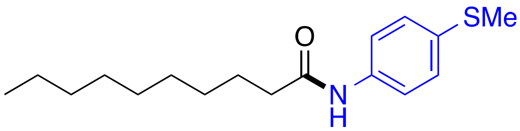

***N*-(4-(Methylthio)phenyl)decanamide (3c).** Following the general procedure A, the title compound was prepared using methyl decanoate (0.5 mmol, 93 mg) and methyl(4-nitrophenyl)sulfane (0.60 mmol, 102 mg). The crude product was purified by preparative TLC using hexanes/CH_2Cl_2 (4:1) as an eluent to afford the title compound (**3c**) as brown solid (98 mg, 67%). **^{1}H NMR** (400 MHz, $CDCl_3$): δ

7.45 (d, *J* = 8.4 Hz, 2 H), 7.23 (d, *J* = 8.6 Hz, 2 H), 7.12 (s, 1 H), 2.46 (s, 3 H), 2.34 (t, *J* = 7.6 Hz, 2 H), 1.72 (qu, *J* = 7.3 Hz, 2 H), 1.38-1.23 (ovrlp, 12 H), 0.88 (d, *J* = 7.0 Hz, 3 H). **^{13}C NMR** (100 MHz, $CDCl_3$): δ 171.4, 135.8, 133.5, 128.2, 120.5, 38.0, 32.0, 29.6, 29.5, 29.4, 25.8, 22.8, 16.9, 14.3. **HRMS** (ESI): Calcd for $C_{17}H_{28}NOS$ [M+H]: 294.1891; Found: 294.1892.

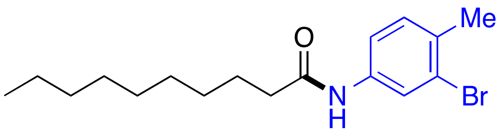

***N*-(3-Bromo-4-methylphenyl)decanamide (3d).** Following the general procedure A, the title compound was prepared using methyl decanoate (1 equiv, 0.50 mmol, 93 mg) and 2-bromo-1-methyl-4-nitrobenzene (1.5 equiv, 0.75 mmol, 163 mg). The crude product was purified by preparative TLC using hexanes/CH_2Cl_2 (5:1) as an eluent to afford the title compound (**3d**) as deep brown solid (130 mg, 68%). **^{1}H NMR** (400 MHz, $CDCl_3$): δ 7.76 (s, 1 H), 7.45 (s, 1 H), 7.34 (d, *J* = 7.7 Hz, 1 H), 7.13 (d, *J* = 8.2 Hz, 1 H), 2.36-2.29 (ovrlp, 5 H), 1.68 (qu, *J* = 7.3 Hz, 2 H), 1.36-1.21 (ovrlp, 12 H), 0.87 (t, *J* = 7.0 Hz, 3 H). **^{13}C NMR** (100 MHz, $CDCl_3$): δ 171.8, 136.9, 133.6, 130.8, 124.8, 123.7, 119.0, 37.8, 32.0, 29.6, 29.5, 29.4, 25.7, 22.8, 22.4, 14.2. **HRMS** (ESI): Calcd for $C_{17}H_{27}BrNO$ [M+H]: 340.1278; Found: 340.1260.

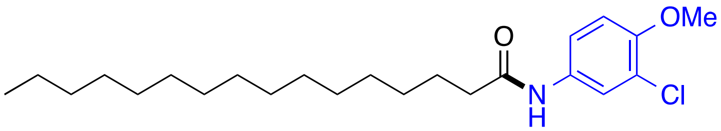

***N*-(3-Chloro-4-methoxyphenyl)palmitamide (3e).** Following the general procedure A, the title compound was prepared using hexadecyl palmitate (1 equiv, 0.50 mmol, 240 mg), 2-chloro-1-methoxy-4-nitrobenzene (1.5 equiv, 0.75 mmol, 141 mg), Ni(glyme)Cl_2 (15 mol %, 16.5 mg) and phen (15 mol %, 13.5 mg). The crude product was purified by preparative TLC using hexanes/CH_2Cl_2 (3:1) as an eluent to afford the title compound (**3e**) as brown solid (105 mg, 53%). **^{1}H NMR** (400 MHz, $CDCl_3$): δ 7.55 (d, *J* = 2.6 Hz, 1 H), 7.38 (dd, *J* = 8.8 Hz, *J* = 2.6 Hz, 1 H), 7.30 (s, 1 H), 6.85 (d, *J* = 8.8 Hz, 1 H), 3.87 (s, 3 H), 2.32 (t, *J* = 7.6 Hz, 2 H), 1.70 (qu, *J* = 7.7 Hz, 2 H), 1.37-1.22 (ovrlp, 24 H), 0.88 (t, *J* = 6.9 Hz, 3 H). **^{13}C NMR** (100 MHz, $CDCl_3$): δ 171.6, 151.9, 131.7, 122.64, 122.56, 119.8, 112.3, 56.5, 37.7, 32.1, 29.84, 29.82, 29.80, 29.76, 29.6, 29.52, 29.50, 29.4, 25.8, 22.8, 14.3. **HRMS** (ESI): Calcd for $C_{23}H_{39}ClNO_2$ [M+H]: 396.2669; Found: 396.2666.

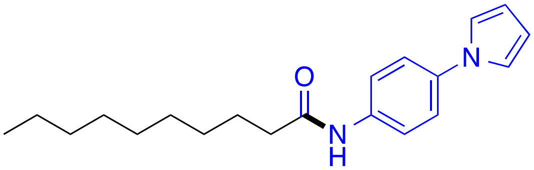

***N*-(4-(1*H*-Pyrrol-1-yl)phenyl)decanamide (3f).** Following the general procedure A, the title compound was prepared using methyl decanoate (0.50 mmol, 93 mg) and 1-(4-nitrophenyl)-1*H*-

pyrrole (0.60 mmol, 113 mg). The crude product was purified by preparative TLC using hexanes/CH_2Cl_2 (5:1) as an eluent to afford the title compound (**3f**) as brown solid (80 mg, 51%). **^{1}H NMR** (400 MHz, $CDCl_3$): δ 7.57 (d, *J* = 8.3 Hz, 2 H), 7.38 (s, 1 H), 7.32 (d, *J* = 8.4 Hz, 2 H), 7.03 (s, 2 H), 6.33 (s, 2 H), 2.36 (t, *J* = 7.6 Hz, 2 H), 1.73 (qu, *J* = 2 Hz, 2 H), 1.39-1.26 (ovrlp, 12 H), 0.88 (t, *J* = 7.1 Hz, 3 H). **^{13}C NMR** (100 MHz, $CDCl_3$): δ 171.7, 137.1, 135.8, 121.14, 121.07, 119.4, 110.4, 37.9, 32.0, 29.6, 29.5, 29.43, 29.41, 25.8, 22.8, 14.2. **HRMS** (ESI): Calcd for $C_{20}H_{29}N_2O$ [M+H]: 313.2286; Found: 313.2274.

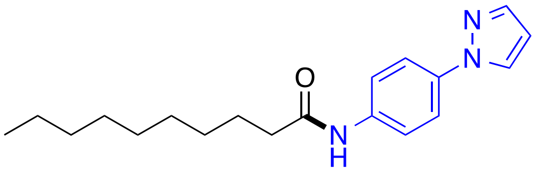

***N*-(4-(1*H*-Pyrazol-1-yl)phenyl)decanamide (3g).** Following the general procedure A, the title compound was prepared using methyl decanoate (1 equiv, 0.50 mmol, 93 mg), 1-(4-nitrophenyl)-1*H*-pyrazole (1.5 equiv, 0.75 mmol, 142 mg), Ni(glyme)Cl_2 (10 mol %, 11.0 mg), and phen (10 mol %, 9.0 mg) under the reaction temperature of 120 ^oC. The crude product was purified by preparative TLC using hexanes/CH_2Cl_2 (4:1) as an eluent to afford the title compound (**3g**) as pale brown solid (78 mg, 50%). **^{1}H NMR** (400 MHz, $CDCl_3$): δ 7.88 (d, *J* = 2.4 Hz, 1 H), 7.71 (s, 1 H), 7.65-7.60 (ovrlp, 4 H), 7.22 (s, 1 H), 6.46 (s, 1 H), 2.37 (t, *J* = 7.6 Hz, 2 H), 1.74 (qu, *J* = 7.6 Hz, 2 H), 1.43-1.22 (ovrlp, 12 H), 0.88 (t, *J* = 7.0 Hz, 3 H). **^{13}C NMR** (100 MHz, $CDCl_3$): δ 171.5, 141.0, 136.6, 136.5, 126.8, 120.7, 120.0, 107.7, 38.0, 32.0, 29.6, 29.5, 29.43, 29.42, 25.7, 22.8, 14.3. **HRMS** (ESI): Calcd for $C_{19}H_{28}N_3O$ [M+H]: 314.2237; Found: 314.2217.

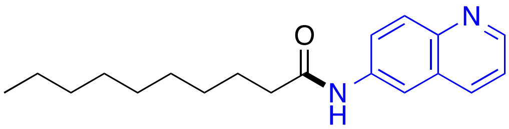

***N*-(Quinolin-6-yl)decanamide (3h).** Following the general procedure A, the title compound was prepared using methyl decanoate (1 equiv, 0.50 mmol, 93 mg), 6-nitroquinoline (1.5 equiv, 0.75 mmol, 131 mg), Ni(glyme)Cl_2 (10 mol %, 11.0 mg), and phen (10 mol %, 9.0 mg). The crude product was purified by preparative TLC using hexanes/CH_2Cl_2 (1:1) as an eluent to afford the title compound (**3h**) as pale brown solid (105 mg, 70%). **^{1}H NMR** (400 MHz, $CDCl_3$): δ 8.81 (s, 1 H), 8.70 (s, 1 H), 8.45 (s, 1 H), 8.05 (d, *J* = 8.4 Hz, 1 H), 7.98 (d, *J* = 9.0 Hz, 1 H), 7.61 (d, *J* = 9.1 Hz, 1 H), 7.34 (dd, *J* = 8.4 Hz, *J* = 4.3 Hz, 1 H), 2.42 (t, *J* = 7.6 Hz, 2 H), 1.74 (qu, *J* = 7.7 Hz, 2 H), 1.36-1.17 (ovrlp, 12 H), 0.86 (t, *J* = 7.1 Hz, 3 H). **^{13}C NMR** (100 MHz, $CDCl_3$): δ 172.5, 149.2 145.4, 136.5, 136.1, 129.8, 129.0, 123.5, 121.7, 116.2, 37.8, 31.9, 29.50, 29.47, 29.39, 29.3, 25.8, 22.7, 14.2. **HRMS** (ESI): Calcd for $C_{19}H_{27}N_2O$ [M+H]: 299.2128; Found: 299.2118.

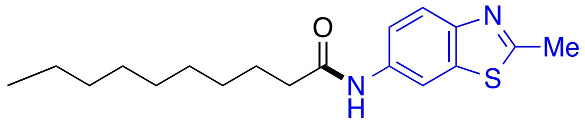

***N*-(2-Methylbenzo[*d*]thiazol-6-yl)decanamide (3i).** Following the general procedure A, the title compound was prepared using methyl decanoate (1 equiv, 0.50 mmol, 93 mg) and 2-methyl-6-nitrobenzo[*d*]thiazole (1.5 equiv, 0.75 mmol, 146 mg), Ni(glyme)Cl_2 (15 mol %, 16.5 mg), and phen (15 mol %, 13.5 mg) under the reaction temperature of 120 ^{o}C. The crude product was purified by preparative TLC using hexanes/CH_2Cl_2 (1:1) as an eluent to afford the title compound (**3i**) as deep brown solid (80 mg, 50%). ^{1}**H NMR** (400 MHz, $CDCl_3$): δ 8.43 (s, 1 H), 7.83 (d, *J* = 8.7 Hz, 1 H), 7.39 (s, 1 H), 7.23 (dd, *J* = 8.7 Hz, *J* = 2.2 Hz, 1 H), 2.80 (s, 3 H), 2.38 (t, *J* = 7.6 Hz, 2 H), 1.74 (qu, *J* = 7.5 Hz, 2 H), 7.40-1.17 (ovrlp, 12 H), 0.88 (t, *J* = 6.9 Hz, 3 H). ^{13}**C NMR** (100 MHz, $CDCl_3$): δ 171.7, 166.6, 150.1, 136.8, 135.1, 122.4, 118.6, 112.5, 38.0, 32.0, 29.6, 29.5, 29.422, 29.415, 25.8, 22.8, 20.2, 14.2. **HRMS** (ESI): Calcd for $C_{18}H_{27}N_2OS$ [M+H]: 319.1844; Found: 319.1842.

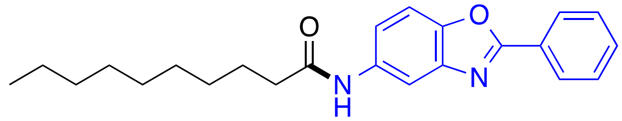

***N*-(2-Phenylbenzo[*d*]oxazol-5-yl)decanamide (3j).** Following the general procedure A, the title compound was prepared using methyl decanoate (0.50 mmol, 93 mg) and 5-nitro-2-phenylbenzo[*d*]oxazole (0.60 mmol, 144 mg). The crude product was purified by preparative TLC using hexanes/CH_2Cl_2 (1:1) as an eluent to afford the title compound (**3j**) as deep brown solid (100 mg, 55%). ^{1}**H NMR** (400 MHz, $CDCl_3$): δ 8.20 (d, *J* = 6.8 Hz, 2 H), 7.90 (s, 1 H), 7.77 (s, 1 H), 7.56-7.45 (ovrlp, 5 H), 2.36 (t, *J* = 7.6 Hz, 2 H), 1.72 (qu, *J* = 7.5 Hz, 2 H), 1.36-1.24 (ovrlp, 12 H), 0.86 (d, *J* = 7.0 Hz, 3 H). ^{13}**C NMR** (100 MHz, $CDCl_3$): δ 171.9, 163.9, 147.6, 142.5, 135.2, 131.7, 129.0, 127.7, 127.1, 118.5, 111.7, 110.5, 37.8, 32.0, 29.6, 29.53, 29.46, 29.4, 25.8, 22.8, 14.2. **HRMS** (ESI): Calcd for $C_{23}H_{29}N_2O_2$ [M+H]: 365.2229; Found: 365.2227.

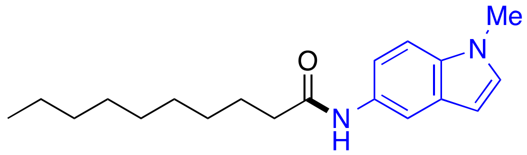

***N*-(1-Methyl-1*H*-indol-5-yl)decanamide (3k).** Following the general procedure A, the title compound was prepared using methyl decanoate (0.50 mmol, 93 mg) and 1-methyl-5-nitro-1*H*-indole (0.60 mmol, 106 mg). The crude product was purified by preparative TLC using hexanes/CH_2Cl_2 (3:1) as an eluent to afford the title compound (**3k**) as pale brown solid (88 mg, 59%). ^{1}**H NMR** (400 MHz, $CDCl_3$): δ 7.81 (s, 1 H), 7.28-7.24 (ovrlp, 2 H), 7.21 (s, 1 H), 7.03 (d, *J* = 3.1 Hz, 1 H), 6.42 (d, *J* = 3.1 Hz, 1 H), 3.76 (s, 3 H), 2.35 (t, *J* = 7.6 Hz, 2 H), 1.74 (qu, *J* = 7.6 Hz, 2 H), 1.39-1.19 (ovrlp, 12 H), 0.88 (t, *J* = 7.0 Hz, 3 H). ^{13}**C NMR** (100 MHz, $CDCl_3$): δ 171.5, 134.3, 130.3, 129.7, 128.6, 116.0, 112.9, 109.4, 101.1, 38.0, 33.1, 32.0, 29.62. 29.57, 29.5, 29.4, 26.0, 22.8, 14.3. **HRMS** (ESI): Calcd for $C_{19}H_{29}N_2O$ [M+H]: 301.2280; Found: 301.2285.

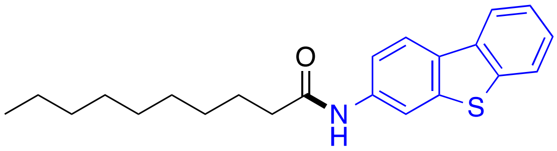

***N*-(Dibenzo[*b*,*d*]thiophen-3-yl)decanamide (3l).** Following the general procedure A, the title compound was prepared using methyl decanoate (0.50 mmol, 93 mg) and 3-nitrodibenzo[*b*,*d*]thiophene (0.60 mmol, 149 mg). The crude product was purified by preparative TLC using hexanes/CH_2Cl_2 (4:1) as an eluent to afford the title compound (**3l**) as off-white solid (140 mg, 79%). **^{1}H NMR** (400 MHz, $CDCl_3$): δ 8.47 (s, 1 H), 8.00 (d, *J* = 7.7 Hz, 1 H), 7.81-7.76 (ovrlp, 2 H), 7.66 (d, *J* = 8.5 Hz, 1 H), 7.42-7.32 (ovrlp, 3 H), 2.37 (t, *J* = 7.7 Hz, 2 H), 1.73 (qu, *J* = 7.0 Hz, 2 H), 1.36-1.19 (ovrlp, 12 H), 0.87 (t, *J* = 6.2 Hz, 3 H). **^{13}C NMR** (100 MHz, $CDCl_3$): δ 172.1, 140.2, 136.2, 135.4, 135.2, 134.9, 126.9, 124.4, 122.95, 122.85, 121.9, 119.8, 113.2, 37.9, 32.0, 29.59, 29.55, 29.5, 29.4, 25.8, 22.8, 14.2. **HRMS** (ESI): Calcd for $C_{22}H_{28}NOS$ [M+H]: 354.1900; Found: 354.1885.

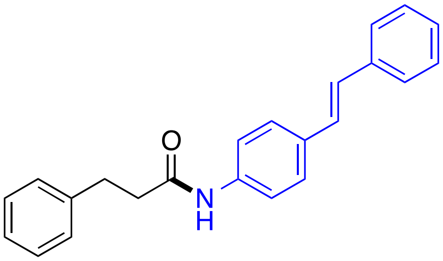

(*E*)-3-Phenyl-*N*-(4-styrylphenyl)propanamide (3m). Following the general procedure A, the title compound was prepared using methyl 3-phenylpropanoate (0.50 mmol, 82 mg) and (*E*)-1-nitro-4-styrylbenzene (0.60 mmol, 135 mg). The crude product was purified by preparative TLC using hexanes/CH_2Cl_2 (3:1) as an eluent to afford the title compound (**3m**) as pale brown solid (125 mg, 76%). **^{1}H NMR** (400 MHz, DMSO-d_6): δ 10.00 (s, 1 H), 7.62-7.52 (ovrlp, 6 H), 7.36 (t, *J* = 7.6 Hz, 2 H), 7.31-7.23 (ovrlp, 5 H), 7.21-7.12 (ovrlp, 3 H), 2.92 (t, *J* = 7.8 Hz, 2 H), 2.64 (t, *J* = 7.6 Hz, 2 H). **^{13}C NMR** (100 MHz, DMSO-d_6): δ 170.4, 141.2, 138.8, 137.2, 131.9, 128.7, 128.34, 128.26, 128.04, 127.4, 126.93, 126.90, 126.3, 126.0, 119.1, 38.0, 30.8. **HRMS** (ESI): Calcd for $C_{23}H_{22}NO$ [M+H]: 328.1710; Found: 328.1686.

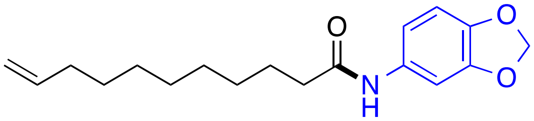

***N*-(Benzo[*d*][1,3]dioxol-5-yl)undec-10-enamide (3n).** Following the general procedure A, the title compound was prepared using methyl undec-10-enoate (0.50 mmol, 106 mg) and 5-nitrobenzo[*d*][1,3]dioxole (0.60 mmol, 100 mg). The crude product was purified by preparative TLC using hexanes/CH_2Cl_2 (3:1) as an eluent to afford the title compound (**3n**) as brown solid (91 mg, 60%). **^{1}H NMR** (400 MHz, $CDCl_3$): δ 7.57 (s, 1 H), 7.21 (s, 1 H), 6.79 (d, *J* = 8.4 Hz, 1 H), 6.70 (d, *J*

= 8.3 Hz, 1 H), 5.92 (2 H), 5.85-5.75 (m, 1 H), 5.01-4.91 (ovrlp, 2 H), 2.30 (t, *J* = 7.6 Hz, 2 H), 2.03 (q, *J* = 7.2 Hz, 2 H), 1.69 (qu, *J* = 7.6 Hz, 2 H), 1.38-1.24 (ovrlp, 10 H). ^{13}**C NMR** (100 MHz, $CDCl_3$): δ 171.8, 147.7, 144.2, 139.2, 132.4, 114.2, 113.4, 108.0, 103.1, 101.2, 37.6, 33.8, 29.42. 29.41, 29.36, 29.2, 29.0, 25.8. **HRMS** (ESI): Calcd for $C_{18}H_{26}NO_3$ [M+H]: 304.1913; Found: 304.1913.

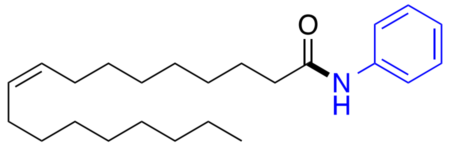

***N*-Phenyloleamide (3o).** Following the general procedure A, the title compound was prepared using methyl oleate (0.50 mmol, 148 mg) and nitrobenzene (0.60 mmol, 62 μL). The crude product was purified by preparative TLC using hexanes/CH_2Cl_2 (5:1) as an eluent to afford the title compound (**3o**) as brown solid (147 mg, 82%). ^{1}**H NMR** (400 MHz, $CDCl_3$): δ 7.51 (d, *J* = 8.0 Hz, 2 H), 7.48 (s, 1 H), 7.29 (t, *J* = 7.8 Hz, 2 H), 7.08 (t, *J* = 7.4 Hz, 1 H), 5.39-5.30 (ovrlp, 2 H), 2.03-1.95 (ovrlp, 4 H), 1.71 (qu, *J* = 7.5 Hz, 2 H), 1.38-1.22 (ovrlp, 20 H), 0.88 (t, *J* = 6.9 Hz, 3 H). ^{13}**C NMR** (100 MHz, $CDCl_3$): δ 171.9, 138.2, 130.1, 129.8, 128.9, 124.2, 120.1, 37.8, 32.0, 29.9, 29.8, 29.6, 29.41, 29.36, 29.2, 27.31, 27.27, 25.8, 22.8, 14.2. **HRMS** (ESI): Calcd for $C_{24}H_{40}NO$ [M+H]: 358.3104; Found: 358.3093.

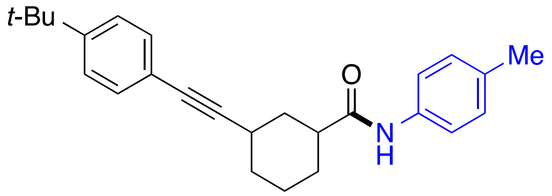

3-((4-(*tert*-Butyl)phenyl)ethynyl)-*N*-(*p*-tolyl)cyclohexane-1-carboxamide (3p). Following the general procedure A, the title compound was prepared using methyl 3-((4-(*tert*-butyl)phenyl)ethynyl)cyclohexane-1-carboxylate (diastereomeric ratio (d.r.) ~ 4.4:1, 1 equiv, 0.63 mmol, 196 mg) and 4-nitrotoluene (1.5 equiv, 0.95 mmol, 130 mg), Ni(glyme)Cl_2 (10 mol %, 13.9 mg), and phen (10 mol %, 11.3 mg), Zn (4 equiv, 2.52 mmol, 165 mg), TMSCl (2 equiv, 1.26 mmol, 161 μL), and NMP (1.3 mL) under the reaction temperature of 120 ^{o}C. The crude product was purified by preparative TLC using hexanes/CH_2Cl_2 (5:1) as an eluent to afford the title compound (**3p**) as pale brown solid (118 mg, 50%). The diastereomeric ratio of the product was determined to be ~4:1 by GC analysis. ^{1}**H NMR** (400 MHz, $CDCl_3$): δ 7.40 (d, *J* = 8.3 Hz, 2 H), 7.34-7.25 (ovrlp, 5 H), 7.10 (d, *J* = 8.0 Hz, 2 H), 2.52-2.43 (m, 1 H), 2.33-2.21 (ovrlp, 4 H), 2.05 (d, *J* = 12.6 Hz, 1 H), 1.97-1.85 (ovrlp, 2 H), 1.76-1.34 (ovrlp, 5 H), 1.29 (s, 9 H). ^{13}**C NMR** (100 MHz, $CDCl_3$): δ 173.3, 150.9, 135.5, 134.0, 131.4, 129.6, 125.3, 120.8, 120.1, 92.4, 80.9, 45.9, 35.9, 34.8, 32.5, 31.3, 30.0, 28.9, 25.2, 21.0. **HRMS** (ESI): Calcd for $C_{26}H_{32}NO$ [M+H]: 374.2484; Found: 374.2487.

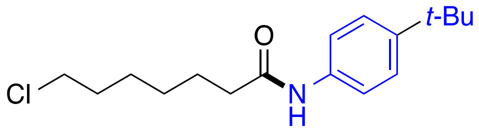

***N*-(4-(*tert*-Butyl)phenyl)-7-chloroheptanamide (3q).** Following the general procedure A, the title compound was prepared using methyl 7-chloroheptanoate (0.50 mmol, 89 mg) and 1-(*tert*-butyl)-4-nitrobenzene (0.60 mmol, 107 mg). The crude product was purified by preparative TLC using hexanes/CH_2Cl_2 (5:1) as an eluent to afford the title compound (**3q**) as viscous, deep brown oil (99 mg, 67%). **^{1}H NMR** (400 MHz, $CDCl_3$): δ 7.51 (s, 1 H), 7.44 (d, *J* = 8.2 Hz, 2 H), 7.31 (d, *J* = 8.2 Hz, 2 H), 3.51 (t, *J* = 6.7 Hz, 2 H), 2.34 (t, *J* = 7.6 Hz, 2 H), 1.80-1.69 (ovrlp, 4 H), 1.48-1.36 (ovrlp, 4 H), 1.29 (s, 9 H). **^{13}C NMR** (100 MHz, $CDCl_3$): δ 171.4, 147.2, 135.5, 125.8, 119.8, 45.1, 37.5, 34.4, 32.5, 31.5, 28.5, 26.8, 25.6. **HRMS** (ESI): Calcd for $C_{17}H_{27}ClNO$ [M+H]: 296.1776; Found: 296.1767.

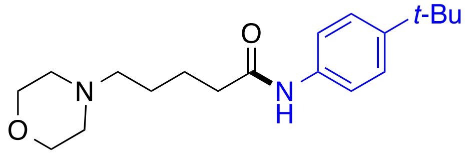

***N*-(4-(*tert*-Butyl)phenyl)-5-morpholinopentanamide (3r).** Following the general procedure A, the title compound was prepared using methyl 5-morpholinopentanoate (**S2**, 1 equiv, 0.50 mmol, 101 mg), 1-(*tert*-butyl)-4-nitrobenzene (1.5 equiv, 0.75 mmol, 134 mg), Ni(glyme)Cl_2 (10 mol %, 11.0 mg), and phen (10 mol %, 9.0 mg) under the reaction temperature of 120 ^{o}C. The crude product was purified by preparative TLC using hexanes/CH_2Cl_2 (1:4) as an eluent to afford the title compound (**3r**) as brown solid (109 mg, 68%). **^{1}H NMR** (400 MHz, $CDCl_3$): δ 7.77 (s, 1 H), 7.43 (d, *J* = 8.3 Hz, 2 H), 7.31 (d, *J* = 8.2 Hz, 2 H), 3.69 (t, *J* = 4.8 Hz, 4 H), 2.41 (t, *J* = 4.5 Hz, 4 H), 2.38-2.33 (ovrlp, 4 H), 1.74 (qu, *J* = 7.6 Hz, 2 H), 1.55 (qu, *J* = 7.7 Hz, 2 H), 1.29 (s, 9 H). **^{13}C NMR** (100 MHz, $CDCl_3$): δ 171.3, 147.2, 135.5, 125.8, 119.8, 67.0, 58.6, 53.8, 37.3, 34.4, 31.4, 26.1, 23.6. **HRMS** (ESI): Calcd for $C_{19}H_{31}N_2O_2$ [M+H]: 319.2386; Found: 319.2386.

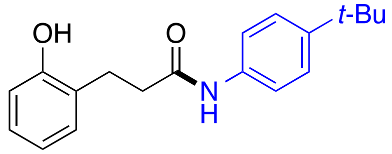

***N*-(4-(*tert*-Butyl)phenyl)-3-(2-hydroxyphenyl)propanamide (3s).** Following the general procedure A, the title compound was prepared using methyl 3-(2-hydroxyphenyl)propanoate (1 equiv, 0.50 mmol, 90 mg), 1-(*tert*-butyl)-4-nitrobenzene (1.3 equiv, 0.65 mmol, 116 mg), Ni(glyme)Cl_2 (10 mol %, 11.0 mg), and phen (10 mol %, 9.0 mg) under the temperature of 140 ^{o}C. The crude product was purified by preparative TLC using hexanes/CH_2Cl_2 (1:1) as an eluent to afford the title compound (**3s**) as pale brown solid (62 mg, 42%). **^{1}H NMR** (400 MHz, $CDCl_3$): δ 8.43 (br s, 1 H), 7.35 (d, *J* = 8.7 Hz, 2 H), 7.32-7.28 (ovrlp, 3 H), 7.13-7.07 (ovrlp, 2 H), 6.90 (d, *J* = 7.7 Hz, 1 H), 6.84 (t, *J* = 7.4 Hz, 1 H), 2.98 (t, *J* = 6.2 Hz, 2 H), 2.78 (t, *J* = 6.2 Hz, 2 H), 1.28 (s, 9 H). **^{13}C NMR** (100 MHz, $CDCl_3$): δ 172.6, 154.9, 148.0, 134.5, 130.7, 128.3, 127.8, 126.0, 120.7, 120.3, 118.0, 38.3, 34.5, 31.5, 24.7. **HRMS** (ESI): Calcd for $C_{19}H_{24}NO_2$ [M+H]: 298.1802; Found: 298.1806.

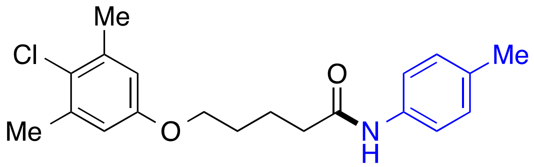

5-(4-Chloro-3,5-dimethylphenoxy)-*N*-(*p*-tolyl)pentanamide (3t). Following the general procedure A, the title compound was prepared methyl 5-(4-chloro-3,5-dimethylphenoxy)pentanoate (**S1**, 0.50 mmol, 135 mg) and 4-nitrotoluene (0.60 mmol, 82 mg). The crude product was purified by preparative TLC using hexanes/CH_2Cl_2 (5:1) as an eluent to afford the title compound (**3t**) as pale brown solid (108 mg, 62%). ^{1}**H NMR** (400 MHz, $CDCl_3$): δ 7.70 (s, 1 H), 7.37 (d, *J* = 8.0 Hz, 2 H), 7.07 (d, *J* = 8.0 Hz, 2 H), 6.59 (s, 2 H), 3.89 (t, *J* = 5.7 Hz, 2 H), 2.38 (t, *J* = 6.8 Hz, 2 H), 2.31 (s, 6 H), 2.28 (s, 3 H), 1.89-1.77 (ovrlp, 4 H). ^{13}**C NMR** (100 MHz, $CDCl_3$): δ 171.2, 156.8, 137.1, 135.5, 133.9, 129.5, 126.2, 120.2, 114.5, 67.7, 37.1, 28.7, 22.5, 21.0, 20.9. **HRMS** (ESI): Calcd for $C_{20}H_{25}ClNO_2$ [M+H]: 346.1579; Found: 346.1571.

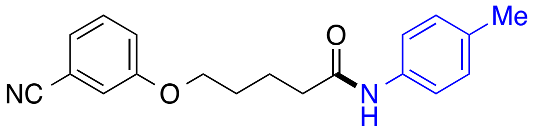

5-(3-Cyanophenoxy)-*N*-(*p*-tolyl)pentanamide (3u). Following the general procedure A, the title compound was prepared using methyl 5-(3-cyanophenoxy)pentanoate (1 equiv, 0.50 mmol, 117 mg), 4-nitrotoluene (1.5 equiv, 0.75 mmol, 103 mg), Ni(glyme)Cl_2 (15 mol %, 16.6 mg), and phen (15 mol %, 13.6 mg) under the reaction temperature of 140 ^{o}C. The crude product was purified by preparative TLC using hexanes/CH_2Cl_2 (1:3) as an eluent to afford the title compound (**3u**) as deep brown solid (63 mg, 41%). ^{1}**H NMR** (400 MHz, $CDCl_3$): δ 7.38 (d, *J* = 8.3 Hz, 2 H), 7.34 (d, *J* = 9.0 Hz, 1 H), 7.22 (d, *J* = 7.6 Hz, 2 H), 7.15-7.06 (ovrlp, 4 H), 4.00 (t, *J* = 5.2 Hz, 2 H), 2.43 (t, *J* = 6.4 Hz, 2 H), 2.31 (s, 3 H), 1.96-1.83 (ovrlp, 4 H). ^{13}**C NMR** (100 MHz, $CDCl_3$): δ 170.7, 159.1, 135.4, 134.1, 130.5, 129.6, 124.6, 120.0, 119.9, 118.9, 117.5, 113.3, 68.1, 37.2, 28.7, 22.3, 21.0. **HRMS** (ESI): Calcd for $C_{19}H_{21}N_2O_2$ [M+H]: 309.1608; Found: 309.1598.

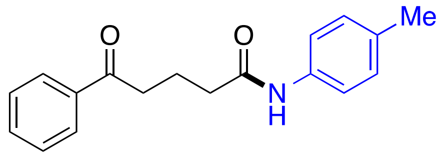

5-Oxo-5-phenyl-*N*-(*p*-tolyl)pentanamide (3v). Following the general procedure A, the title compound was prepared using methyl 5-oxo-5-phenylpentanoate (1 equiv, 0.50 mmol, 103 mg), 4-nitrotoluene (1.5 equiv, 0.75 mmol, 103 mg), Ni(glyme)Cl_2 (10 mol %, 11.0 mg), and phen (10 mol %, 9.0 mg) under the reaction temperature of 120 ^{o}C. The crude product was purified by preparative TLC using hexanes/CH_2Cl_2 (3:1) as an eluent to afford the title compound (**3v**) as brown solid (115 mg, 82%). ^{1}**H NMR** (400 MHz, $CDCl_3$): δ 7.94 (d, *J* = 7.1 Hz, 2 H), 7.82 (s, 1 H), 7.54 (t, *J* = 7.4 Hz, 1 H),

7.45-7.39 (ovrlp, 4 H), 7.08 (d, *J* = 8.0 Hz, 2 H), 3.08 (t, *J* = 6.8 Hz, 2 H), 2.44 (t, *J* = 7.1 Hz, 2 H), 2.28 (s, 3 H), 2.14 (qu, *J* = 7.2 Hz, 2 H). **^{13}C NMR** (100 MHz, $CDCl_3$): δ 200.3, 171.0, 136.8, 135.5, 133.8, 133.3, 129.5, 128.7, 128.2, 120.1, 37.5, 26.5, 20.9, 20.3. **HRMS** (ESI): Calcd for $C_{18}H_{20}NO_2$ [M+H]: 282.1497; Found: 282.1488.

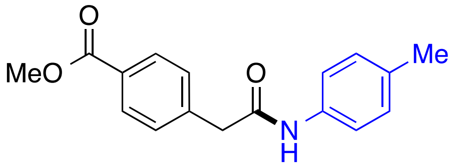

Methyl 4-(2-Oxo-2-(*p*-tolylamino)ethyl)benzoate (3w). Following the general procedure A, the title compound was prepared using methyl 4-(2-methoxy-2-oxoethyl)benzoate (0.50 mmol, 104 mg) and 4-nitrotoluene (0.60 mmol, 82 mg). The crude product was purified by preparative TLC using hexanes/CH_2Cl_2 (3:1) as an eluent to afford the title compound (**3w**) as pale-brown solid (112 mg, 79%). **^{1}H NMR** (400 MHz, $CDCl_3$): δ 8.01 (d, *J* = 8.2 Hz, 2 H), 7.47 (s, 1 H), 7.37 (d, *J* = 7.9 Hz, 2 H), 7.30 (d, *J* = 8.2 Hz, 2 H), 7.06 (d, *J* = 8.0 Hz, 2 H), 3.91 (s, 3 H), 3.71 (s, 2 H), 2.28 (s, 3 H). **^{13}C NMR** (100 MHz, $CDCl_3$): δ 168.3, 166.9, 140.0, 135.1, 134.4, 130.3, 129.6, 129.4, 120.2, 52.3, 44.6, 21.0. **HRMS** (ESI): Calcd for $C_{17}H_{18}NO_3$ [M+H]: 284.1289; Found: 284.1272.

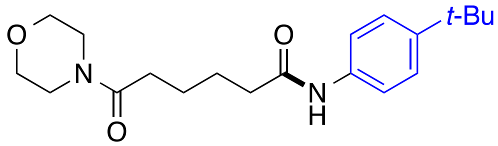

***N*-(4-(*tert*-Butyl)phenyl)-6-morpholino-6-oxohexanamide (3x).** Following the general procedure A, the title compound was prepared using methyl 6-morpholino-6-oxohexanoate (**S3**, 1 equiv, 0.50 mmol, 115 mg), 1-(*tert*-butyl)-4-nitrobenzene (1.5 equiv, 0.75 mmol, 135 mg), Ni(glyme)Cl_2 (10 mol %, 11.0 mg), and phen (10 mol %, 9.0 mg) under the temperature of 120 ^{o}C. The crude product was purified by preparative TLC using hexanes/EtOAc (2:1) as an eluent to afford the title compound (**3x**) as viscous, deep brown oil (89 mg, 51%). **^{1}H NMR** (400 MHz, $CDCl_3$): δ 8.46 (s, 1 H), 7.50 (d, *J* = 8.7 Hz, 2 H), 7.30 (d, *J* = 8.7 Hz, 2 H), 3.66-3.60 (ovrlp, 6 H), 3.44 (t, *J* = 4.8 Hz, 2 H), 2.42-2.35 (ovrlp, 4 H), 1.77-1.67 (m, 4 H), 1.29 (s, 9 H). **^{13}C NMR** (100 MHz, $CDCl_3$): δ 171.6, 171.3, 146.9, 135.8, 125.7, 119.8, 66.9, 66.6, 46.0, 42.0, 37.1, 34.4, 32.6, 31.4, 25.2, 24.4. **HRMS** (ESI): Calcd for $C_{20}H_{31}N_2O_3$ [M+H]: 347.2335; Found: 347.2330.

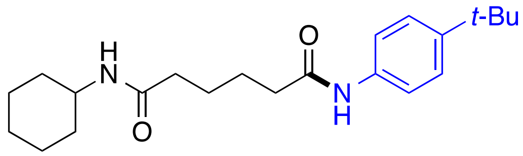

***N*^{1}-(4-(*tert*-Butyl)phenyl)-*N*^{6}-cyclohexyladipamide (3y).** Following the general procedure A, the title compound was prepared using methyl 6-(cyclohexylamino)-6-oxohexanoate (**S4**, 1 equiv, 0.50 mmol,

121 mg) and 1-(*tert*-butyl)-4-nitrobenzene (1.2 equiv, 0.60 mmol, 108 mg), Ni(glyme)Cl_2 (10 mol %, 11.0 mg), and phen (10 mol %, 9.0 mg). The crude product was purified by recrystallization using CH_2Cl_2/hexanes as solvents to afford the title compound (**3y**) as white solid (138 mg, 77%). ^{1}**H NMR** (400 MHz, $CDCl_3$): δ 8.21 (s, 1 H), 7.49 (d, *J* = 8.5 Hz, 2 H), 7.31 (d, *J* = 8.5 Hz, 2 H), 5.73 (d, *J* = 8.2 Hz, 1 H), 3.79-3.71 (m, 1 H), 2.39 (t, *J* = 5.5 Hz, 2 H), 2.21 (d, *J* = 5.9 Hz, 2 H), 1.89 (d, *J* = 11.9 Hz, 2 H), 1.79-1.63 (ovrlp, 6 H), 1.62-1.57 (m, 1 H), 1.37-1.25 (ovrlp, 11 H), 1.21-1.07 (ovrlp, 3 H). ^{13}**C NMR** (100 MHz, $CDCl_3$): δ 172.1, 171.3, 147.1, 135.7, 125.8, 119.8, 48.4, 37.2, 36.5, 34.5, 33.3, 31.5, 25.7, 25.2, 25.0, 24.9. **HRMS** (ESI): Calcd for $C_{22}H_{35}N_2O_2$ [M+H]: 359.2707; Found: 359.2680.

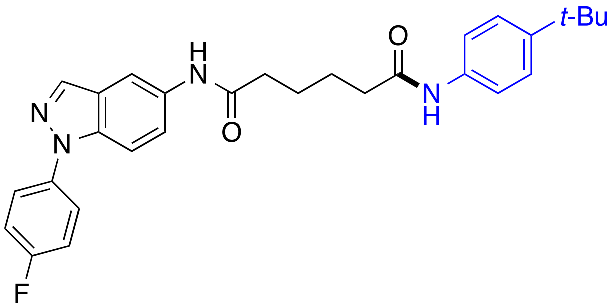

***N*^{1}-(4-(*tert*-Butyl)phenyl)-*N*^{6}-(1-(4-fluorophenyl)-1*H*-indazol-5-yl)adipamide (3z).** Following the general procedure A, the title compound was prepared using methyl 6-((1-(4-fluorophenyl)-1*H*-indazol-5-yl)amino)-6-oxohexanoate (**S5**, 1 equiv, 0.50 mmol, 185 mg) and 1-(*tert*-butyl)-4-nitrobenzene (1.5 equiv, 0.75 mmol, 134 mg), Ni(glyme)Cl_2 (15 mol %, 16.5 mg), and phen (15 mol %, 13.5 mg) under the reaction temperature of 120 ^{o}C. The crude product was purified by recrystallization using CH_2Cl_2/hexanes as solvents to afford the title compound (**3z**) as off-white solid (112 mg, 46%). ^{1}**H NMR** (400 MHz, DMSO-d_6): δ 10.04 (s, 1 H), 9.81 (s, 1 H), 8.31 (s, 1 H), 8.28 (d, *J* = 1.4 Hz, 1 H), 7.81-7.74 (ovrlp, 3 H), 7.56 (dd, *J* = 9.1 Hz, *J* = 1.8 Hz, 1 H), 7.50 (d, *J* = 8.7 Hz, 2 H), 7.41 (dd, $^{3}J_{HH}$ = 8.8 Hz, $^{3}J_{HF}$ = 8.8 Hz, 2 H), 7.28 (d, *J* = 8.7 Hz, 2 H), 2.40-2.32 (ovrlp, 4 H), 1.70-1.62 (ovrlp, 4 H), 1.23 (s, 9 H). ^{13}**C NMR** (100 MHz, DMSO-d_6): δ 171.1, 170.9, 160.2 (d, $^{1}J_{CF}$ = 242.0 Hz), 145.2, 136.7, 136.2 (d, $^{4}J_{CF}$ = 2.8 Hz), 135.6, 134.9, 133.7, 125.2, 125.1, 123.8 (d, $^{3}J_{CF}$ = 8.5 Hz), 121.3, 118.9, 116.4 (d, $^{2}J_{CF}$ = 22.8 Hz), 110.4, 110.1, 36.32, 36.27, 33.9, 31.2, 25.0. **HRMS** (ESI): Calcd for $C_{29}H_{32}FN_4O_2$ [M+H]: 487.2504; Found: 487.2509.

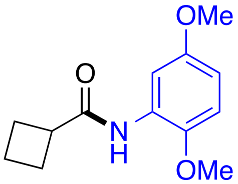

***N*-(2,5-Dimethoxyphenyl)cyclobutanecarboxamide (4a).** Following the general procedure A, the title compound was prepared using ethyl cyclobutanecarboxylate (1 equiv, 0.50 mmol, 64 mg) and 1,4-dimethoxy-2-nitrobenzene (1.5 equiv, 0.75 mmol, 137 mg), Ni(glyme)Cl_2 (10 mol %, 11.0 mg), and phen (10 mol %, 9.0 mg) under the reaction temperature of 120 ^{o}C. The crude product was purified by preparative TLC using hexanes/CH_2Cl_2 (1:1) as an eluent to afford the title compound (**4a**) as viscous brown oil (78 mg, 66%). ^{1}**H NMR** (400 MHz, $CDCl_3$): δ 8.18 (d, *J* = 3.1 Hz, 1 H), 7.71 (s,

1 H), 6.77 (d, *J* = 8.9 Hz, 1 H), 6.55 (dd, *J* = 8.8 Hz, *J* = 3.0 Hz, 1 H), 3.83 (s, 3 H), 3.78 (s, 3 H), 3.20 (qu, *J* = 8.5 Hz, 1 H), 2.44-2.35 (m, 2 H), 2.28-2.20 (m, 2 H), 2.04-1.91 (m, 2 H). ^{13}**C NMR** (100 MHz, $CDCl_3$): δ 173.3, 154.0, 142.0, 128.5, 110.8, 108.6, 105.7, 56.3, 55.9, 41.3, 25.5, 18.2. **HRMS** (ESI): Calcd for $C_{13}H_{18}NO_3$ [M+H]: 236.1287; Found: 236.1287.

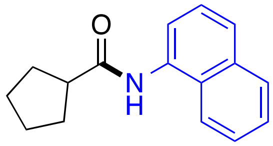

***N*-(Naphthalen-1-yl)cyclopentanecarboxamide (4b).** Following the general procedure A, the title compound was prepared using methyl cyclopentanecarboxylate (1 equiv, 0.50 mmol, 64 mg) and 1-nitronaphthalene (1.5 equiv, 0.75 mmol, 130 mg), Ni(glyme)Cl_2 (10 mol %, 11.0 mg), and phen (10 mol %, 9.0 mg) under the reaction temperature of 120 ^{o}C. The crude product was purified by preparative TLC using hexanes/CH_2Cl_2 (5:1) as an eluent to afford the title compound (**4b**) as deep brown solid (71 mg, 59%). ^{1}**H NMR** (400 MHz, $CDCl_3$): δ 7.96 (d, *J* = 7.5 Hz, 1 H), 7.87 (d, *J* = 7.7 Hz, 1 H), 7.82 (d, *J* = 8.0 Hz, 1 H), 7.69 (d, *J* = 8.2 Hz, 1 H), 7.53-7.45 (ovrlp, 4 H), 2.88 (qu, *J* = 8.2 Hz, 1 H), 2.10-2.01 (ovrlp, 4 H), 1.98-1.97 (m, 2 H), 1.74-1.63 (m, 2 H). ^{13}**C NMR** (100 MHz, $CDCl_3$): δ 175.0, 134.3, 132.6, 129.0, 127.2, 126.4, 126.0, 125.9, 125.7, 120.9, 120.5, 47.0, 30.8, 26.2. **HRMS** (ESI): Calcd for $C_{16}H_{18}NO$ [M+H]: 240.1400; Found: 240.1376.

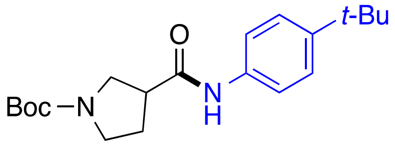

***tert*-Butyl 3-((4-(*tert*-butyl)phenyl)carbamoyl)pyrrolidine-1-carboxylate (4c).** Following the general procedure A, the title compound was prepared using 1-(*tert*-butyl) 3-methyl pyrrolidine-1,3-dicarboxylate (1 equiv, 0.50 mmol, 115 mg) and 1-(*tert*-butyl)-4-nitrobenzene (1.3 equiv, 0.65 mmol, 116 mg) under the reaction temperature of 120 ^{o}C. The crude product was purified by preparative TLC using hexanes/CH_2Cl_2 (1:3) as an eluent to afford the title compound (**4c**) as viscous deep brown oil (84 mg, 48%). ^{1}**H NMR** (400 MHz, $CDCl_3$): δ 8.21 (s, 1 H), 7.45 (d, *J* = 8.4 Hz, 2 H), 7.30 (d, *J* = 8.2 Hz, 2 H), 3.70-3.51 (ovrlp, 3 H), 3.35-3.28 (m, 1 H), 3.01 (br s, 1 H), 2.29-1.99 (ovrlp, 2 H), 1.46 (s, 9 H), 1.28 (s, 9 H). ^{13}**C NMR** (100 MHz, $CDCl_3$): δ 170.8, 154.5, 147.4, 135.4, 125.8, 119.9, 119.8, 79.7, 48.9, 45.60(44.65), 34.4, 31.4, 29.5(29.2), 28.6. **HRMS** (ESI): Calcd for $C_{20}H_{31}N_2O_3$ [M+H]: 369.2154; Found: 369.2156.

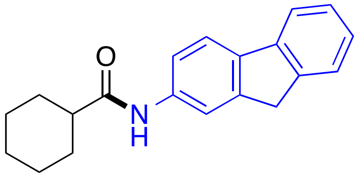

***N*-(9*H*-Fluoren-2-yl)cyclohexanecarboxamide (4d).** Following the general procedure A, the title compound was prepared using methyl cyclohexanecarboxylate (1 equiv, 0.50 mmol, 71 mg) and 2-nitro-9*H*-fluorene (1.3 equiv, 0.65 mmol, 137 mg) under the temperature of 120 °C. The crude product was purified by preparative TLC using hexanes/CH_2Cl_2 (5:1) as an eluent to afford the title compound (**4d**) as pale brown solid (90 mg, 62%). ^{1}**H NMR** (400 MHz, $CDCl_3$): δ 7.94 (s, 1 H), 7.72-7.68 (ovrlp, 2 H), 7.51 (d, J = 7.4 Hz, 1 H), 7.37-7.33 (ovrlp, 2 H), 7.28-7.25 (ovrlp, 2 H), 3.88 (s, 2 H), 2.28-2.21 (m, 1 H), 1.99 (d, J = 13.0 Hz, 2 H), 1.85 (d, J = 12.0 Hz, 2 H), 1.73-1.52 (ovrlp, 3 H), 1.35-1.28 (ovrlp, 3 H). ^{13}**C NMR** (100 MHz, $CDCl_3$): δ 174.4, 144.5, 143.3, 141.5, 138.0, 137.1, 126.9, 126.4, 125.1, 120.2, 119.6, 118.5, 116.9, 46.8, 37.2, 29.9, 25.8. **HRMS** (ESI): Calcd for $C_{20}H_{22}NO$ [M+H]: 292.1701; Found: 292.1702.

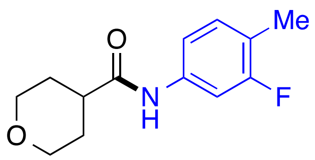

***N*-(3-Fluoro-4-methylphenyl)tetrahydro-2*H*-pyran-4-carboxamide (4e).** Following the general procedure A, the title compound was prepared using methyl tetrahydro-2*H*-pyran-4-carboxylate (1 equiv, 0.50 mmol, 72 mg) and 2-fluoro-1-methyl-4-nitrobenzene (1.3 equiv, 0.65 mmol, 101 mg) under the reaction temperature of 120 °C. The crude product was purified by preparative TLC using hexanes/CH_2Cl_2 (3:1) as an eluent to afford the title compound (**4e**) as brown solid (59 mg, 50%). ^{1}**H NMR** (400 MHz, $CDCl_3$): δ 7.67 (s, 1 H), 7.40 (d, J = 11.5 Hz, 1 H), 7.10-7.06 (ovrlp, 2 H), 4.04 (d, J = 8.2 Hz, 2 H), 3.41 (t, J = 11.5 Hz, 2 H), 2.53-2.44 (m, 1 H), 2.21 (s, 3 H), 1.98-1.76 (ovrlp, 4 H). ^{13}**C NMR** (100 MHz, $CDCl_3$): δ 172.9, 161.1 (d, $^1J_{CF}$ = 242.2 Hz), 137.0 (d, $^3J_{CF}$ = 10.6 Hz), 131.4 (d, $^3J_{CF}$ = 8.5 Hz), 120.7 (d, $^2J_{CF}$ = 17.5 Hz), 115.2 (d, $^4J_{CF}$ = 2.8 Hz), 107.5 (d, $^2J_{CF}$ = 26.9 Hz), 67.2, 43.2, 29.3, 14.2, 14.1. **HRMS** (ESI): Calcd for $C_{13}H_{16}FNO_2$ [M+H]: 238.1251; Found: 238.1230.

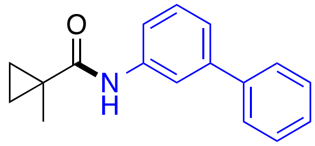

***N*-([1,1'-Biphenyl]-3-yl)-1-methylcyclopropane-1-carboxamide (4f).** Following the general procedure A, the title compound was prepared using ethyl 1-methylcyclopropane-1-carboxylate (1 equiv, 0.35 mmol, 45 mg), 3-nitro-1,1'-biphenyl (1.3 equiv, 0.455 mmol, 91 mg), Ni(glyme)Cl_2 (10 mol %, 7.7 mg), and phen (10 mol %, 6.3 mg), Zn (4 equiv, 1.4 mmol, 92 mg), TMSCl (2 equiv, 0.70 mmol, 89 μL), and NMP (0.70 mL) at 120 °C. The crude product was purified by preparative TLC using hexanes/CH_2Cl_2 (5:1) as an eluent to afford the title compound (**4f**) as pale brown solid (61 mg, 69%). ^{1}**H NMR** (400 MHz, $CDCl_3$): δ 7.79 (t, J = 1.9 Hz, 1 H), 7.60 (d, J = 1.6 Hz, 1 H), 7.58 (s, 1 H), 7.53 (s, 1 H), 7.48 (dt, J = 7.8 Hz, J = 1.4 Hz, 1 H), 7.44-7.38 (ovrlp, 3 H), 7.36-7.31 (ovrlp, 2 H), 1.49 (s, 3 H), 1.33 (q, J = 2.8 Hz, 2 H), 0.69 (q, J = 2.8 Hz, 2 H). ^{13}**C NMR** (100 MHz, $CDCl_3$): δ 173.4, 142.2, 140.8, 138.6, 129.4, 128.8, 127.6, 127.3, 123.1, 118.9, 20.0, 16.8. **HRMS** (ESI): Calcd for $C_{17}H_{18}NO$ [M+H]: 252.1390; Found: 252.1375.

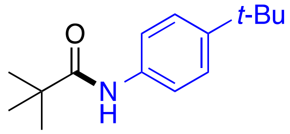

***N*-(4-(*tert*-butyl)phenyl)pivalamide (4g).**[19] Following the general procedure A, the title compound was prepared using ethyl pivalate (0.50 mmol, 1 equiv, 65 mg), 1-(*tert*-butyl)-4-nitrobenzene (0.75 mmol, 1.5 equiv, 134 mg), manganese (Mn, 5 equiv, 138 mg), iodotrimethylsilane (TMSI, 2 equiv, 143 μL), Ni(glyme)Cl_2 (15 mol %, 16.5 mg), and phen (15 mol %, 13.5 mg) at the reaction temperature of 140 ^{o}C. The crude product was purified by preparative TLC using hexanes/EtOAc (8:1) as an eluent to afford the title compound (**4g**) as pale brown solid (73 mg, 62%). **^{1}H NMR** (400 MHz, CD_2Cl_2): 7.43 (d, *J* = 8.2 Hz, 2 H), 7.36-7.30 (ovrlp, 3 H), 1.30 (s, 9 H), 1.28 (s, 9 H). **^{13}C NMR** (100 MHz, CD_2Cl_2): 176.7, 147.5, 136.2, 126.1, 120.3, 39.8, 34.7, 31.5, 27.8.

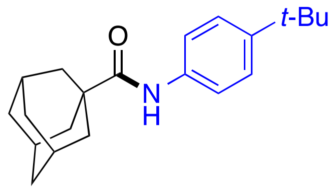

***N*-(4-(*tert*-butyl)phenyl)adamantane-1-carboxamide (4h).** Following the general procedure A, the title compound was prepared using ethyl adamantane-1-carboxylate (0.50 mmol, 1 equiv, 104 mg), 1-(*tert*-butyl)-4-nitrobenzene (0.75 mmol, 1.5 equiv, 134 mg), manganese (Mn, 5 equiv, 138 mg), iodotrimethylsilane (TMSI, 2 equiv, 143 μL), Ni(glyme)Cl_2 (15 mol %, 16.5 mg), and phen (15 mol %, 13.5 mg) at the reaction temperature of 140 ^{o}C. The crude product was purified by preparative TLC using hexanes/EtOAc (8:1) as an eluent to afford the title compound (**4h**) as pale brown solid (136 mg, 87%). **^{1}H NMR** (400 MHz, CD_2Cl_2): 7.46 (d, *J* = 8.6 Hz, 2 H), 7.38 (s, 1 H), 7.34 (d, *J* = 8.6 Hz, 2 H), 2.09-2.05 (m, 3 H), 1.96-1.93 (m, 6 H), 1.80-1.72 (m, 6 H), 1.31 (s, 9 H). **^{13}C NMR** (100 MHz, CD_2Cl_2): 176.2, 147.4, 136.2, 126.0, 120.3, 41.8, 39.7, 36.9, 34.6, 31.5, 28.8. **HRMS** (ESI): Calcd for $C_{21}H_{30}NO$ [M+H]: 312.2327; Found: 312.2327.

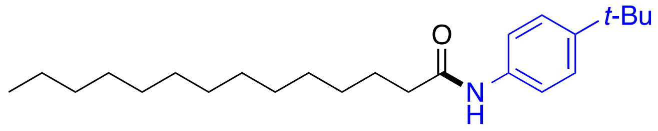

***N*-(4-(*tert*-butyl)phenyl)tetradecanamide (4i).** Following the general procedure A, the title compound was prepared using isopropyl tetradecanoate (0.50 mmol, 135 mg), 1-(*tert*-butyl)-4-nitrobenzene (0.75 mmol, 134 mg), manganese (Mn, 5 equiv, 138 mg), iodotrimethylsilane (TMSI, 2 equiv, 143 μL), Ni(glyme)Cl_2 (15 mol %, 16.5 mg), and phen (15 mol %, 13.5 mg) at the reaction temperature of 140 ^{o}C. The crude product was purified by preparative TLC using hexanes/EtOAc (8:1) as an eluent to afford the title compound (**4i**) as pale brown solid (154 mg, 86%). **^{1}H NMR** (400 MHz, CD_2Cl_2): 8.14 (s, 1 H), 7.49 (d, *J* = 7.6 Hz, 2 H), 7.32 (d, *J* = 7.6 Hz, 2 H), 2.35 (t, *J* = 6.4 Hz, 2 H), 1.71 (d, *J* = 6.6 Hz, 2 H), 1.37-1.25 (ovrlp, 29 H), 0.92 (d, *J* = 6.4 Hz, 3 H). **^{13}C NMR** (100 MHz, CD_2Cl_2): 172.3, 147.3, 136.3, 126.0, 120.3, 38.0, 34.7, 32.4, 31.6, 30.20, 30.18, 30.1, 30.0, 29.9, 29.8, 26.3, 23.2, 14.4. **HRMS** (ESI): Calcd for $C_{24}H_{42}NO$ [M+H]: 360.3262; Found: 360.3266.

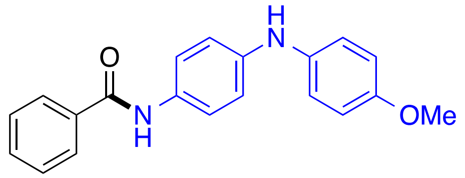

***N*-(4-((4-Methoxyphenyl)amino)phenyl)benzamide (5a).** Following the general procedure B, the title compound was prepared using methyl benzoate (1 equiv, 0.50 mmol, 68 mg) and 4-methoxy-*N*-(4-nitrophenyl)aniline (1.2 equiv, 0.60 mmol, 147 mg). The crude product was purified by preparative TLC using hexanes/CH_2Cl_2 (3:1) as an eluent to afford the title compound (**5a**) as deep purple solid (105 mg, 66%). ^{1}**H NMR** (400 MHz, $CDCl_3$): δ 7.86 (d, *J* = 7.4 Hz, 2 H), 7.71 (s, 1 H), 7.55-7.44 (ovrlp, 5 H), 7.05 (d, *J* = 8.4 Hz, 2 H), 6.93 (d, *J* = 8.4 Hz, 2 H), 6.86 (d, *J* = 8.3 Hz, 2 H), 5.49 (s, 1 H), 3.80 (s, 3 H). ^{13}**C NMR** (100 MHz, $CDCl_3$): δ 165.5, 155.2, 142.1, 136.0, 135.1, 131.6, 130.2, 128.7, 126.9, 122.1, 121.7, 116.5, 114.7, 55.6. **HRMS** (ESI): Calcd for $C_{20}H_{19}N_2O_2$ [M+H]: 319.1447; Found: 319.1444.

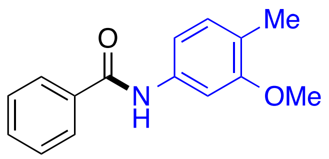

***N*-(3-Methoxy-4-methylphenyl)benzamide (5b).**

(i) Synthesized from benzyl benzoate: Following the general procedure B, the title compound was prepared using benzyl benzoate (0.50 mmol, 106 mg) and 2-methoxy-1-methyl-4-nitrobenzene (0.60 mmol, 109 mg). The crude product was purified by preparative TLC using hexanes/CH_2Cl_2 (3:1) as an eluent to afford the title compound (**5b**) as deep-brown solid (105 mg, 87%). ^{1}**H NMR** (400 MHz, $CDCl_3$): δ 8.22 (s, 1 H), 7.83 (d, *J* = 7.6 Hz, 2 H), 7.50-7.44 (ovrlp, 2 H), 7.39 (t, *J* = 7.5 Hz, 2 H), 7.03 (d, *J* = 8.0 Hz, 1 H), 6.93 (d, *J* = 7.4 Hz, 1 H), 3.76 (s, 3 H), 2.17 (s, 3 H). ^{13}**C NMR** (100 MHz, $CDCl_3$): δ 166.0, 158.0, 137.1, 135.1, 131.7, 130.5, 128.7, 127.1, 122.9, 111.9, 103.3, 55.3, 15.9. **HRMS** (ESI): Calcd for $C_{15}H_{16}NO_2$ [M+H]: 242.1181; Found: 242.1185.

(ii) Synthesized from phenyl benzoate: Following the general procedure B, the title compound was prepared using phenyl benzoate (0.50 mmol, 99 mg) and 2-methoxy-1-methyl-4-nitrobenzene (0.60 mmol, 109 mg). The crude product was purified by preparative TLC using hexanes/EtOAc (6:1) as an eluent to afford the title compound (**5b**) as deep-brown solid (91 mg, 76%). Spectral and analytical data were identical to those reported for the same compound above.

(iii) Synthesized from 2-naphthyl benzoate: Following the general procedure B, the title compound was prepared using 2-naphthyl benzoate (0.50 mmol, 124 mg) and 2-methoxy-1-methyl-4-nitrobenzene (0.60 mmol, 109 mg). The crude product was purified by preparative TLC using hexanes/EtOAc (6:1) as an eluent to afford the title compound (**5b**) as deep-brown solid (89 mg, 74%). Spectral and analytical data were identical to those reported for the same compound above.

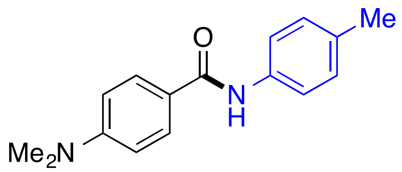

4-(Dimethylamino)-*N*-(*p*-tolyl)benzamide (5c). Following the general procedure B, the title compound was prepared using ethyl 4-(dimethylamino)benzoate (1 equiv, 0.50 mmol, 97 mg), 4-nitrotoluene (1.5 equiv, 0.75 mmol, 103 mg), Ni(glyme)Cl_2 (15 mol %, 16.5 mg), and phen (15 mol %, 13.5 mg). The crude product was purified by preparative TLC using hexanes/ EtOAc (4:1) as an eluent to afford the title compound (**5c**) as deep purple solid (64 mg, 50%). **^{1}H NMR** (400 MHz, $CDCl_3$): δ 7.86 (d, *J* = 7.4 Hz, 2 H), 7.71 (s, 1 H), 7.55-7.44 (ovrlp, 5 H), 7.05 (d, *J* = 8.4 Hz, 2 H), 6.93 (d, *J* = 8.4 Hz, 2 H), 6.86 (d, *J* = 8.3 Hz, 2 H), 5.49 (s, 1 H), 3.80 (s, 3 H). **^{13}C NMR** (100 MHz, $CDCl_3$): δ 165.5, 155.2, 142.1, 136.0, 135.1, 131.6, 130.2, 128.7, 126.9, 122.1, 121.7, 116.5, 114.7, 55.6. **HRMS** (ESI): Calcd for $C_{20}H_{19}N_2O_2$ [M+H]: 319.1447; Found: 319.1444.

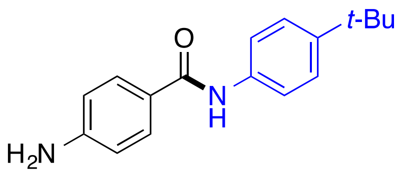

4-Amino-*N*-(4-(*tert*-butyl)phenyl)benzamide (5d). Following the general procedure B, the title compound was prepared using methyl 4-aminobenzoate (1 equiv, 0.50 mmol, 76 mg), 1-(*tert*-butylmethyl)-4-nitrobenzene (1.3 equiv, 0.65 mmol, 117 mg), Ni(glyme)Cl_2 (15 mol %, 16.5 mg), and phen (15 mol %, 13.5 mg). The crude product was purified by preparative TLC using hexanes/ EtOAc (1:1) as an eluent to afford the title compound (**5d**) as deep-brown solid (70 mg, 52%). **^{1}H NMR** (400 MHz, $CDCl_3$): δ 7.76 (s, 1 H), 7.69 (d, *J* = 8.2 Hz, 2 H), 7.53 (d, *J* = 8.3 Hz, 2 H), 7.35 (d, *J* = 8.2 Hz, 2 H), 6.67 (d, *J* = 8.1 Hz, 2 H), 4.20 (br s, 2 H), 1.31 (s, 9 H). **^{13}C NMR** (100 MHz, $CDCl_3$): δ 165.6, 150.0, 147.2, 135.8, 129.0, 125.9, 124.5, 120.1, 114.3, 34.5, 31.5. **HRMS** (ESI): Calcd for $C_{17}H_{21}N_2O$ [M+H]: 269.1654; Found: 269.1660.

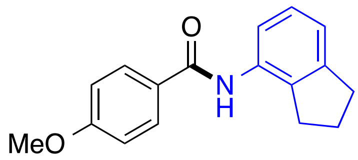

***N*-(2,3-Dihydro-1*H*-inden-4-yl)-4-methoxybenzamide (5e).** Following the general procedure B, the title compound was prepared using methyl 4-methoxybenzoate (0.50 mmol, 83 mg) and 4-nitro-2,3-dihydro-1*H*-indene (0.65 mmol, 106 mg). The crude product was purified by preparative TLC using hexanes/CH_2Cl_2 (3:1) as an eluent to afford the title compound (**5e**) as pale brown solid (106 mg, 79%). **^{1}H NMR** (400 MHz, $CDCl_3$): δ 7.85-7.81 (ovrlp, 3 H), 7.64 (s, 1 H), 7.18 (t, *J* = 7.6 Hz, 1 H), 7.04 (d, *J* = 7.2 Hz, 1 H), 6.96 (d, *J* = 8.4 Hz, 2 H), 3.85 (s, 3 H), 2.96 (t, *J* = 7.2 Hz, 2 H), 2.87 (t, *J* =

7.2 Hz, 2 H), 2.12 (qu, *J* = Hz, 2 H). **^{13}C NMR** (100 MHz, $CDCl_3$): δ 165.1, 162.5, 145.4, 134.4, 134.3, 129.0, 127.4, 127.3, 120.9, 119.0, 114.1, 55.6, 33.3, 30.2, 24.9. **HRMS** (ESI): Calcd for $C_{17}H_{18}NO_2$ [M+H]: 268.1338; Found: 268.1340.

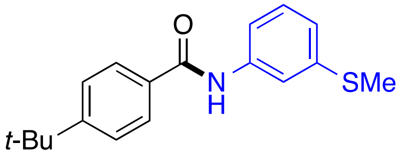

4-(*tert*-Butyl)-*N*-(3-(methylthio)phenyl)benzamide (5f). Following the general procedure B, the title compound was prepared using methyl 4-(*tert*-butyl)benzoate (1 equiv, 0.50 mmol, 96 mg) and methyl(3-nitrophenyl)sulfane (1.2 equiv, 0.60 mmol, 102 mg). The crude product was purified by preparative TLC using hexanes/CH_2Cl_2 (4:1) as an eluent to afford the title compound (**5f**) as brown solid (124 mg, 83%). **^{1}H NMR** (400 MHz, $CDCl_3$): δ 8.41 (s, 1 H), 7.77 (d, *J* = 8.2 Hz, 2 H), 7.66 (t, *J* = 2.0 Hz, 1 H), 7.38-7.36 (ovrlp, 3 H), 7.17 (t, *J* = 8.0 Hz, 1 H), 6.97 (d, *J* = 7.5 Hz, 1 H), 2.40 (s, 3 H), 1.30 (s, 9 H). **^{13}C NMR** (100 MHz, $CDCl_3$): δ 166.1, 155.4, 139.5, 138.8, 131.8, 129.2, 127.1, 125.6, 122.4, 118.0, 117.1, 35.0, 31.2, 15.6. **HRMS** (ESI): Calcd for $C_{18}H_{22}NOS$ [M+H]: 300.1422; Found: 300.1425.

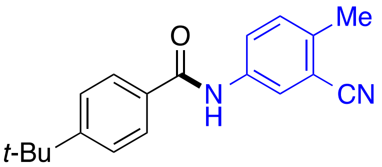

4-(*tert*-Butyl)-*N*-(3-cyano-4-methylphenyl)benzamide (5g). Following the general procedure B, the title compound was prepared using methyl 4-(*tert*-butyl)benzoate (1 equiv, 0.50 mmol, 96 mg), 2-methyl-5-nitrobenzonitrile (1.5 equiv, 0.75 mmol, 122 mg), Ni(glyme)Cl_2 (10 mol %, 11.0 mg), and phen (10 mol %, 9.0 mg). The crude product was purified by preparative TLC using hexanes/CH_2Cl_2 (1:1) as an eluent to afford the title compound (**5g**) as pale-brown solid (88 mg, 60%). **^{1}H NMR** (400 MHz, $CDCl_3$): δ 8.62 (s, 1 H), 7.95 (d, *J* = 2.3 Hz, 1 H), 7.80 (d, *J* = 8.5 Hz, 2 H), 7.75 (dd, *J* = 8.4 Hz, *J* = 2.3 Hz, 1 H), 7.41 (d, *J* = 8.5 Hz, 2 H), 7.21 (d, *J* = 8.4 Hz, 1 H), 2.46 (s, 3 H), 1.31 (s, 9 H). **^{13}C NMR** (100 MHz, $CDCl_3$): δ 166.3, 155.8, 137.5, 136.7, 131.3, 130.8, 127.2, 125.7, 125.1, 124.0, 118.0, 112.9, 35.0, 31.2, 19.9. **HRMS** (ESI): Calcd for $C_{19}H_{21}N_2O$ [M+H]: 293.1654; Found: 293.1657.

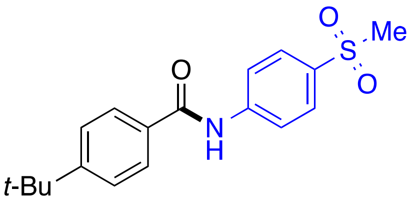

4-(*tert*-Butyl)-*N*-(4-(methylsulfonyl)phenyl)benzamide (5h). Following the general procedure B, the

title compound was prepared using methyl 4-(*tert*-butyl)benzoate (1 equiv, 0.50 mmol, 96 mg) and 1-(methylsulfonyl)-4-nitrobenzene (1.3 equiv, 0.65 mmol, 131 mg), Ni(glyme)Cl_2 (10 mol %, 11.0 mg), and phen (10 mol %, 9.0 mg). The crude product was purified by preparative TLC using hexanes/CH_2Cl_2 (1:1) as an eluent to afford the title compound (**5h**) as off-white solid (102 mg, 62%). **^{1}H NMR** (400 MHz, $CDCl_3$): δ 8.60 (s, 1 H), 7.88-7.80 (ovrlp, 6 H), 7.47 (d, *J* = 8.2 Hz, 2 H), 3.02 (s, 3 H), 1.33 (s, 9 H). **^{13}C NMR** (100 MHz, $CDCl_3$): δ 166.3, 156.2, 143.4, 135.1, 131.2, 128.6, 127.3, 125.9, 120.3, 44.8, 35.1, 31.2. **HRMS** (ESI): Calcd for $C_{18}H_{22}NO_3S$ [M+H]: 332.1320; Found: 332.1317.

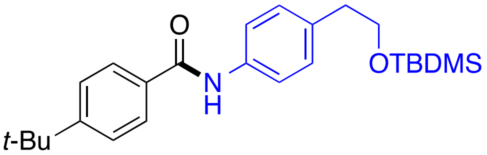

4-(*tert*-Butyl)-*N*-(4-(2-((*tert*-butyldimethylsilyl)oxy)ethyl)phenyl)benzamide (5i). Following the general procedure B, the title compound was prepared using methyl 4-(*tert*-butyl)benzoate (1 equiv, 0.50 mmol, 96 mg) and *tert*-butyldimethyl(4-nitrophenethoxy)silane (1.5 equiv, 0.75 mmol, 211 mg), Ni(glyme)Cl_2 (15 mol %, 16.5 mg), and phen (15 mol %, 13.5 mg). The crude product was purified by preparative TLC using hexanes/CH_2Cl_2 (3:1) as an eluent to afford the title compound (**5i**) as viscous brown oil (84 mg, 41%). **^{1}H NMR** (400 MHz, $CDCl_3$): δ 7.86 (s, 1 H), 7.79 (d, *J* = 8.5 Hz, 2 H), 7.55 (d, *J* = 8.4 Hz, 2 H), 7.47 (d, *J* = 8.5 Hz, 2 H), 7.19 (d, *J* = 8.4 Hz, 2 H), 3.79 (t, *J* = 7.0 Hz, 2 H), 2.80 (t, *J* = 7.0 Hz, 2 H), 1.34 (s, 9 H), 0.88 (9 H), -0.00 (s, 6 H). **^{13}C NMR** (100 MHz, $CDCl_3$): δ 165.7, 155.4, 136.3, 135.5, 132.3, 129.8, 127.0, 125.8, 120.2, 64.6, 39.2, 35.1, 31.3, 26.1, 18.5. **HRMS** (ESI): Calcd for $C_{25}H_{38}NO_2Si$ [M+H]: 412.2672; Found: 412.2653.

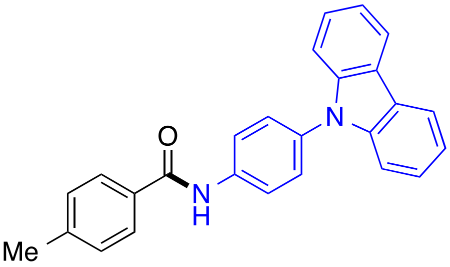

***N*-(4-(9*H*-Carbazol-9-yl)phenyl)-4-methylbenzamide (5j).** Following the general procedure B, the title compound was prepared using methyl 4-methylbenzoate (1 equiv, 0.50 mmol, 75 mg), 9-(4-nitrophenyl)-9*H*-carbazole (1.3 equiv, 0.65 mmol, 187 mg), Ni(glyme)Cl_2 (10 mol %, 11.0 mg), and phen (10 mol %, 9.0 mg). The crude product was purified by preparative TLC using hexanes/CH_2Cl_2 (3:1) as an eluent to afford the title compound (**5j**) as deep-brown solid (120 mg, 64%). **^{1}H NMR** (400 MHz, $CDCl_3$): δ 8.14 (d, *J* = 7.8 Hz, 2 H), 7.98 (s, 1 H), 7.88 (d, *J* = 8.8 Hz, 2 H), 7.83 (d, *J* = 8.2 Hz, 2 H), 7.55 (d, *J* = 8.7 Hz, 2 H), 7.43-7.38 (ovrlp, 4 H), 7.32 (d, *J* = 8.2 Hz, 2 H), 7.30-7.26 (m, 2 H), 2.45 (s, 3 H). **^{13}C NMR** (100 MHz, $CDCl_3$): δ 166.1, 142.8, 141.1, 137.4, 133.8, 131.9, 129.6, 127.9, 127.3, 126.1, 123.4, 121.7, 120.4, 120.0, 109.8, 21.6. **HRMS** (ESI): Calcd for $C_{26}H_{21}N_2O$ [M+H]: 377.1654; Found: 377.1656.

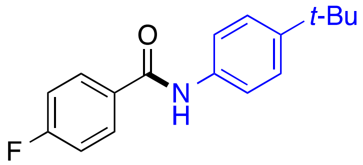

***N*-(4-(*tert*-Butyl)phenyl)-4-fluorobenzamide (5k).** Following the general procedure B, the title compound was prepared using ethyl 4-fluorobenzoate (0.50 mmol, 84 mg) and 1-(*tert*-butyl)-4-nitrobenzene (0.65 mmol, 117 mg). The crude product was purified by preparative TLC using hexanes/CH_2Cl_2 (5:1) as an eluent to afford the title compound (**5k**) as off-white solid (116 mg, 86%). **^{1}H NMR** (400 MHz, $CDCl_3$): δ 7.95 (s, 1 H), 7.85 (dd, $^3J_{HH}$ = 8.1 Hz, $^4J_{CF}$ = 5.6 Hz, 2 H), 7.53 (d, J = 8.4 Hz, 2 H), 7.36 (d, J = 8.4 Hz, 2 H), 7.10 (dd, $^3J_{HH}$ = 8.1 Hz, $^3J_{CF}$ = 8.1 Hz, 2 H), 1.32 (s, 9 H). **^{13}C NMR** (100 MHz, $CDCl_3$): δ 164.93 (d, $^1J_{CF}$ = 250.9 Hz), 164.91, 147.9, 135.3, 131.3 (d, $^4J_{CF}$ = 3.1 Hz), 129.6 (d, $^3J_{CF}$ = 8.9 Hz), 126.0, 120.4, 115.9 (d, $^2J_{CF}$ = 21.8 Hz), 34.6, 31.5. **HRMS** (ESI): Calcd for $C_{17}H_{18}FNONa$ [M+Na]: 294.1270; Found: 294.1273.

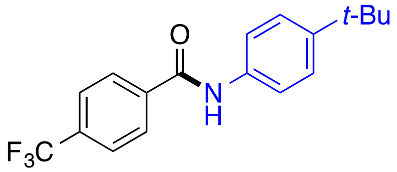

***N*-(4-(*tert*-Butyl)phenyl)-4-(trifluoromethyl)benzamide (5l).** Following the general procedure B, the title compound was prepared using ethyl 4-(trifluoromethyl)benzoate (0.50 mmol, 102 mg) and 1-(*tert*-butyl)-4-nitrobenzene (0.60 mmol, 117 mg). The crude product was purified by preparative TLC using hexanes/CH_2Cl_2 (5:1) as an eluent to afford the title compound (**5l**) as brown solid (134 mg, 84%). **^{1}H NMR** (400 MHz, $CDCl_3$): δ 8.69 (s, 1 H), 7.85 (d, J = 7.9 Hz, 2 H), 7.55-7.52 (ovrlp, 4 H), 7.31 (d, J = 8.4 Hz, 2 H), 1.30 (s, 9 H). **^{13}C NMR** (100 MHz, $CDCl_3$): δ 165.2, 148.2, 138.3, 135.1, 133.3 (q, $^2J_{CF}$ = 32.5 Hz), 127.8, 125.9, 125.6 (q, $^3J_{CF}$ = 3.7 Hz), 123.7 (q, $^1J_{CF}$ = 270.9 Hz), 120.8, 34.5, 31.4. **HRMS** (ESI): Calcd for $C_{18}H_{19}F_3NO$ [M+H]: 322.1419; Found: 322.1422.

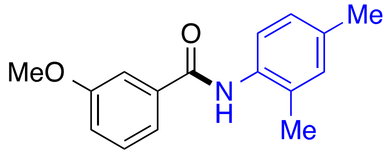

***N*-(2,4-Dimethylphenyl)-3-methoxybenzamide (5m).** Following the general procedure B, the title compound was prepared using ethyl 3-methoxybenzoate (1 equiv, 0.50 mmol, 90 mg), 2,4-dimethyl-1-nitrobenzene (1.5 equiv, 0.75 mmol, 113 mg), Ni(glyme)Cl_2 (10 mol %, 11.0 mg), and phen (10 mol %, 9.0 mg). The crude product was purified by preparative TLC using hexanes/CH_2Cl_2 (3:1) as an eluent to afford the title compound (**5m**) as pale-brown solid (76 mg, 60%). **^{1}H NMR** (400 MHz, $CDCl_3$): δ 7.73 (d, J = 8.1 Hz, 1 H), 7.63 (s, 1 H), 7.45 (s, 1 H), 7.40-7.35 (ovrlp, 2 H), 7.09-7.04 (ovrlp, 3 H), 3.86 (s, 3 H), 2.31 (s, 3 H), 2.28 (s, 3 H). **^{13}C NMR** (100 MHz, $CDCl_3$): δ 165.7, 160.1, 136.7, 135.3, 133.2, 131.4, 129.9, 129.8, 127.5, 123.6, 118.8, 118.0, 112.7, 55.6, 21.0, 17.9. **HRMS**

(ESI): Calcd for $C_{16}H_{18}NO_2$ [M+H]: 256.1338; Found: 256.1339.

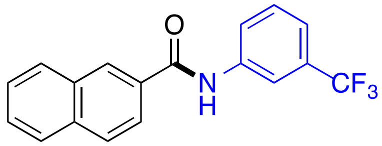

***N*-(3-(Trifluoromethyl)phenyl)-2-naphthamide (5n).** Following the general procedure B, the title compound was prepared using methyl 2-naphthoate (0.50 mmol, 93 mg) and 1-nitro-3-(trifluoromethyl)benzene (0.65 mmol, 124 mg). The crude product was purified by preparative TLC using hexanes/CH_2Cl_2 (5:1) as an eluent to afford the title compound (**5n**) as off-white solid (122 mg, 77%). **^{1}H NMR** (400 MHz, $CDCl_3$): δ 8.33 (s, 1 H), 8.27 (s, 1 H), 7.98 (s, 1 H), 7.93-7.85 (ovrlp, 5 H), 7.60-7.52 (ovrlp, 2 H), 7.47 (t, J = 8.0 Hz, 1 H), 7.40 (d, J = 7.8 Hz, 1 H). **^{13}C NMR** (100 MHz, $CDCl_3$): δ 138.7, 135.1, 132.7, 131.7, 131.6 (q, $^2J_{CF}$ = 32.3 Hz), 129.8, 129.1, 129.0, 128.3, 128.1, 128.0, 127.8, 127.2 (q, $^1J_{CF}$ = 270.6 Hz), 123.52, 123.46 (q, $^4J_{CF}$ = 0.8 Hz), 121.2 (q, $^3J_{CF}$ = 3.8 Hz), 117.1 (q, $^3J_{CF}$ = 4.0 Hz). **HRMS** (ESI): Calcd for $C_{18}H_{13}F_3NO$ [M+H]: 316.0952; Found: 316.0943.

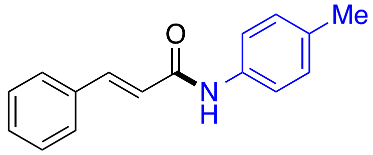

***N*-(*p*-Tolyl)cinnamamide (5o).** Following the general procedure B, the title compound was prepared using methyl cinnamate (0.50 mmol, 81 mg) and 4-nitrotoluene (0.65 mmol, 89 mg). The crude product was purified by preparative TLC using hexanes/CH_2Cl_2 (1:1) as an eluent to afford the title compound (**5o**) as off-white solid (102 mg, 86%). **^{1}H NMR** (400 MHz, $CDCl_3$): δ 8.35 (s, 1 H), 7.70 (d, J = 15.6 Hz, 1 H), 7.54 (d, J = 8.0 Hz, 2 H), 7.37 (d, J = 7.1 Hz, 2 H), 7.31-7.22 (ovrlp, 3 H), 7.07 (d, J = 7.9 Hz, 2 H), 6.65 (d, J = 15.5 Hz, 1 H), 2.27 (s, 3 H). **^{13}C NMR** (100 MHz, $CDCl_3$): δ 164.6, 142.0, 135.7, 134.8, 134.1, 129.8, 129.6, 128.8, 128.0, 121.4, 120.5, 21.0. **HRMS** (ESI): Calcd for $C_{16}H_{16}NO$ [M+H]: 238.1226; Found: 238.1230.

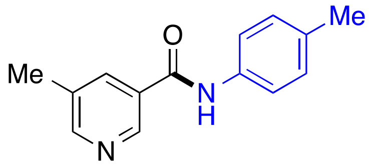

5-Methyl-*N*-(*p*-tolyl)nicotinamide (5p). Following the general procedure B, the title compound was prepared using methyl 5-methylnicotinate (0.50 mmol, 76 mg) and 4-nitrotoluene (0.60 mmol, 89 mg). The crude product was purified by preparative TLC using hexanes/CH_2Cl_2 (1:1) as an eluent to afford the title compound (**5p**) as off-white solid (88 mg, 78%). **^{1}H NMR** (400 MHz, $CDCl_3$): δ 8.83 (s, 1 H), 8.75 (s, 1 H), 8.48 (s, 1 H), 7.94 (s, 1 H), 7.49 (d, J = 8.0 Hz, 2 H), 7.11 (d, J = 8.0 Hz, 2 H), 2.32 (ovrlp, 6 H). **^{13}C NMR** (100 MHz, $CDCl_3$): δ 164.4, 152.6, 145.1, 136.0, 135.2, 134.6, 133.6, 130.7,

129.6, 120.8, 21.0, 18.4. **HRMS** (ESI): Calcd for $C_{14}H_{15}N_2O$ [M+H]: 227.1184; Found: 227.1188.

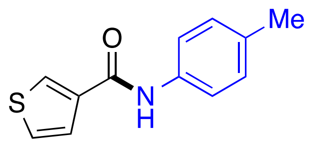

***N*-(*p*-Tolyl)thiophene-3-carboxamide (5q).** Following the general procedure B, the title compound was prepared using ethyl thiophene-3-carboxylate (0.50 mmol, 78 mg) and 4-nitrotoluene (0.60 mmol, 89 mg). The crude product was purified by preparative TLC using hexanes/CH_2Cl_2 (5:1) as an eluent to afford the title compound (**5q**) as off-white solid (90 mg, 83%). ^{1}**H NMR** (400 MHz, $CDCl_3$): δ 7.97-7.92 (ovrlp, 2 H), 7.49-7.46 (ovrlp, 3 H), 7.32 (dd, *J* = 5.0 Hz, *J* = 2.9 Hz, 1 H), 7.12 (d, *J* = 8.0 Hz, 2 H), 2.31 (s, 3 H). ^{13}**C NMR** (100 MHz, $CDCl_3$): δ 161.4, 138.0, 135.3, 134.3, 129.6, 128.7, 126.7, 126.4, 120.7, 21.0. **HRMS** (ESI): Calcd for $C_{12}H_{12}NOS$ [M+H]: 218.0646; Found: 218.0635.

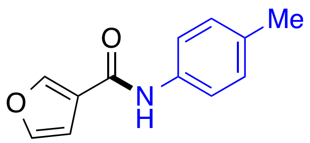

***N*-(*p*-Tolyl)furan-3-carboxamide (5r).** Following the general procedure B, the title compound was prepared using ethyl furan-3-carboxylate (0.50 mmol, 70 mg) and 4-nitrotoluene (0.65 mmol, 89 mg). The crude product was purified by preparative TLC using hexanes/CH_2Cl_2 (5:1) as an eluent to afford the title compound (**5r**) as pale-brown solid (66 mg, 66%). ^{1}**H NMR** (400 MHz, $CDCl_3$): δ 8.14 (s, 1 H), 8.00 (s, 1 H), 7.45 (d, *J* = 8.0 Hz, 2 H), 7.39 (s, 1 H), 7.08 (d, *J* = 8.0 Hz, 2 H), 6.74 (s, 1 H), 2.29 (s, 3 H). ^{13}**C NMR** (100 MHz, $CDCl_3$): δ 161.2, 145.3, 143.8, 135.2, 134.3, 129.5, 123.1, 120.9, 108.7, 21.0. **HRMS** (ESI): Calcd for $C_{12}H_{12}NO_2$ [M+H]: 202.0874; Found: 202.0863.

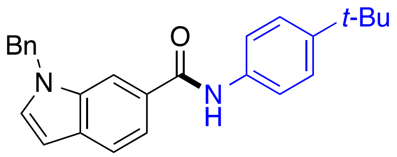

1-Benzyl-*N*-(4-(*tert*-butyl)phenyl)-1*H*-indole-6-carboxamide (5s). Following the general procedure B, the title compound was prepared using methyl 1-benzyl-1*H*-indole-6-carboxylate (0.50 mmol, 126 mg) and 1-(*tert*-butyl)-4-nitrobenzene (0.65 mmol, 116 mg). The crude product was purified by preparative TLC using hexanes/CH_2Cl_2 (3:1) as an eluent to afford the title compound (**5s**) as off-white solid (96 mg, 50%). ^{1}**H NMR** (400 MHz, $CDCl_3$): δ 7.98 (s, 1 H), 7.95 (s, 1 H), 7.66 (d, *J* = 8.1 Hz, 1 H), 5.57-7.50 (ovrlp, 3 H), 7.35 (d, *J* = 8.1 Hz, 2 H), 7.29-7.22 (ovrlp, 4 H), 7.08 (d, *J* = 6.3 Hz, 2 H), 6.58 (s, 1 H), 5.31 (s, 2 H), 1.31 (s, 9 H). ^{13}**C NMR** (100 MHz, $CDCl_3$): δ 166.6, 147.3, 137.0, 136.2, 135.8, 131.5, 131.2, 129.0, 128.4, 127.9, 127.0, 125.9, 121.0, 120.0, 117.6, 110.3, 102.2, 50.3, 34.5, 31.5. **HRMS** (ESI): Calcd for $C_{26}H_{27}N_2O$ [M+H]: 383.2123; Found: 383.2126.

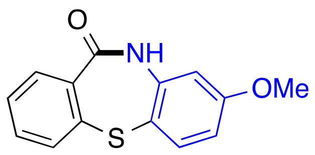

8-Methoxydibenzo[*b*,*f*][1,4]thiazepin-11(10*H*)-one (5t).

(i) 0.50 mmol. Following the general procedure B, the title compound was prepared using *n*-pentyl 2-((4-methoxy-2-nitrophenyl)thio)benzoate (**S6**, 1 equiv, 0.50 mmol, 188 mg), Ni(glyme)Cl_2 (10 mol %, 11.0 mg), and phen (10 mol %, 9.0 mg) under the reaction temperature of 90 ^oC. The crude product was purified by preparative TLC using CH_2Cl_2 as an eluent to afford the title compound (**5t**) as brown solid (87 mg, 68%). **^{1}H NMR** (400 MHz, DMSO-d_6): δ 10.61 (s, 1 H), 7.66 (dd, J = 7.3 Hz, J = 1.4 Hz, 1 H), 7.50 (td, J = 7.6 Hz, J = 1.5 Hz, 1 H), 7.46-7.40 (ovrlp, 3 H), 6.80 (d, J = 2.7 Hz, 1 H), 6.73 (dd, J = 8.6 Hz, J = 2.7 Hz, 1 H), 3.72 (s, 3 H). **^{13}C NMR** (100 MHz, DMSO-d_6): δ 168.5, 160.3, 141.1, 138.0, 136.8, 133.4, 131.9, 131.2, 131.1, 128.8, 119.9, 111.2, 108.6, 55.4. **HRMS** (ESI): Calcd for $C_{14}H_{12}NO_2S$ [M+H]: 258.0583; Found: 258.0576.

(ii) Gram scale. Following the general procedure B, the title compound was prepared using *n*-pentyl 2-((4-methoxy-2-nitrophenyl)thio)benzoate (1 equiv, 2.93 mmol, 1.10 g), Ni(glyme)Cl_2 (10 mol %, 64 mg), and phen (10 mol %, 53 mg), Zn (4 equiv, 11.7 mmol, 767 mg), TMSCl (2 equiv, 5.86 mmol, 748 μL), and NMP (12 mL) under the reaction temperature of 90 ^oC. Spectral and analytical data were identical to those reported for the same compound above.

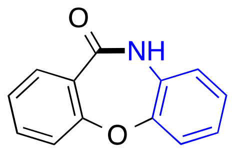

Dibenzo[*b*,*f*][1,4]oxazepin-11(10*H*)-one (5u). Following the general procedure B, the title compound was prepared using methyl 2-(2-nitrophenoxy)benzoate (0.50 mmol, 137 mg), Ni(glyme)Cl_2 (10 mol %, 11.0 mg), and phen (10 mol %, 9.0 mg). The crude product was purified by preparative TLC using CH_2Cl_2 as an eluent to afford the title compound (**6i**) as brown solid (79 mg, 74%). **^{1}H NMR** (400 MHz, $CDCl_3$): δ 9.21 (s, 1 H), 7.96 (d, J = 7.4 Hz, 1 H), 7.53 (d, J = 7.3 Hz, 1 H), 7.31-7.22 (ovrlp, 3 H), 7.18-7.10 (ovrlp, 3 H). **^{13}C NMR** (100 MHz, $CDCl_3$): δ 167.9, 159.8, 151.1, 134.6, 132.1, 130.8, 126.1, 126.0, 125.4, 125.3, 121.8, 121.5, 121.0. **HRMS** (ESI): Calcd for $C_{13}H_{10}NO_2$ [M+H]: 212.0712; Found: 212.0714.

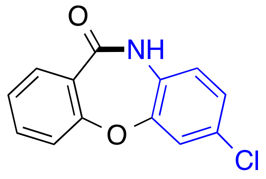

7-Chlorodibenzo[*b*,*f*][1,4]oxazepin-11(10*H*)-one (5v). Following the general procedure B, the title compound was prepared using methyl 2-(5-chloro-2-nitrophenoxy)benzoate (0.50 mmol, 154 mg), Ni(glyme)Cl_2 (10 mol %, 11.0 mg), and phen (10 mol %, 9.0 mg) under the reaction temperature of 90 ^oC. The crude product was purified by recrystallization (CH_2Cl_2/hexanes) as solvent to afford the title compound (**5u**) as deep-brown solid (65 mg, 53%). ^{1}**H NMR** (400 MHz, DMSO-d_6): δ 10.61 (s, 1 H), 7.77 (d, *J* = 7.3 Hz, 1 H), 7.63 (t, *J* = 7.4 Hz, 1 H), 7.50 (s, 1 H), 7.38-7.31 (ovrlp, 2 H), 7.26 (d, *J* = 7.9 Hz, 1 H), 7.17 (d, *J* = 8.4 Hz, 1 H). ^{13}**C NMR** (100 MHz, DMSO-d_6): δ 165.1, 158.1, 150.3, 134.3, 131.1, 130.1, 128.0, 125.7, 125.5, 125.1, 122.4, 121.2, 120.4. **HRMS** (ESI): Calcd for $C_{13}H_9ClNO_2$ [M+H]: 246.0322; Found: 246.0322.

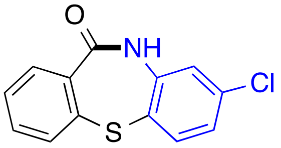

8-Chlorodibenzo[*b*,*f*][1,4]thiazepin-11(10*H*)-one (6a). Following the general procedure B, the title compound was prepared using methyl 2-((4-chloro-2-nitrophenyl)thio)benzoate (0.50 mmol, 162 mg), Ni(glyme)Cl_2 (10 mol %, 11.0 mg), and phen (10 mol %, 9.0 mg) under the reaction temperature of 90 ^oC. The crude product was purified by preparative TLC using hexanes/CH_2Cl_2 (1:1) as an eluent to afford the title compound (**6b**) as brown solid (61 mg, 47%). ^{1}**H NMR** (400 MHz, DMSO-d_6): δ 10.76 (s, 1 H), 7.68 (dd, *J* = 7.4 Hz, *J* =1.4 Hz, 1 H), 7.57 (d, *J* = 8.3 Hz, 1 H), 7.54-7.44 (ovrlp, 3 H), 7.27 (d, *J* = 2.0 Hz, 1 H), 7.21 (dd, *J* = 8.3 Hz, *J* = 2.1 Hz, 1 H). ^{13}**C NMR** (100 MHz, DMSO-d_6): δ 167.9, 141.0, 137.3, 135.3, 133.7, 132.0, 131.2, 131.1, 128.9, 127.5, 124.9, 122.3. **HRMS** (ESI): Calcd for $C_{13}H_9ClNOS$ [M+H]: 262.0093; Found: 262.0095.

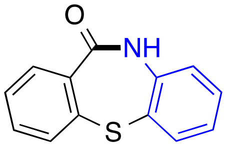

Dibenzo[*b*,*f*][1,4]thiazepin-11(10*H*)-one (6b). Following the general procedure B, the title compound was prepared using methyl 2-((2-nitrophenyl)thio)benzoate (0.50 mmol, 145 mg), Ni(glyme)Cl_2 (10 mol %, 11.0 mg), and phen (10 mol %, 9.0 mg) under the reaction temperature of 90 ^oC. The crude product was purified by preparative TLC using CH_2Cl_2 as an eluent to afford the title compound (**6a**) as brown solid (76 mg, 67%). ^{1}**H NMR** (400 MHz, DMSO-d_6): δ 10.69 (s, 1 H), 7.68 (dd, *J* = 7.1 Hz, *J* = 1.2 Hz, 1 H), 7.57-7.52 (ovrlp, 2 H), 7.50-7.42 (ovrlp, 2 H), 7.36 (t, *J* = 7.3 Hz, 1 H), 7.23 (d, *J* = 7.7 Hz, 1 H), 7.14 (t, *J* = 7.3 Hz, 1 H). ^{13}**C NMR** (100 MHz, DMSO-d_6): δ 168.4, 139.9, 137.8, 136.3, 132.5, 132.0, 131.4, 131.3, 129.8, 129.0, 128.9, 125.4, 123.2. **HRMS** (ESI): Calcd for $C_{13}H_{10}NOS$ [M+H]: 228.0483; Found: 228.0485.

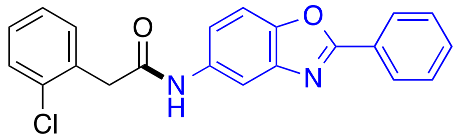

2-(2-Chlorophenyl)-*N*-(2-phenylbenzo[*d*]oxazol-5-yl)acetamide (6c). Following the general procedure A, the title compound was prepared using methyl 2-(2-chlorophenyl)acetate (1 equiv, 0.50 mmol, 92 mg), 5-nitro-2-phenylbenzo[*d*]oxazole (1.2 equiv, 0.60 mmol, 144 mg), Ni(glyme)Cl_2 (10 mol %, 11.0 mg), and phen (10 mol %, 9.0 mg). The crude product was purified by preparative TLC using hexanes/CH_2Cl_2 (3:1) as an eluent to afford the title compound (**6d**) as deep brown solid (117 mg, 65%). ^{1}**H NMR** (400 MHz, $CDCl_3$): δ 8.20 (d, J = 5.9 Hz, 2 H), 7.86 (s, 1 H), 7.54-7.47 (ovrlp, 3 H), 7,45-7.31 (ovrlp, 7 H), 3.76 (s, 2 H). ^{13}**C NMR** (100 MHz, $CDCl_3$): δ 169.4, 164.0, 147.8, 142.6, 134.7, 134.6, 131.7, 129.7, 129.4, 129.0, 127.81, 127.76, 127.1, 118.4, 111.9, 110.5, 44.9. **HRMS** (ESI): Calcd for $C_{21}H_{16}ClN_2O_2$ [M+H]: 363.0900; Found: 363.0898.

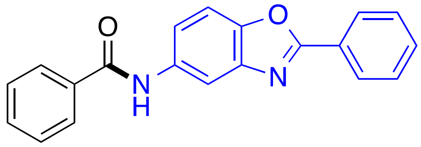

***N*-(2-Phenylbenzo[*d*]oxazol-5-yl)benzamide (6d).** Following the general procedure B, the title compound was prepared using methyl benzoate (1 equiv, 0.35 mmol, 48 mg), 5-nitro-2-phenylbenzo[*d*]oxazole (1.3 equiv, 0.455 mmol, 109 mg), Ni(glyme)Cl_2 (10 mol %, 7.7 mg), and phen (10 mol %, 6.3 mg), Zn (4 equiv, 1.4 mmol, 92 mg), TMSCl (2 equiv, 0.70 mmol, 89 μL), and NMP (0.70 mL). The crude product was purified by preparative TLC using hexanes/CH_2Cl_2 (3:1) as an eluent to afford the title compound (**6e**) as deep-brown solid (66 mg, 60%). ^{1}**H NMR** (400 MHz, $CDCl_3$): δ 8.23 (d, J = 4.6 Hz, 2 H), 8.12 (s, 1 H), 8.03 (s, 1 H), 7.89 (d, J = 6.7 Hz, 2 H), 7.65 (d, J = 7.7 Hz, 1 H), 7.57-7.44 (ovrlp, 7 H). ^{13}**C NMR** (100 MHz, $CDCl_3$): δ 166.1, 164.1, 148.0, 142.7, 135.01, 134.98, 132.0, 131.8, 129.0, 128.9, 127.8, 127.2, 127.1, 118.9, 112.3, 110.7. **HRMS** (ESI): Calcd for $C_{20}H_{15}N_2O_2$ [M+H]: 315.1133; Found: 315.1135.

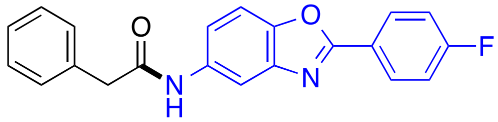

***N*-(2-(4-Fluorophenyl)benzo[*d*]oxazol-5-yl)-2-phenylacetamide (6e).** Following the general procedure A, the title compound was prepared using methyl 2-phenylacetate (1 equiv, 0.50 mmol, 75 mg), 2-(4-fluorophenyl)-5-nitrobenzo[*d*]oxazole (1.3 equiv, 0.65 mmol, 168 mg), Ni(glyme)Cl_2 (10 mol %, 11.0 mg), and phen (10 mol %, 9.0 mg) under the reaction temperature of 120 ^{o}C. The crude product was purified by preparative TLC using hexanes/CH_2Cl_2 (3:1) as an eluent to afford the title compound (**6f**) as pale-brown solid (120 mg, 69%). ^{1}**H NMR** (400 MHz, $CDCl_3$): δ 8.22 (dd, $^{3}J_{HH}$ = 8.7 Hz, $^{4}J_{HF}$ = 5.4 Hz, 2 H), 7.86 (d, J = 1.6 Hz, 1 H), 7.46-7.41 (ovrlp, 3 H), 7.38-7.34 (ovrlp, 4 H), 7.25 (s, 1 H), 7.20 (dd, $^{3}J_{HH}$ = 8.6 Hz, $^{3}J_{HF}$ = 8.6 Hz, 2 H), 3.79 (s, 2 H). ^{13}**C NMR** (100 MHz, $CDCl_3$): δ 169.3, 165.0 (d, $^{1}J_{CF}$ = 251.4 Hz), 163.2, 147.9, 142.6, 134.8, 134.5, 130.0 (d, $^{3}J_{CF}$ = 8.9 Hz), 129.7, 129.5, 127.9, 123.5 (d, $^{4}J_{CF}$ = 2.9 Hz), 118.3, 116.3 (d, $^{2}J_{CF}$ = 22.1 Hz), 111.9, 110.5, 44.9. **HRMS**

(ESI): Calcd for $C_{21}H_{16}FN_2O_2$ [M+H]: 347.1190; Found: 347.1196.

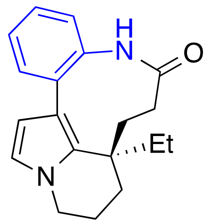

(*R*)-3a-Ethyl-2,3,3a,4,5,7-hexahydrobenzo[2,3]azonino[6,5,4-*hi*]indolizin-6(1*H*)-one ((-)-Rhazinilam, 6f).[16] Following the general procedure A, the title compound was prepared using methyl (*R*)-3-(8-ethyl-1-(2-nitrophenyl)-5,6,7,8-tetrahydroindolizin-8-yl)propanoate[16] (1 equiv, 0.0269 mmol, 9.6 mg), Ni(glyme)Cl_2 (20 mol %, 1.2 mg), phen (20 mol %, 1.0 mg), Zn (4 equiv, 0.108 mmol, 7 mg), TMSCl (2 equiv, 0.0538 mmol, 7 μL), and NMP (0.3 mL) in a 2 mL-Teflon-screw cap test tube under the reaction temperature of 120 ^{o}C. The crude product was purified by preparative TLC using hexanes/CH_2Cl_2 (3:1) as an eluent to afford the title compound (**6c**) as off-white solid (4.7 mg, 59%). **^{1}H NMR** (400 MHz, $CDCl_3$): δ 7.43 (dd, *J* = 7.2 Hz, *J* = 1.7 Hz, 1 H), 7.37-7.28 (ovrlp, 2 H), 7.21 (d, *J* = 7.7 Hz, 1 H), 6.56 (s, 1 H), 6.51 (d, *J* = 2.6 Hz, 1 H), 5.76 (d, *J* = 2.6 Hz, 1 H), 4.01 (dd, *J* = 11.8 Hz, *J* = 5.5 Hz, 1 H), 3.79 (td, *J* = 12.1 Hz, *J* = 4.7 Hz, 1 H), 2.50-2.31 (m, 1 H), 2.31-2.17 (m, 1 H), 1.98 (dd, *J* = 13.0 Hz, *J* = 7.8 Hz, 1 H), 1.89-1.82 (m, 1 H), 1.72 (td, *J* = 13.5 Hz, *J* = 3.1 Hz, 1 H), 1.53-1.42 (ovrlp, 3 H), 1.30-1.20 (m, 1 H), 0.72 (t, *J* = 7.4 Hz, 3 H). **HRMS** (ESI): Calcd for $C_{19}H_{23}N_2O$ [M+H]: 295.1805; Found: 295.1795.

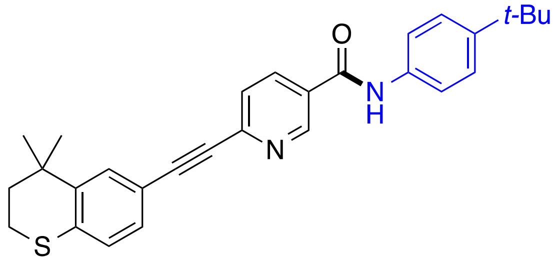

***N*-(4-(*tert*-Butyl)phenyl)-6-((4,4-dimethylthiochroman-6-yl)ethynyl)nicotinamide (6g).** Following the general procedure B, the title compound was prepared using ethyl 6-((4,4-dimethylthiochroman-6-yl)ethynyl)nicotinate (Tazarotene, 1 equiv, 0.10 mmol, 35 mg), 1-(*tert*-butyl)-4-nitrobenzene (1.5 equiv, 0.15 mmol, 27 mg), Ni(glyme)Cl_2 (15 mol %, 3.3 mg), and phen (15 mol %, 2.7 mg), Zn (4 equiv, 0.40 mmol, 26 mg), TMSCl (2 equiv, 0.20 mmol, 26 μL), and NMP (0.30 mL). The crude product was purified by preparative TLC using hexanes/CH_2Cl_2 (3:1) as an eluent to afford the title compound (**6g**) as pale-brown solid (28 mg, 62%). **^{1}H NMR** (400 MHz, $CDCl_3$): δ 9.05 (s, 1 H), 8.17 (dd, *J* = 8.1 Hz, *J* = 2.3 Hz, 1 H), 8.05 (s, 1 H), 7.61-7.55 (ovrlp, 4 H), 7.39 (d, *J* = 8.7 Hz, 2 H), 7.23 (dd, *J* = 8.2 Hz, *J* = 1.8 Hz, 1 H), 7.07 (d, *J* = 8.1 Hz, 1 H), 3.05 (d, *J* = 6.0 Hz, 2 H), 1.95 (d, *J* = 6.1 Hz, 2 H), 1.33-1.32 (ovrlp, 15 H). **^{13}C NMR** (100 MHz, $CDCl_3$): δ 163.4, 148.3, 146.4, 142.3, 135.6, 135.D 2, 134.9, 130.6, 129.5, 129.1, 126.83, 126.77, 126.1, 120.4, 116.9, 93.1, 87.8, 37.2, 34.6, 33.1, 31.5, 30.1, 23.4. **HRMS** (ESI): Calcd for $C_{29}H_{31}N_2OS$ [M+H]: 455.2157; Found: 455.2157.

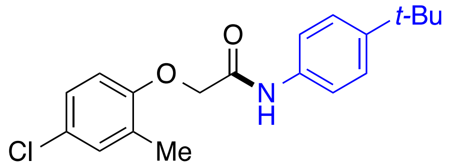

***N*-(4-(*tert*-Butyl)phenyl)-2-(4-chloro-2-methylphenoxy)acetamide (6h).** Following the general procedure A, the title compound was prepared using methyl 2-(4-chloro-2-methylphenoxy)acetate (MCPA-methyl, 1 equiv, 0.25 mmol, 54 mg), 1-(*tert*-butyl)-4-nitrobenzene (1.5 equiv, 0.375 mmol, 67 mg), Ni(glyme)Cl_2 (15 mol %, 8.3 mg), and phen (15 mol %, 6.8 mg), Zn (4 equiv, 1.0 mmol, 65.4 mg), TMSCl (2 equiv, 0.50 mmol, 64 μL), and NMP (0.50 mL). The crude product was purified by preparative TLC using hexanes/CH_2Cl_2 (5:1) as an eluent to afford the title compound (**6h**) as off-white solid (40 mg, 48%). **^{1}H NMR** (400 MHz, $CDCl_3$): δ 8.20 (s, 1 H), 7.48 (d, *J* = 8.7 Hz, 2 H), 7.37 (d, *J* = 8.7 Hz, 2 H), 7.18 (d, *J* = 2.2 Hz, 1 H), 7.14 (dd, *J* = 8.6 Hz, *J* = 2.4 Hz, 1 H), 6.75 (d, *J* = 8.6 Hz, 1 H), 4.57 (s, 2 H), 2.33 (s, 3 H), 1.31 (s, 9 H). **^{13}C NMR** (100 MHz, $CDCl_3$): δ 166.0, 154.0, 148.2, 134.2, 131.1, 128.6, 127.15, 127.09, 126.1, 120.0, 113.2, 68.2, 34.6, 31.5, 16.4. **HRMS** (ESI): Calcd for $C_{19}H_{23}ClNO_2$ [M+H]: 332.1417; Found: 332.1418.

Screening Conditions for Reaction between Methyl Decanoate and Various Nitrobenzene-derived Intermediates.

(i) General Considerations. For all intermediates, the reaction was performed with 7.5 mol% Ni(glyme)Cl_2 / phen catalyst loading. 0.5 M ester (= 1 equiv) in 0.5 ml NMP was used. The reactions were performed with naphthalene as internal standard. Conversions and yields were analyzed by preparation of GC samples from the crude reaction mixtures and measuring the ratio of peak area of the ester and amide versus naphthalene. A peak area to concentration conversion factor of 0.87 was found through a calibration curve of isolated product amide versus naphthalene and this was used for determination of yield. Errors as large as 5 percentage points between independent experiments could generally be observed.

(ii) Nitrosobenzene. Reactions were set up by varying the loading of zinc (2-4 equiv) and TMSCl (1-2 equiv) systematically (Table S12). At the end of the reaction, 3ml water and 3 ml EtOAc were added and a GC sample was prepared with 50 μL from the top organic layer.

(iii) *N*-Phenylhydroxyamine. Reactions were set up by varying the loading of zinc (2-4 equiv) and TMSCl (1-2 equiv) systematically (Table S13). At the end of the reaction, a few drops of the reaction mixture were added to 1 mL Et_2O. The resulting suspension was shaken and filtered for GC sample preparation. The results show that starting with fewer equivalents than the standard conditions for the reaction with nitrobenzene leads to poorer yields.

(iv) Aniline. Reactions were set up under conditions that might be relevant at a point in the reaction where a reasonable amount of aniline might have formed, including the addition of $ZnCl_2$, as surrogate for the Zn(II) species that should form during the reaction and might influence the reactivity (Table S14). At the end of the reaction, a few drops of the reaction mixture were added to 1 mL Et_2O. The resulting suspension was shaken and filtered for GC sample preparation. Despite a rather thorough screening of potentially relevant conditions, no yield higher than 45% was observed, ruling out aniline as an important intermediate.

(v) Azoxybenzene. Reactions were set up by varying the loading of zinc (1-4 equiv) and TMSCl (0.09-1 equiv) systematically (Table S15). At the end of the reaction, a few drops of the reaction mixture were added to 1 mL Et_2O. The resulting suspension was shaken and filtered for GC sample preparation.

Stoichiometric experiment of 4,4'-difluoroazobenzene

In a nitrogen atmosphere glove box, 71.2 mg Zn (1.1 mmol, 2.2 eq), 219 mg Ni(glyme)Cl_2 (1.0 mmol, 2 eq), 180 mg 1,10-phenanthroline (1.0 mmol, 2 eq), 1.8 mmol NMP (dried by storing over 3Å molecular sieves), and 1.2 mg trimethylsilylchloride (0.015 mmol, 0.022 eq) were added to an oven dried scintillation vial. After stirring for 30 minutes, the mixture had turned from grey to black. 108 mg 4,4'-difluoroazobenzene (0.49 mmol, 1 eq) and 16.6 mg α,α,α-trifluorotoluene (0.114 mmol) were then added to the above solution. After stirring the mixture for 2 hours at room temperature, 0.5 ml was filtered into an oven dried young's NMR tube equipped with a DMSO-d6 capillary. The remainder of the mixture was heated to 90°C for three days. Another 0.5 ml was then filtered into another oven dried young's NMR tube equipped with a DMSO-d6 capillary. To the remainder of the reaction mixture was added 80.8 mg methyl decanoate (0.434 mmol, 1.97 eq) and the resulting mixture was heated to 90°C over night. 20 µl of the reaction mixture was taken for a GC/MS sample and the remainder was filtered into an oven dried young's NMR tube equipped with a DMSO-d6 capillary. ^{19}F{H} NMR (376.3 MHz), referenced to α,α,α-trifluorotoluene (-63.72 ppm, 1.00 integral) RT sample: -111.25 ppm (integral 1.15, free azobenzene), two merged broad peaks at -126.67 ppm and -129.42 ppm (combined integral 0.60). 3d 90°C sample: -131.59 ppm (integral 0.63) along with a few ill-defined peaks. Sample after reaction with ester: -122.45 ppm (integral 1.71, amidate), along with some ill-defined species. GC/MS analysis of the mixture after reaction with ester indicates the presence of the amide product and some aniline side-product, with a ratio of ~10:1 favoring the amide.

Supplementary References

1. Fulmer, G. R., Miller, A. J. M., Sherden, N. H., Gottlieb, H. E., Nudelman, A., Stoltz, B. M., Bercaw, J. E. & Goldberg, K. I. NMR chemical shifts of trace impurities: common laboratory solvents, organics, and gases in deuterated solvents relevant to the organometallic chemist. *Organometallics* **29**, 2176-2179 (2010).

2. Pinnick, H. W. & Lajis, N. H. *N*-Bromosuccinimide oxidation of silyl ethers. *J. Org. Chem.* **43**, 371-372 (1978).

3. Cheung, C. W., Ren, P. & Hu, X. Mild and phosphine-free iron-catalyzed cross-coupling of nonactivated secondary alkyl halides with alkynyl Grignard reagents. *Org. Lett.* **16**, 2566-2569 (2014).

4. Terauchi, J., Kuno, H., Nara, H., Oki, H. & Sato, K (Takeda Pharmaceutical Co. Limited, Japan). Preparation of heterocyclic amides as MMP-13 inhibitors for treating osteoarthritis and rheumatoid arthritis. US Patent 2005105760, Nov 10, 2005.

5. Nagai, K., Nagasawa, K., Takahashi, H., Baba, M., Fujioka, S., Kondoh, E., Tanaka, K. & Itoh, Y. (Sato Pharmaceutical Co. Ltd., Japan). Preparation of ring-fused compounds as inhibitors of transport protein urate transporter 1 (URAT1). US Patent 2012102405, Aug 02, 2012.

6. Santos, P. F., Reis, L. V., Duarte, I., Serrano, J. P., Almeida, P., Oliveira, A. S. & Ferreira, L. F. V. Synthesis and photochemical evaluation of iodinated squarylium cyanine dyes**.** *Helv. Chim. Acta.* **88**, 1135-1143 (2005).

7. Kawashita, Y., Nakamichi, N., Kawabata, H. & Hayashi, M. Direct and practical synthesis of 2-arylbenzoxazoles promoted by activated carbon. *Org. Lett.* **5**, 3713-3715 (2003).

8. Liou, G.-S. & Lin, H.-Y. Synthesis and electrochemical properties of novel aromatic poly(amine−amide)s with anodically highly stable yellow and blue electrochromic behaviors. *Macromolecules* **42**, 125-134 (2009).

9. Kim, M.-H., Kim, S.-H., Ku, S.-K., Park, C.-H., Joe, B.-Y., Chun, K.-W., Ye, I.-H., Choi, J.-H., Ryu, D.-K., Park, J.-S., Lee, H.-C., Choi, J.-S. & Kim, Y.-C (Jeil Pharmaceutical Co., Ltd., S. Korea). Preparation of tricyclic compounds as PARP inhibitors. US Patent 2010056038, May 20, 2010.

10. Obolda, A.; Peng, Q.; He, C.; Zhang, T.; Ren, J.; Ma, H.; Shuai, Z.; Li, F. Triplet-polaron-interaction-induced upconversion from triplet to singlet: a possible way to obtain highly efficient OLEDs. *Adv. Mater.* **28**, 4740-4746 (2016).

11. Vosooghi, M., Arshadi, H., Saeedi, M., Mahdavi, M., Jafapour, F., Shafiee, A. & Foroumadi, A. A novel and efficient route for the synthesis of 5-nitrobenzo[d]oxazole derivatives. *J. Fluor. Chem.* **161**, 83-86 (2014).

12. Rogers, K. & Patzke, H. (Envivo Pharmaceuticals, Inc., USA & MethylGene Inc.) Preparation of

benzo-fused 7-membered heterocyclic compounds and methods for treating cognitive disorders using inhibitors of histone deacetylase. US Patent 2009137462, Nov 12, 2009.

13. Polisetti, D. R., Kodra, J. T., Lau, J., Bloch, P., Valcarce-Lopez, M. C., Blume, N., Guzel, M., Santhosh, K. C., Mjalli, A. M. M., Andrews, R. C.; Subramanian, G., Ankersen, M., Vedso, P., Murray, A.; Jeppesen, L. (Novo Nordisk A/S, Den., Valcarce-Lopez,mariacarmen; et al.) Preparation of thiazolyl aryl ureas as activators of glucokinase. US Patent 2004002481, Jan 08, 2004.

14. Burstein, E. S. (ACADIA Pharmaceuticals Inc., USA). Use of *n*-desmethylclozapine and related compounds as dopamine stabilizing agents useful in the treatment of neuropsychiatric disease. US Patent 2006107948, Oct 12, 2006.

15. Wagh, B. S.; Patil, B. P.; Jain, M. S.; Harak, S. S.; Wagh, S. B. Synthesis and evaluation of antipsychotic activity of 11-(4-aryl-1-piperazinyl)dibenz[b,f][1,4]oxazepines and their 8-chloro analogues. *Heterocycl. Commun.* **13**, 165-172 (2007).

16. Dagoneau, D., Xu, Z., Wang, Q. & Zhu, J. Enantioselective total syntheses of (-)-Rhazinilam, (-)-Leucomidine B, and (+)-Leuconodine F. *Angew. Chem. Int. Ed.* **55**, 760-763 (2016).

17. Sheppeck, J. E., Gilmore, J. L., Dhar, T. G. M.; Xiao, H.-Y.; Wang, J.; Yang, B. V.; Doweyko, L. M. (Bristol-Myers Squibb Company, USA). Indazole compounds as modulators of glucocorticoid receptor, AP-1, and/or NF-κB activity and their preparation, pharmaceutical compositions and use in the treatment of diseases. US Patent 2008057857, May 15, 2008.

18. Binaschi, M., Boldetti, A., Gianni, M., Maggi, C. A., Gensini, M., Bigioni, M., Parlani, M., Giolitti, A., Fratelli, M., Valli, C., Terao, M. & Garattini, E. Antiproliferative and Differentiating Activities of a Novel Series of Histone Deacetylase Inhibitors. *ACS Med. Chem. Lett.* **1**, 411-415 (2010).

19. Calle, M., Lozano, A. E., de La Campa, J. G. & de Abajo, J. Novel aromatic polyimides derived from 5'-*t*-butyl-2'-pivaloylimino-3,4,3'',4''-m-terphenyltetracarboxylic dianhydride with potential application on gas separation processes. *Macromolecules* **43**, 2268-2275 (2010).
